# Antiprotozoal *Nor*-Triterpene Alkaloids from *Buxus sempervirens* L.

**DOI:** 10.3390/antibiotics10060696

**Published:** 2021-06-10

**Authors:** Lara U. Szabó, Marcel Kaiser, Pascal Mäser, Thomas J. Schmidt

**Affiliations:** 1 Institute of Pharmaceutical Biology and Phytochemistry (IPBP), University of Münster, PharmaCampus Corrensstraße 48, D-48149 Münster, Germany; lszabo@uni-muenster.de; 2 Swiss Tropical and Public Health Institute (Swiss TPH), Socinstrasse 57, CH-4051 Basel, Switzerland; marcel.kaiser@unibas.ch (M.K.); pascal.maeser@swisstph.ch (P.M.); 3 University of Basel, Petersplatz 1, CH-4003 Basel, Switzerland

**Keywords:** *Buxus sempervirens* L., *nor*-cycloartane alkaloids, antiprotozoal activity, natural product isolation, structure elucidation

## Abstract

Malaria and human African trypanosomiasis (HAT; sleeping sickness) are life-threatening tropical diseases caused by protozoan parasites. Due to limited therapeutic options, there is a compelling need for new antiprotozoal agents. In a previous study, O-tigloylcyclovirobuxeine-B was recovered from a *B*. *sempervirens* L. (common box; Buxaceae) leaf extract by bioactivity-guided isolation. This *nor*-cycloartane alkaloid was identified as possessing strong and selective in vitro activity against the causative agent of malaria tropica, *Plasmodium falciparum* (*Pf*). The purpose of this study is the isolation of additional alkaloids from *B*. *sempervirens* L. to search for further related compounds with strong antiprotozoal activity. In conclusion, 25 alkaloids were obtained from *B*. *sempervirens* L., including eight new natural products and one compound first described for this plant. The structure elucidation was accomplished by UHPLC/+ESI-QqTOF-MS/MS and NMR spectroscopy. The isolated alkaloids were tested against *Pf* and *Trypanosoma brucei rhodesiense* (*Tbr*), the causative agent of East African sleeping sickness. To assess their selectivity, cytotoxicity against mammalian cells (L6 cell line) was tested as well. Several of the compounds displayed promising in vitro activity against the pathogens in a sub-micromolar range with concurrent high selectivity indices (SI). Consequently, various alkaloids from *B*. *sempervirens* L. have the potential to serve as a novel antiprotozoal lead structure.

## 1. Introduction

*Buxus sempervirens* L. (common box; Buxaceae) is an evergreen shrub or small tree, with *nor*-triterpene alkaloids of the *nor*-cycloartane type as its main chemical constituents. Decoctions of leaves were ethnopharmacologically used against a variety of indications, including malaria [1,2].

Malaria is a life-threatening infectious disease caused by protozoans of the genus *Plasmodium*. The vector of these parasites is the infected female *Anopheles* mosquito. The World Health Organization (WHO) estimated that 229 million malaria cases and 409,000 malaria deaths occurred worldwide in 2019. The incidence has remained virtually unchanged in recent years and, because of the coronavirus pandemic, the WHO estimated 100,000 additional deaths in 2020. The progress in the fight against malaria has stalled. There is a high incidence of treatment failure due to resistance and, thus, the development of new antimalarial drugs is indispensable.

Phytochemical studies on *B*. *sempervirens* L. led to the isolation of almost 200 alkaloids [3], which have displayed interesting bioactivities including antibacterial, antimycobacterial, antimalarial, and acetyl- and butyryl-cholinesterase inhibition activity [4,5].

In a previous study by our group [6], O-tigloylcyclovirobuxeine-B, after bioactivity-guided isolation from the dichloromethane extract of *B. sempervirens* leaves, was identified as the constituent mainly responsible for the plant’s antiplasmodial activity. It appeared likely that further congeneric alkaloids also yield contributions to the overall antiplasmodial effect of the total leaf extract. Moreover, a lupane triterpene of *B*. *sempervirens* L. showed prominent bioactivity even against drug-resistant malaria parasites [7]. Consequently, the isolation, as well as the antiplasmodial testing of further *Buxus*-alkaloids, is of great interest.

In a recent study [8], it was determined that the alkaloid-enriched fraction of *Pachysandra terminalis* (Buxaceae) possessed promising activity against another protozoan parasite, *Trypanosoma brucei rhodesiense* (*Tbr*), the causative agent of East African sleeping sickness, a poverty-related neglected tropical disease. This plant contains aminosteroids structurally related to the *nor*-triterpene alkaloids of *B*. *sempervirens* L. Furthermore, an isolated compound of *B*. *sempervirens* L., cyclovirobuxeine-B, was found to be highly active and selective against *Tbr* in vitro [9]. These findings suggested that additional *Buxus*-alkaloids could likewise represent strong trypanocides. The chemotherapeutic agents currently in use against African sleeping sickness (human African trypanosomiasis (HAT)) are toxic and have many other associated disadvantages, such as long hospitalization. Accordingly, there is a compelling need for new treatments.

The aim of this study was the identification and characterization of potent and selective antiprotozoal compounds as lead structures against tropical diseases. For this purpose, we report on the systematic isolation of 25 alkaloids from a *B*. *sempervirens* L. leaf extract, including eight new natural products and one compound first described for this plant. The compounds were obtained by centrifugal partition chromatography (CPC), preparative high performance liquid chromatography (prep-HPLC), and column chromatography (CC). Additionally, we present the results of the in vitro testing of the isolated alkaloids against the pathogens of malaria and HAT.

## 2. Results and Discussion

### 2.1. Identification of Isolated Buxus-Alkaloids

**Figure 1:**
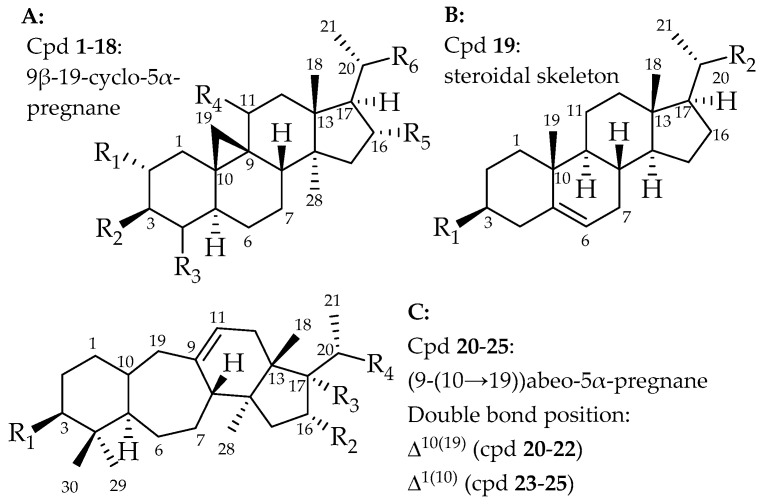
Core skeletons of isolated *Buxus*-alkaloids **1**–**25**. For complete structures, see Table 1.

**Scheme 1:**
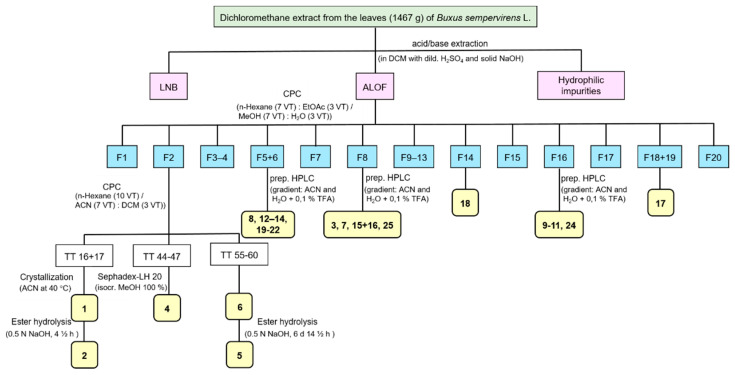
From the leaves of *B*. *sempervire*ns L. Abbreviations: lipophilic and neutral fraction (LNB); alkaloid fraction (ALOF); centrifugal partition chromatography (CPC); fraction (F); and test tube (TT).

The isolation scheme of alkaloids from *B*. *sempervirens* L. (Scheme 1) consisted of various separation methods such as CPC, prep-HPLC, and CC. It resulted in the isolation of 25 compounds. The structure elucidation and identification of the isolated *Buxus*-alkaloids were accomplished by UHPLC/+ESI-QqTOF-MS/MS (henceforth termed “LC/MS”) and NMR spectroscopy. Eighteen alkaloids (**1**–**18**), with a 9β-19-cyclo-5α-pregnane core skeleton, were isolated in addition to one steroidal alkaloid (**19**) and six substances, which possess a (9(10→19))abeo-5α-pregnane core skeleton (**20**–**25**) (Figure 1). The spectral data of the previously known compounds (Table 1), O-tigloylcyclovirobuxeine-B (**1**) [6,10,11], cyclovirobuxeine-B (**2**) [9,12], cyclomicrophylline-A (**5**) [13,14], cyclomicrophyllidine-A (**6**) [15,16], N-benzoyl-O-acetyl-cycloxo-buxoline-F (**9**) [10], N-benzoyl-cycloxo-buxoline-F (**10**) [10], N_b_-dimethylcycloxobuxoviricine (**12**) [17], (*E*)-cyclobuxophyllinine-M (**13**) and (*Z*)-cyclobuxophyllinine-M (**14**) [18,19], (*E*)-cyclosuffrobuxinine-M (**15**) and (*Z*)-cyclosuffrobuxinine-M (**16**) [20,21,22], cyclomicrobuxinine (**17**) [21,23], cyclomicrobuxine (**18**) [23], irehine (**19**) [24], 16-α-hydroxybuxaminone (**20**) [25], N_20_-acetylbuxamine-E (**21**) [26], and N-benzoyl-O-acetylbuxodienine-E (**22**) [27], were in full agreement with findings reported in the literature. Additionally, 2D-NMR spectra (COSY, HSQC, and HMBC) were evaluated to confirm the identification of the known compounds. To the best of our knowledge, cyclomicrophyllidine-A (**6**), previously known only from *B*. *microphylla* Sieb. *et* Zucc., has been isolated from the leaves of *B*. *sempervirens* L. for the first time.

The eight compounds **3**, **4**, **7**, **8**, **11**, and **23**–**25** are described for the first time, to the best of our knowledge.

**Figure 2:**
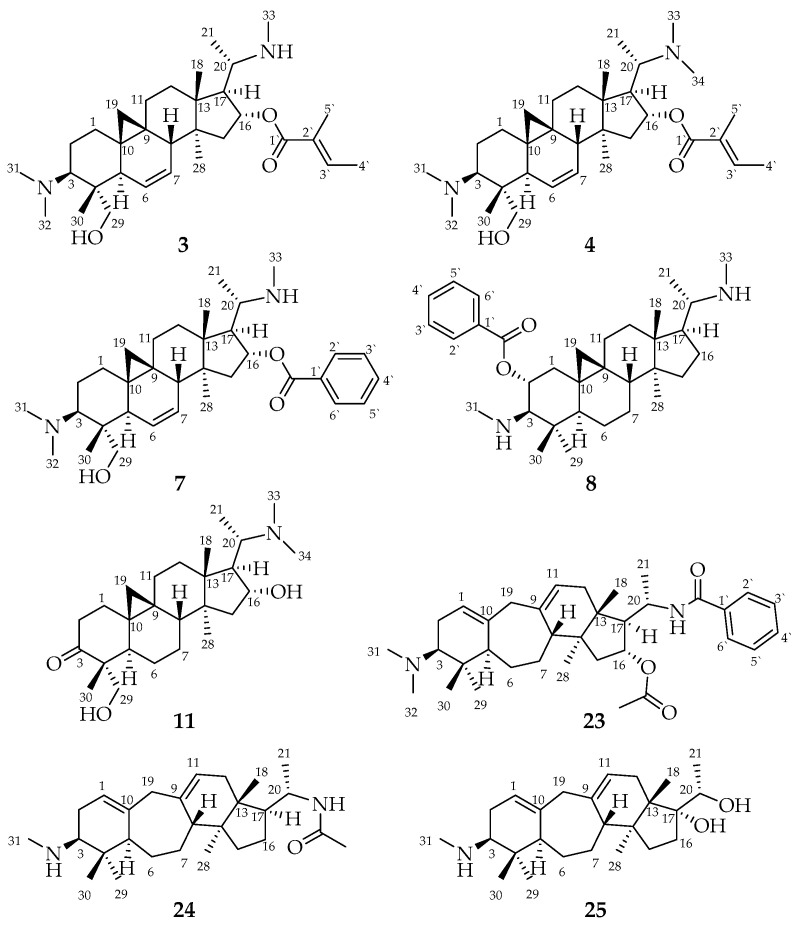
Chemical structure of the new natural products **3**, **4**, **7**, **8**, **11** and **23**–**25**.

The molecular formula of compound **3**, obtained as a colorless gum, was determined as C_32_H_52_N_2_O_3_ by LC/MS (Figures S5–S7, Supplementary Materials). The ^1^H- and ^13^C-NMR spectroscopic data (Table 2; Figures S8–S17, Supplementary Materials) displayed partly similar signals to **1** and differed from 9β-19-cyclo-5α-pregnane by the presence of signals for a methylene instead of a methyl group. The chemical shift of the methylene (δ_C_ = 67.80; δ_H_ = 4.05 and 3.79 (1H, d, 11.6 Hz)) suggested a hydroxyl-containing structure. HMBC correlations of the methylene carbon with H-3, H-5, H-8 and H-30 confirmed compound **3** as the C-29 hydroxylated analogue of **1** (Figure 2).

Compound **4**, obtained from CPC fraction 2 as a white powder, possesses the molecular formula C_33_H_54_N_2_O_3_ according to LC/MS analysis (Figures S18–S20, Supplementary Materials), which indicates a further methylated analogue of **3**. The ^1^H- and ^13^C-NMR spectroscopic data (Table 2; Figures S21–S30, Supplementary Materials) displayed great similarity between **4** and **3**. In contrast to a monomethylated amine in **3**, **4** displayed signals of two magnetically equivalent N-methyl groups at δ_C_ = 40.71 and δ_H_ = 2.14 (6H, s), which showed correlation with C-20 in the HMBC experiment. In contrast to compound **3**, the structure of **4** possesses a tertiary amine (dimethylamino group) at position C-20 instead of a secondary amine (monomethylamino group) (Figure 2).

For compound **7**, the molecular formula was determined as C_34_H_50_N_2_O_3_ by LC/MS (Figures S39–S41, Supplementary Materials). Most signals in the NMR spectra (Table 2; Figures S42–S50, Supplementary Materials) were in common with compound **3**. The only difference was observed in the signals of the ester side chain at C-16 of **7**, which differed from **3** by the presence of a benzoate moiety instead of a tiglate moiety (Figure 2).

Compound **8**, isolated from CPC fractions 5 and 6, displayed the molecular formula C_33_H_50_N_2_O_2_ in LC/MS analysis (Figures S51–S53, Supplementary Materials). The signals of the NMR spectra (Table 2; Figures S54–S62, Supplementary Materials) for the major part were in agreement with the previous 9β-19-cyclo-5α-pregnane derivatives (**1**–**7**). In the ^1^H-NMR spectrum the two doublet signals (J = 4.6 Hz) of the methylene group at C-19 of the cyclopropane ring could be detected at δ_H_ = 0.80 and 0.71. This downfield shift in comparison to the corresponding signal in compounds **1**–**7**, which all display the signal for one of the protons at C-19 between δ_H_ = 0.4 and −0.2, indicates the absence of anisotropic shielding by the Δ^6^ double bond. This observation was in full agreement with the absence of an olefinic proton signal for position 6 and so, in conclusion, the B-ring in **8** is fully saturated [17,28]. In addition, the typical proton signals of a benzoic acid ester at δ_H_ = 8.06 (m (2H), Pos. 2′/6′), 7.66 (m (1H), Pos. 4′), and 7.53 (m (2H), Pos 3′/5′) could be identified. The carbonyl carbon of the ester resonated at δ_C_ = 166.74 and showed ^3^J couplings in the ^1^H/^13^C-HMBC spectrum with the protons in position 2 of the A-ring and positions 2′/6′ of the benzoic acid. The proton in position 2, accordingly, displayed the usual chemical shift caused by geminal relationship with an ester group, i.e., δ_H_ = 5.30 (td (1H), J = 10.9 and 4.8 Hz) [29]. The signals at δ_H_ = 2.96 (s (3H)) and 2.67 (s (3H)) indicated a *Buxus*-alkaloid with two monomethylated amino groups at C-3 and C-20. The resulting structure is presented in Figure 2.

For compound **11**, the molecular formula was determined as C_26_H_43_NO_3_ by LC/MS (Figures S71–S73, Supplementary Materials). In the ^1^H, ^13^C, and HSQC-NMR spectra for **11** (Table 2; Figures S74–S81, Supplementary Materials) the presence of six sp^3^ quaternary carbons, five methines, nine methylenes, and six methyl groups were detected. The present compound showed similarity in the NMR spectra with the signals of cyclomicrophylline A (**5**). For instance, the ^1^H/^13^C-HSQC spectrum displayed the typical interactions of the proton in the hydroxylated position 16 at δ_H_ = 4.31 with δc = 77.20 and the signals of the hydroxylated methylene group in position 29 at δ_H_ = 3.86 and 3.32 (d, 11.2 Hz) with δc = 64.35. Alternatively, the resonances of the olefinic methine protons are missing, which suggested a saturated B-ring. In the ^13^C-NMR spectrum, a signal at δc = 217.33 could be detected in the low field in the typical shift range of a ketone [29]. The ^2^J and ^3^J couplings in the ^1^H/^13^C-HMBC spectrum of the ketone carbon with positions 1, 2, 29 and 30 clearly assigned it to C-3. This position also appears plausible from a biosynthetic point of view [28] for the elucidated new structure (Figure 2).

In the +ESI-QqTOF MS/MS spectrum (Figure S121, Supplementary Materials), compound **23** exhibited identical fragmentation (the fragmentation pathway is reported for the first time in Figure S122, Supplementary Materials) with N-benzoyl-O-acetylbuxodienine-E (**22**). The molecular formula C_35_H_50_N_2_O_3_, derived from the quasimolecular ions (*m*/*z* 274.1989 [M + 2H]^2+^, 547.3917 [M + H]^+^) and the resulting 12 double bond equivalents of the two substances, agree. Thus, the present compound’s structure must be very similar to that of the already known congener and represents an isomer of **22**. The UV spectrum (Figure S123, Supplementary Materials) of structure **23** indicated the presence of a secondary benzamide with an absorption maximum at 225 nm [10]. The absorption of a diene system, as in compound **22** (λ_max_ at 237, 245 and 253 nm, Figure S114, Supplementary Materials), was not detectable. The signals of the NMR spectra (Table 3; Figures S124–S133, Supplementary Materials) of **22** and **23** were very similar for the most part. Deviations occurred in the chemical shifts at positions C-11 and C-19 in the A-ring. In the ^1^H/^13^C-HSQC spectrum of compound **23**, two signals could be detected at δ_H_ = 5.42 (br s)/δc = 121.95, as well as δ_H_ = 5.31 (br m)/δc = 119.37, which indicated olefinic structural elements. In the ^1^H/^13^C-HMBC spectrum, these signals did not show any interaction with each other, as would be expected in a conjugated Δ^9(11),10(19)^ system as found in **22**. Instead, the chemical shifts agree with a (9-(10→19))abeo-pregnane with the presence of two isolated double bonds, i.e., Δ^1(10)^ and Δ^9(11)^ [28,30]. Consequently, compound **23** could be identified as a constitutional isomer of **22** with a double bond between C-1 and C-10, instead of C-10 and C-19 (Figure 2).

For compound **24,** the molecular formula was determined as C_27_H_44_N_2_O by LC/MS. The +ESI-QqTOF mass spectrum (Figure S135, Supplementary Materials) showed protonated ion signals at *m*/*z* 207.4721 [M + 2H]^2+^ and *m*/*z* 413.0273 [M + H]^+^, in which the intensity of the [M + H]^+^ clearly outweighed the signal of the [M + 2H]^2+^ (as in the +ESI-QqTOF mass spectrum of compound **9**, **10** and **21**–**23**). This implied a structure with two nitrogen groups, which have clearly different basicities. The fragmentation in the +ESI-QqTOF MS/MS spectrum (Figure S136, Supplementary Materials) with a neutral loss of 31 Da suggested the presence of a monomethylamino group at C-3 or C-20 (*m*/*z* = 382 [M-CH_3_NH_2_]^+^). The fragment at *m*/*z* = 323 [382-CH_3_CONH_2_]^+^ indicated the neutral loss of an acetamide group (-59 Da). By means of the NMR spectroscopic data (Table 3; Figures S137–S145, Supplementary Materials), compound **24**, in analogy with **23,** could clearly be assigned to the *Buxus*-alkaloids with a (9-(10→19))abeo-pregnane backbone and isolated double bonds between C-1 and 10 and between C-9 and 11. The acetamide group, already suspected by the fragmentation, could be located at the nitrogen atom at C-20 by the cross signal of the carbonyl carbon (δ_C_ = 170.18) with the proton at position 20 (δ_H_ = 3.99) in the ^1^H/^13^C-HMBC spectrum. This amide group has a significantly reduced basicity in contrast to the secondary amine group in position 3, which provides an explanation for the low intensity of the [M + 2H]^2+^ quasimolecular ion in the +ESI-QqTOF mass spectrum. The resulting structure is presented in Figure 2.

The LC/MS analysis (Figures S146–S148, Supplementary Materials) of compound **25** indicated the molecular formula C_25_H_41_NO_2_. The fragmentation (Figure S148, Supplementary Materials) already gave rise to some structural features that are contained in compound **25**. The fragment at *m*/*z* 339 [M-(CH_3_NH_2_)-(H_2_O)]^+^ resulted from the loss of a monomethylated amino group, together with the elimination of a hydroxyl group as a water molecule. The following fragment at *m*/*z* 321 [339-H_2_O]^+^ showed an additional hydroxyl group. By evaluating the NMR spectra (Table 3; Figures S149–S158, Supplementary Materials), compound **25** was clearly identified as a *Buxus*-alkaloid with a (9-(10→9))abeo-pregnane skeleton and isolated double bonds between C-1 and C-10 and between C-9 and C-11. A methine proton signal at δ_H_ = 3.84 (δ_C_ = 73.7 according to the ^1^H/^13^C-HSQC spectrum) resonated as a quartet (J = 6.1 Hz) and should therefore be in geminal position with a methyl group and must represent position 20. In the ^1^H/^13^C-HMBC spectrum, this proton showed cross peaks with carbons at δ_C_ = 18.21 (CH_3_), 38.63 (CH_2_), and 85.50 (quaternary C). The signal at δ_C_ = 18.21 was assigned the methyl group in position 21 (δ_H_ = 1.18 (d, J = 6.1)), whereas δ_C_ = 38.63 could be assigned to the methylene group in position 16. The quaternary carbon at δ_C_ = 85.50 resonated in the typical shift range of a tertiary alcohol group and could be assigned to position 17. The present alkaloid thus possesses a vicinal diol structure at positions 17 and 20 (Figure 2).

The generic names of the new natural products were chosen based on the existing classification for *Buxus*-alkaloids [31,32]: O-tigloylcyclomicrophylline-B (**3**), O-tigloylcyclomicrophylline-A (**4**), cyclomicrophyllidine-B (**7**), O-benzoyl-cycloprotobuxoline-D (**8**), 29-hydroxy-cyclomikuranine-L (**11**), N-benzoyl-O-acetylbuxadine-E (**23**), N_20_-acetylbuxadine-G (**24**), and 17,20-dihydroxybuxadine-M (**25**). Compound **24** was concurrently isolated by Xiang et al. [33]. We prefer the systematic generic name N_20_-acetylbuxadine-G for this substance. The chemical structures of the new natural products are reported in Figure 2.

### 2.2. In Vitro Antiprotozoal Activity of Isolated Compounds

After isolation, all alkaloids were tested in vitro for antiplasmodial and antitrypanosomal activity against *Plasmodium falciparum (Pf)* and *Trypanosoma brucei rhodesiense (Tbr).* Furthermore, to assess their selectivity against the parasites, cytotoxicity against L6 rat skeletal myoblasts, as mammalian control cells, was tested (Table 4).

Compounds **3**, **4** and **6–8** showed conspicuous activity against *Pf* with IC_50_ values <1.0 µM. The new natural products, O-benzoyl-cycloprotobuxoline-D (**8**) and cyclomicrophyllidine-B (**7**), were the most active antiplasmodial compounds with IC_50_ values of 0.18 and 0.2 µM and SI values of 74 and 145, respectively. Except for compounds **17** (IC_50_ 10.9 µM) and **18** (IC_50_ 6.8 µM), all other 9β-19-cyclo-5α-pregnanes (**1**, **2**, **5**, **9**, **11**–**16**) and the steroidal alkaloid (**19**) displayed moderate activities with IC_50_ values in the range of 1.05 to 4.3 µM. The (9-(10→19))abeo-5α-pregnanes (**20**–**25**) were less active with IC_50_ values of >4.0 µM.

Promising antitrypanosomal activities with IC_50_ values between 1.1 and 1.5 µM were recorded for compounds **2**, **6**, **8** and **24**. In common with the tests against *Pf*, O-benzoyl-cycloprotobuxoline-D (**8**) (IC_50_ 1.1 µM; SI 12) was the most effective antitrypanosomal *Buxus*-alkaloid in this set of compounds. Cyclomicrophylline-A (**5**) showed the highest selectivity against *Tbr* (SI 42). Compounds **1**, **4**, **5**, **7**, **9** and **13**–**16** were moderately active, with IC_50_ values varying between 2.1 and 3.6 µM. The IC_50_ values of the other alkaloids against *Tbr* were >5.7 µM, indicating a low level of activity.

## 3. Materials and Methods

### 3.1. Plant Material

The same plant material was used as described previously [11].

### 3.2. Extraction and Isolation of Alkaloids from the B. sempervirens Leaf Extract

The extraction of plant material, the first part of the isolation procedure, and the isolation of O-tigloylcyclovirobuxeine-B (**1**) was equal to our previous study (Scheme 1) [11].

After the second CPC separation of Fraction 2 (F2) [11], test tubes 44–47 (7 mg) contained compound **4** as a main constituent. A Sephadex-LH 20 column (15 × 40 cm, flow 0.4 mL/min) was applied for purification of **4** using MeOH (isocratic, 100 mL) as eluent. Compound **4** (2.2 mg) was obtained as a white powder. The CPC test tubes 55–60 consisted of pure compound **6** (11.8 mg). CPC fractions 5 + 6 (103.2 mg), 8 (138.0 mg), and 16 (108.4 mg) were separated on an RP18 phase (Macherey-Nagel, Nucleodur C-18 HTec, 250 × 21 mm, 5 μm) using a H_2_O (+0.1% TFA; A): ACN (+0.1% TFA; B) gradient (0 min: 5% B; 5 min: 20% B; 12.5 min: 30% B; 14 min: 32% B; 22 min: 35% B; 35 min: 100% B; and 40 min: 100% B) by prep-HPLC. Fractions 5 and 6 resulted in the isolation of compound **8** (1.6 mg, t_R_ 20.0 min), **12** (2.8 mg, t_R_ 27.6 min), **13** + **14** (5.4 mg, t_R_ 29.2 min), **19** (6.5 mg, t_R_ 20.8 min), **20** (5.6 mg, t_R_ 19.2 min), **21** (3.7 mg, t_R_ 27.2 min), and **22** (4.3 mg, t_R_ 29.6 min). The separation of fraction 8 yielded substance **3** (6.7 mg, t_R_ 16.4 min), **7** (4.0 mg, t_R_ 17.2 min), **15** + **16** (19.6 mg, t_R_ 28.0 min), and **25** (14.5 mg, t_R_ 24.4 min). Compound **9** (1.9 mg, t_R_ 26.2 min), **10** (2.0 mg, t_R_ 27.0 min), **11** (3.8 mg, t_R_ 19.0 min), and **24** (7.3 mg, t_R_ 28.2 min) were obtained by prep-HPLC of fraction 16.

CPC fractions F14 and F18+F19 presented **18** (28.6 mg) and **17** (391.5 mg) as pure compounds, respectively. Compound **23** (10.0 mg) was obtained from the crude dichloromethane extract by CC on silica gel (Merck, type-60, 70–230 mesh). The separation was introduced by gradient elution of ethyl acetate (EtOAc) (100%) until eluates became clear (eluates contained no alkaloid), followed by EtOAc saturated with aq. ammonia (3.5 L, flow 1 mL/min). Six fractions (five alkaloidal) were collected in 20 mL tubes (Bs1, Bs2a, Bs3a, Bs4a, Bs5a (starting from tube 78) and Bs6a). Bs2a was further purified using isocratic aq. ammonia-saturated EtOAc in a smaller silica gel column (flow 0.4 mL/min) and yielded 10.0 mg of compound **23** (white powder).

### 3.3. Alkaline Hydrolysis of the Esters **1** and **6**

The corresponding free alcohols **2** and **5** were obtained from the already isolated compounds **1** and **6** by non-aqueous alkaline ester hydrolysis [34,35]. The esters **1** (32.0 mg) and **6** (4.7 mg) were hydrolyzed with 0.5 N NaOH in a dichloromethane-methanol mixture (9: 1 *v*/*v*) at room temperature. The completion of the reaction (**1** to **2**: 4 ½ h and **6** to **5**: 6 d, 14 ½ h) was monitored by thin layer chromatography (TLC plate silica gel 60 F_254_, Merck KGaA, Darmstadt, Germany; mobile phase: butan-1-ol:H_2_O:CH_3_COOH (10:3:1) (*v*/*v*/*v*), detected with Liebermann-Burchard reagent (acetic anhydride (5 mL), sulfuric acid (5 mL), and ethanol (50 mL), European Pharmacopoeia Reagent)), and the resulting alcohols were purified by extraction (four times with 100 mL dichloromethane). The yield in each case was >85%: **2** (22.8 mg) and **5** (3.2 mg). Since these two compounds are only found in small amounts in the crude extract of *B*. *sempervirens* L., ester hydrolysis was used to increase the yield.

### 3.4. Spectroscopic Analysis of Isolated Compounds

NMR spectra were recorded on an Agilent DD2 600 MHz spectrometer (Agilent, Santa Clara, CA, USA) at 25 °C in CDCl_3_ or CD_3_OD. Spectra were referenced to the solvent signals (CDCl_3_: ^1^H: 7.260 ppm; ^13^C: 77.160 ppm; CD_3_OD: ^1^H: 3.310 ppm; and ^13^C: 49.000 ppm) and were evaluated with MestReNova version 11.0 software (Mestrelab Research, Santiago de Compostela, Spain).

UHPLC/+ESI-QqTOF-MS/MS measurements were performed as described previously [11]. The sample concentration of the crude extract was 10 mg/mL in case of the CPC fractions 1 mg/mL, and for the isolated compounds it was 0.1 mg/mL.

UV spectra of compounds **22** and **23** were recorded with a U-2900 spectrophotometer (Hitachi, Tokyo, Japan) in methanol.

### 3.5. Spectral Data of Isolated Buxus-Alkaloids

O-tigloylcyclovirobuxeine-B (**1**): previously described in [6,11].

Cyclovirobuxeine-B (**2**): white powder; ^1^H NMR (600 MHz, CDCl_3_; δ (ppm), intensity, mult., J (Hz)): 5.62 (1H, ddd, 10.6, 1.4, 1.4, H-6), 5.40 (1H, ddd, 10.6, 6.0, 3.1, H-7), 4.17 (1H, ddd, 9.2, 6.7, 2.1, H-16), 2.57 (1H, m, H-20), 2.53 (1H, dd, 5.8, 2.2, H-8), 2.47 (3H, s, H-33/34), 2.29 (6H, s, H-31/32), 2.06 (1H, m, H-3), 1.98 (1H, dd, 13.2, 9.7, H-15α), 1.86 (1H, m, H-5), 1.82 (1H, dd, 14.6, 5.7, H-11α), 1.75 (1H, m, H-2α), 1.69 (1H, td, 13.3, 5.0, H-12α), 1.63 (1H, dd, 10.5, 6.7, H-17), 1.54 (1H, m, H-2β), 1.53 (2H, m, H-1), 1.41 (1H, ddd, 15.0, 5.1, 1.9, H-11β), 1.36 (1H, m, H-12β), 1.28 (1H, m, H-15β), 1.12 (3H, d, 6.2, H-21), 1.06 (3H, s, H-29), 0.94 (3H, s, H-28), 0.91 (3H, s, H-18), 0.79 (3H, s, H-30), 0.72 (1H, d, 4.0, H-19α), -0.21 (1H, d, 4.0, H-19β);

^13^C NMR (150 MHz, CDCl_3_; δ (ppm)): 128.37 (CH, C-7), 127.72 (CH, C-6), 78.70 (CH, C-16), 71.43 (CH, C-3), 61.15 (CH, C-17), 59.17 (CH, C-20), 49.86 (qC, C-14), 48.78 (CH, C-5), 45.62 (qC, C-13), 44.31 (CH_3_, C-31/32), 43.37 (CH, C-8), 41.94 (CH_2_, C-15), 41.60 (qC, C-4), 33.52 (CH_3_, C-33/34), 32.10 (CH_2_, C-12), 31.15 (CH_2_, C-1), 28.86 (qC, C-10), 26.17 (CH_3_, C-29), 24.96 (CH_2_, C-11), 20.92 (qC, C-9), 20.03 (CH_2_, C-2), 18.77 (CH_2_, C-19), 18.63 (CH_3_, C-21), 18.39 (CH_3_, C-28), 16.66 (CH_3_, C-30), 15.70 (CH_3_, C-18).

+ESI-QqTOF-MS (*m*/*z*): 415.3696 [M + H]^+^, 208.1902 [M + 2H]^2+^ (calcd for C_27_H_47_N_2_O^+^: 415.3688, for C_27_H_48_N_2_O^2+^: 208.1884).

O-tigloylcyclomicrophylline-B (**3**): colorless gum; ^1^H NMR and ^13^C NMR (600/150 MHz, CDCl_3_) see Table 2;

+ESI-QqTOF-MS (*m*/*z*): 513.4221 [M + H]^+^, 257.2174 [M + 2H]^2+^ (calcd for C_32_H_53_N_2_O_3_^+^: 513.4056, for C_32_H_54_N_2_O_3_^2+^: 257.2067).

O-tigloylcyclomicrophylline-A (**4**): white powder; ^1^H NMR and ^13^C NMR (600/150 MHz, CD_3_OD) see Table 2;

+ESI-QqTOF-MS (*m*/*z*): 527.4220 [M + H]^+^, 264.2186 [M + 2H]^2+^ (calcd for C_33_H_55_N_2_O_3_^+^: 527.4213, for C_33_H_56_N_2_O_3_^2+^: 264.2146).

Cyclomicrophylline-A (**5**): white powder; ^1^H NMR (600 MHz, CD_3_OD; δ (ppm), intensity, mult., J (Hz)): 5.51 (1H, m, H-6), 5.48 (1H, m, H-7), 4.15 (1H, ddd, 9.4, 6.8, 2.2, H-16), 3.81 (1H, d, 10.3, H-29α), 3.52 (1H, d, 10.4, H-29β), 2.76 (1H, m, H-20), 2.67 (1H, dd, 12.0, 3.5, H-3), 2.59 (1H, dd, 5.5, 2.5, H-8), 2.34 (6H, s, H-31/32), 2.29 (6H, s, H-33/34), 2.03 (1H, m, H-15α), 1.94 (1H, d, 2.6, H-5), 1.89 (1H, m, H-11α), 1.88 (1H, m, H-17), 1.81 (1H, m, H-2α), 1.72 (1H, m, H-12α), 1.65 (1H, m, H-2β), 1.61 (2H, m, H-1), 1.48 (1H, ddd, 15.1, 5.1, 1.9, H-11β), 1.37 (1H, m, H-12β), 1.19 (1H, dd, 13.6, 2.2, H-15β), 1.05 (3H, s, H-30), 0.98 (3H, s, H-28), 0.96 (3H, s, H-18), 0.93 (3H, d, 6.6, H-21), 0.78 (1H, d, 4.1, H-19α), -0.10 (1H, d, 4.1, H-19β);

^13^C NMR (150 MHz, CD_3_OD; δ (ppm)): 130.60 (CH, C-7), 126.75 (CH, C-6), 80.17 (CH, C-16), 73.21 (CH, C-3), 73.08 (CH_2_, C-29), 63.88 (CH, C-20), 57.77 (CH, C-17), 50.93 (qC, C-14), 46.31 (qC, C-13), 45.92 (CH, C-5), 44.51 (CH, C-8), 44.50 (CH_3_, C-33/34), 43.36 (qC, C-4), 42.97 (CH_3_, C-31/32), 42.39 (CH_2_, C-15), 32.95 (CH_2_, C-12), 31.79 (CH_2_, C-1), 29.16 (qC, C-10), 25.74 (CH_2_, C-11), 21.91 (qC, C-9), 19.54 (CH_2_, C-2), 19.24 (CH_2_, C-19), 19.20 (CH_3_, C-28), 15.70 (CH_3_, C-18), 12.81 (CH_3_, C-30), 10.53 (CH_3_, C-21).

+ESI-QqTOF-MS (*m*/*z*): 445.3835 [M + H]^+^, 223.1989 [M + 2H]^2+^ (calcd for C_28_H_49_N_2_O_2_^+^: 445.3794, for C_28_H_50_N_2_O_2_^2+^: 223.1936).

Cyclomicrophyllidine-A (**6**): colorless gum; ^1^H NMR (600 MHz, CD_3_OD; δ (ppm), intensity, mult., J (Hz)): 8.00 (2H, m, H-2′/6′), 7.59 (1H, m, H-4′), 7.47 (2H, m, H-3′/5′), 5.49 (1H, m, H-6), 5.46 (1H, m, H-7), 5.34 (1H, ddd, 8.6, 5.9, 1.1, H-16), 3.80 (1H, m, H-29α), 3.51 (1H, m, H-29β), 2.66 (1H, m, H-20), 2.66 (1H, m, H-3), 2.59 (1H, dd, 5.4, 2.5, H-8), 2.35 (1H, m, H-17), 2.34 (6H, s, H-31/32), 2.16 (1H, m, H-15α), 2.09 (6H, s, H-33/34), 1.94 (1H, br s, H-5), 1.86 (1H, m, H-11α), 1.82 (1H, m, H-2α), 1.72 (1H, dd, 13.3, 5.1, H-12α), 1.64 (1H, m, H-2β), 1.61 (2H, m, H-1), 1.47 (1H, m, H-11β), 1.37 (1H, ddd, 13.1, 5.6, 1.9, H-12β), 1.24 (1H, d(d), 14.2, (<1), H-15β), 1.05 (3H, s, H-30), 1.04 (3H, s, H-28), 0.96 (3H, s, H-18), 0.90 (3H, d, 6.4, H-21), 0.78 (1H, d, 4.1, H-19α), -0.10 (1H, d, 4.1, H-19β);

^13^C NMR (150 MHz, CD_3_OD; δ (ppm)): 167.64 (qC, O**C**O), 134.21 (CH, C-4′), 132.17 (qC, C-1′), 130.60 (CH, C-7), 130.32 (CH, C-2′/6′), 129.58 (CH, C-3′/5′), 126.75 (CH, C-6), 81.87 (CH, C-16), 73.19 (CH, C-3), 73.11 (CH_2_, C-29), 61.36 (CH, C-20), 56.95 (CH, C-17), 50.93 (qC, C-14), 46.28 (qC, C-13), 45.93 (CH, C-5), 44.51 (CH, C-8), 43.36 (qC, C-4), 43.27 (CH_2_, C-15), 42.98 (CH_3_, C-31/32), 40.77 (CH_3_, C-33/34), 32.95 (CH_2_, C-12), 31.80 (CH_2_, C-1), 29.17 (qC, C-10), 25.74 (CH_2_, C-11), 21.91 (qC, C-9), 19.53 (CH_2_, C-2), 19.25 (CH_2_, C-19), 18.30 (CH_3_, C-28), 15.70 (CH_3_, C-18), 12.81 (CH_3_, C-30), 10.49 (CH_3_, C-21).

+ESI-QqTOF-MS (*m*/*z*): 549.4043 [M + H]^+^, 275.2089 [M + 2H]^2+^ (calcd for C_35_H_53_N_2_O_3_^+^: 549.4056, for C_35_H_54_N_2_O_3_^2+^: 275.2067).

Cyclomicrophyllidine-B (**7**): colorless gum; ^1^H NMR and ^13^C NMR (600/150 MHz, CD_3_OD) see Table 2;

+ESI-QqTOF-MS (*m*/*z*): 535.3964 [M + H]^+^, 268.2029 [M + 2H]^2+^ (calcd for C_34_H_51_N_2_O_3_^+^: 535.3900, for C_34_H_52_N_2_O_3_^2+^: 268.1989).

O-benzoyl-cycloprotobuxoline-D (**8**): colorless gum; ^1^H NMR and ^13^C NMR (600/150 MHz, CD_3_OD) see Table 2;

+ESI-QqTOF-MS (*m*/*z*): 507.4024 [M + H]^+^, 254.2062 [M + 2H]^2+^ (calcd for C_33_H_51_N_2_O_2_^+^: 507.7830, for C_33_H_52_N_2_O_2_^2+^: 254.3955).

N-benzoyl-O-acetyl-cycloxo-buxoline-F (**9**): colorless gum; ^1^H NMR (600 MHz, CD_3_OD; δ (ppm), intensity, mult., J (Hz)): 7.75 (2H, m, H-2′/6′), 7.53 (1H, m, H-4′), 7.46 (2H, m, H-3′/5′), 4.36 (1H, dd, 12.7, 4.3, H-3), 3.90 (1H, m, H-29α), 3.77 (1H, d, 11.6, H-29β), 3.42 (1H, m, H-20), 2.91 (3H, s, H-33/34), 2.75 (3H, s, H-33/34), 2.66 (1H, s, H-12α), 2.47 (1H, m, H-1α), 2.40 (1H, m, H-12β), 2.38 (1H, m, H-17), 2.20 (1H, m, H-8), 2.10 (1H, m, H-5), 2.09 (3H, s, Ac-C**H_3_**), 2.06 (1H, m, H-16α), 1.86 (1H, dd, 13.1, 4.0, H-2α), 1.73 (1H, m, H-15α), 1.72 (1H, m, H-2β), 1.67 (1H, m, H-15β), 1.66 (1H, m, H-16β), 1.62 (1H, m, H-7α), 1.60 (1H, d, 3.9, H-19α), 1.56 (1H, dd, 13.3, 3.9, H-6α), 1.42 (1H, m, H-7β), 1.37 (1H, m, H-1β), 1.29 (3H, d, 6.6, H-21), 1.26 (1H, d, 3.9, H-19β), 1.15 (1H, m, H-6β), 1.14 (3H, s, H-28), 0.96 (3H, s, H-18), 0.90 (3H, s, H-30);

^13^C NMR (150 MHz, CD_3_OD; δ (ppm)): 212.85 (qC, C-11), 173.10 (qC, Ac-**C**O), 170.67 (qC, O**C**NH), 136.35 (qC, C-1′), 132.47 (CH, C-4′), 129.45 (CH, C-3′/5′), 128.42 (CH, C-2′/6′), 67.16 (CH, C-20), 66.98 (CH_2_, C-29), 52.74 (CH_2_, C-12), 52.42 (CH, C-3), 49.79 (qC, C-14), 48.09 (CH, C-17), 47.01 (qC, C-13), 44.37 (qC, C-4), 43.57 (CH_3_, C-33/34), 42.90 (CH, C-5), 42.79 (CH, C-8), 39.29 (qC, C-10), 35.96 (CH_3_, C-33/34), 35.19 (qC, C-9), 35.05 (CH_2_, C-15), 32.13 (CH_2_, C-19), 29.13 (CH_2_, C-1), 28.33 (CH_2_, C-2), 26.23 (CH_2_, C-16), 25.72 (CH_2_, C-7), 20.94 (CH_3_, Ac-**C**H_3_), 19.83 (CH_2_, C-6), 19.34 (CH_3_, C-28), 17.24 (CH_3_, C-18), 11.98 (CH_3_, C-30), 11.65 (CH_3_, C-21).

+ESI-QqTOF-MS (*m*/*z*): 563.3939 [M + H]^+^, 282.2024 [M + 2H]^2+^ (calcd for C_35_H_51_N_2_O_4_^+^: 563.3849, for C_35_H_52_N_2_O_4_^2+^: 282.1964); MS/MS (*m*/*z*): 518 [M-(CH_3_)_2_NH]^+^, 476 [518-CH_2_=CO]^+^, 458 [476-H_2_O]^+^, 122 [C_6_H_5_CONH_3_]^+^, 105 [C_6_H_5_CO]^+^.

N-benzoyl-cycloxo-buxoline-F (**10**): colorless gum; ^1^H NMR (600 MHz, CD_3_OD; δ (ppm), intensity, mult., J (Hz)): 7.83 (2H, m, H-2′/6′), 7.55 (1H, m, H-4′), 7.47 (2H, m, H-3′/5′), 4.15 (1H, dd, 12.6, 4.0, H-3), 3.41 (1H, m, H-20), 3.39 (1H, m, H-29α), 3.13 (1H, d, 12.7, H-29β), 2.91 (3H, s, H-33/34), 2.75 (3H, s, H-33/34), 2.65 (1H, m, H-12α), 2.43 (1H, m, H-1α), 2.40 (1H, m, H-12β), 2.38 (1H, m, H-17), 2.20 (1H, m, H-8), 2.09 (1H, m, H-5), 2.06 (1H, m, H-16α), 1.94 (1H, dd, 13.1, 4.0, H-2α), 1.75 (1H, m, H-2β), 1.73 (1H, m, H-15α), 1.68 (1H, m, H-6α), 1.66 (1H, m, H-15β), 1.64 (1H, m, H-16β), 1.62 (1H, m, H-7α), 1.61 (1H, d, 3.8, H-19α), 1.48 (1H, m, H-7β), 1.34 (1H, m, H-1β), 1.29 (3H, d, 5.4, H-21), 1.26 (1H, d, 3.9, H-19β), 1.14 (3H, s, H-28), 1.07 (1H, m, H-6β), 0.95 (3H, s, H-18), 0.72 (3H, s, H-30);

^13^C NMR (150 MHz, CD_3_OD; δ (ppm)): 212.87 (qC, C-11), 171.59 (qC, O**C**NH), 135.42 (qC, C-1′), 132.83 (CH, C-4′), 129.57 (CH, C-3′/5′), 128.53 (CH, C-2′/6′), 67.19 (CH, C-20), 65.18 (CH_2_, C-29), 53.36 (CH, C-3), 52.78 (CH_2_, C-12), 49.90 (qC, C-14), 48.09 (CH, C-17), 47.08 (qC, C-13), 45.88 (qC, C-4), 43.56 (CH_3_, C-33/34), 42.79 (CH, C-5), 42.34 (CH, C-8), 39.52 (qC, C-10), 35.96 (CH_3_, C-33/34), 35.42 (qC, C-9), 34.96 (CH_2_, C-15), 31.65 (CH_2_, C-19), 29.27 (CH_2_, C-1), 27.79 (CH_2_, C-2), 26.23 (CH_2_, C-16), 25.46 (CH_2_, C-7), 19.49 (CH_2_, C-6), 19.34 (CH_3_, C-28), 17.22 (CH_3_, C-18), 11.90 (CH_3_, C-30), 11.65 (CH_3_, C-21).

+ESI-QqTOF-MS (*m*/*z*): 521.3766 [M + H]^+^, 261.1941 [M + 2H]^2+^ (calcd for C_33_H_49_N_2_O_3_^+^: 521.7660, for C_33_H_50_N_2_O_3_^2+^: 261.387); MS/MS (*m*/*z*): 476 [M-(CH_3_)_2_NH]^+^, 458 [476-H_2_O]^+^, 122 [C_6_H_5_CONH_3_]^+^, 105 [C_6_H_5_CO]^+^.

29-hydroxy-cyclomikuranine-L (**11**): colorless gum; ^1^H NMR and ^13^C NMR (600/150 MHz, CD_3_OD) see Table 3;

+ESI-QqTOF-MS (*m*/*z*): 418.3362 [M + H]^+^ (calcd for C_26_H_44_NO_3_^+^: 418.3321).

N_b_-dimethylcycloxobuxoviricine (**12**): white powder; ^1^H NMR (600 MHz, CD_3_OD; δ (ppm), intensity, mult., J (Hz)): 6.96 (1H, d, 10.1, H-1), 5.92 (1H, d, 10.1, H-2), 4.32 (1H, ddd, 9.4, 7.4, 2.1, H-16), 3.59 (1H, m, H-20), 2.96 (3H, s, H-33/34), 2.81 (3H, s, H-33/34), 2.23 (1H, dd, 11.1, 7.4, H-17), 2.15 (1H, dd, 11.1, 7.4, H-5), 2.10 (1H, m, H-11α), 2.05 (1H, m, H-15α), 2.00 (1H, dd, 10.1, 6.8, H-8), 1.88 (1H, m, H-12α), 1.67 (1H, m, H-11β), 1.62 (1H, m, H-6α), 1.62 (1H, m, H-12β), 1.56 (1H, m, H-7α), 1.44 (1H, dd, 13.8, 2.1, H-15β), 1.39 (1H, d, 4.7, H-19α), 1.32 (3H, d, 6.6, H-21), 1.30 (1H, m, H-7β), 1.21 (3H, s, H-28), 1.16 (1H, dd, 12.8, 4.0, H-6β), 1.09 (3H, s, H-29), 1.08 (3H, s, H-18), 0.97 (3H, s, H-30), 0.88 (1H, d, 4.7, H-19β);

^13^C NMR (150 MHz, CD_3_OD; δ (ppm)): 207.60 (qC, C-3), 156.58 (CH, C-1), 127.19 (CH, C-2), 76.92 (CH, C-16), 68.12 (CH, C-20), 56.08 (CH, C-17), 49.38 (qC, C-14), 47.40 (qC, C-13), 47.18 (qC, C-4), 46.97 (CH_2_, C-15), 46.06 (CH, C-5), 45.31 (CH, C-8), 43.69 (CH_3_, C-33/34), 36.55 (CH_3_, C-33/34), 32.33 (CH_2_, C-12), 31.41 (qC, C-10), 30.88 (CH_2_, C-19), 28.15 (CH_2_, C-11), 25.41 (qC, C-9), 24.88 (CH_2_, C-7), 21.86 (CH_3_, C-29), 20.61 (CH_2_, C-6), 20.50 (CH_3_, C-28), 19.62 (CH_3_, C-30), 18.48 (CH_3_, C-18), 11.48 (CH_3_, C-21).

+ESI-QqTOF-MS (*m*/*z*): 400.3291 [M + H]^+^ (calcd for C_26_H_42_NO_2_^+^: 400.3216).

(*E*)-cyclobuxophyllinine-M (**13**) and (*Z*)-cyclobuxophyllinine-M (**14**) (93.5%:6.5%): white powder; ^1^H NMR (600 MHz, CDCl_3_; δ (ppm), intensity, mult., J (Hz)): 6.58 (1H, q, 7.5, H-20 (*E*)), 5.74 (1H, q, 7.4, H-20 (*Z*)), 2.75 (3H, s, H-31/32), 2.64 (1H, m, H-3), 2.19 (1H, dd, 17.2, 1.5, H-15α), 2.11 (1H, m, H-11α), 2.08 (1H, m, H-12α), 2.06 (1H, m, H-12β), 2.04 (1H, m, H-2α), 2.01 (1H, m, H-15β), 1.87 (1H, m, H-2β), 1.84 (3H, d, 7.6, H-21), 1.68 (1H, m, H-6α), 1.66 (1H, m, H-8), 1.57 (1H, m, H-1α), 1.43 (1H, m, H-1β), 1.41 (1H, m, H-5), 1.38 (1H, m, H-7α), 1.33 (3H, s, H-18), 1.29 (1H, m, H-11β), 1.16 (3H, s, H-29), 1.11 (1H, qd, 12.6, 2.5, H-7β), 0.98 (3H, s, H-30), 0.94 (3H, s, H-28), 0.84 (1H, qd, 12.7, 2.4, H-6β), 0.67 (1H, d, 4.6, H-19α), 0.49 (1H, d, 4.6, H-19β);

^13^C NMR (150 MHz, CDCl_3_; δ (ppm)): 206.60 (qC, C-16), 146.59 (qC, C-17), 132.23 (CH, C-20 (*Z*)), 130.62 (CH, C-20 (*E*)), 69.81 (CH, C-3), 49.43 (CH_2_, C-15), 48.07 (CH, C-5), 46.76 (qC, C-13), 45.78 (CH, C-8), 42.48 (qC, C-14), 39.42 (qC, C-4), 32.87 (CH_3_, C-31/32), 31.84 (CH_2_, C-1), 30.27 (CH_2_, C-19), 29.36 (CH_2_, C-12), 26.20 (CH_2_, C-7), 26.12 (qC, C-10), 26.04 (CH_2_, C-11), 25.36 (CH_3_, C-29), 24.40 (CH_3_, C-18), 23.73 (CH_2_, C-2), 21.03 (CH_3_, C-28), 20.89 (CH_2_, C-6), 19.88 (qC, C-9), 15.02 (CH_3_, C-30), 13.39 (CH_3_, C-21).

+ESI-QqTOF-MS (*m*/*z*): 370.3182 [M + H]^+^ (calcd for C_25_H_40_NO^+^: 370.3110).

(*E*)-cyclosuffrobuxinine-M (**15**) and (*Z*)-cyclosuffrobuxinine-M (**16**) (69.9%:30.1%): light yellow gum; ^1^H NMR (600 MHz, CDCl_3_; δ (ppm), intensity, mult., J (Hz)): 6.63 (1H, q, 7.5, H-20 (*E*)), 5.78 (1H, q, 7.3, H-20 (*Z*)), 4.92 (1H, s, H-29α), 4.87 (1H, s, H-29β), 3.56 (1H, m, H-3), 2.85 (3H, s, H-31/32), 2.32 (1H, m, H-2α), 2.26 (1H, m, H-15α (*Z*)), 2.23 (1H, m, H-15α (*E*)), 2.20 (1H, m, H-11α (*E*)), 2.15 (1H, d, 4.2, H-5), 2.12 (3H, d, 7.4, H-21 (*Z*)), 2.10 (1H, m, H-11α (*Z*)), 2.10 (2H, m, H-12α (*E*)), 2.06 (1H, m, H-15β (*E*)), 2.03 (1H, m, H-15β (*Z*)), 1.85 (3H, d, 7.5, H-21 (*E*)), 1.81 (1H, m, H-1α), 1.75 (1H, dd, 12.4, 4.7, H-8), 1.72 (1H, m, H-12β (*Z*)), 1.60 (1H, m, H-2β), 1.57 (1H, m, H-6α), 1.51 (1H, m, H-1β), 1.42 (1H, m, H-7α), 1.37 (1H, m, H-11β), 1.33 (3H, s, H-18 (*E*)), 1.25 (3H, s, H-18 (*Z*)), 1.19 (1H, dd, 12.6, 2.3, H-7β), 1.09 (1H, qd, 12.7, 2.3, H-6β), 0.98 (3H, s, H-28 (*E*)), 0.95 (3H, s, H-28 (*Z*)), 0.44 (1H, d, 4.5, H-19α), 0.22 (1H, d, 4.7, H-19β (*E*)), 0.19 (1H, d, 4.8, H-19β (*Z*));

^13^C NMR (150 MHz, CDCl_3_; δ (ppm)): 209.46 (qC, C-16 (*Z*)), 207.28 (qC, C-16 (*E*)), 146.72 (qC, C-17 (*Z*)), 146.51 (qC, C-4 (*Z*)), 146.46 (qC, C-4 (*E*)), 146.45 (qC, C-17 (*E*)), 132.91 (CH, C-20 (*Z*)), 131.46 (CH, C-20 (*E*)), 104.37 (CH_2_, C-29 (*Z*)), 104.36 (CH_2_, C-29 (*E*)), 62.83 (CH, C-3 (*Z*)), 62.81 (CH, C-3 (*E*)), 50.76 (CH_2_, C-15 (*Z*)), 49.30 (CH_2_, C-15 (*E*)), 46.84 (qC, C-13 (*E*)), 46.74 (qC, C-13 (*Z*)), 45.40 (CH, C-8 (*E*)), 45.38 (CH, C-8 (*Z*)), 44.26 (CH, C-5 (*E*)), 44.18 (CH, C-5 (*Z*)), 42.58 (qC, C-14 (*E*)), 42.11 (qC, C-14 (*Z*)), 31.79 (CH_3_, C-31/32 (*Z*)), 31.77 (CH_3_, C-31/32 (*E*)), 31.65 (qC, C-10), 31.03 (CH_2_, C-1 (*E*)), 30.95 (CH_2_, C-1 (*Z*)), 30.66 (CH_2_, C-2), 29.32 (CH_2_, C-12 (*E*)), 28.50 (CH_2_, C-12 (*Z*)), 27.92 (CH_2_, C-19 (*E*)), 27.83 (CH_2_, C-19 (*Z*)), 26.65 (CH_2_, C-11 (*E*)), 26.48 (CH_2_, C-11 (*Z*)), 26.40 (CH_3_, C-18 (*Z*)), 25.35 (CH_2_, C-7 (*E*)), 25.25 (CH_2_, C-7 (*Z*)), 24.30 (CH_3_, C-18 (*E*)), 23.55 (qC, C-9 (*Z*)), 23.41 (qC, C-9 (*E*)), 23.24 (CH_2_, C-6 (*E*)), 23.14 (CH_2_, C-6 (*Z*)), 20.99 (CH_3_, C-28 (*E*)), 20.59 (CH_3_, C-28 (*Z*)), 14.40 (CH_3_, C-21 (*Z*)), 13.46 (CH_3_, C-21 (*E*)).

+ESI-QqTOF-MS (*m*/*z*): 354.2848 [M + H]^+^ (calcd for C_24_H_36_NO^+^: 354.2797).

Cyclomicrobuxinine (**17**): light yellow powder; ^1^H NMR (600 MHz, CDCl_3_; δ (ppm), intensity, mult., J (Hz)): 4.90 (1H, ddd, 9.5, 6.6, 2.0, H-16), 4.85 (1H, s, H-29α), 4.61 (1H, s, H-29β), 3.02 (1H, d, 6.6, H-17), 2.95 (1H, dd, 11.8, 4.2, H-3), 2.53 (3H, s, H-31/32), 2.19 (1H, m, H-11α), 2.17 (1H, m, H-2α), 2.15 (3H, s, H-21), 2.10 (1H, m, H-5), 2.02 (1H, m, H-12α), 1.94 (1H, m, H-15α), 1.76 (1H, m, H-1α), 1.71 (1H, m, H-12β), 1.54 (1H, m, H-8), 1.51 (1H, m, H-6α), 1.43 (1H, dd, 13.9, 2.0, H-15β), 1.36 (1H, m, H-7α), 1.34 (1H, m, H-1β), 1.26 (1H, m, H-11β), 1.23 (1H, m, H-2β), 1.21 (3H, s, H-28), 1.17 (1H, m, H-7β), 1.04 (1H, m, H-6β), 0.90 (3H, s, H-18), 0.31 (1H, d, 4.4, H-19α), 0.07 (1H, d, 4.5, H-19β);

^13^C NMR (150 MHz, CDCl_3_; δ (ppm)): 209.61 (qC, C-20), 153.08 (qC, C-4), 101.59 (CH_2_, C-29), 72.04 (CH, C-16), 70.66 (CH, C-17), 63.65 (CH, C-3), 48.51 (qC, C-14), 47.77 (qC, C-13), 47.28 (CH, C-8), 45.93 (CH_2_, C-15), 44.34 (CH, C-5), 34.39 (CH_3_, C-31/32), 34.27 (CH_2_, C-2), 32.44 (qC, C-10), 31.90 (CH_2_, C-1), 31.54 (CH_2_, C-12), 31.37 (CH_3_, C-21), 27.81 (CH_2_, C-19), 26.87 (CH_2_, C-11), 25.52 (CH_2_, C-7), 23.68 (CH_2_, C-6), 22.85 (qC, C-9), 20.75 (CH_3_, C-18), 20.71 (CH_3_, C-28).

+ESI-QqTOF-MS (*m*/*z*): 372.2963 [M + H]^+^ (calcd for C_24_H_38_NO_2_^+^: 372.2903).

Cyclomicrobuxine (**18**): light yellow powder; ^1^H NMR (600 MHz, CDCl_3_; δ (ppm), intensity, mult., J (Hz)): 5.07 (1H, s, H-29α), 4.90 (1H, m, H-16), 4.75 (1H, s, H-29β), 3.02 (1H, d, 6.7, H-17), 2.96 (1H, m, H-3), 2.49 (6H, br s, H-31/32), 2.19 (1H, m, H-11α), 2.16 (3H, s, H-21), 2.09 (1H, m, H-5), 2.06 (1H, m, H-2α), 2.02 (1H, m, H-12α), 1.94 (1H, m, H-15α), 1.72 (1H, m, H-12β), 1.57 (1H, m, H-6α), 1.54 (1H, dd, 12.1, 4.7, H-8), 1.44 (1H, m, H-15β), 1.43 (1H, m, H-1α), 1.42 (1H, m, H-2β), 1.36 (1H, m, H-7α), 1.27 (1H, m, H-1β), 1.26 (1H, m, H-11β), 1.21 (3H, s, H-28), 1.19 (1H, m, H-7β), 1.02 (1H, m, H-6β), 0.92 (3H, s, H-18), 0.33 (1H, d, 4.5, H-19α), 0.08 (1H, d, 4.5, H-19β);

^13^C NMR (150 MHz, CDCl_3_; δ (ppm)): 209.53 (qC, C-20), 152.64 (qC, C-4), 104.62 (CH_2_, C-29), 72.08 (CH, C-16), 70.63 (CH, C-17), 68.64 (CH, C-3), 48.49 (qC, C-14), 47.76 (qC, C-13), 47.29 (CH, C-8), 45.94 (CH_2_, C-15), 44.52 (CH, C-5), 42.47 (CH_3_, C-31/32), 32.08 (qC, C-10), 31.93 (CH_2_, C-1), 31.51 (CH_2_, C-12), 31.37 (CH_3_, C-21), 27.10 (CH_2_, C-2), 27.76 (CH_2_, C-19), 26.90 (CH_2_, C-11), 25.53 (CH_2_, C-7), 23.86 (CH_2_, C-6), 22.78 (qC, C-9), 20.75 (CH_3_, C-18), 20.74 (CH_3_, C-28).

+ESI-QqTOF-MS (*m*/*z*): 386.3094 [M + H]^+^ (calcd for C_25_H_40_NO_2_^+^: 386.3059).

Irehine (**19**): light yellow powder; ^1^H NMR (600 MHz, CD_3_OD; δ (ppm), intensity, mult., J (Hz)): 5.34 (1H, m, H-6), 3.40 (1H, m, H-3), 3.35 (1H, m, H-20), 2.87 (3H, s, H-33/34), 2.70 (3H, s, H-33/34), 2.24 (1H, m, H-4α), 2.21 (1H, m, H-4β), 2.04 (1H, m, H-7α), 2.00 (1H, m, H-7β), 1.98 (1H, m, H-12α), 1.91 (1H, m, H-16α), 1.88 (1H, m, H-1α), 1.81 (1H, m, H-15α), 1.79 (1H, m, H-2α), 1.69 (1H, m, H-17), 1.62 (1H, m, H-11α), 1.55 (1H, m, H-16β), 1.54 (1H, m, H-8), 1.51 (1H, m, H-11β), 1.49 (1H, m, H-2β), 1.33 (1H, m, H-12β), 1.33 (3H, d, 6.6, H-21), 1.31 (1H, m, H-15β), 1.20 (1H, m, H-14), 1.08 (1H, m, H-1β), 1.04 (3H, s, H-19), 1.00 (1H, m, H-9), 0.79 (3H, s, H-18);

^13^C NMR (150 MHz, CD_3_OD; δ (ppm)): 142.33 (qC, C-5), 122.12 (CH, C-6), 72.36 (CH, C-3), 67.01 (CH, C-20), 57.58 (CH, C-14), 53.13 (CH, C-17), 51.41 (CH, C-9), 43.99 (qC, C-13), 43.36 (CH_3_, C-33/34), 42.95 (CH_2_, C-4), 38.51 (CH_2_, C-1), 37.64 (qC, C-10), 35.73 (CH_3_, C-33/34), 33.08 (CH, C-8), 32.81 (CH_2_, C-7), 32.25 (CH_2_, C-2), 29.36 (CH_2_, C-12), 26.83 (CH_2_, C-16), 25.28 (CH_2_, C-15), 22.04 (CH_2_, C-11), 19.82 (CH_3_, C-19), 12.26 (CH_3_, C-18), 11.96 (CH_3_, C-21).

+ESI-QqTOF-MS (*m*/*z*): 346.3215 [M + H]^+^ (calcd for C_23_H_40_NO^+^: 346.3110).

16-α-hydroxybuxaminone (**20**): light yellow powder; ^1^H NMR (600 MHz, CDCl_3_; δ (ppm), intensity, mult., J (Hz)): 5.99 (1H, s, H-19), 5.63 (1H, br s, H-11), 4.93 (1H, m, H-16), 3.02 (1H, dd, 12.9, 4.0, H-3), 2.99 (1H, d, 6.6, H-17), 2.94 (3H, s, H-31/32), 2.79 (3H, s, H-31/32), 2.52 (1H, d, 18.4, H-12α), 2.43 (1H, m, H-1α), 2.24 (1H, m, H-1β), 2.20 (1H, m, H-6α), 2.17 (3H, s, H-21), 2.12 (1H, m, H-12β), 2.07 (1H, m, H-5), 2.05 (1H, m, H-15α), 2.02 (1H, m, H-8), 1.95 (1H, m, H-2α), 1.83 (1H, m, H-2β), 1.53 (1H, m, H-7α), 1.49 (1H, dd, 13.8, 1.9, H-15β), 1.40 (1H, m, H-6β), 1.35 (3H, s, H-29), 1.31 (1H, m, H-7β), 0.96 (3H, s, H-30), 0.95 (3H, s, H-28), 0.68 (3H, s, H-18);

^13^C NMR (150 MHz, CDCl_3_; δ (ppm)): 209.07 (qC, C-20), 138.03 (qC, C-9), 131.99 (qC, C-10), 131.05 (CH, C-19), 129.94 (CH, C-11), 76.34 (CH, C-3), 71.76 (CH, C-16), 69.03 (CH, C-17), 51.77 (CH, C-5), 48.94 (CH, C-8), 48.15 (qC, C-14), 47.12 (CH_3_, C-31/32), 45.78 (qC, C-13), 43.66 (CH_2_, C-15), 41.41 (qC, C-4), 40.47 (CH_3_, C-31/32), 39.41 (CH_2_, C-1), 37.36 (CH_2_, C-12), 31.23 (CH_3_, C-21), 29.53 (CH_2_, C-6), 25.31 (CH_2_, C-7), 24.24 (CH_3_, C-29), 23.45 (CH_2_, C-2), 18.45 (CH_3_, C-18), 18.36 (CH_3_, C-28), 15.13 (CH_3_, C-30).

+ESI-QqTOF-MS (*m*/*z*): 400.3287 [M + H]^+^ (calcd for C_26_H_42_NO_2_^+^: 400.3216).

N_20_-acetylbuxamine-E (**21**): colorless gum; ^1^H NMR (600 MHz, CD_3_OD; δ (ppm), intensity, mult., J (Hz)): 6.05 (1H, s, H-19), 5.61 (1H, br s, H-11), 3.92 (1H, m, H-20), 3.29 (1H, m, H-3), 2.97 (3H, s, H-31/32), 2.79 (3H, s, H-31/32), 2.41 (1H, m, H-1α), 2.27 (1H, m, H-1β), 2.21 (1H, m, H-5), 2.21 (1H, m, H-7α), 2.18 (1H, m, H-12α), 2.14 (1H, m, H-8), 2.08 (1H, m, H-12β), 2.06 (1H, m, H-2α), 1.92 (1H, m, H-16α), 1.91 (1H, m, H-17), 1.90 (3H, s, Ac-CH_3_), 1.84 (1H, m, H-2β), 1.59 (1H, m, H-6α), 1.50 (1H, dd, 12.0, 6.2, H-15α), 1.45 (1H, m, H-15β), 1.42 (1H, m, H-16β), 1.32 (1H, m, H-6β), 1.29 (1H, m, H-7β), 1.20 (3H, s, H-29), 1.10 (3H, d, 6.4, H-21), 0.91 (3H, s, H-30), 0.81 (3H, s, H-18), 0.74 (3H, s, H-28);

^13^C NMR (150 MHz, CD_3_OD; δ (ppm)): 171.86 (qC, Ac-**C**O), 139.24 (qC, C-9), 133.06 (qC, C-10), 131.85 (CH, C-19), 131.62 (CH, C-11), 77.22 (CH, C-3), 52.26 (CH, C-17), 52.04 (CH, C-5), 50.73 (CH, C-8), 49.84 (qC, C-14), 49.55 (CH, C-20), 47.24 (CH_3_, C-31/32), 44.22 (qC, C-13), 41.93 (qC, C-4), 40.87 (CH_3_, C-31/32), 40.08 (CH_2_, C-1), 39.21 (CH_2_, C-12), 33.88 (CH_2_, C-15), 30.73 (CH_2_, C-7), 27.45 (CH_2_, C-16), 26.27 (CH_2_, C-6), 24.22 (CH_2_, C-2), 23.78 (CH_3_, C-29), 22.67 (CH_3_, Ac-**C**H_3_), 21.18 (CH_3_, C-21), 17.38 (CH_3_, C-28), 16.13 (CH_3_, C-18), 15.07 (CH_3_, C-30).

+ESI-QqTOF-MS (*m*/*z*): 427.3719 [M + H]^+^, 214.1903 [M + 2H]^2+^ (calcd for C_28_H_47_N_2_O^+^: 427.3688, for C_28_H_48_N_2_O^2+^: 214.1884); MS/MS (*m*/*z*): 382 [M-(CH_3_)_2_NH]^+^, 340 [382-CH_2_=CO]^+^, 323 [340-NH_3_]^+^.

N-benzoyl-O-acetylbuxodienine-E (**22**): white powder; UV (MeOH; λmax, (log ε)): 237 (4.54), 245 (4.53), 253 (4.35); ^1^H NMR (600 MHz, CD_3_OD; δ (ppm), intensity, mult., J (Hz)): 7.79 (2H, m, H-2′/6′), 7.51 (1H, m, H-4′), 7.45 (2H, m, H-3′/5′), 5.94 (1H, br s, H-19), 5.55 (1H, br m, H-11), 5.06 (1H, ddd, 9.0, 6.6, 1.4, H-16), 4.43 (1H, m, H-20), 2.37 (1H, m, H-17), 2.35 (1H, m, H-12α), 2.30 (6H, s, H-31/32), 2.30 (1H, m, H-1α), 2.22 (1H, m, H-3), 2.15 (1H, m, H-6α), 2.13 (1H, m, H-1β), 2.13 (1H, m, H-12β), 2.13 (1H, m, H-15α), 2.10 (1H, m, H-8), 2.01 (1H, m, H-5), 1.80 (1H, dd, 12.0, 3.4, H-2α), 1.62 (3H, s, Ac-CH_3_), 1.55 (1H, qd, 14.1, 5.5, H-2β), 1.47 (1H, m, H-7α), 1.42 (1H, m, H-6β), 1.35 (1H, dd, 14.3, 1.5, H-15β), 1.30 (1H, m, H-7β), 1.28 (3H, d, 6.7, H-21), 1.03 (3H, s, H-29), 0.92 (3H, s, H-18), 0.89 (3H, s, H-28), 0.74 (3H, s, H-30);

^13^C NMR (150 MHz, CD_3_OD; δ (ppm)): 172.39 (qC, Ac-**C**O), 168.63 (qC, O**C**NH), 139.68 (qC, C-9), 137.46 (qC, C-10), 135.84 (qC, C-1′), 132.55 (CH, C-4′), 129.65 (CH, C-19), 129.46 (CH, C-3′/5′), 129.13 (CH, C-11), 128.28 (CH, C-2′/6′), 80.75 (CH, C-16), 73.14 (CH, C-3), 57.90 (CH, C-17), 53.15 (CH, C-5), 50.43 (CH, C-8), 48.31 (qC, C-14), 48.17 (CH, C-20), 44.86 (qC, C-13), 44.79 (CH_3_, C-31/32), 44.12 (qC, C-4), 43.79 (CH_2_, C-15), 42.26 (CH_2_, C-1), 38.88 (CH_2_, C-12), 31.18 (CH_2_, C-6), 26.63 (CH_2_, C-7), 25.29 (CH_3_, C-29), 24.19 (CH_2_, C-2), 20.96 (CH_3_, Ac-**C**H_3_), 20.68 (CH_3_, C-21), 17.89 (CH_3_, C-28), 17.02 (CH_3_, C-18), 15.46 (CH_3_, C-30).

+ESI-QqTOF-MS (*m*/*z*): 547.3977 [M + H]^+^, 274.2040 [M + 2H]^2+^ (calcd for C_35_H_51_N_2_O_3_^+^: 547.3900, for C_35_H_52_N_2_O_3_^2+^: 274.1989); MS/MS (*m*/*z*): 442 [M-((CH_3_)_2_NH)-(CH_3_COOH)]^+^, 321 [442-C_7_H_7_NO]^+^, 148 [C_9_H_10_NO]^+^, 105 [C_7_H_5_O]^+^.

N-benzoyl-O-acetylbuxadine-E (**23**): white powder; UV (MeOH; λmax, (log ε)): 225 (4.01); ^1^H NMR and ^13^C NMR (600/150 MHz, CD_3_OD) see Table 3;

+ESI-QqTOF-MS (*m*/*z*): 1093.7853 [2M + H]^+^, 547.3917 [M + H]^+^, 274.2038 [M + 2H]^2+^ (calcd for C_35_H_51_N_2_O_3_^+^: 547.3900, for C_35_H_52_N_2_O_3_^2+^: 274.1989); MS/MS (*m*/*z*): 442 [M-((CH_3_)_2_NH)-(CH_3_COOH)]^+^, 321 [442-C_7_H_7_NO]^+^, 148 [C_9_H_10_NO]^+^, 105 [C_7_H_5_O]^+^ (fragmentation pathway reported in Figure S122, Supplementary Materials).

N_20_-acetylbuxadine-G (**24**): colorless gum; ^1^H NMR and ^13^C NMR (600/150 MHz, CDCl_3_) see Table 3;

+ESI-QqTOF-MS (*m*/*z*): 413.0273 [M + H]^+^, 207.4721 [M + 2H]^2+^ (calcd for C_27_H_45_N_2_O^+^: 413.3532, for C_27_H_46_N_2_O^2+^: 207.1805); MS/MS (*m*/*z*): 382 [M-CH_3_NH_2_]^+^, 323 [382-CH_3_CONH_2_]^+^.

17,20-dihydroxybuxadine-M (**25**): colorless gum; ^1^H NMR and ^13^C NMR (600/150 MHz, CDCl_3_) see Table 3;

+ESI-QqTOF-MS (*m*/*z*): 388.3272 [M + H]^+^ (calcd for C_25_H_42_NO_2_^+^: 388.3216); MS/MS (*m*/*z*): 339 [M-(CH_3_NH_2_)-(H_2_O)]^+^, 321 [339-H_2_O]^+^.

### 3.6. In Vitro Bioassays

In vitro assays for the bioactivity of the isolated *Buxus*-alkaloids against *Tbr* (bloodstream trypomastigotes, STIB 900 strain), *Trypanosoma cruzi* (*Tc*) (amastigotes, Tulahuen C4 strain), *Leishmania donovani* (*Ldo*) (amastigotes, MHOM-ET-67/L82 strain) and *Pf* (intraerythrocytic forms, NF54 strain), and cytotoxicity tests against mammalian cells (L6-cell line from rat-skeletal myoblasts), were performed at the Swiss Tropical and Public Health Institute (Swiss TPH, Basel, Switzerland) according to established standard protocols [36]. The alkaloids were inactive against *Tc* and *Ldo* at the concentration tested. Such a pattern of activity was also observed in other studies [37].

## 4. Conclusions

The present study provides an extended chemical analysis of alkaloids isolated from a *B*. *sempervirens* L. leaf extract. The structure of eight new natural products could be elucidated and the NMR data of already known *Buxus*-alkaloids are reported here in full for the first time. Moreover, cyclomicrophyllidine-A (**6**) was primarily obtained from the leaves of *B*. *sempervirens* L. Several of the isolated compounds displayed promising and selective in vitro activity against the protozoan parasites *Pf* and *Tbr*. Of the 25 structures isolated, five compounds (**3**, **4**, **6**–**8**) showed auspicious antiplasmodial activity with IC_50_ values of <1.0 μM. Against the causative agent of HAT, compound **2**, **6**, **8,** and **24** exhibited interesting IC_50_ values in the range of 1.1–1.5 µM. The new natural product O-benzoyl-cycloprotobuxoline-D (**8**) was the most active substance in both cases. The highest selectivity was reached by cyclomicrophyllidine-B (**7**) (SI 145 for *Pf*) and cyclomicrophylline-A (**5**) (SI 42 for *Tbr*), respectively. Accordingly, these *Buxus*-alkaloids have the potential to serve as antiprotozoal lead structures. Structure-activity relationship studies are in progress in order to identify structural functionalities that increase activity and reduce toxicity. In conclusion, the work presented here may represent an important first step in the development of this new class of antiprotozoal drugs against malaria and HAT.

## Figures and Tables

**Table 1:** Chemical structures of isolated *Buxus*-alkaloids **1–25**. For core skeletal structures see Figure 1.

Cpd	Structure
	Core skeleton **A**:
**1**	R_1+4_: H, R_2_: N(CH_3_)_2_, R_3_: (CH_3_)_2_, Δ^6^, R_5_: O-tiglate, R_6_: NHCH_3_
**2**	R_1+4_: H, R_2_: N(CH_3_)_2_, R_3_: (CH_3_)_2_, Δ^6^, R_5_: OH, R_6_: NHCH_3_
**3**	R_1+4_: H, R_2_: N(CH_3_)_2_, R_3_: CH_3_+CH_2_OH, Δ^6^, R_5_: O-tiglate, R_6_: NHCH_3_
**4**	R_1+4_: H, R_2_: N(CH_3_)_2_, R_3_: CH_3_+CH_2_OH, Δ^6^, R_5_: O-tiglate, R_6_: N(CH_3_)_2_
**5**	R_1+4_: H, R_2_: N(CH_3_)_2_, R_3_: CH_3_+CH_2_OH, Δ^6^, R_5_: OH, R_6_: N(CH_3_)_2_
**6**	R_1+4_: H, R_2_: N(CH_3_)_2_, R_3_: CH_3_+CH_2_OH, Δ^6^, R_5_: O-benzoate, R_6_: N(CH_3_)_2_
**7**	R_1+4_: H, R_2_: N(CH_3_)_2_, R_3_: CH_3_+CH_2_OH, Δ^6^, R_5_: O-benzoate, R_6_: NHCH_3_
**8**	R_1_: O-benzoate, R_2_: NHCH_3_, R_3_: (CH_3_)_2_, R_4+5_: H, R_6_: NHCH_3_
**9**	R_1+5_: H, R_2_: benzamide, R_3_: CH_3_+CH_2_OAc, R_4_: =O, R_6_: N(CH_3_)_2_
**10**	R_1+5_: H, R_2_: benzamide, R_3_: CH_3_+CH_2_OH, R_4_: =O, R_6_: N(CH_3_)_2_
**11**	R_1+4_: H, R_2_: =O, R_3_: CH_3_+CH_2_OH, R_5_: OH, R_6_: N(CH_3_)_2_
**12**	R_1+4_: H, Δ^1,2^, R_2_: =O, R_3_: (CH_3_)_2_, R_5_: OH, R_6_: N(CH_3_)_2_
**13**	R_1+4+6_: H, R_2_: NHCH_3_, R_3_: (CH_3_)_2_, R_5_: =O, Δ^17(20)^ (*E*)
**14**	R_1+4+6_: H, R_2_: NHCH_3_, R_3_: (CH_3_)_2_, R_5_: =O, Δ^17(20)^ (*Z*)
**15**	R_1+4+6_: H, R_2_: NHCH_3_, R_3_: =CH_2_, R_5_: =O, Δ^17(20)^ (*E*)
**16**	R_1+4+6_: H, R_2_: NHCH_3_, R_3_: =CH_2_, R_5_: =O, Δ^17(20)^ (*Z*)
**17**	R_1+4_: H, R_2_: NHCH_3_, R_3_: =CH_2_, R_5_: OH, R_6_: =O
**18**	R_1+4_: H, R_2_: N(CH_3_)_2_, R_3_: =CH_2_, R_5_: OH, R_6_: =O
	Core skeleton **B**:
**19**	R_1_: OH, R_2_: N(CH_3_)_2_
	Core skeleton C:
**20**	R_1_: N(CH_3_)_2_, R_2_: OH, R_3_: H_,_ R_4_: =O
**21**	R_1_: N(CH_3_)_2_, R_2+3_: H, R_4_: NHAc
**22**	R_1_: N(CH_3_)_2_, R_2_: OAc, R_3_: H_,_ R_4_: benzamide
**23**	R_1_: N(CH_3_)_2_, R_2_: OAc, R_3_: H_,_ R_4_: benzamide
**24**	R_1_: NHCH_3_, R_2+3_: H, R_4_: NHAc
**25**	R_1_: NHCH_3_, R_2_: H, R_3+4_: OH

**Table 2:** ^1^H- and ^13^C-NMR data of compounds **3** (600/150 MHz, CDCl_3_), **4**, **7** and **8** (600/150 MHz, CD_3_OD).

	3		4		7		8	
Pos.	δ_C_ [ppm]	δ_H_ [ppm], mult., J [Hz]	δ_C_ [ppm]	δ_H_ [ppm], mult., J [Hz]	δ_C_ [ppm]	δ_H_ [ppm], mult., J [Hz]	δ_C_ [ppm]	δ_H_ [ppm], mult., J [Hz]
1	29.82, CH_2_	1.74, d, 12.4 1.69, m	31.72, CH_2_	1.63, m (2H)	30.44, CH_2_	1.75, m (2H)	38.75, CH_2_	1.98, dd, 12.5, 4.9 1.79, m
2	20.01, CH_2_	1.96, m 1.87, m	19.62, CH_2_	1.82, m 1.68, m	20.94, CH_2_	2.07, dd, 12.5, 3.5 1.92, m	74.49, CH	5.30, td, 10.9, 4.8
3	72.94, CH	3.46, dd, 12.8, 3.5	73.29, CH	2.71, dd, 12.3, 3.4	75.62, CH	3.49, m	73.20, CH	3.35, d, 11.0
4	42.69, qC	-	43.36, qC	-	43.21, qC	-	41.53, qC	-
5	43.78, CH	2.19, m	45.82, CH	1.95, s	45.18, CH	2.11, br s	48.78, CH	1.73, m
6	125.03, CH	5.54, d, 10.8	126.72, CH	5.49, m	126.06, CH	5.52, m	21.93, CH_2_	1.77, m 0.94, m
7	129.83, CH	5.50, ddd, 10.8, 5.8, 2.8	130.42, CH	5.45, ddd, 10.6, 5.8, 2.9	130.96, CH	5.56, m	26.75, CH_2_	1.47, m 1.23, m
8	42.71, CH	2.65, m	44.49, CH	2.63, m	44.05, CH	2.74, m	48.95, CH	1.66, dd, 12.5, 4.8
9	20.74, qC	-	21.72, qC	-	21.80, qC	-	20.91, qC	-
10	27.32, qC	-	29.07, qC	-	28.55, qC	-	25.47, qC	-
11	24.83, CH_2_	1.90, dd, 14.6, 4.7 1.46, m	26.07, CH_2_	1.90, m 1.51, m	25.74, CH_2_	1.99, m 1.58, m	27.10, CH_2_	2.11, m 1.22, m
12	31.87, CH_2_	1.84, dd, 13.1, 4.3 1.48, m	33.43, CH_2_	1.76, dd, 13.5, 5.1 1.46, m	33.13, CH_2_	1.92, m 1.62, dd, 13.5, 5.0	33.11, CH_2_	1.75, m 1.73, m
13	47.53, qC	-	46.27, qC	-	47.88, qC	-	46.89, qC	-
14	49.48, qC	-	50.79, qC	-	50.35, qC	-	50.04, qC	-
15	42.33, CH_2_	2.16, m 1.44, m	43.28, CH_2_	2.08, m 1.11, d, 13.9	43.59, CH_2_	2.39, dd, 14.7, 8.7 1.46, dd, 14.6, 1.2	36.46, CH_2_	1.56, m 1.52, m
16	78.68, CH	5.26, dd, 8.3, 5.5	81.26, CH	5.12, ddd, 8.5, 5.9, 1.0	80.89, CH	5.28, ddd, 8.3, 6.4, 1.3	27.01, CH_2_	2.02, m 1.58, m
17	55.41, CH	2.46, dd, 10.8, 5.5	56.84, CH	2.21, m	55.65, CH	2.58, dd, 10.8, 6.3	51.04, CH	2.06, m
18	15.77, CH_3_	1.04, s	16.28, CH_3_	1.00, s	15.91, CH_3_	1.13, s	18.68, CH_3_	1.08, s
19	17.94, CH_2_	0.83, d, 4.4 −0.03, d, 4.4	19.09, CH_2_	0.79, d, 4.1 −0.07, d, 4.2	18.61, CH_2_	0.85, d, 4.3 0.08, d, 4.5	30.11, CH_2_	0.80, d, 4.6 0.71, d, 4.6
20	57.14, CH	3.41, m	61.37, CH	2.63, m	58.42, CH	3.58, m	60.44, CH	3.24, m
21	15.45, CH_3_	1.41, d, 6.5	10.53, CH_3_	0.89, d, 6.4	15.54, CH_3_	1.41, d, 6.5	15.45, CH_3_	1.31, d, 6.4
28	17.45, CH_3_	0.98, s	18.16, CH_3_	0.98, s	18.46, CH_3_	1.04, s	19.71, CH_3_	1.01, s
29	67.80, CH_2_	4.05, d, 11.6 3.79, d, 11.5	72.91, CH_2_	3.81, d, 10.4 3.52, d, 10.3	70.91, CH_2_	3.92, m 3.65, m	25.16, CH_3_	1.20, s
30	12.89, CH_3_	1.10, s	12.78, CH_3_	1.05, s	12.31, CH_3_	1.14, s	15.76, CH_3_	1.06, s
31/32	45.64, CH_3_	2.96, s (3H)	42.94, CH_3_	2.37, s (6H)	45.40, CH_3_	2.94, s (3H)	37.69, CH_3_	2.96, s (3H)
	39.09, CH_3_	2.77, s (3H)			39.31, CH_3_	2.78, s (3H)		
33/34	29.06, CH_3_	2.66, s (3H)	40.71, CH_3_	2.14, s (6H)	30.01, CH_3_	2.69, s (3H)	29.88, CH_3_	2.67, s (3H)
OCO					167.54, qC	-	166.74, qC	-
1′	169.61, qC	-	169.27, qC	-	131.45, qC	-	130.97, qC	-
2′	127.74, qC	-	130.20, qC	-	130.48, CH	8.03, m	130.53, CH	8.06, m
3′	141.17, CH	6.91, qq, 7.0, 1.4	137.75, CH	6.80, qq, 7.0, 1.3	129.76, CH	7.50, m	129.95, CH	7.53, m
4′	14.81, CH_3_	1.81, m	14.31, CH_3_	1.79, m	134.55, CH	7.63, m	134.86, CH	7.66, m
5′	11.90, CH_3_	1.80, m	12.16, CH_3_	1.81, m	129.76, CH	7.50, m	129.95, CH	7.53, m
6′					130.48, CH	8.03, m	130.53, CH	8.06, m

**Table 3:** ^1^H- and ^13^C-NMR data of compounds **11, 23** (600/150 MHz, CD_3_OD), **24** and **25** (600/150 MHz, CDCl_3_).

	11		23		24		25	
Pos.	δ_C_ [ppm]	δ_H_ [ppm], mult., J [Hz]	δ_C_ [ppm]	δ_H_ [ppm], mult., J [Hz]	δ_C_ [ppm]	δ_H_ [ppm], mult., J [Hz]	δ_C_ [ppm]	δ_H_ [ppm], mult., J [Hz]
1	33.21, CH_2_	1.91, m 1.57, m	121.95, CH	5.42, br s	117.16, CH	5.39, s	116.85, CH	5.39, br s
2	38.83, CH_2_	2.68, m 2.29, m	22.98, CH_2_	2.19, m (2H)	24.92, CH_2_	2.43, m 2.28, m	24.91, CH_2_	2.46, m 2.33, m
3	217.33, qC	-	68.30, CH	2.19, m	65.73, CH	2.80, m	65.87, CH	2.78, m
4	56.13, qC	-	39.17, qC	-	36.60, qC	-	36.46, qC	-
5	42.14, CH	2.33, m	52.91, CH	1.73, m	50.90, CH	1.87, m	50.51, CH	1.87, m
6	22.08, CH_2_	1.62, m 1.01, dd, 12.6, 2.4	31.54, CH_2_	1.97, m 1.73, m	29.98, CH_2_	2.04, m 1.30, m	31.38, CH_2_	2.10, m 1.26, m
7	26.98, CH_2_	1.38, m (2H)	28.41, CH_2_	1.54, dd, 14.0, 5.7 1.44, m	27.79, CH_2_	1.69, m 1.44, m	27.47, CH_2_	1.64, m 1.48, m
8	49.18, CH	1.61, m	48.47, CH	2.03, m	47.44, CH	2.03, m	48.46, CH	2.04, d, 10.2
9	21.31, qC	-	141.29, qC	-	138.47, qC	-	138.16, qC	-
10	26.69, qC	-	141.10, qC	-	140.39, qC	-	140.55, qC	-
11	27.24, CH_2_	2.21, m 1.26, m	119.37, CH	5.31, br m	120.81, CH	5.29, s	120.60, CH	5.35, br s
12	32.60, CH_2_	1.86, m 1.66, m	37.85, CH_2_	2.19, m 1.99, m	36.97, CH_2_	1.97, m 1.90, m	30.87, CH_2_	2.40, m 1.64, m
13	47.36, qC	-	45.34, qC	-	44.02, qC	-	48.06, qC	-
14	48.90, qC	-	48.45, qC	-	48.81, qC	-	48.53, qC	-
15	48.01, CH_2_	2.06, m 1.48, dd, 13.9, 2.3	43.83, CH_2_	2.01, m 1.32, dd, 14.3, 1.4	32.93, CH_2_	1.43, m 1.34, m	33.30, CH_2_	1.55, m 1.48, m
16	77.20, CH	4.31, m	80.73, CH	5.02, ddd, 8.9, 6.6, 1.4	26.47, CH_2_	1.83, m 1.46, m	38.63, CH_2_	2.14, m 1.90, m
17	56.12, CH	2.23, m	58.04, CH	2.34, dd, 10.8, 6.6	51.73, CH	1.76, m	85.50, qC	-
18	19.65, CH_3_	1.13, s	16.62, CH_3_	0.84, s	15.57, CH_3_	0.73, s	17.64, CH_3_	0.73, s
19	30.56, CH_2_	0.83, d, 4.1 0.68, d, 4.4	47.16, CH_2_	2.93, d, 14.2 2.78, d, 14.3	45.87, CH_2_	2.93, m 2.82, m	46.11, CH_2_	2.96, d, 14.6 2.82, m
20	68.22, CH	3.59, m	48.17, CH	4.40, dq, 13.2, 6.5	49.31, CH	3.99, m	73.74, CH	3.84, q, 6.1
21	11.36, CH_3_	1.32, d, 6.6	20.74, CH_3_	1.27, d, 6.6	21.30, CH_3_	1.14, d, 6.3	18.21, CH_3_	1.18, d, 6.1
28	21.28, CH_3_	1.23, s	17.89, CH_3_	0.92, s	17.49, CH_3_	0.72, s	18.56, CH_3_	1.03, s
29	64.35, CH_2_	3.86, d, 11.2 3.32, m	29.41, CH_3_	0.93, s	26.31, CH_3_	1.15, s	27.32, CH_3_	1.14, s
30	16.98, CH_3_	0.97, s	22.83, CH_3_	1.00, s	18.22, CH_3_	1.01, s	19.97, CH_3_	1.09, s
31/32			45.32, CH_3_	2.30, s (6H)	33.38, CH_3_	2.72, s (3H)	34.09, CH_3_	2.75, s (3H)
33/34	43.67, CH_3_	2.96, s (3H)						
	36.65, CH_3_	2.81, s (3H)						
Ac-CH_3_			20.97, CH_3_	1.62, s	23.35, CH_3_	1.99, s		
Ac-CO			172.40, qC	-	170.18, qC	-		
OCNH			168.61, qC	-	-	5.46, d (1H), 9.0		
1′			135.84, qC	-				
2′			128.28, CH	7.78, m				
3′			129.45, CH	7.44, m				
4′			132.54, CH	7.51, m				
5′			129.45, CH	7.44, m				
6′			128.28, CH	7.78, m				

**Table 4:** In vitro antiprotozoal and cytotoxic activity of isolated *Buxus*-alkaloids.

Cpd	*Pf*	*Tbr*	Cytotox.	SI *Pf*	SI *Tbr*
**1**	0.52 ± 0.14 (1.05 µM)	1.6 ± 0.55 * (3.2 µM)	9.4 ± 3.8 (19 µM)	18	6
**2**	1.07 ± 0.11 (2.6 µM)	0.6 ± 0.03 (1.5 µM)	14.7 ± 3.1 (35.5 µM)	14	25
**3**	0.5 ± 0.06 (0.98 µM)	4.6 ± 0.005 (9 µM)	35.8 ± 13.6 (69.9 µM)	72	8
**4**	0.35 ± 0.08 (0.7 µM)	1.5 ± 0.76 * (2.9 µM)	12 ± 1.4 (22.8 µM)	34	8
**5**	0.78 ± 0.08 (1.76 µM)	1.04 ± 0.27 * (2.34 µM)	44 ± 2.3 (99 µM)	56	42
**6**	0.4 ± 0.02 (0.7 µM)	0.69 ± 0.06 (1.3 µM)	9.3 ± 3 (17 µM)	23	14
**7**	0.11 ± 0.01 (0.2 µM)	1.9 ± 0.3 (3.6 µM)	16 ± 0.2 (29.9 µM)	145	8
**8**	0.09 ± 0.03 * (0.18 µM)	0.55 ± 0.18 (1.1 µM)	6.65 ± 0.38 (13.1 µM)	74	12
**9**	2.15 ± 0.29 (3.8 µM)	1.35 ± 0.45 * (2.4 µM)	41 ± 0.7 (73 µM)	19	30
**11**	0.9 ± 0.13 (2.2 µM)	2.9 ± 1 (7 µM)	44 ± 1.3 (105.4 µM)	49	15
**12**	1.4 ± 0.44 * (3.5 µM)	2.5 ± 0.1 (6.3 µM)	19 ± 1.2 (47.6 µM)	14	8
**13** + **14**	1.6 ± 0.18 * (4.3 µM)	0.8 ± 0.06 (2.2 µM)	5.3 ± 0.05 (14.4 µM)	3	7
**15** + **16**	1.1 ± 0.01 (3.1 µM)	0.75 ± 0.001 (2.1 µM)	10.4 ± 4.17 (29.4 µM)	9	14
**17**	4.1 ± 0.8 (10.9 µM)	48 ± 0.2 (129.3 µM)	30.5 ± 9.6 (82 µM)	7	0.6
**18**	2.6 ± 0.005 (6.8 µM)	16.9 ± 2.3 (44 µM)	26.8 ± 8.8 (69.6 µM)	10	2
**19**	1.3 ± 0.35 * (3.8 µM)	2.2 ± 0.07 (6.4 µM)	13.4 ± 0.5 (38.8 µM)	10	6
**20**	1.6 ± 0.57 * (4 µM)	5.26 ± 0.3 (13.2 µM)	45 ± 1 (112.7 µM)	28	9
**21**	3.1 ± 0.24 * (7.3 µM)	53 ± 1 (124 µM)	54.8 ± 1.1 (129 µM)	18	1
**22**	3 ± 0.3 (6.2 µM)	3.4 ± 1 (6.2 µM)	8.5 ± 0.1 (15.6 µM)	3	3
**23**	4.2 ± 0.4 (7.7 µM)	3.1 ± 0.6 (5.7 µM)	27.5 ± 16 (50.4 µM)	7	9
**24**	2.3 ± 0.27 (5.6 µM)	0.52 ± 0.27 * (1.3 µM)	16.9 ± 1.1 (41 µM)	7	33
**25**	3.6 ± 0.05 (9.3 µM)	6.7 ± 0.49 (17.3 µM)	44.5 ± 5.5 (114.9 µM)	12	7
**Chloroquine**	0.006 ± 0.001 (0.019 µM)				
**Melarsoprol**		0.005 ± 0.001 (0.013 µM)			
**Podophyllotoxin**			0.006 ± 0.001 (0.014 µM)		

All IC_50_ values are expressed in µg/mL and values in µM are given in parentheses. While * *n* = 3 was reported as the mean value from three independent measurements with the standard deviation, all other values were determined with *n* = 2 as the mean value from two independent measurements with the fluctuation range. Note that the purity of compound **10** was <90%, so that activity was not determined.

## Data Availability

Data is contained within the article and Supplementary Materials. The underlying raw data are available from the corresponding author on request.

## Supplementary Materials

The following are available online at https://www.mdpi.com/article/10.3390/antibiotics10060696/s1, the ^1^H and ^13^C NMR spectra of all the isolated compounds are provided as Supplementary Figures S1–S4, S8–S11, S21–S24, S31–S38, S42–S45, S54–S57, S63–S70, S74–S77, S82–S118, S124–S127, S137–S140, and S149–S152. For the new natural products, additional LC/MS chromatograms, UV, and 2D NMR spectra, as well as key correlations of HMBC and COSY spectra, are supplied as Supplementary Figures S5–S7, S12–S20, S25–S30, S39–S41, S46–S53, S58–S62, S71–S73, S78–S81, S119–S123, S128–S136, S141–S148, and S153–158.
