# Supplementary material for: Antiprotozoal Nor-Triterpene Alkaloids from Buxus sempervirens L
Source: Antibiotics (Basel). 2021 Jun 10;10(6):696. doi: 10.3390/antibiotics10060696 (PMC8228141; doi:10.3390/antibiotics10060696)
Supplement: Supplementary file 1 [file antibiotics-10-00696-s001.zip › antibiotics-1247205-supplementary.pdf]

antibiotics

MDPI

Article

Antiprotozoal *Nor*-Triterpene Alkaloids from *Buxus sempervirens* L.

Lara U. Szabó [1], Marcel Kaiser [2,3], Pascal Mäser [2,3] and Thomas J. Schmidt [1,*]

[1] Institute of Pharmaceutical Biology and Phytochemistry (IPBP), University of Münster, Pharma Campus Corrensstraße 48, D-48149 Münster, Germany; lszabo@uni-muenster.de
[2] Swiss Tropical and Public Health Institute (Swiss TPH), Socinstrasse 57, CH-4051 Basel, Switzerland; marcel.kaiser@unibas.ch (M.K.); pascal.maeser@swisstph.ch (P.M.)
[3] University of Basel, Petersplatz 1, CH-4003 Basel, Switzerland
* Correspondence: thomschm@uni-muenster.de; Tel.: +49-251-83-33378

Citation: Szabó, L.U.; Kaiser, M.; Mäser, P.; Schmidt, T.J. Antiprotozoal *Nor*-Triterpene Alkaloids from *Buxus sempervirens* L. *Antibiotics* **2021**, *10*, x. https://doi.org/10.3390/xxxxx

Received: 19 May 2021
Accepted: 8 June 2021
Published:

Publisher's Note: MDPI stays neutral with regard to jurisdictional claims in published maps and institutional affiliations.

Copyright: © 2021 by the authors. Licensee MDPI, Basel, Switzerland. This article is an open access article distributed under the terms and conditions of the Creative Commons Attribution (CC BY) license (http://creativecommons.org/licenses/by/4.0/).

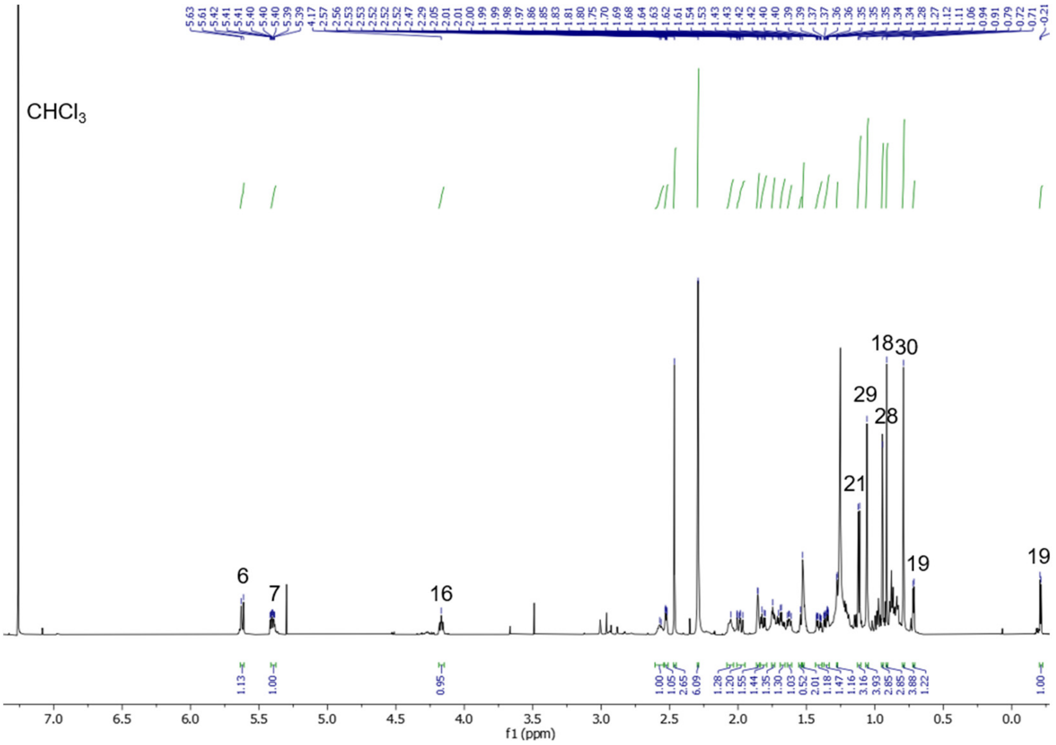

Figure S1. ^{1}H NMR spectrum of Cyclovirobuxeine-B (**2**) ($CDCl_3$, 600 MHz). The assignment of the signals between 1.275 and 2.6 ppm can be found in the enlarged Figure S2.

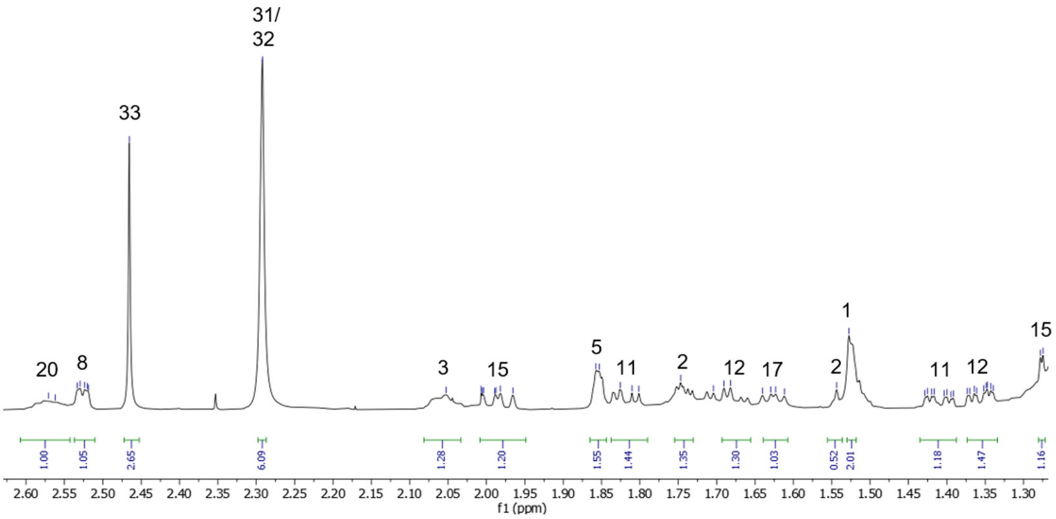

Figure S2. Detail of the ^{1}H NMR spectrum of Cyclovirobuxeine-B (**2**) ($CDCl_3$, 600 MHz).

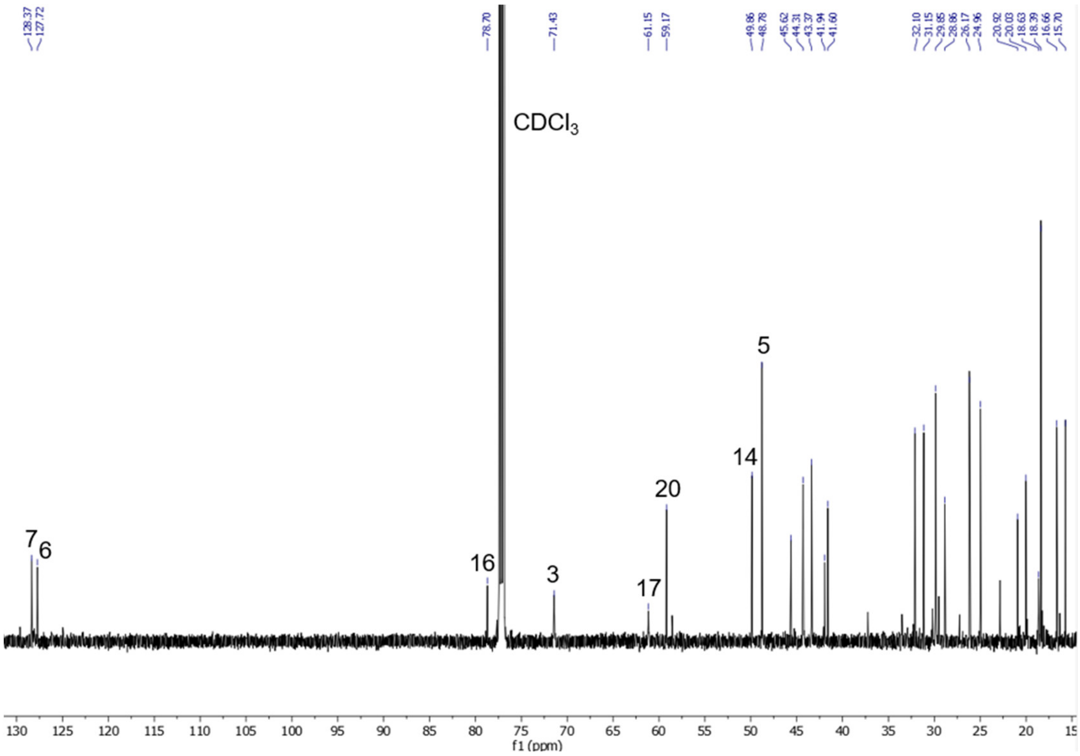

Figure S3. ^{13}C NMR spectrum of Cyclovirobuxeine-B (**2**) ($CDCl_3$, 150 MHz). The assignment of the signals between 15 and 46 ppm can be found in the enlarged Figure S4.

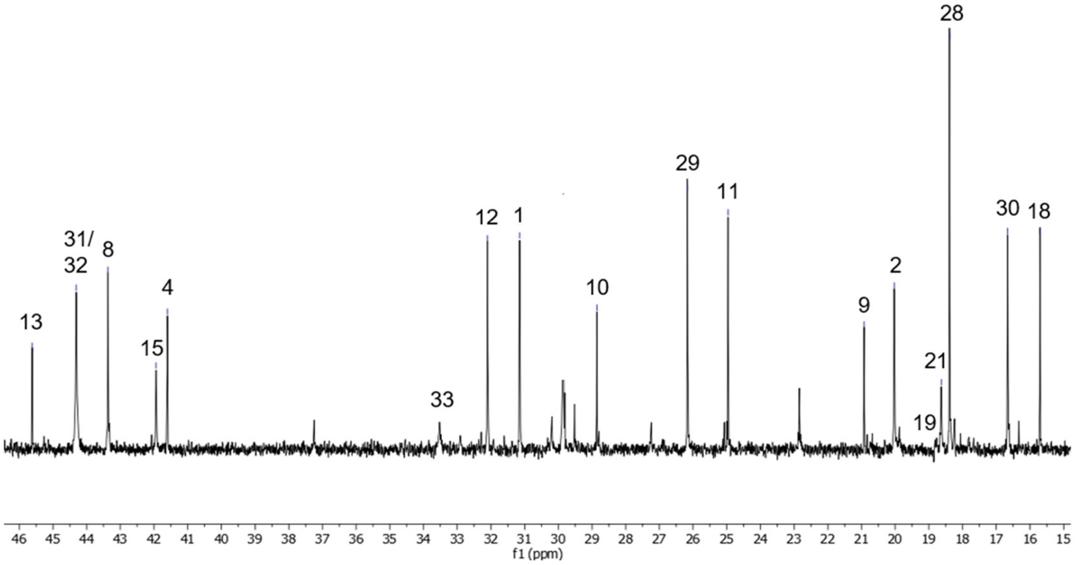

Figure S4. Detail of the ^{13}C NMR spectrum of Cyclovirobuxeine-B (**2**) ($CDCl_3$, 150 MHz).

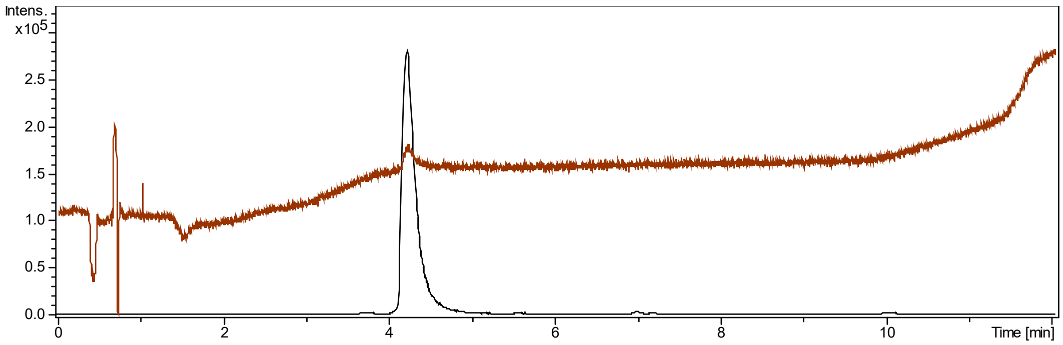

Figure S5. UHPLC/+ESI-QqTOF-MS/MS chromatogram of O-tigloylcyclomicrophylline-B (**3**). Base peak chromatogram 200.0000-1000.0000 +All MS (black); UV-Chromatogramm, 200-400 nm (red).

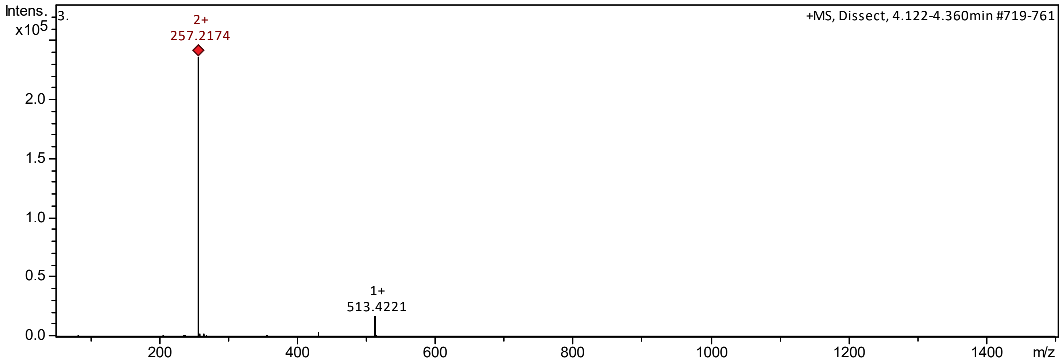

Figure S6. +ESI-QqTOF MS spectrum of O-tigloylcyclomicrophylline-B (**3**); *m/z* 257.2174 $[M+2H]^{2+}$ and 513.4221 $[M+H]^{+}$.

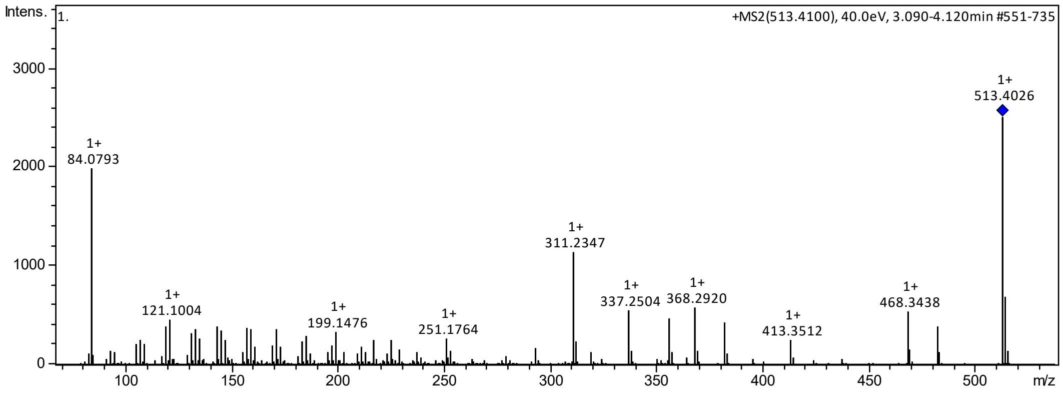

Figure S7. +ESI-QqTOF MS/MS spectrum of O-tigloylcyclomicrophylline-B (**3**).

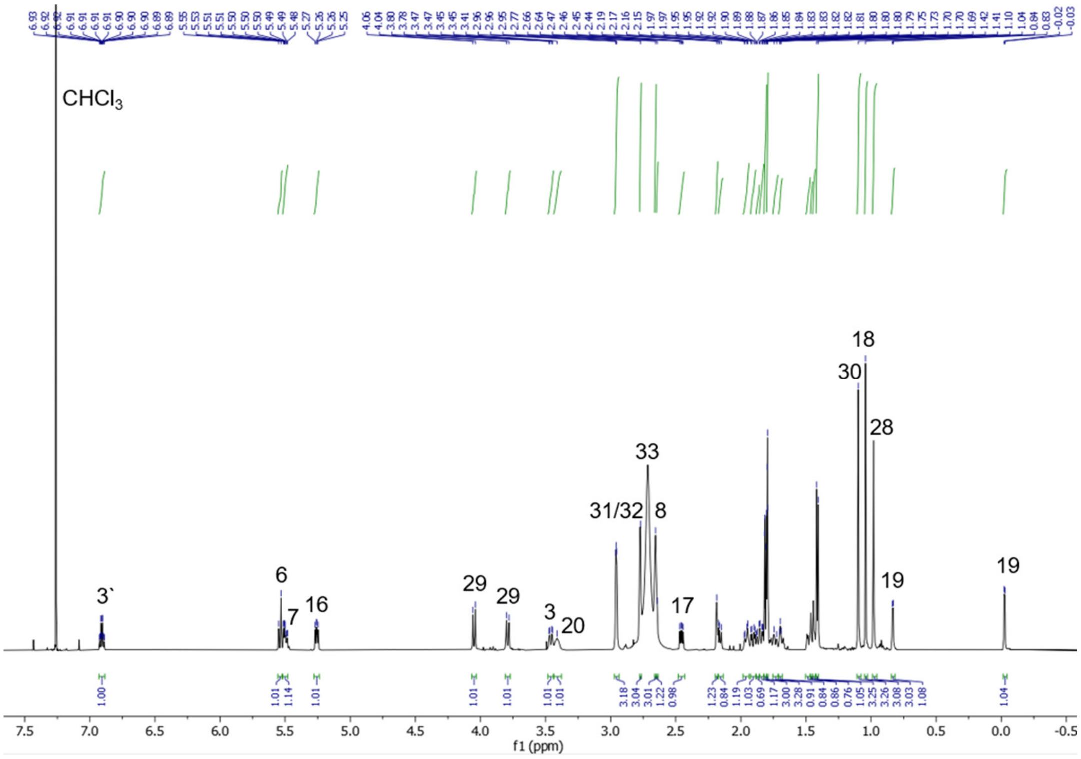

Figure S8. ^{1}H NMR spectrum of O-tigloylcyclomicrophylline-B (**3**) ($CDCl_3$, 600 MHz). The assignment of the signals between 1.35 and 2.25 ppm can be found in the enlarged Figure S9.

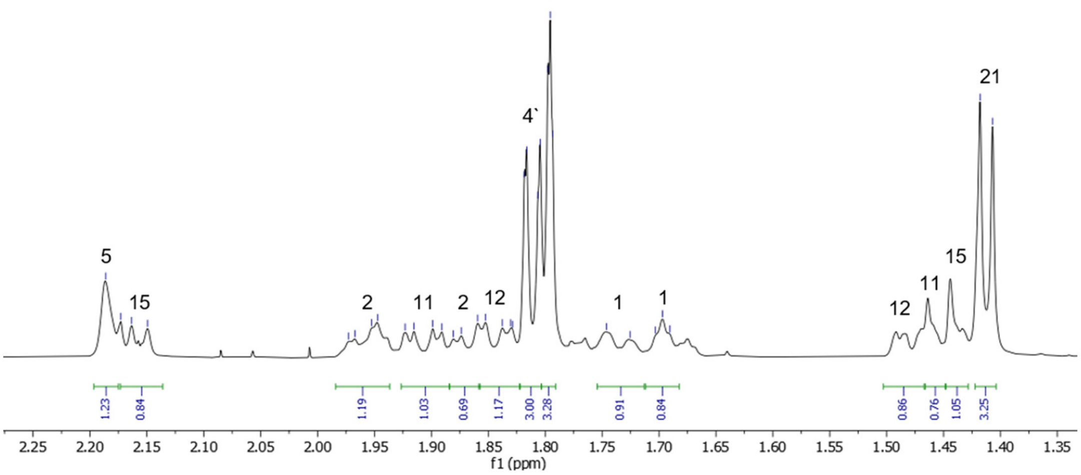

Figure S9. Detail of the ^{1}H NMR spectrum of O-tigloylcyclomicrophylline-B (**3**) ($CDCl_3$, 600 MHz).

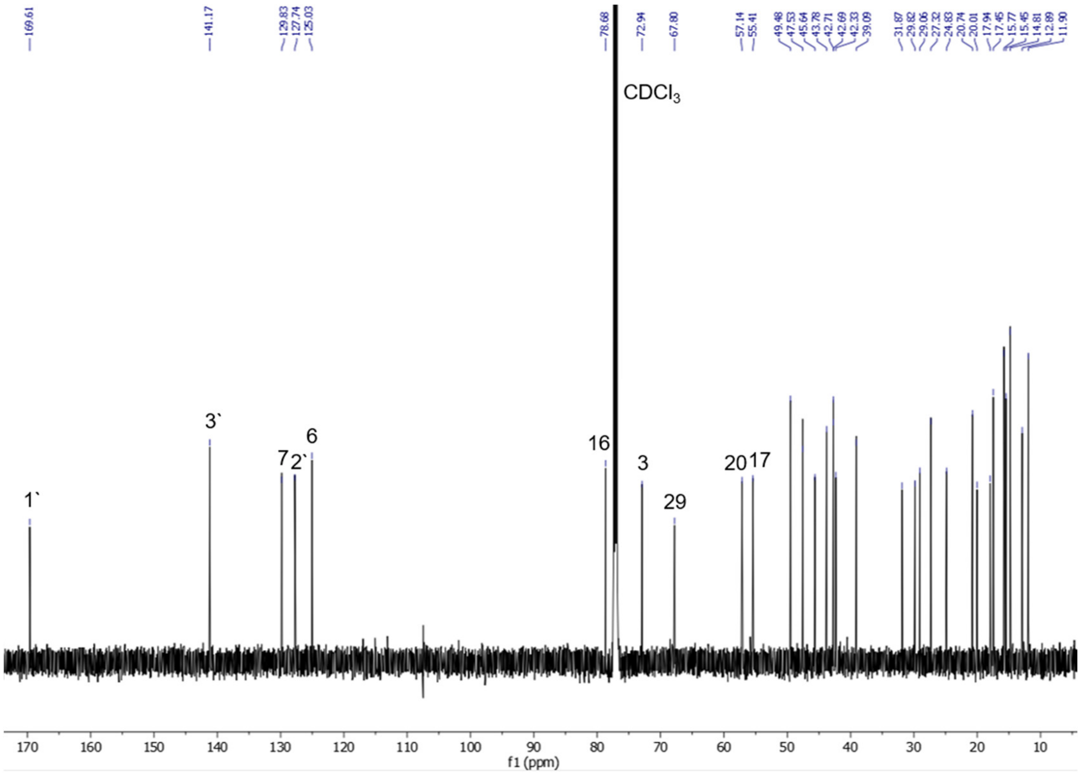

Figure S10. ^{13}C NMR spectrum of O-tigloylcyclomicrophylline-B (**3**) (CDCl$_3$, 150 MHz). The assignment of the signals between 10 and 50 ppm can be found in the enlarged Figure S11.

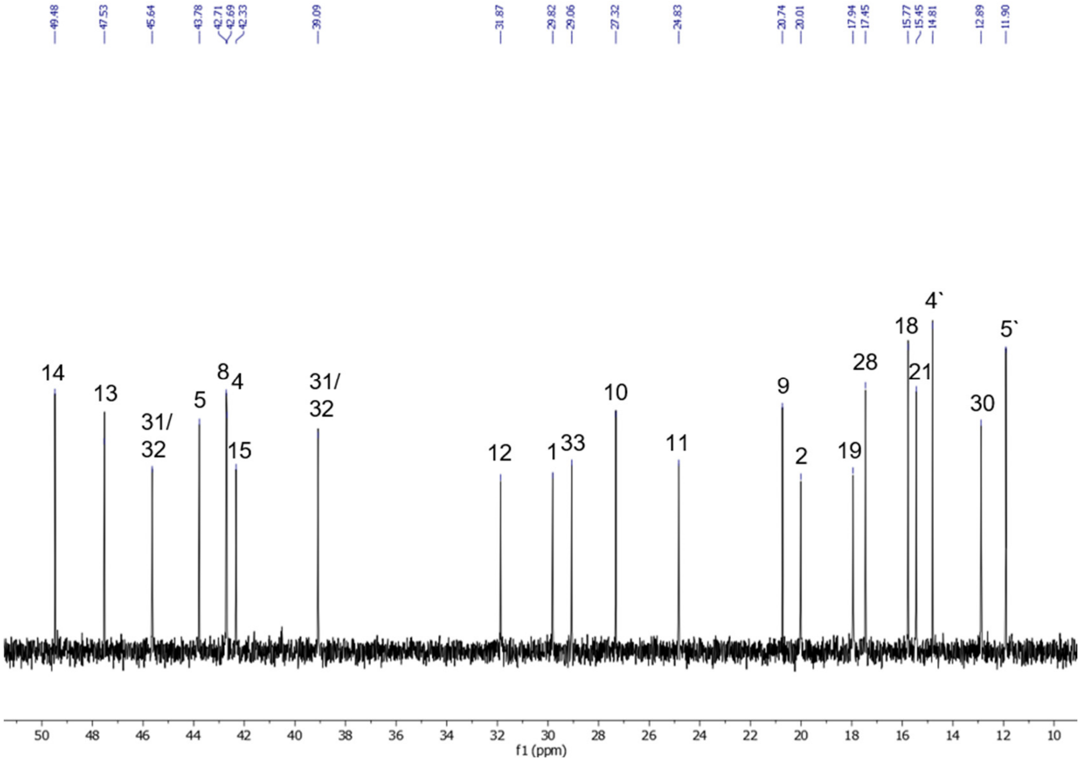

Figure S11. Detail of the ^{13}C NMR spectrum of O-tigloylcyclomicrophylline-B (**3**) (CDCl$_3$, 150 MHz).

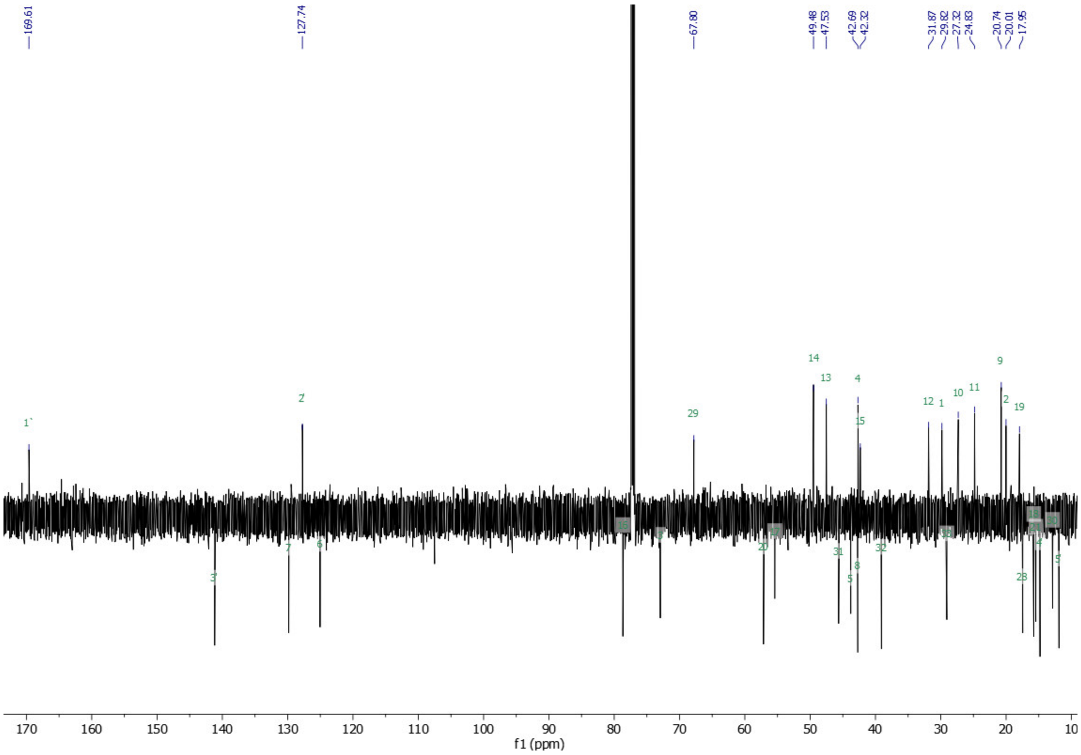

Figure S12. ^{13}C APT spectrum of O-tigloylcyclomicrophylline-B (**3**) ($CDCl_3$, 150 MHz).

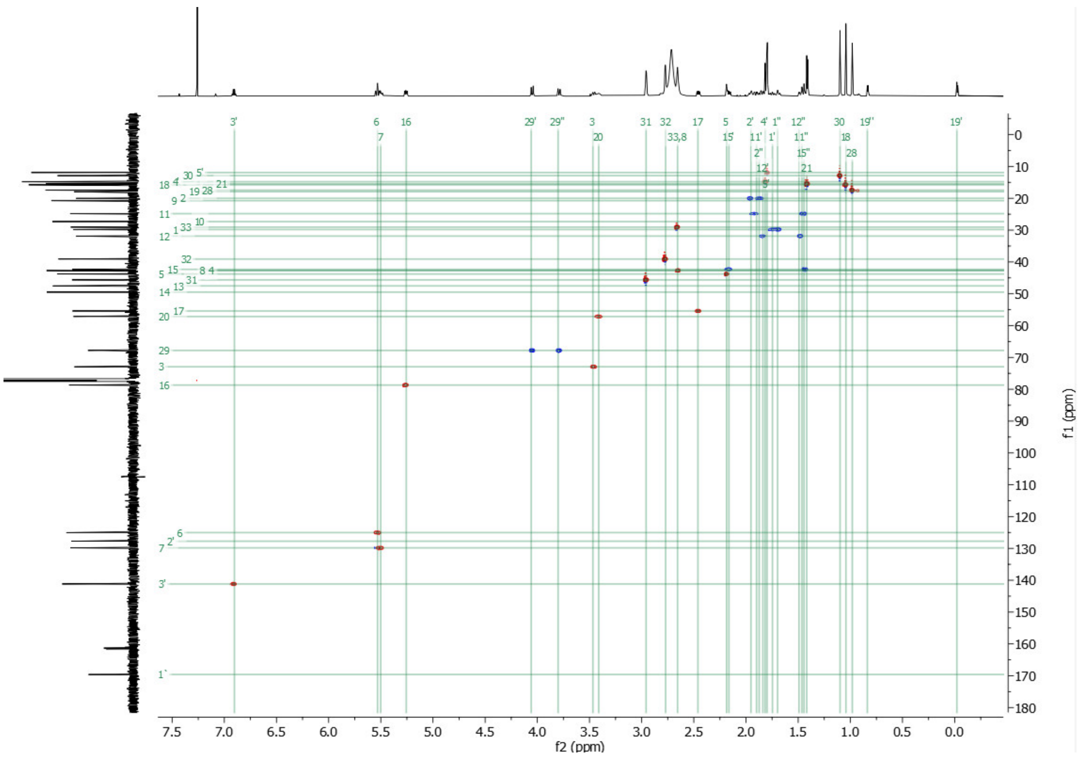

Figure S13. $^{1}H/^{13}C$ HSQC spectrum of O-tigloylcyclomicrophylline-B (**3**) ($CDCl_3$, 600/150 MHz).

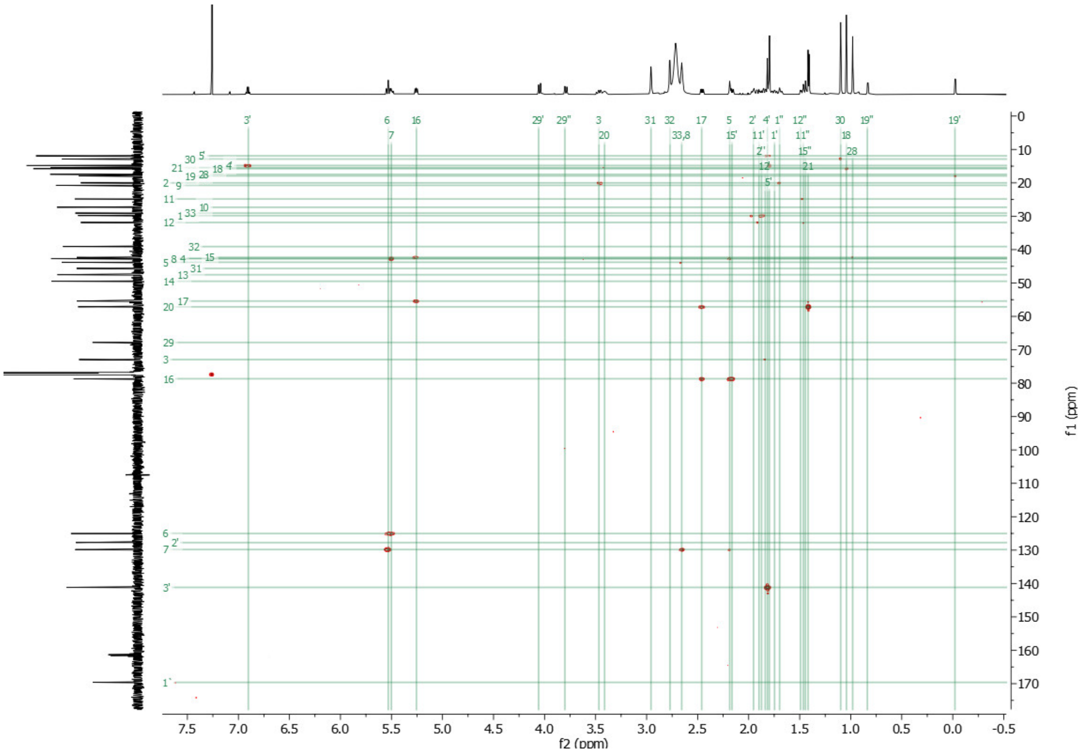

Figure S14. $^{1}H/^{13}C$ H2BC spectrum of O-tigloylcyclomicrophylline-B (**3**) ($CDCl_3$, 600/150 MHz).

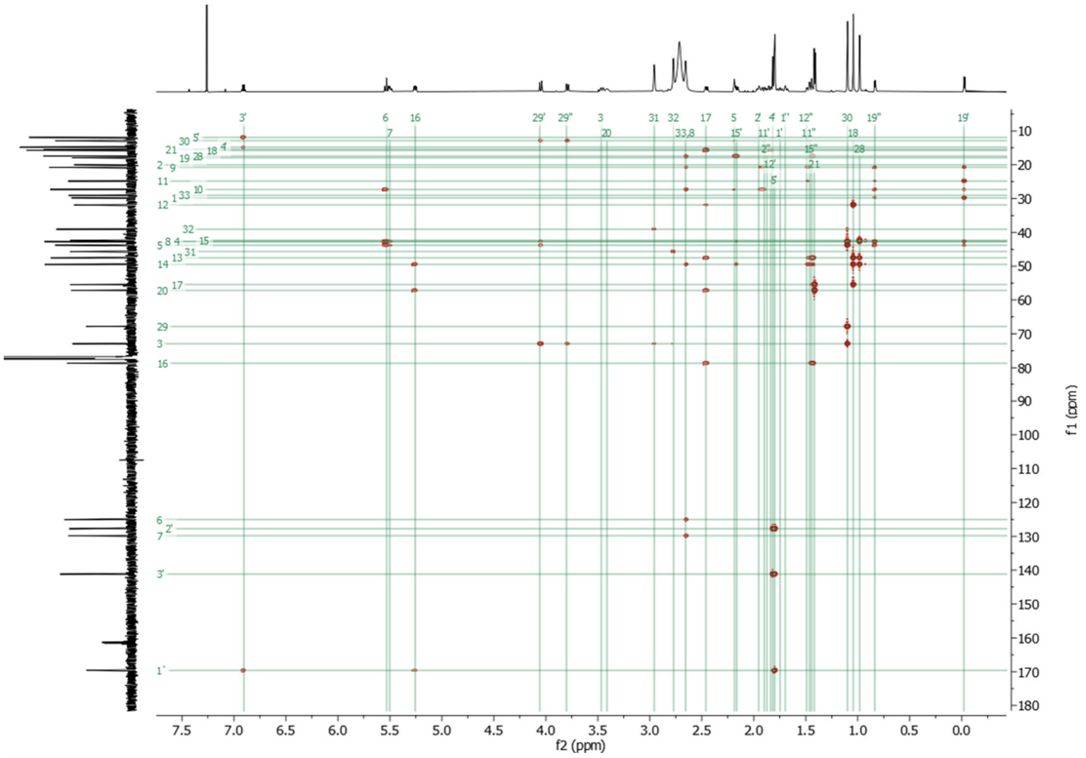

Figure S15. $^{1}H/^{13}C$ HMBC spectrum of O-tigloylcyclomicrophylline-B (**3**) ($CDCl_3$, 600/150 MHz).

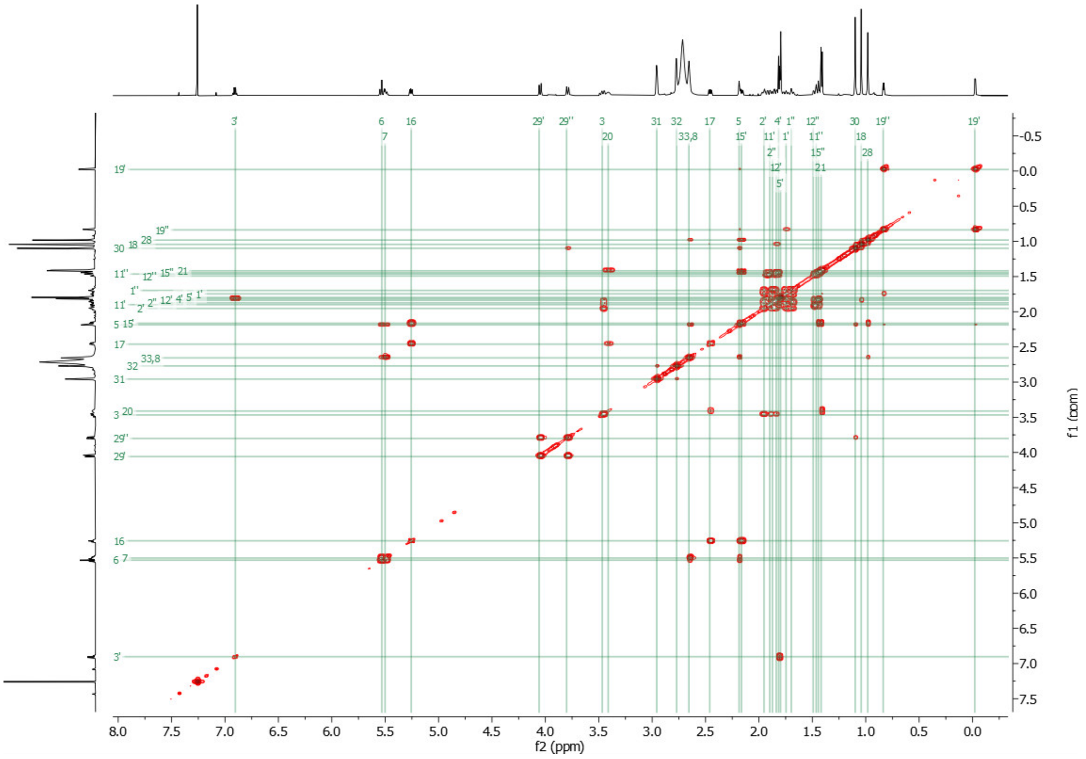

Figure S16. ^{1}H/^{1}H COSY spectrum of O-tigloylcyclomicrophylline-B (**3**) ($CDCl_3$, 600 MHz).

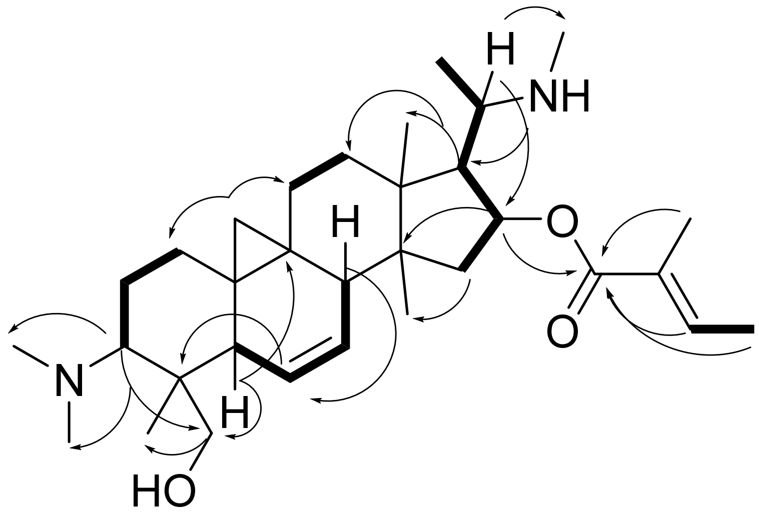

Figure S17. Key COSY (bold lines) and HMBC (arrows) correlations of O-tigloylcyclomicrophylline-B (**3**).

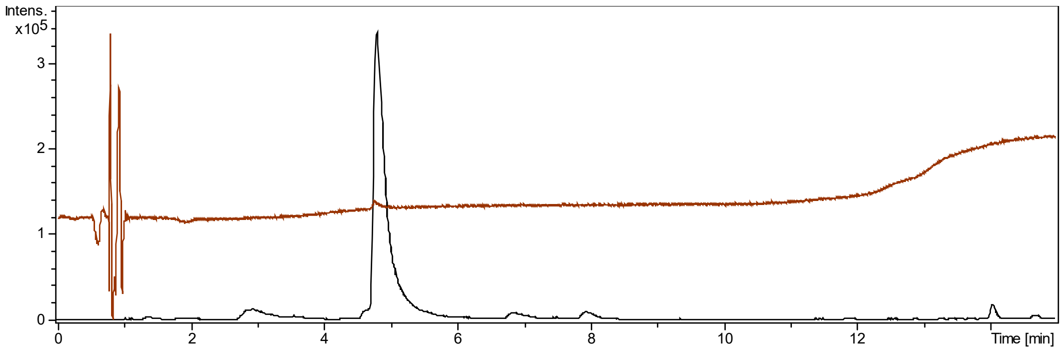

Figure S18. UHPLC/+ESI-QqTOF-MS/MS chromatogram of O-tigloylcyclomicrophylline-A (**4**). Base peak chromatogram 200.0000-1000.0000 +All MS (black); UV-Chromatogramm, 200-400 nm (red).

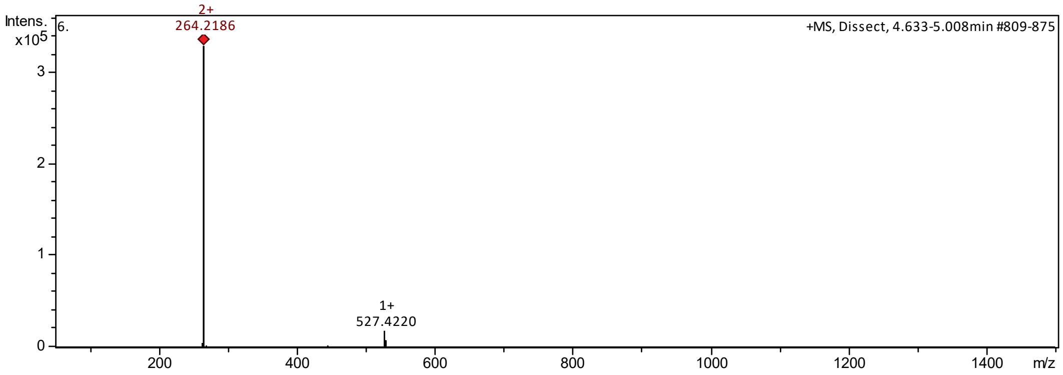

Figure S19. +ESI-QqTOF MS spectrum of O-tigloylcyclomicrophylline-A (**4**); *m/z* 264.2186 $[M+2H]^{2+}$ and 527.4220 $[M+H]^{+}$.

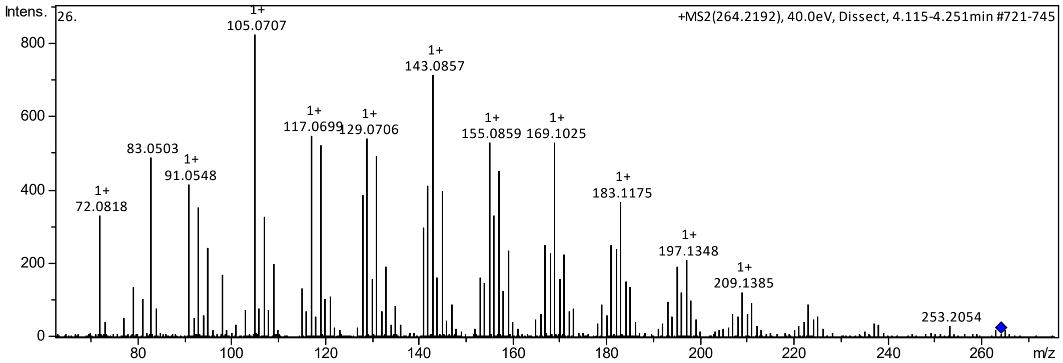

Figure S20. +ESI-QqTOF MS/MS spectrum of O-tigloylcyclomicrophylline-A (**4**).

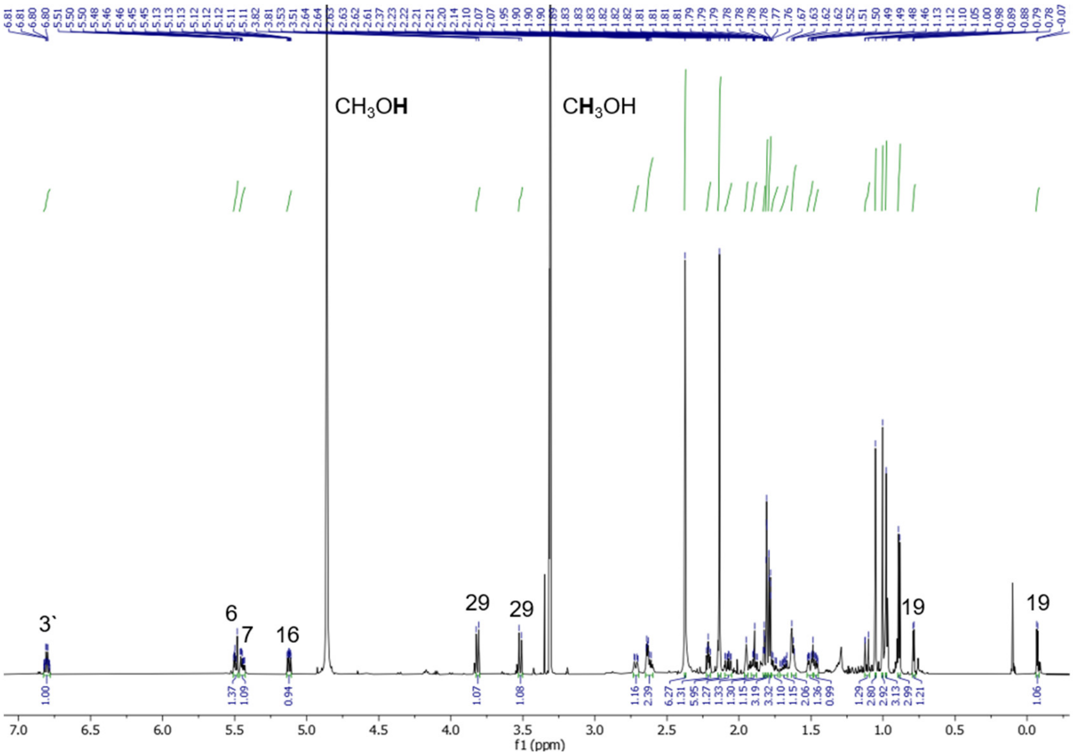

Figure S21. ^{1}H NMR spectrum of O-tigloylcyclomicrophylline-A (**4**) (CD_3OD, 600 MHz). The assignment of the signals between 0.85 and 2.8 ppm can be found in the enlarged Figure S22.

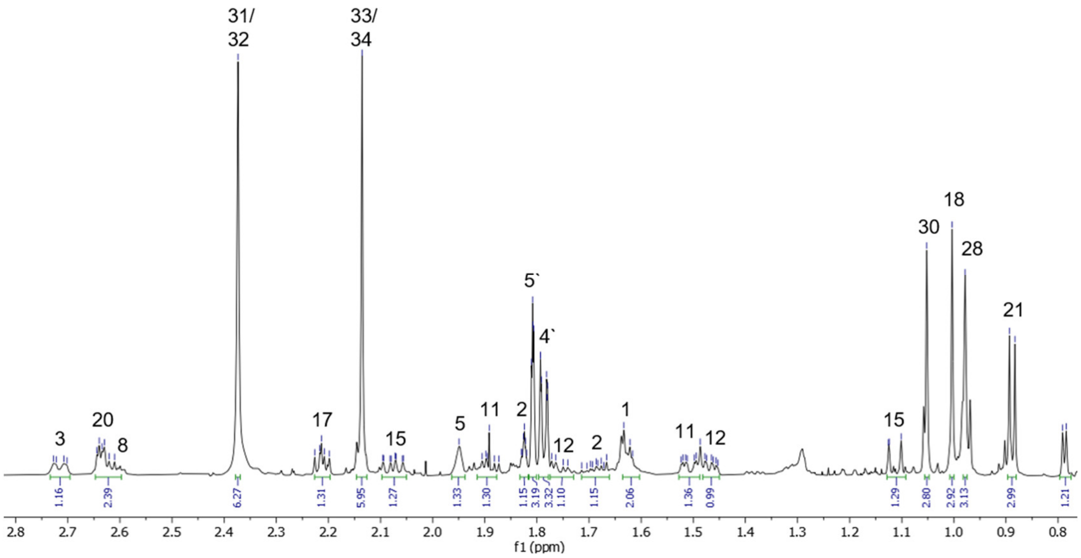

Figure S22. Detail of the ^{1}H NMR spectrum of O-tigloylcyclomicrophylline-A (**4**) (CD_3OD, 600 MHz).

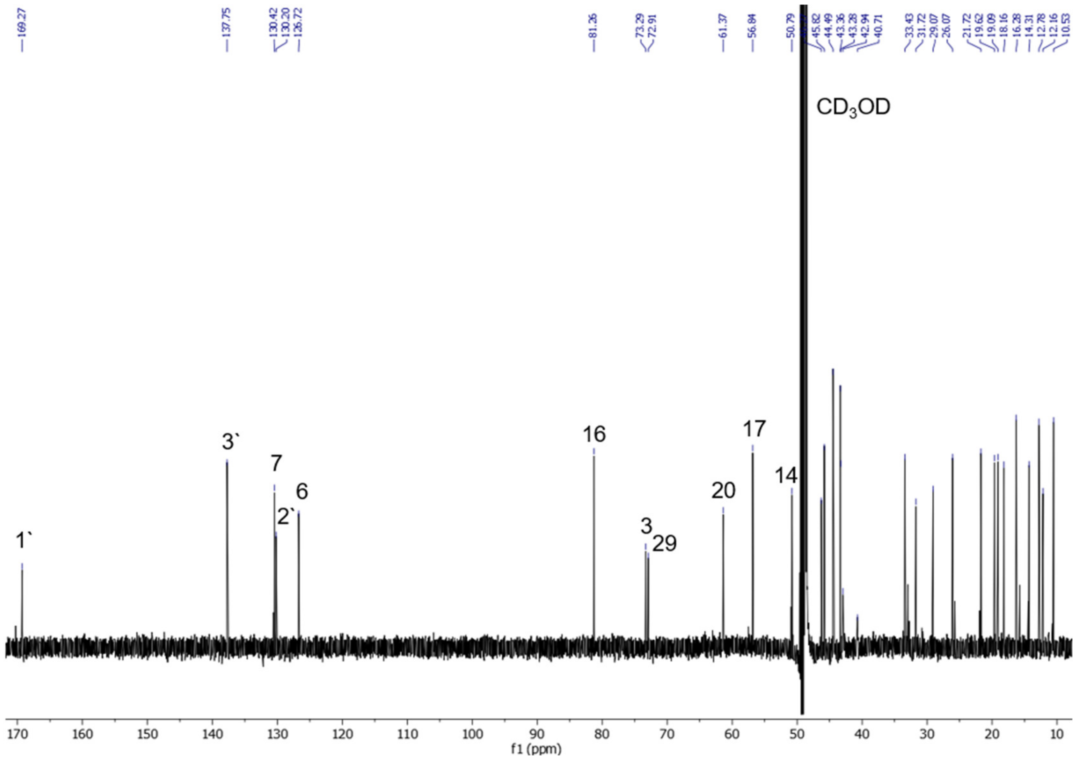

Figure S23. ^{13}C NMR spectrum of O-tigloylcyclomicrophylline-A (**4**) (CD_3OD, 150 MHz). The assignment of the signals between 10 and 47 ppm can be found in the enlarged Figure S24.

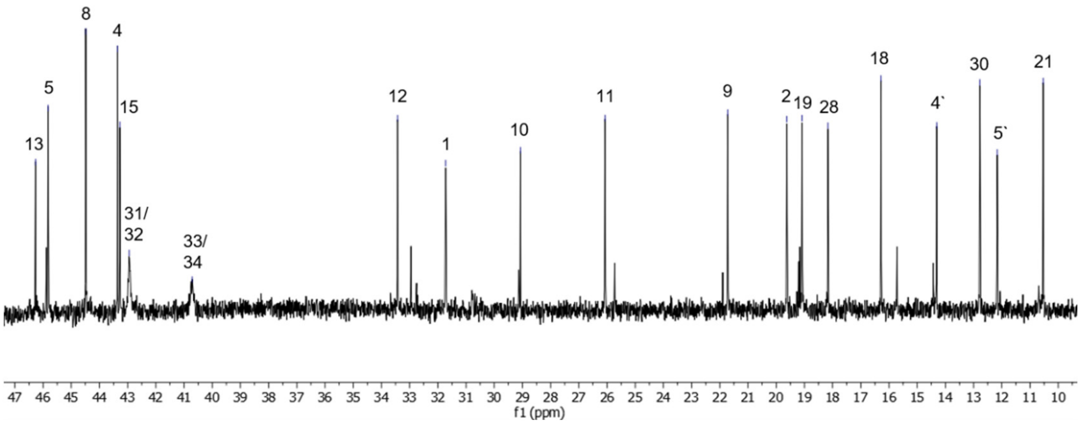

Figure S24. Detail of the ^{13}C NMR spectrum of O-tigloylcyclomicrophylline-A (**4**) (CD_3OD, 150 MHz).

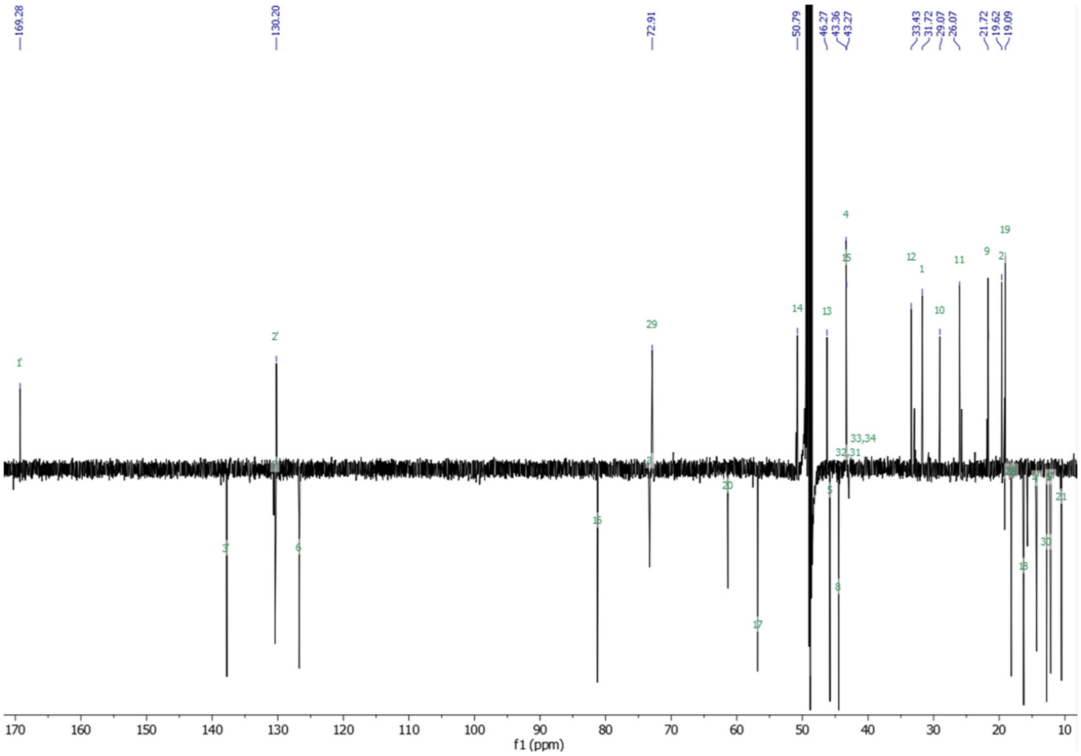

Figure S25. ^{13}C APT spectrum of O-tigloylcyclomicrophylline-A (**4**) (CD_3OD, 150 MHz).

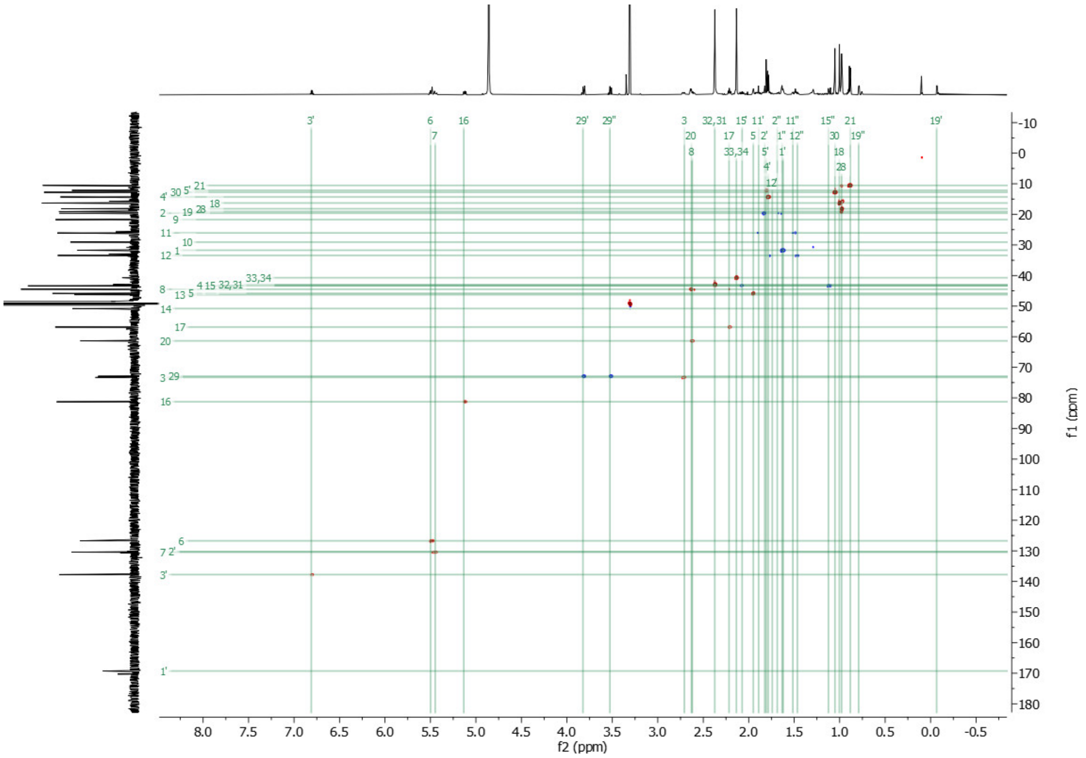

Figure S26. $^{1}H/^{13}C$ HSQC spectrum of O-tigloylcyclomicrophylline-A (**4**) (CD_3OD, 600/150 MHz).

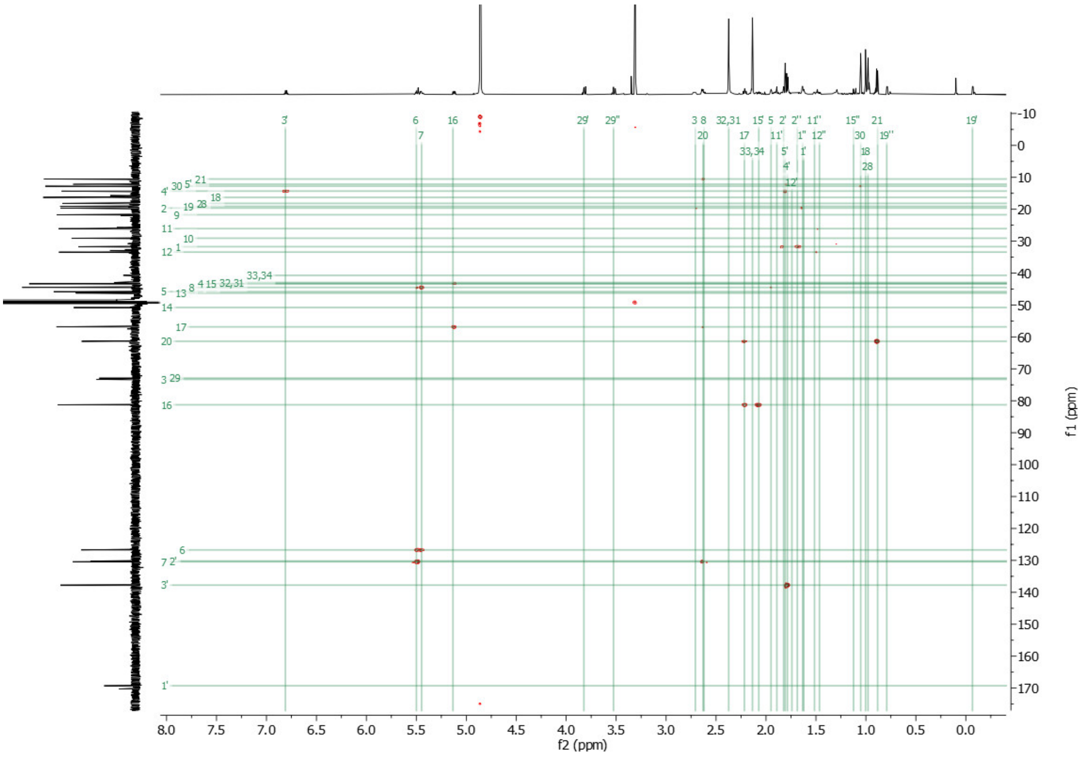

Figure S27. ^{1}H/^{13}C H2BC spectrum of O-tigloylcyclomicrophylline-A (**4**) (CD_3OD, 600/150 MHz).

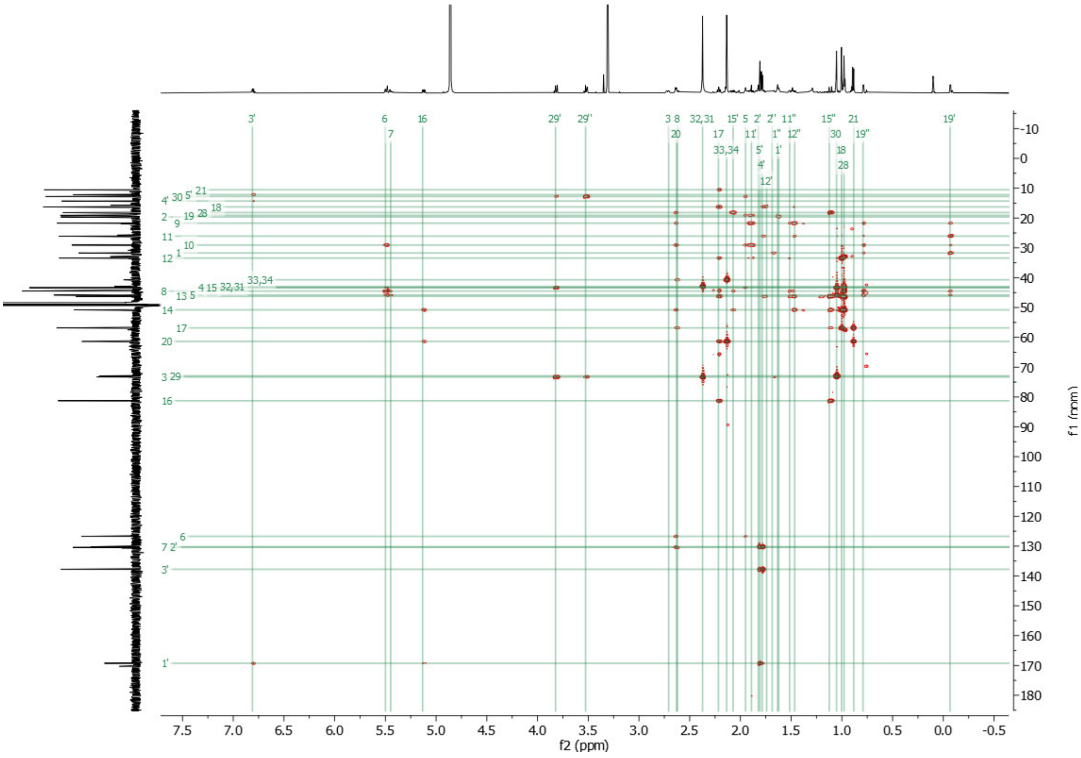

Figure S28. ^{1}H/^{13}C HMBC spectrum of O-tigloylcyclomicrophylline-A (**4**) (CD_3OD, 600/150 MHz).

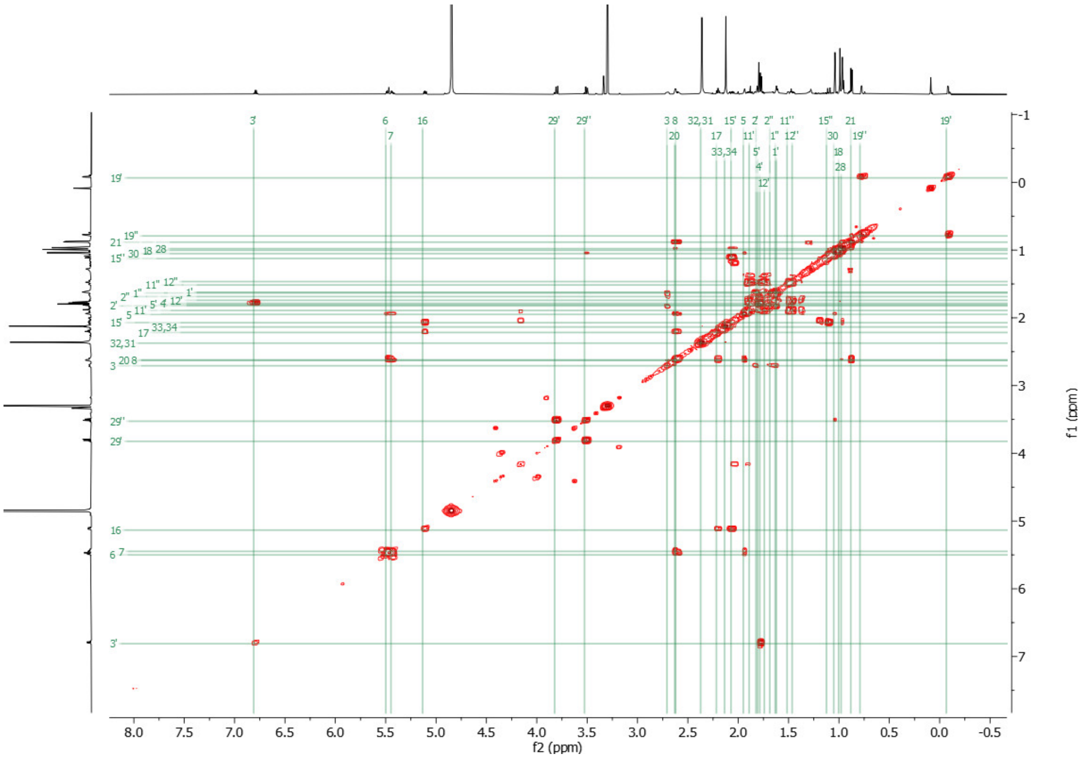

Figure S29. $^1H/^1H$ COSY spectrum of O-tigloylcyclomicrophylline-A (**4**) (CD_3OD, 600 MHz).

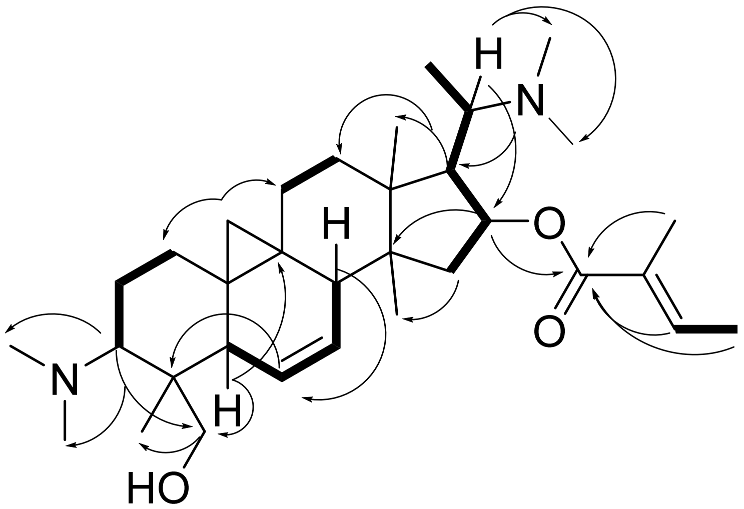

Figure S30. Key COSY (bold lines) and HMBC (arrows) correlations of O-tigloylcyclomicrophylline-A (**4**).

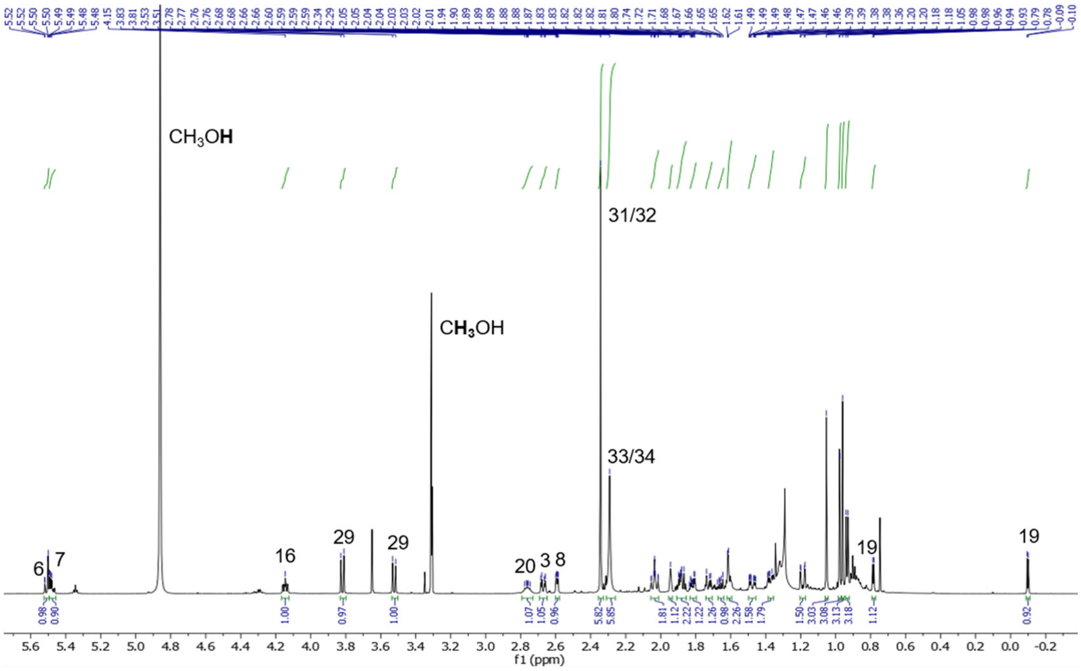

Figure S31. ^{1}H NMR spectrum of Cyclomicrophylline-A (**5**) (CD_3OD, 600 MHz). The assignment of the signals between 0.9 and 2.05 ppm can be found in the enlarged Figure S32.

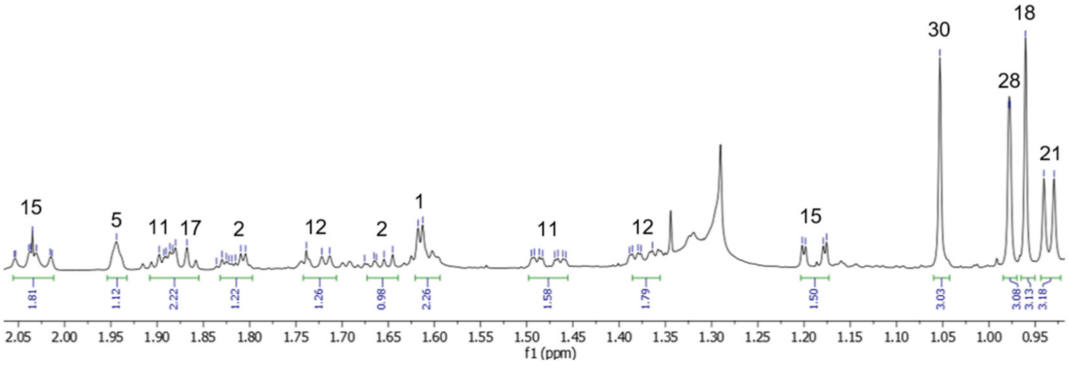

Figure S32. Detail of the ^{1}H NMR spectrum of Cyclomicrophylline-A (**5**) (CD_3OD, 600 MHz).

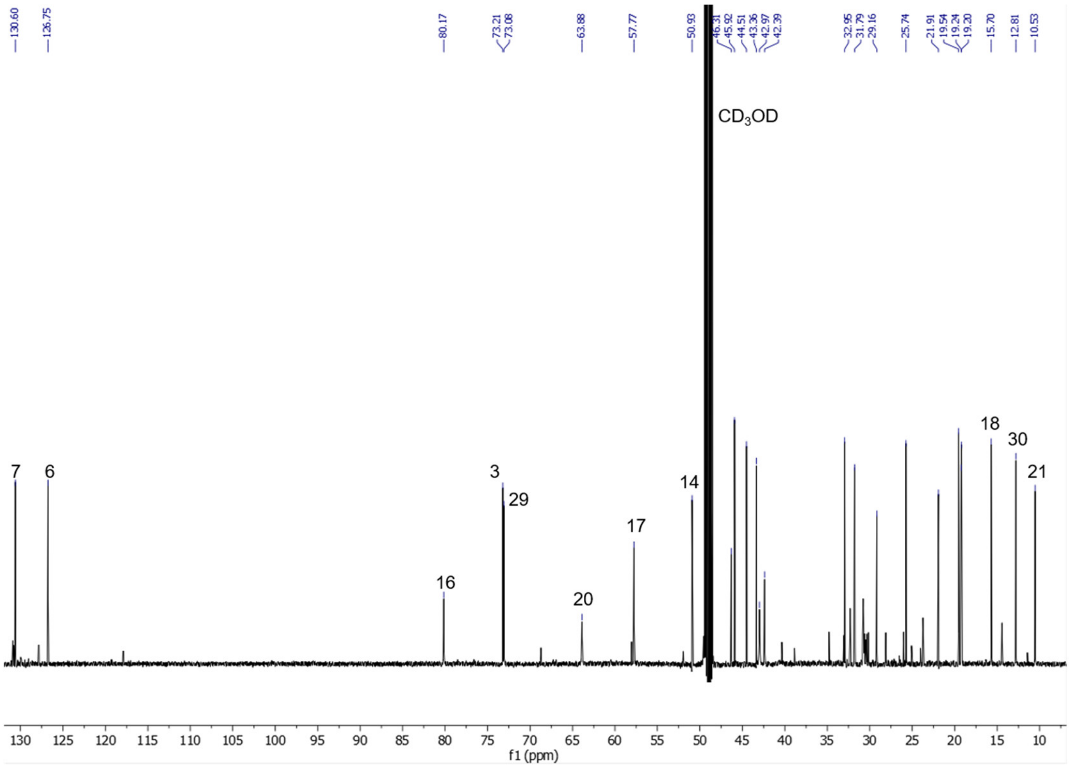

Figure S33. ^{13}C NMR spectrum of Cyclomicrophylline-A (**5**) (CD_3OD, 150 MHz). The assignment of the signals between 19 and 46.5 ppm can be found in the enlarged Figure S34.

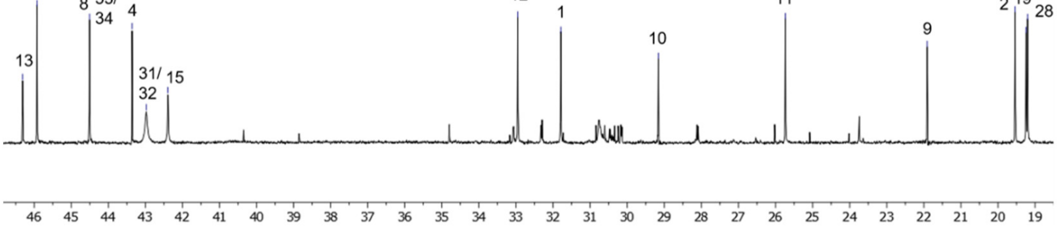

Figure S34. Detail of the ^{13}C NMR spectrum of Cyclomicrophylline-A (**5**) (CD_3OD, 150 MHz).

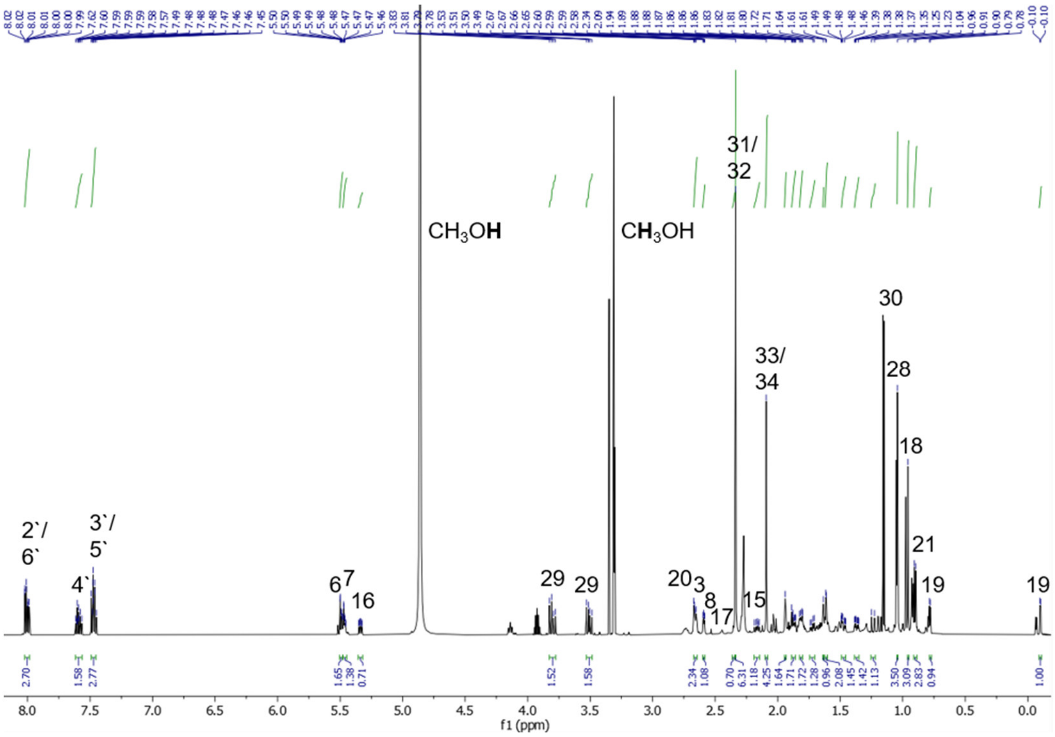

Figure S35. ^{1}H NMR spectrum of Cyclomicrophyllidine-A (**6**) (CD_3OD, 600 MHz). The assignment of the signals between 1.2 and 2.0 ppm can be found in the enlarged Figure S36.

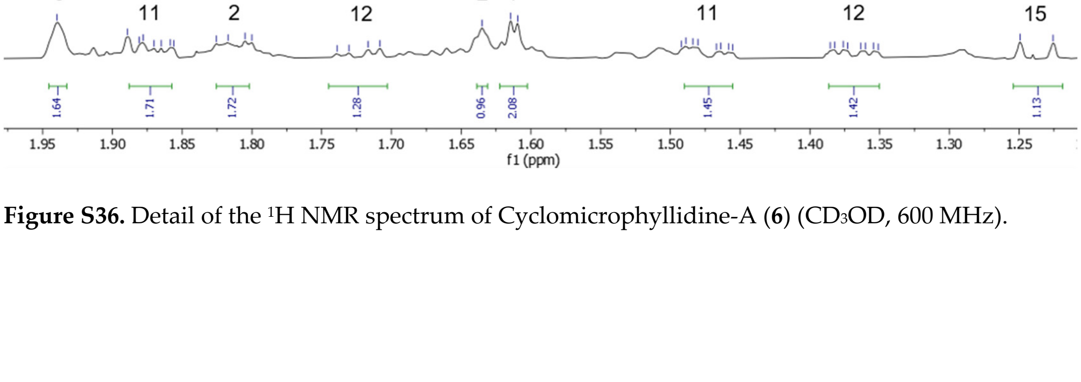

Figure S36. Detail of the ^{1}H NMR spectrum of Cyclomicrophyllidine-A (**6**) (CD_3OD, 600 MHz).

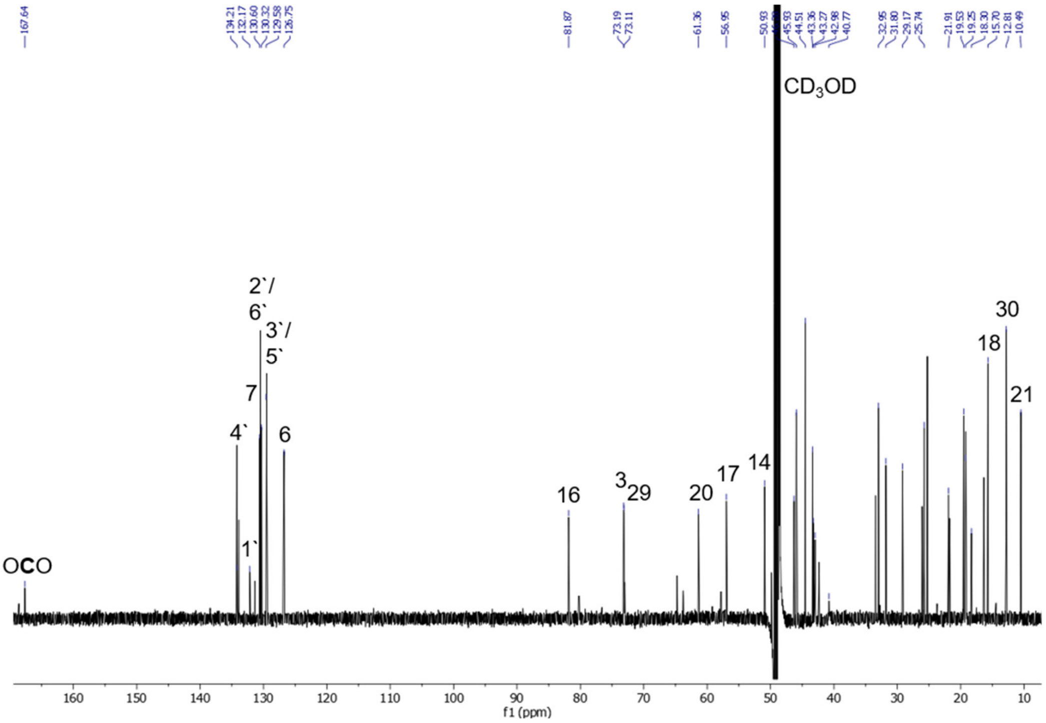

Figure S37. ^{13}C NMR spectrum of Cyclomicrophyllidine-A (**6**) (CD_3OD, 150 MHz). The assignment of the signals between 18 and 47 ppm can be found in the enlarged Figure S38.

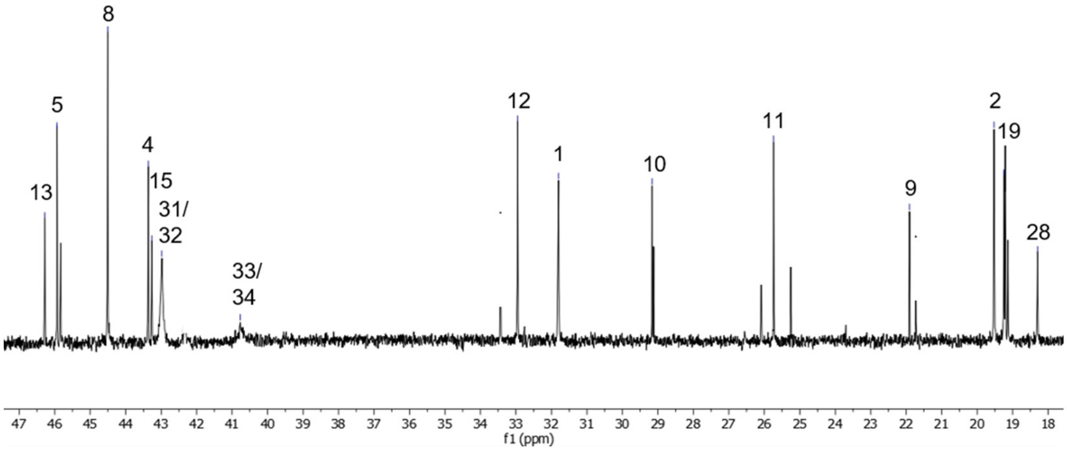

Figure S38. Detail of the ^{13}C NMR spectrum of Cyclomicrophyllidine-A (**6**) (CD_3OD, 150 MHz).

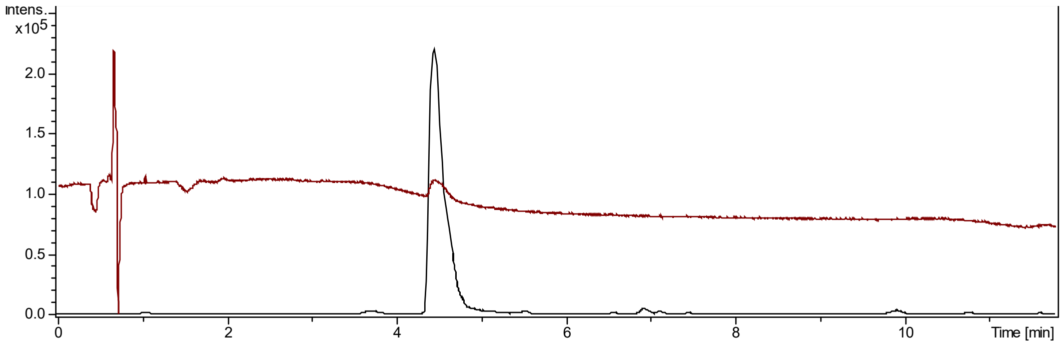

Figure S39. UHPLC/+ESI-QqTOF-MS/MS chromatogram of Cyclomicrophyllidine-B (**7**). Base peak chromatogram 200.0000-1000.0000 +All MS (black); UV-Chromatogramm, 200-400 nm (red).

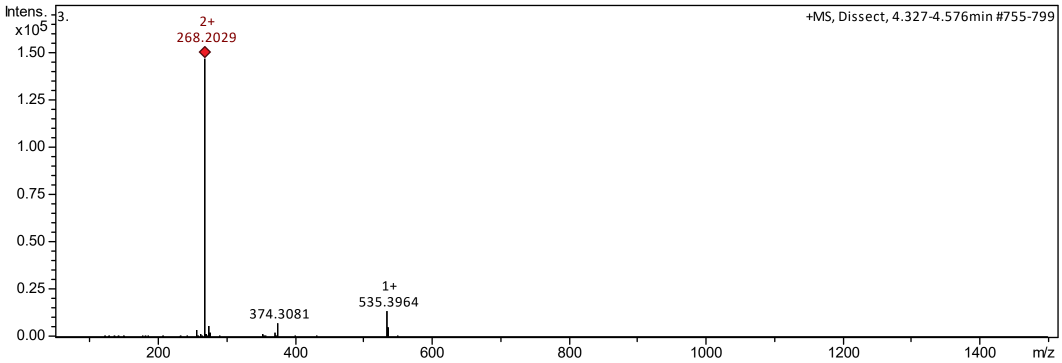

Figure S40. +ESI-QqTOF MS spectrum of Cyclomicrophyllidine-B (**7**); *m/z* 268.2029 $[M+2H]^{2+}$ and 535.3964 $[M+H]^{+}$.

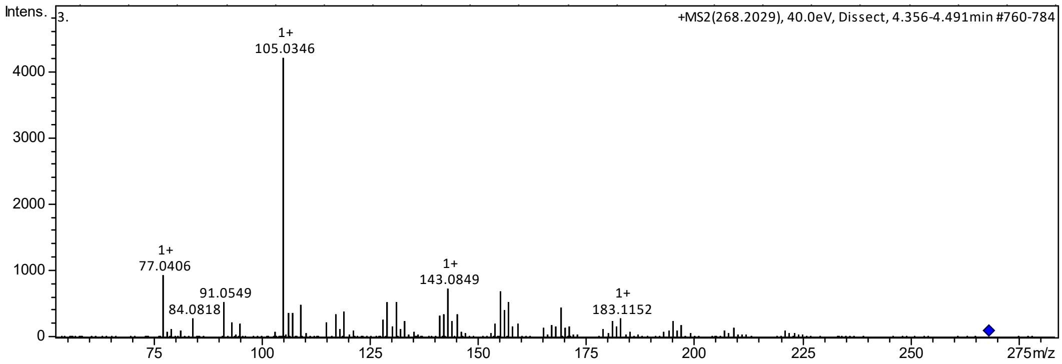

Figure S41. +ESI-QqTOF MS/MS spectrum of Cyclomicrophyllidine-B (**7**).

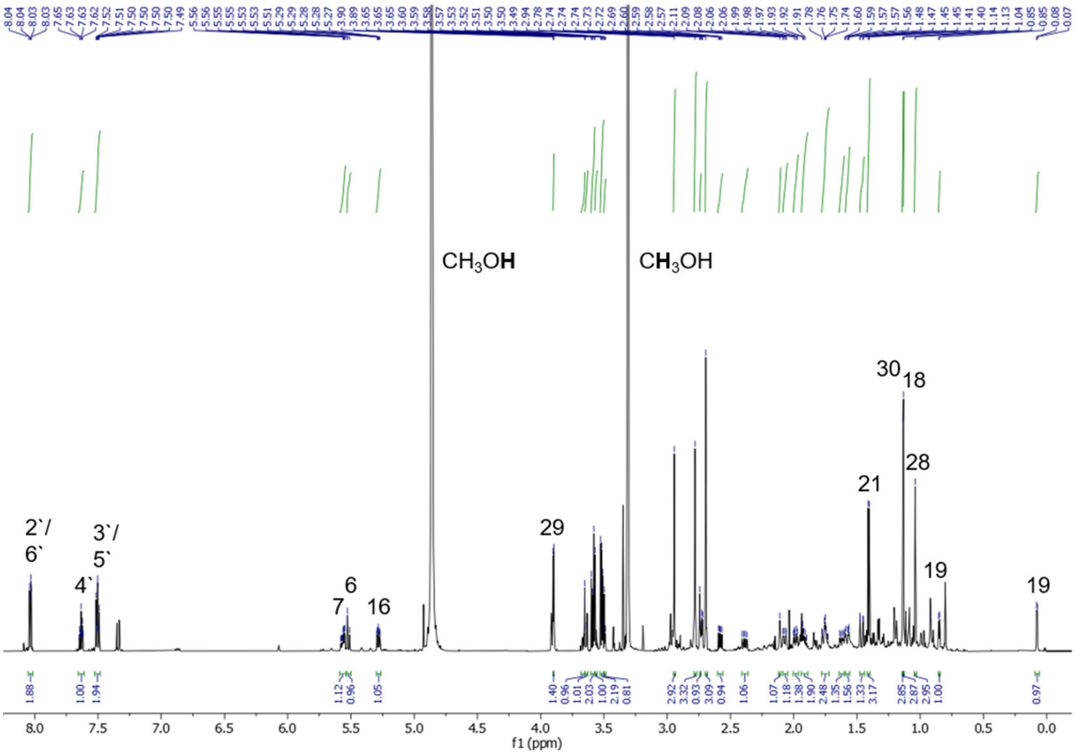

Figure S42. ^{1}H NMR spectrum of Cyclomicrophyllidine-B (**7**) (CD_3OD, 600 MHz). The assignment of the signals between 1.45 and 3.65 ppm can be found in the enlarged Figure S43.

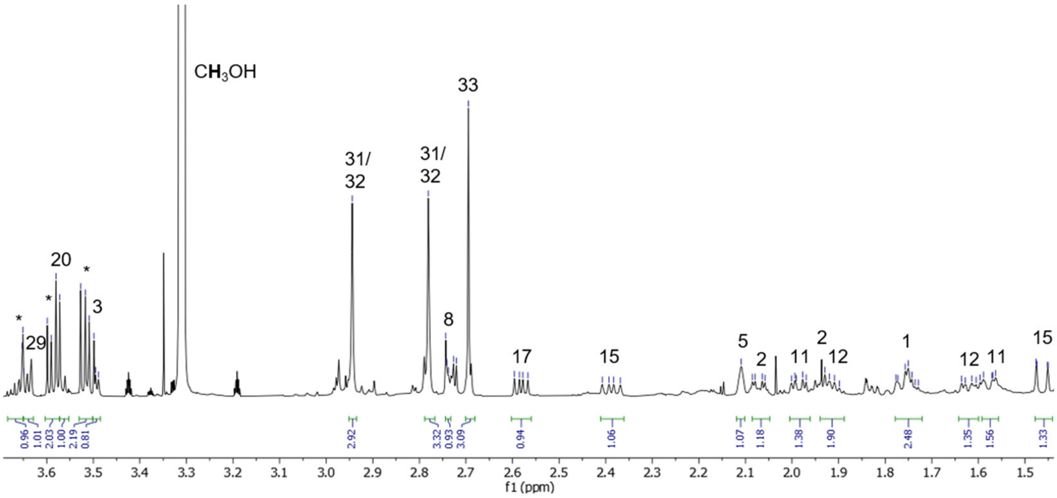

Figure S43. Detail of the ^{1}H NMR spectrum of Cyclomicrophyllidine-B (**7**) (CD_3OD, 600 MHz) (*signals of 9.4% glycerol).

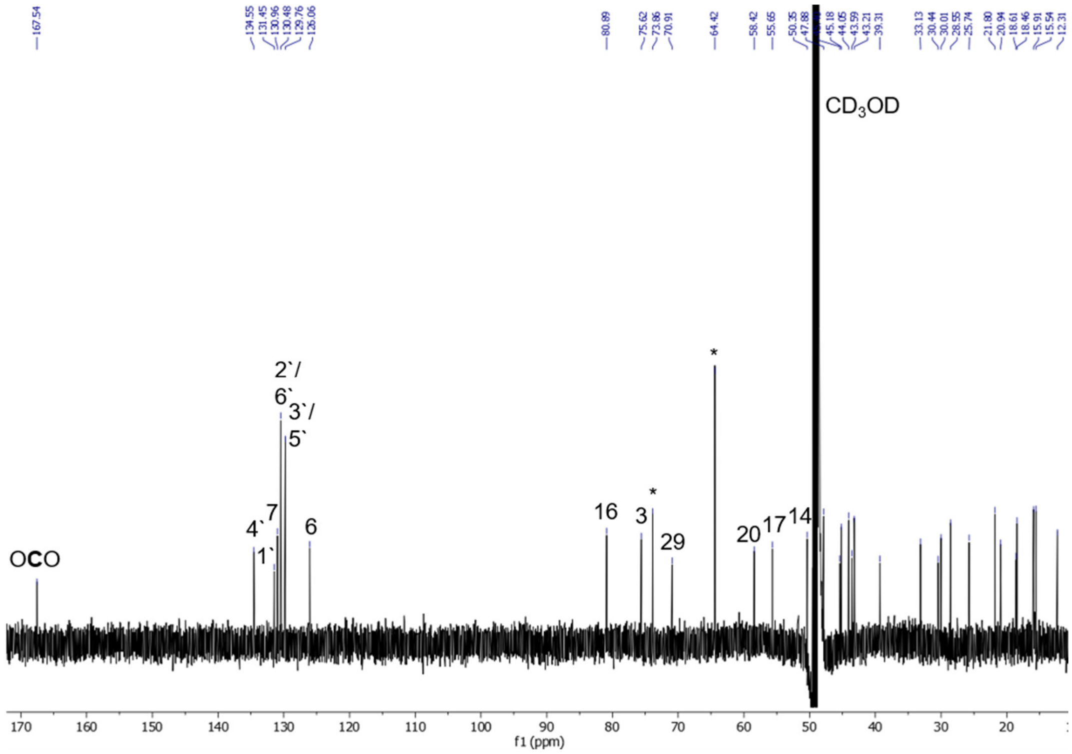

Figure S44. ^{13}C NMR spectrum of Cyclomicrophyllidine-B (**7**) (CD_3OD, 150 MHz). The assignment of the signals between 11 and 48 ppm can be found in the enlarged Figure S45 (*signals of 9.4% glycerol).

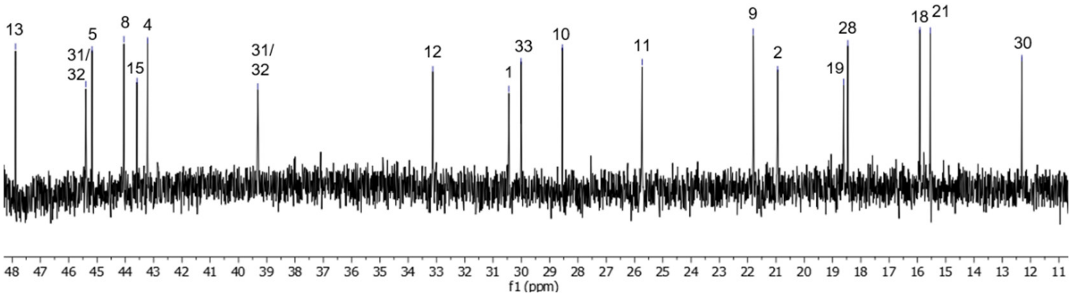

Figure S45. Detail of the ^{13}C NMR spectrum of Cyclomicrophyllidine-B (**7**) (CD_3OD, 150 MHz).

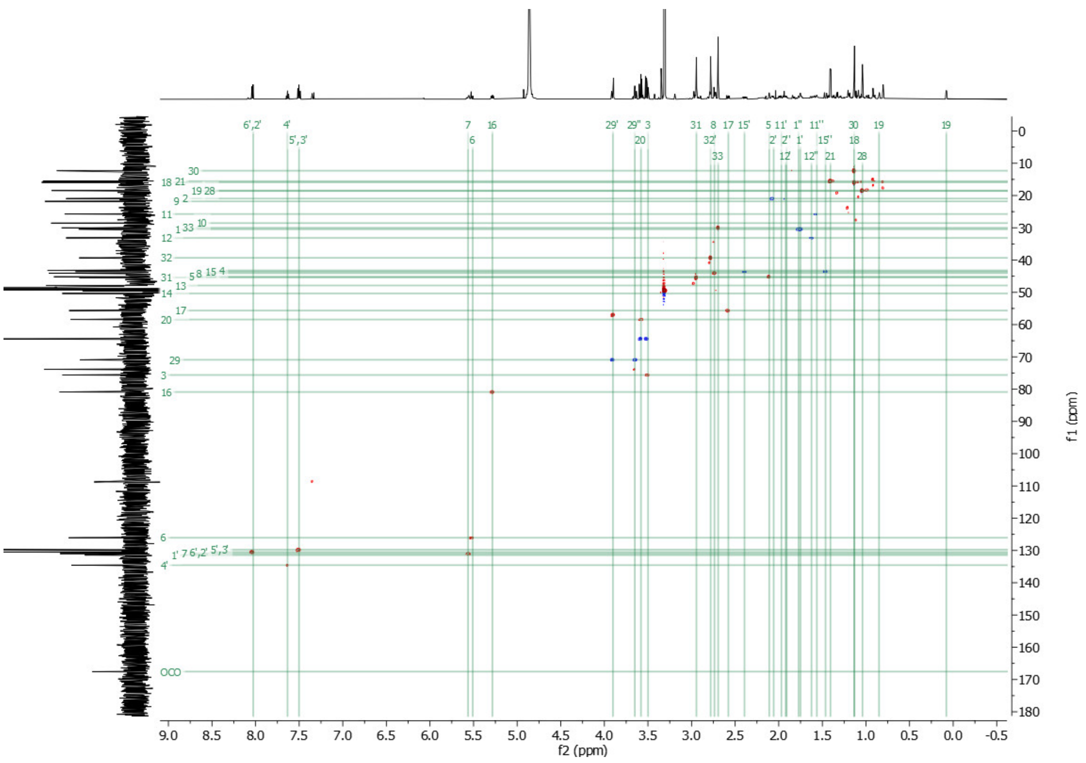

Figure S46. $^{1}H/^{13}C$ HSQC spectrum of Cyclomicrophyllidine-B (**7**) (CD_3OD, 600/150 MHz).

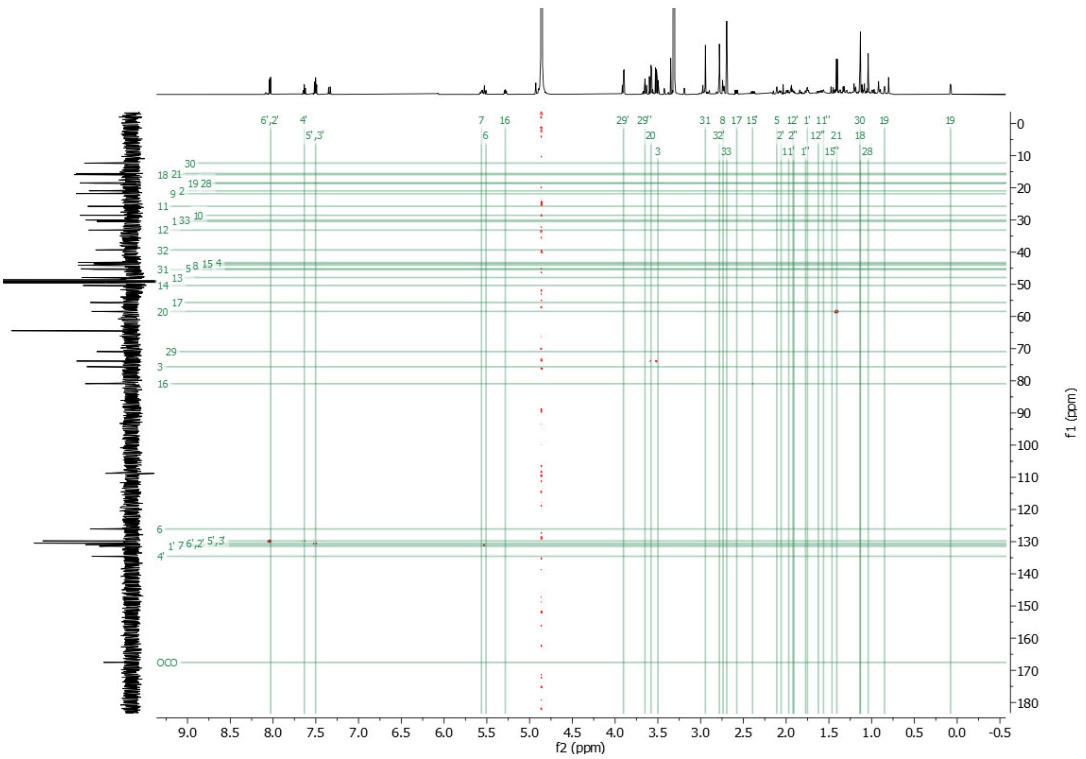

Figure S47. $^{1}H/^{13}C$ H2BC spectrum of Cyclomicrophyllidine-B (**7**) (CD_3OD, 600/150 MHz).

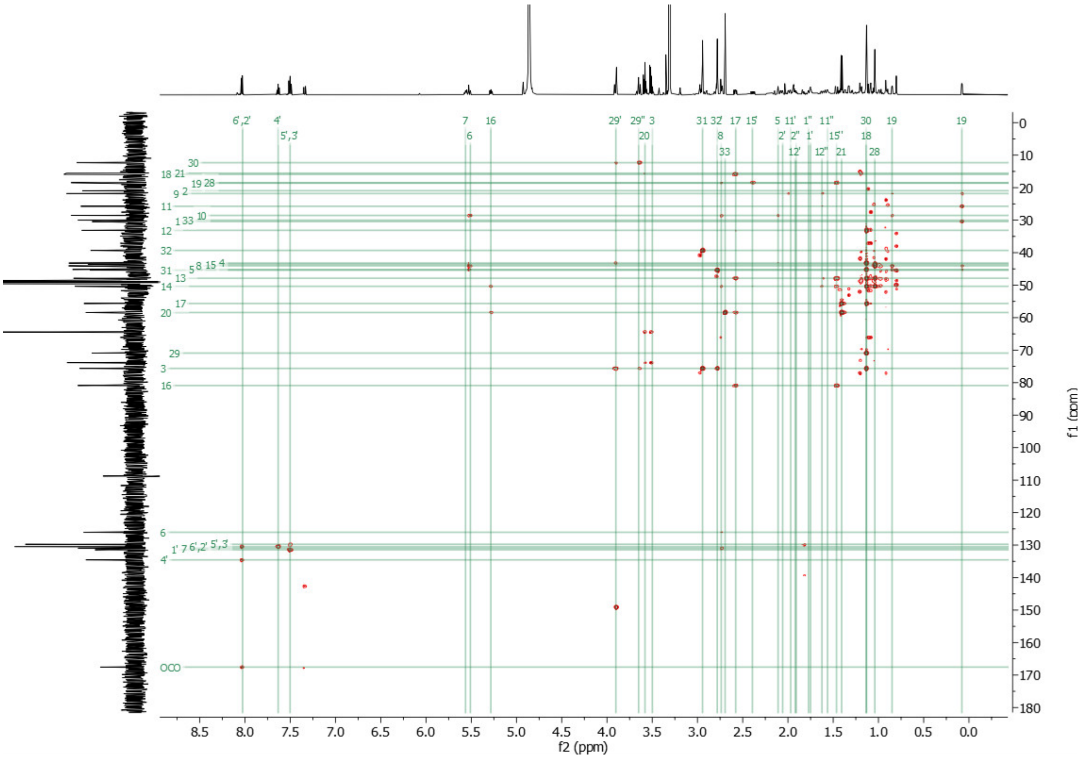

Figure S48. ^{1}H/^{13}C HMBC spectrum of Cyclomicrophyllidine-B (**7**) (CD_3OD, 600/150 MHz).

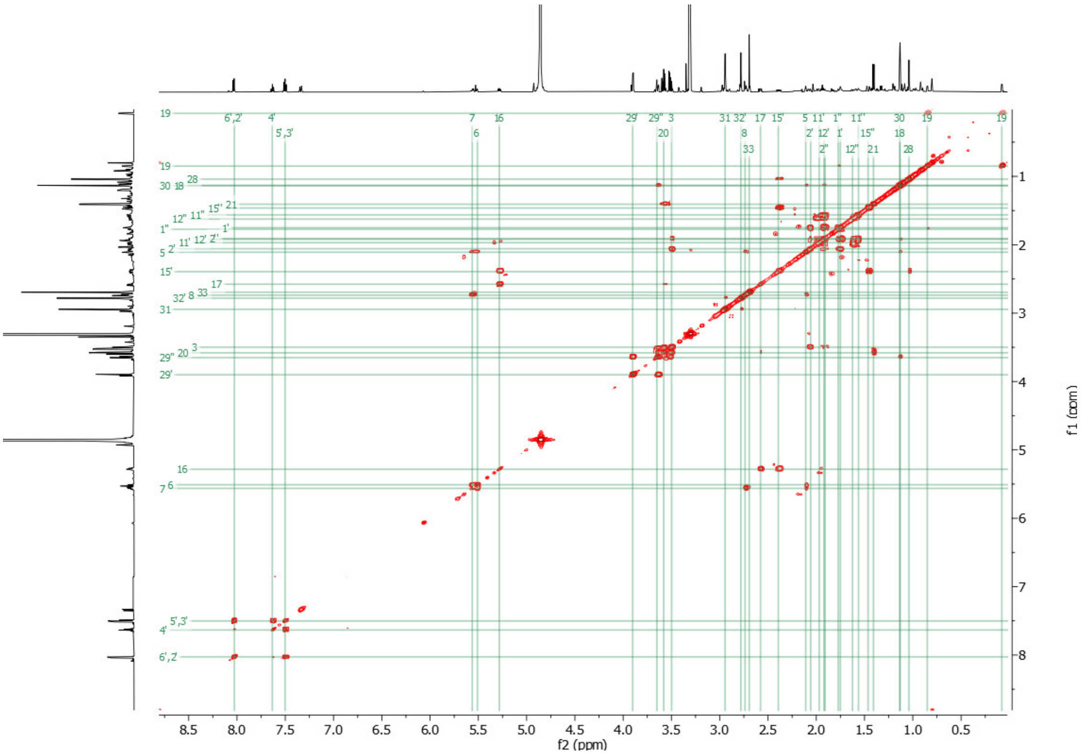

Figure S49. ^{1}H/^{1}H COSY spectrum of Cyclomicrophyllidine-B (**7**) (CD_3OD, 600 MHz).

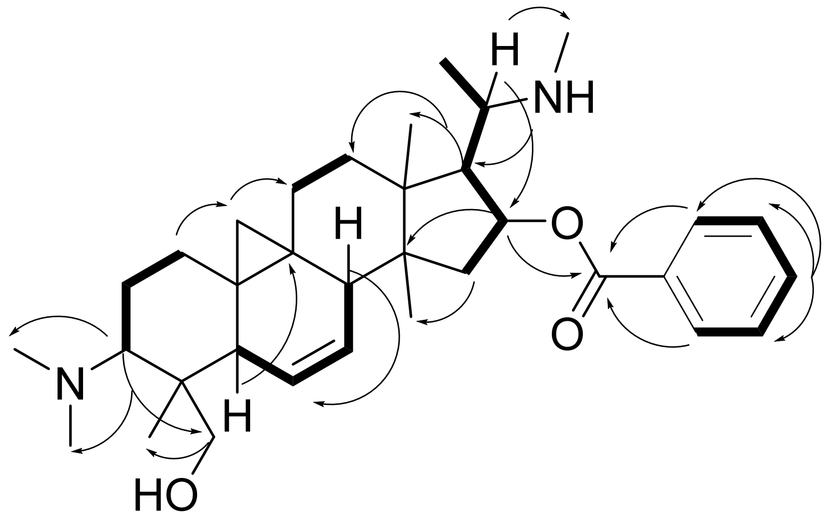

Figure S50. Key COSY (bold lines) and HMBC (arrows) correlations of Cyclomicrophyllidine-B (**7**).

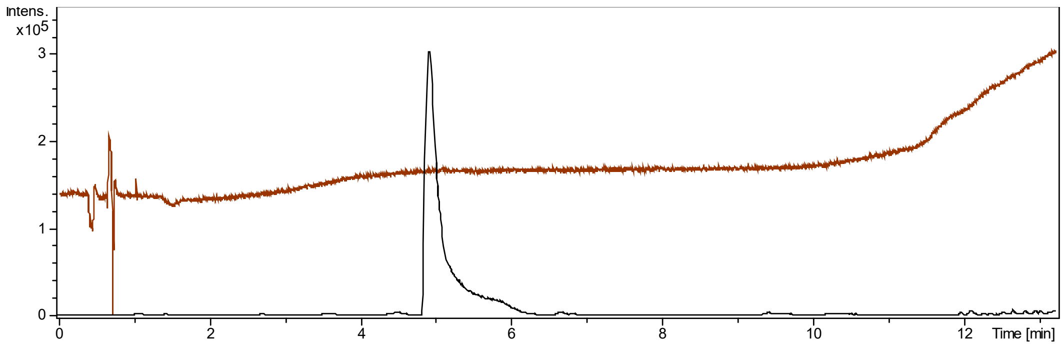

Figure S51. UHPLC/+ESI-QqTOF-MS/MS chromatogram of O-benzoyl-cycloprotobuxoline-D (**8**). Base peak chromatogram 200.0000-1000.0000 +All MS (black); UV-Chromatogramm, 200-400 nm (red).

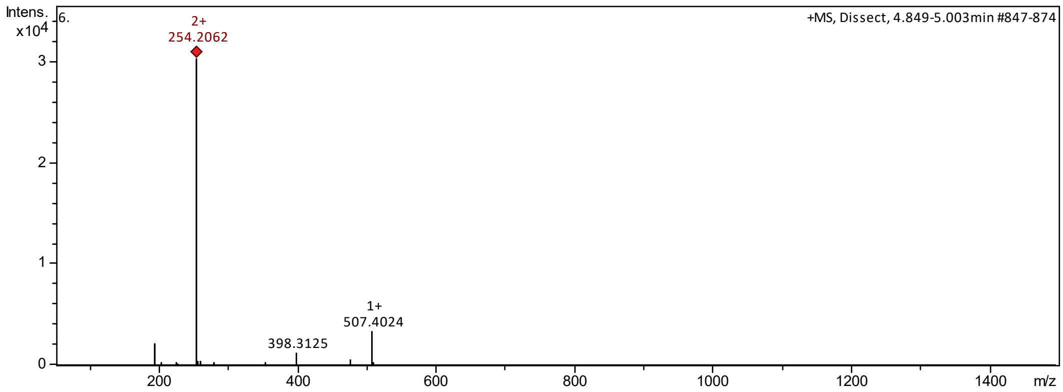

Figure S52. +ESI-QqTOF MS spectrum of O-benzoyl-cycloprotobuxoline-D (**8**); *m*/*z* 254.2062 $[M+2H]^{2+}$ and 507.4042 $[M+H]^{+}$.

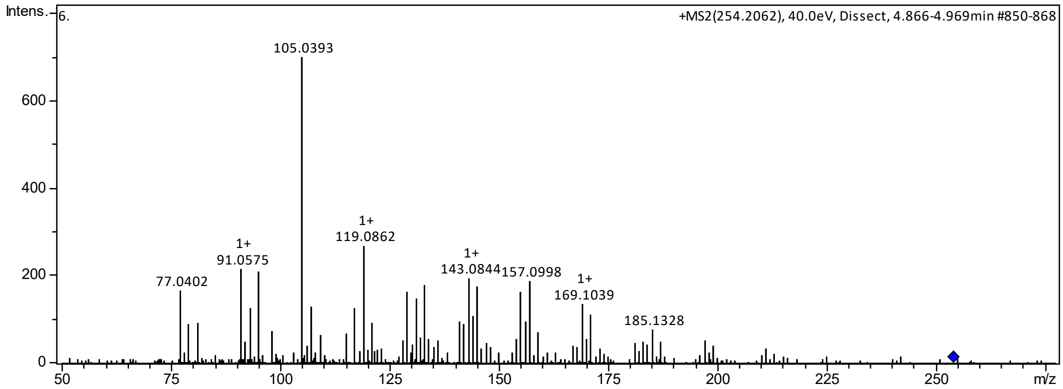

Figure S53. +ESI-QqTOF MS/MS spectrum of O-benzoyl-cycloprotobuxoline-D (**8**).

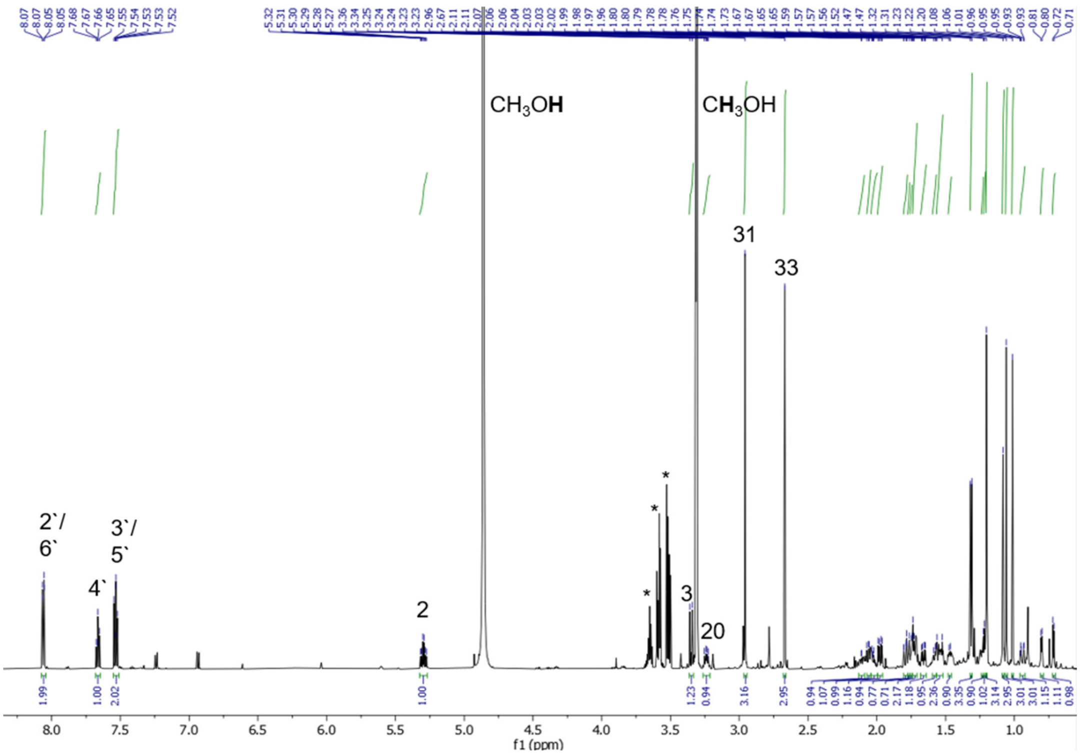

Figure S54. ^{1}H NMR spectrum of O-benzoyl-cycloprotobuxoline-D (**8**) (CD_3OD, 600 MHz). The assignment of the signals between 0.7 and 2.15 ppm can be found in the enlarged Figure S55 (*signals of 17.2% glycerol).

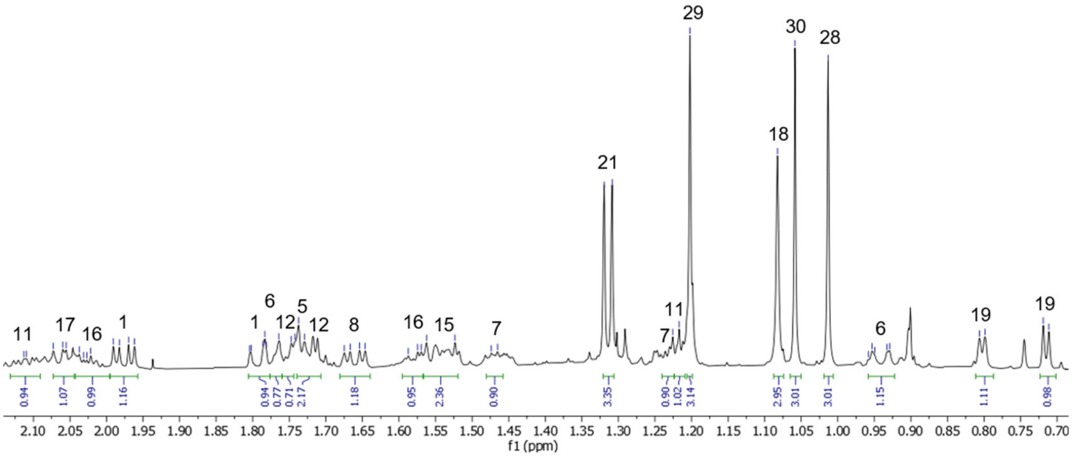

Figure S55. Detail of the ^{1}H NMR spectrum of O-benzoyl-cycloprotobuxoline-D (**8**) (CD_3OD, 600 MHz).

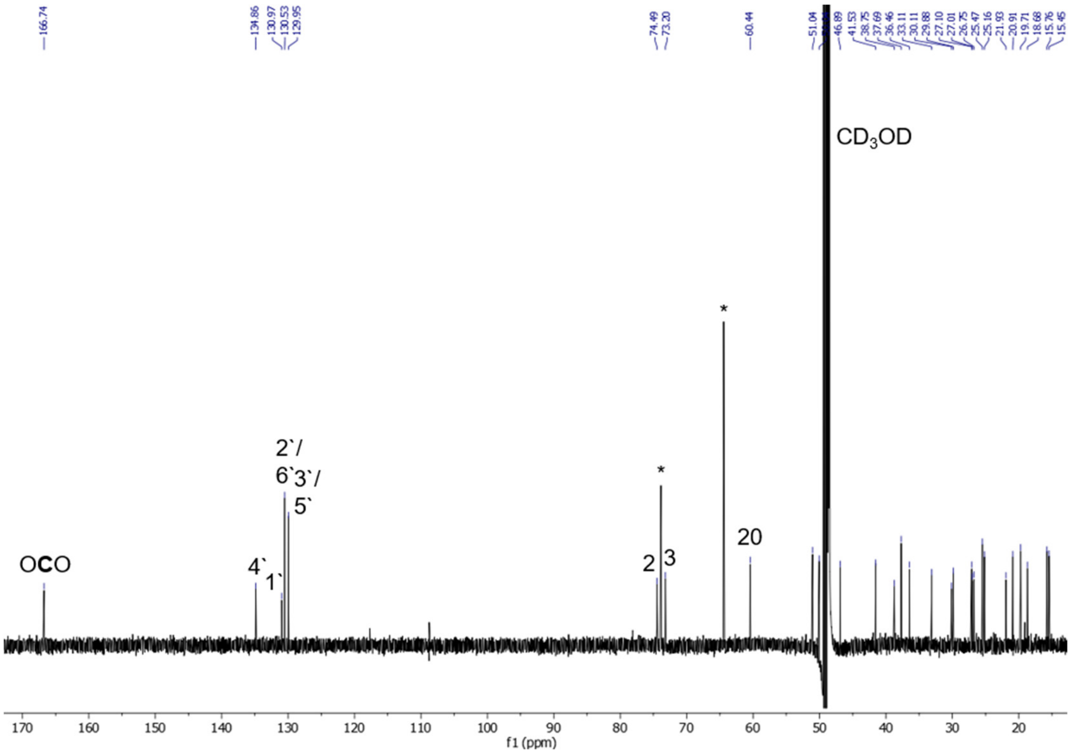

Figure S56. ^{13}C NMR spectrum of O-benzoyl-cycloprotobuxoline-D (**8**) (CD_3OD, 150 MHz). The assignment of the signals between 15 and 52 ppm can be found in the enlarged Figure S57 (*signals of 17.2% glycerol).

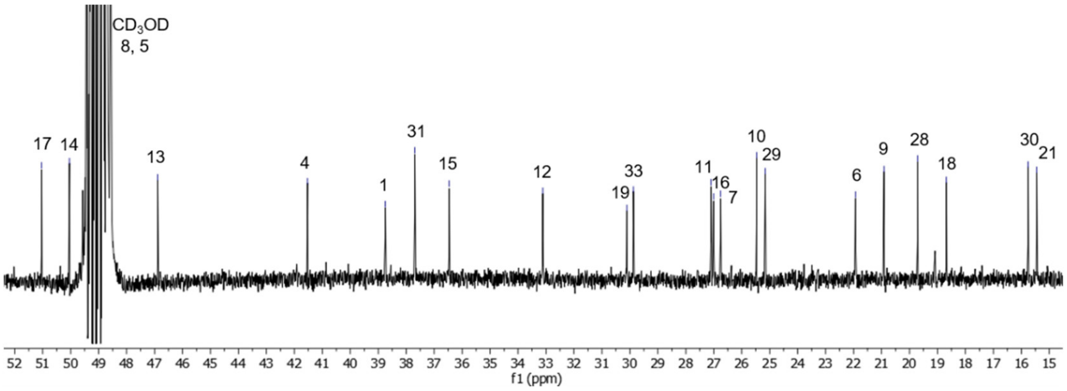

Figure S57. Detail of the ^{13}C NMR spectrum of O-benzoyl-cycloprotobuxoline-D (**8**) (CD_3OD, 150 MHz).

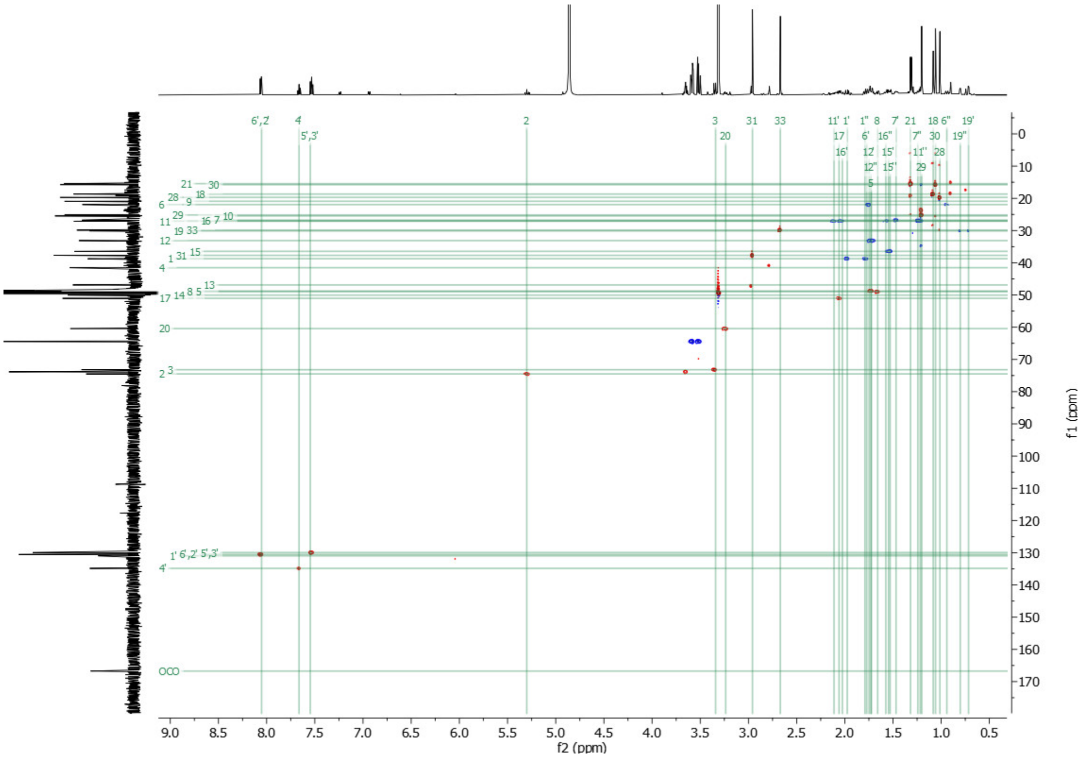

Figure S58. $^1H/^{13}C$ HSQC spectrum of O-benzoyl-cycloprotobuxoline-D (**8**) (CD_3OD, 600/150 MHz).

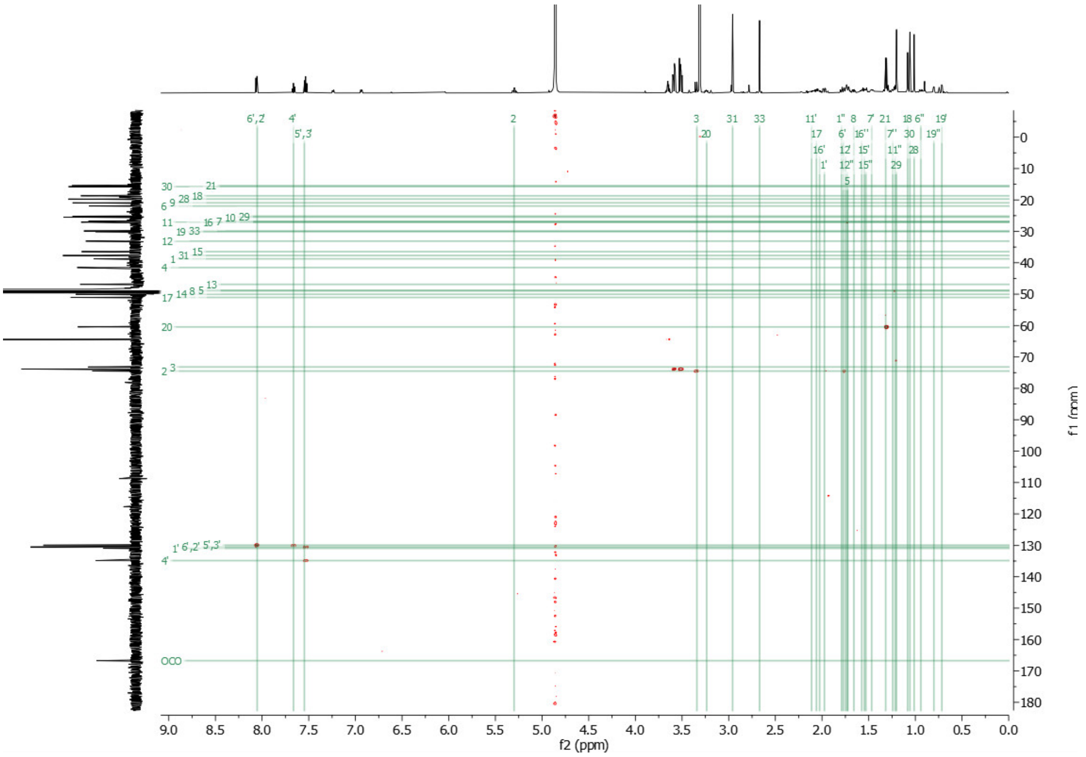

Figure S59. $^{1}H/^{13}C$ H2BC spectrum of O-benzoyl-cycloprotobuxoline-D (**8**) (CD_3OD, 600/150 MHz).

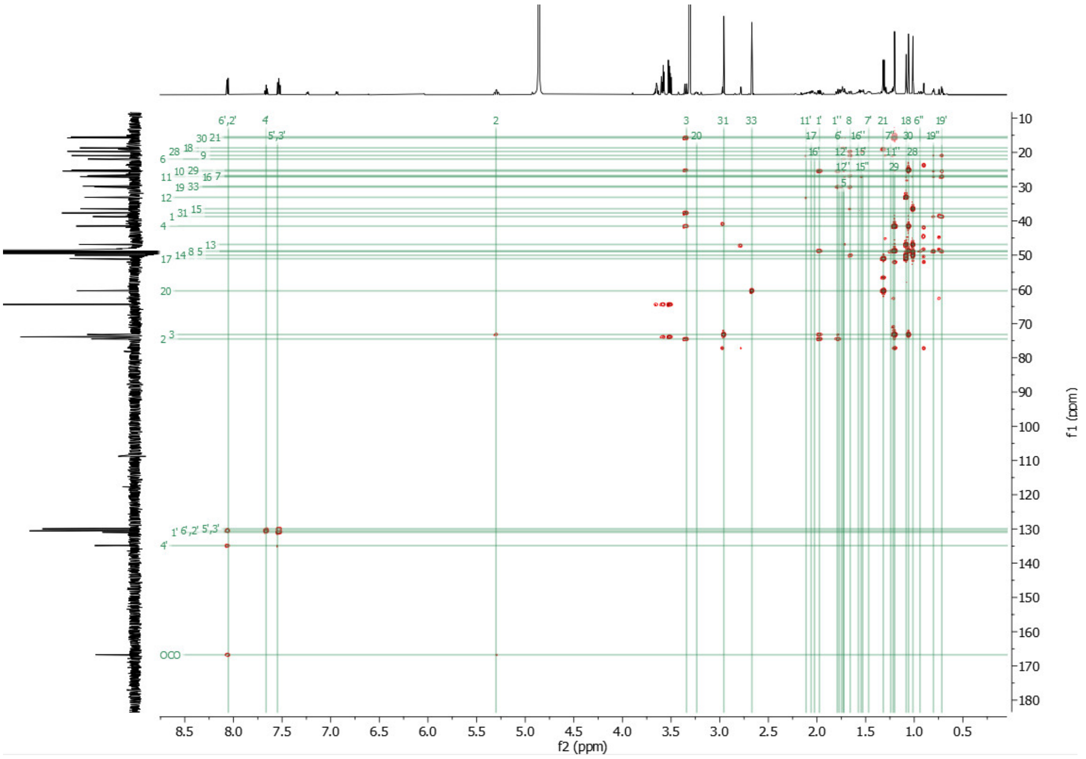

Figure S60. $^{1}H/^{13}C$ HMBC spectrum of O-benzoyl-cycloprotobuxoline-D (**8**) (CD_3OD, 600/150 MHz).

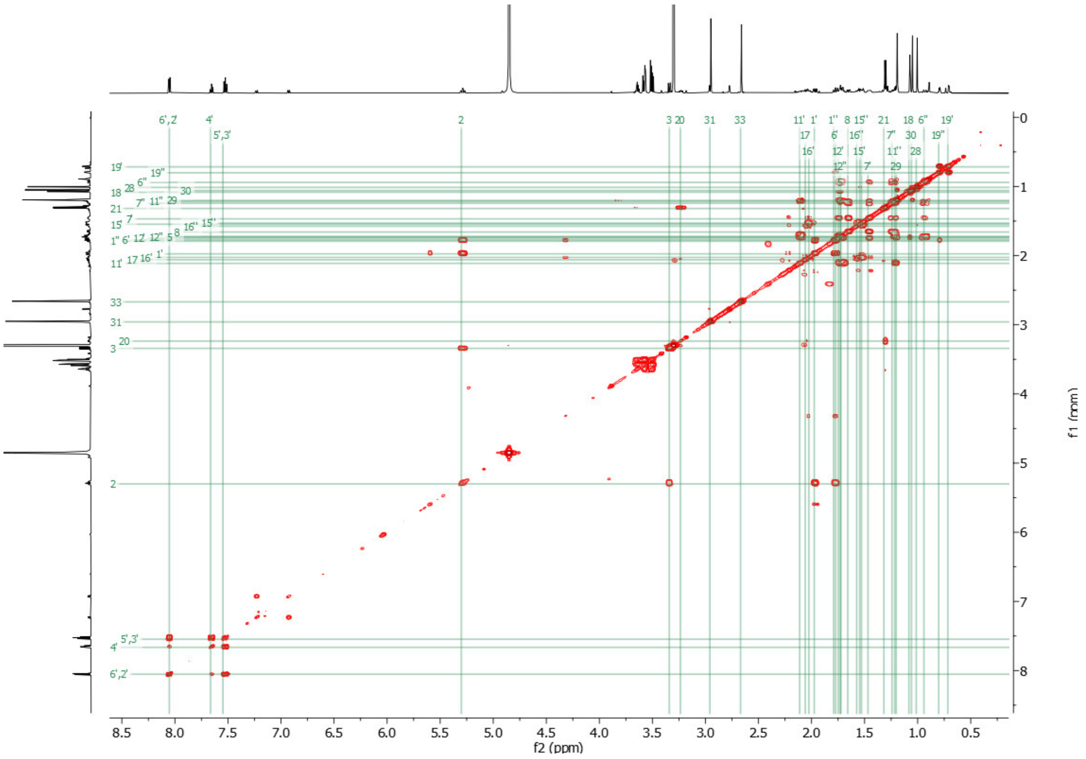

Figure S61. $^{1}H/^{1}H$ COSY spectrum of O-benzoyl-cycloprotobuxoline-D (**8**) (CD_3OD, 600 MHz).

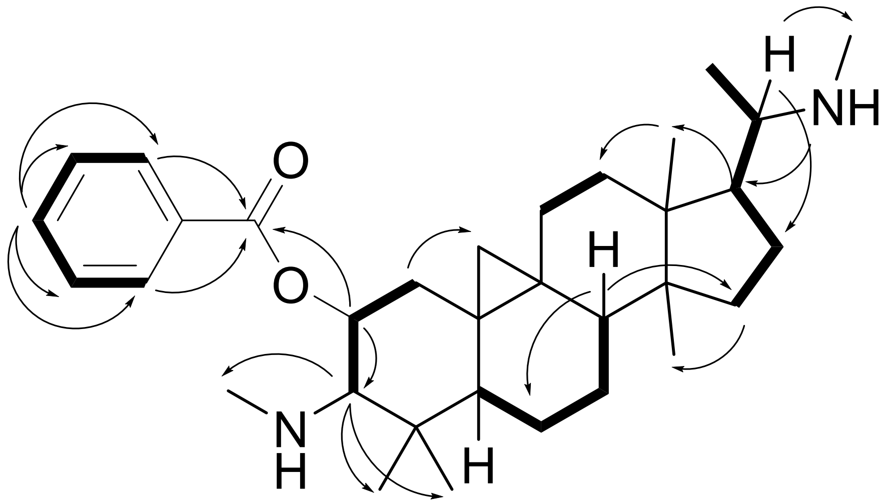

Figure S62. Key COSY (bold lines) and HMBC (arrows) correlations of O-benzoyl-cycloprotobuxoline-D (**8**).

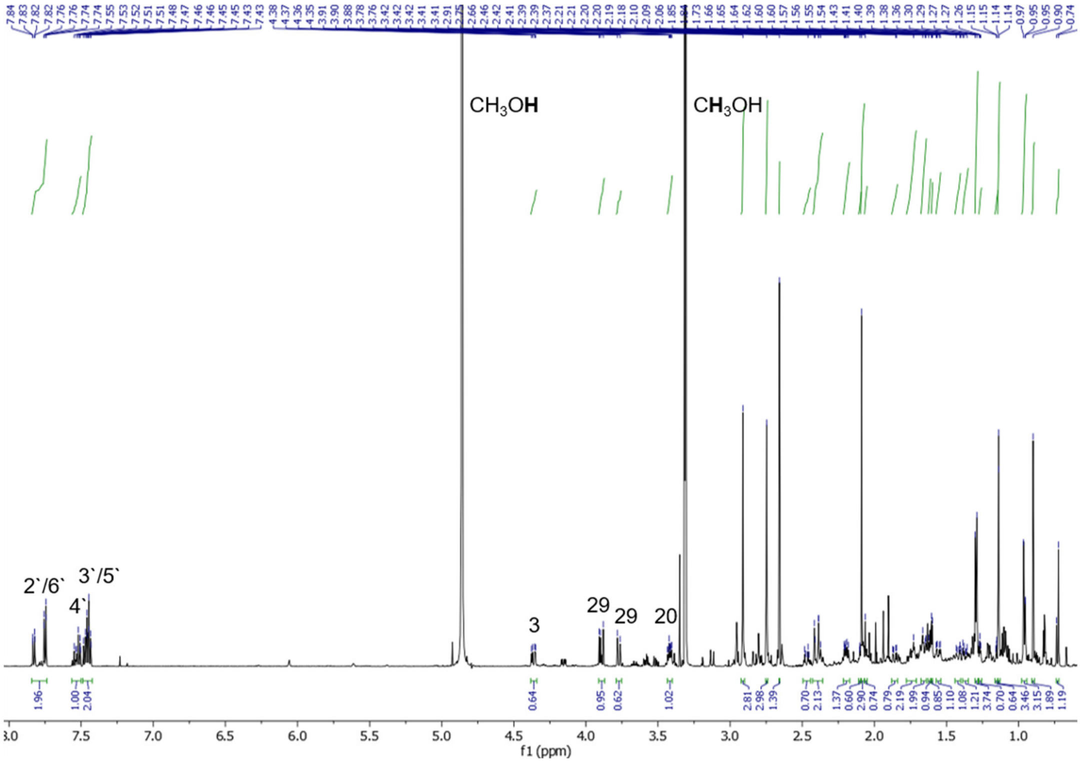

Figure S63. ^{1}H NMR spectrum of N-benzoyl-O-acetyl-cycloxo-buxoline-F (**9**) (CD_3OD, 600 MHz). The assignment of the signals between 0.85 and 2.93 ppm can be found in the enlarged Figure S64.

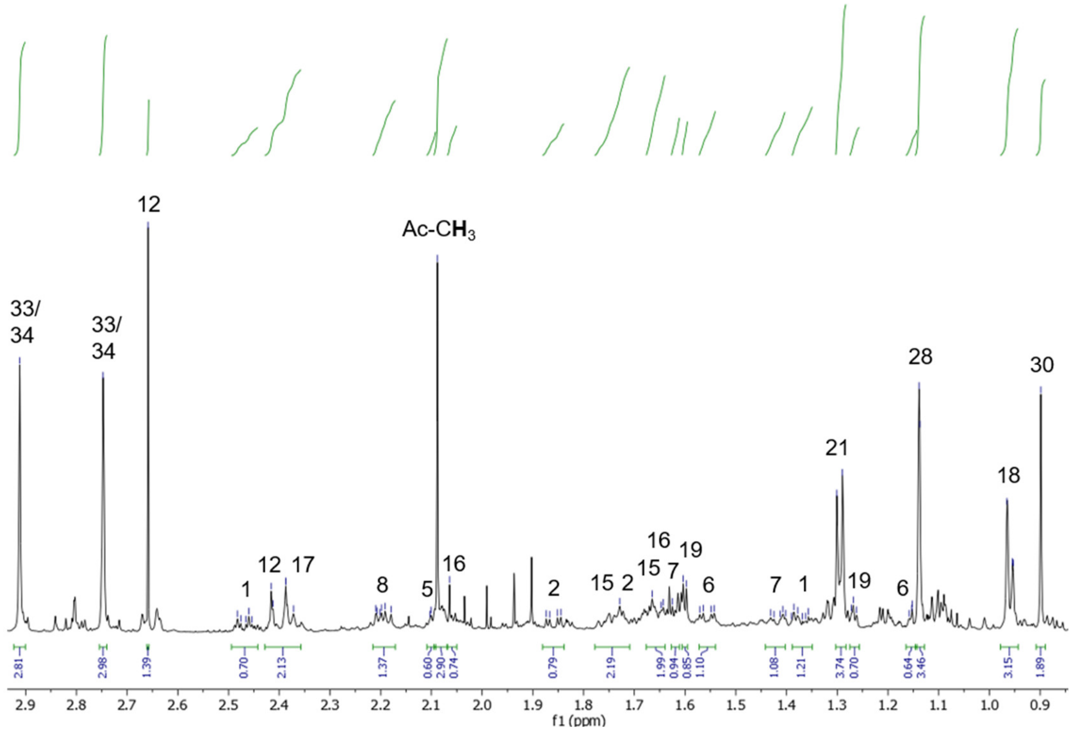

Figure S64. Detail of the ^{1}H NMR spectrum of N-benzoyl-O-acetyl-cycloxo-buxoline-F (**9**) (CD_3OD, 600 MHz).

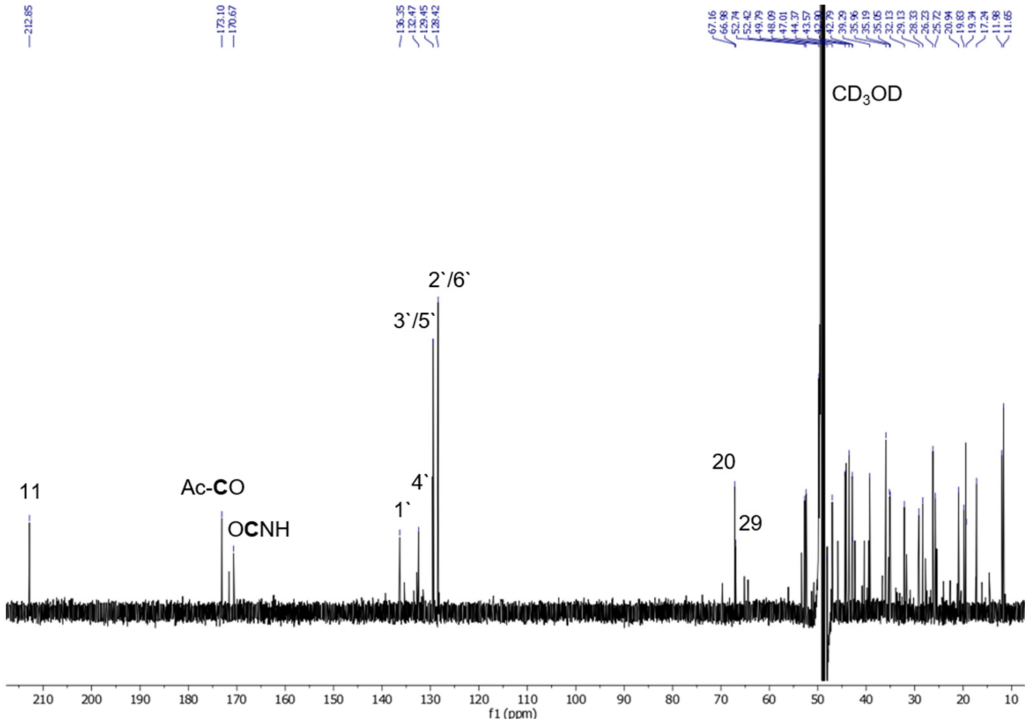

Figure S65. ^{13}C NMR spectrum of N-benzoyl-O-acetyl-cycloxo-buxoline-F (**9**) (CD_3OD, 150 MHz). The assignment of the signals between 11.5 and 52.8 ppm can be found in the enlarged Figure S66.

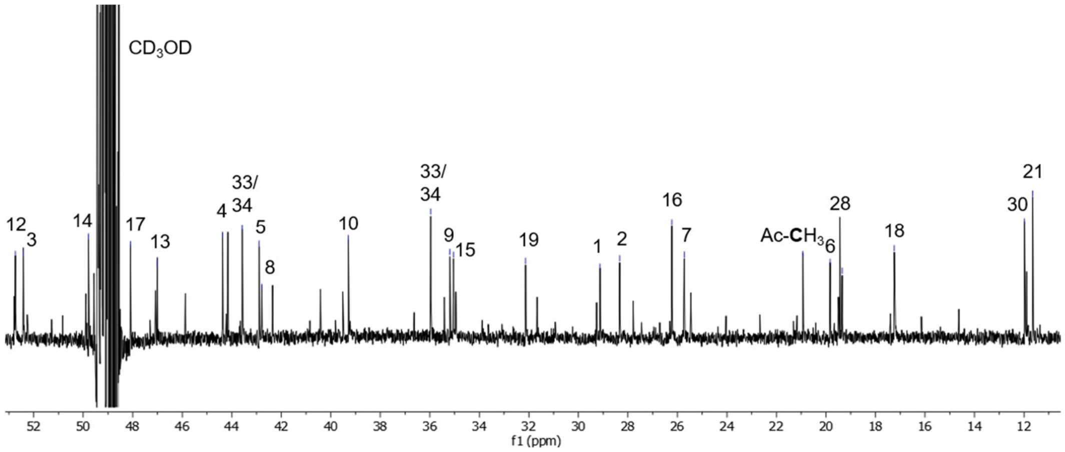

Figure S66. Detail of the ^{13}C NMR spectrum of N-benzoyl-O-acetyl-cycloxo-buxoline-F (**9**) (CD_3OD, 150 MHz).

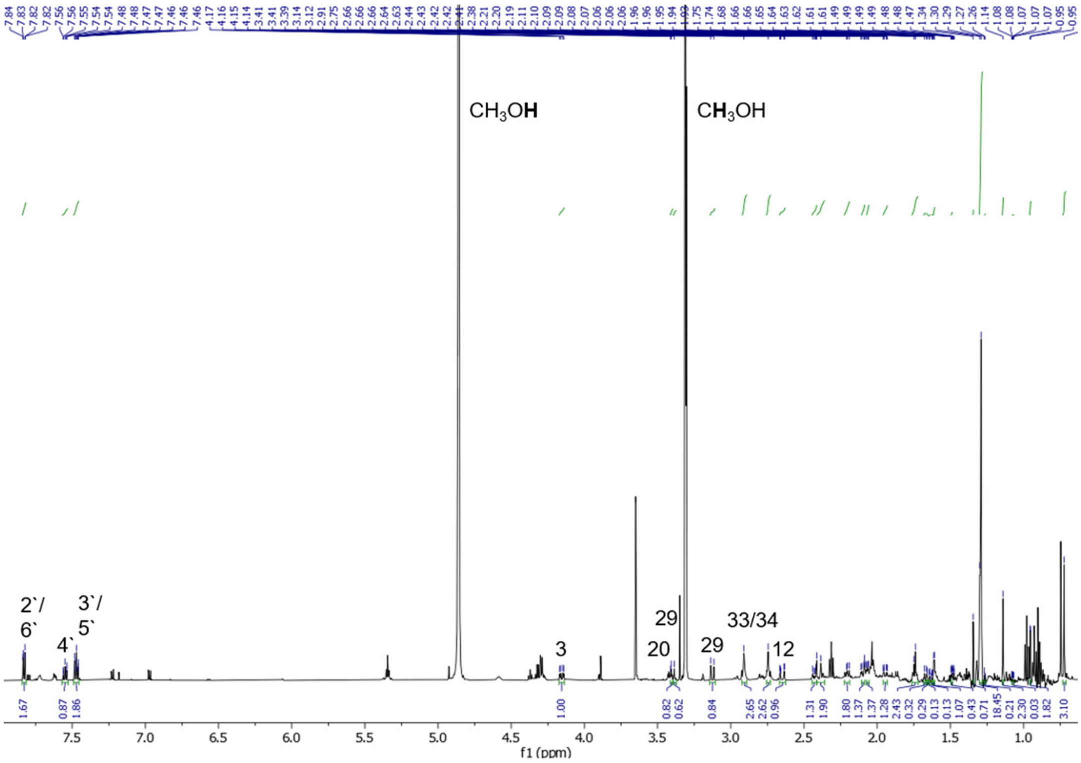

Figure S67. ^{1}H NMR spectrum of N-benzoyl-cycloxo-buxoline-F (**10**) (CD_3OD, 600 MHz). The assignment of the signals between 0.7 and 2.5 ppm can be found in the enlarged Figure S68.

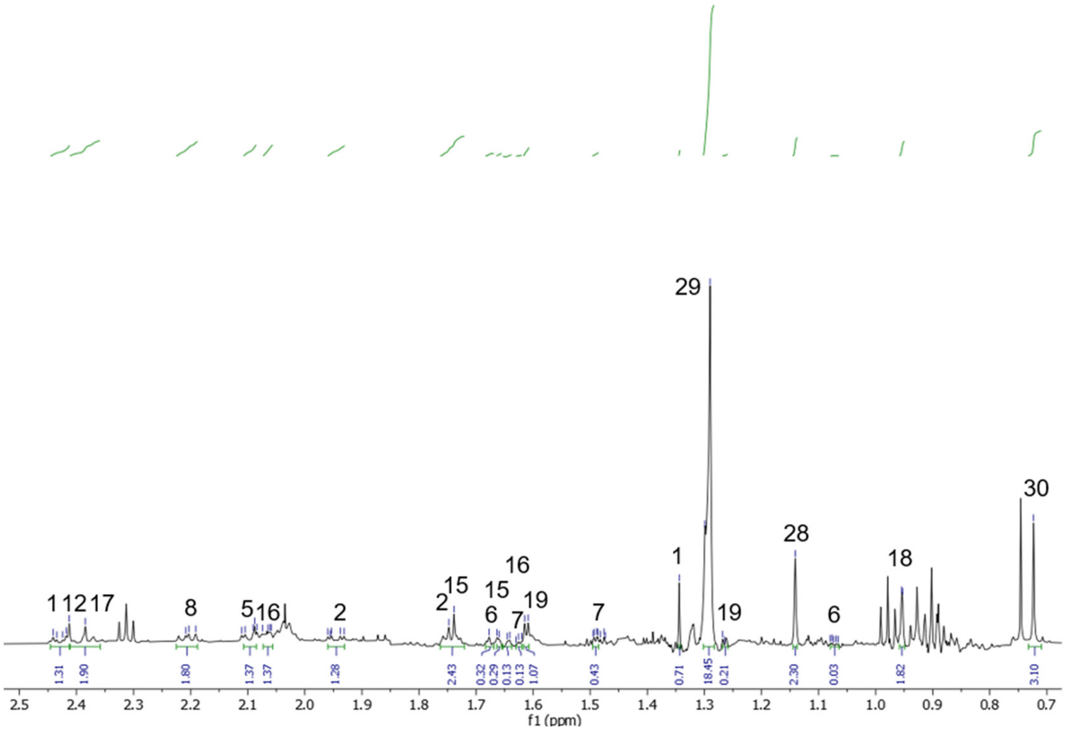

Figure S68. Detail of the ^{1}H NMR spectrum of N-benzoyl-cycloxo-buxoline-F (**10**) (CD_3OD, 600 MHz).

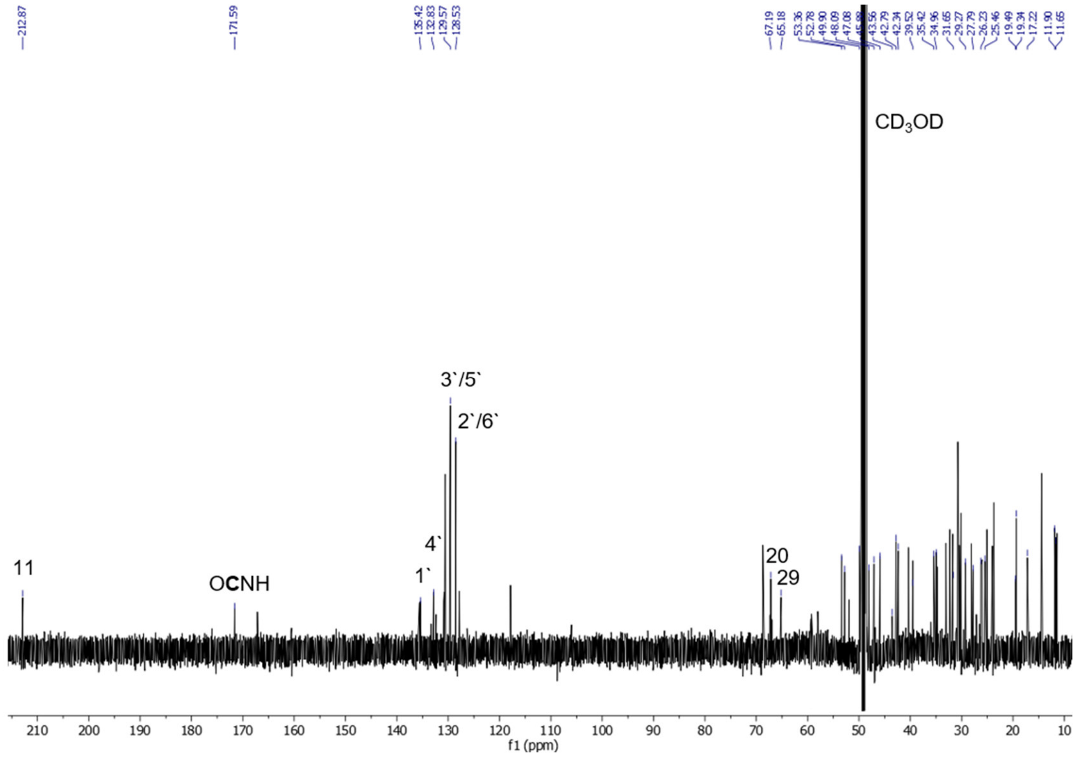

Figure S69. ^{13}C NMR spectrum of N-benzoyl-cycloxo-buxoline-F (**10**) (CD3OD, 150 MHz). The assignment of the signals between 11 and 54 ppm can be found in the enlarged Figure S70.

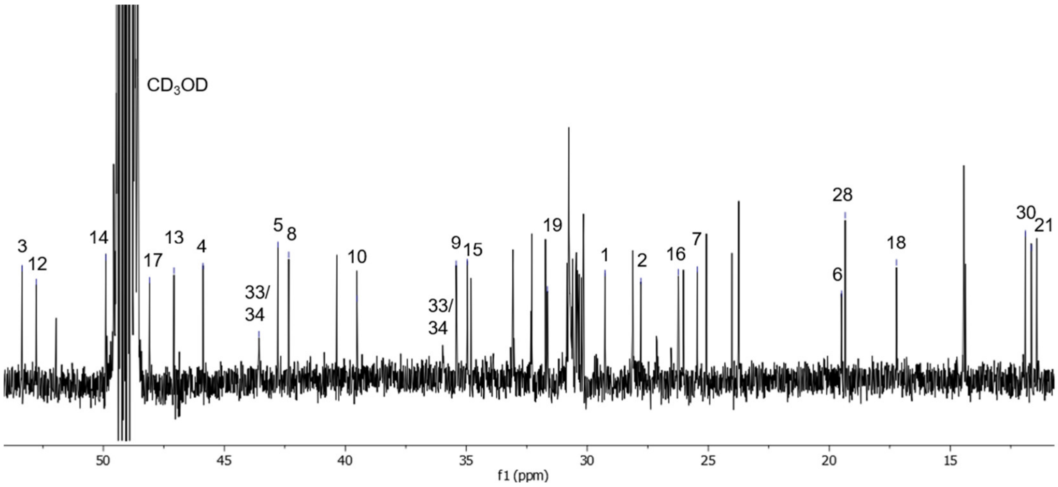

Figure S70. Detail of the ^{13}C NMR spectrum of N-benzoyl-cycloxo-buxoline-F (**10**) (CD3OD, 150 MHz).

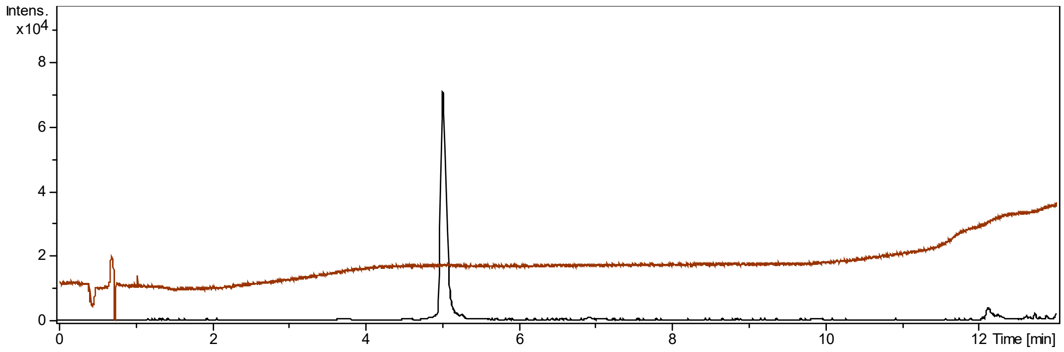

Figure S71. UHPLC/+ESI-QqTOF-MS/MS chromatogram of 29-hydroxy-cyclomikuranine-L (**11**). Base peak chromatogram 200.0000-1000.0000 +All MS (black); UV-Chromatogramm, 200-400 nm (red).

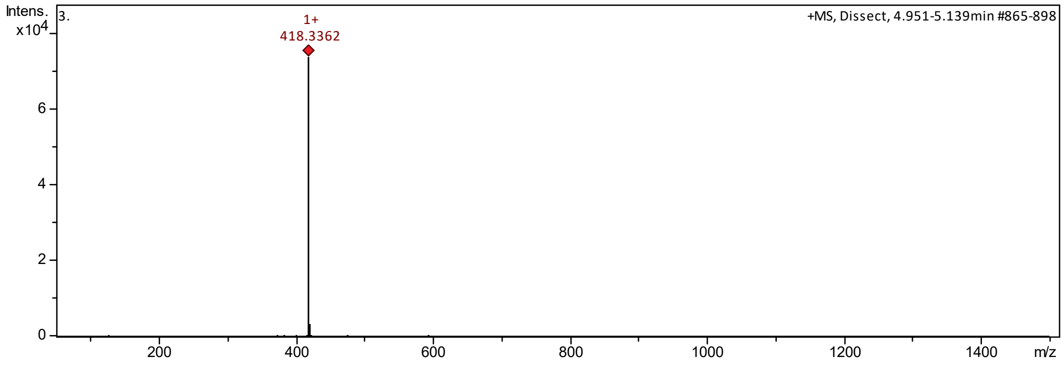

Figure S72. +ESI-QqTOF MS spectrum of 29-hydroxy-cyclomikuranine-L (**11**); *m/z* 418.3362 [M+H]+.

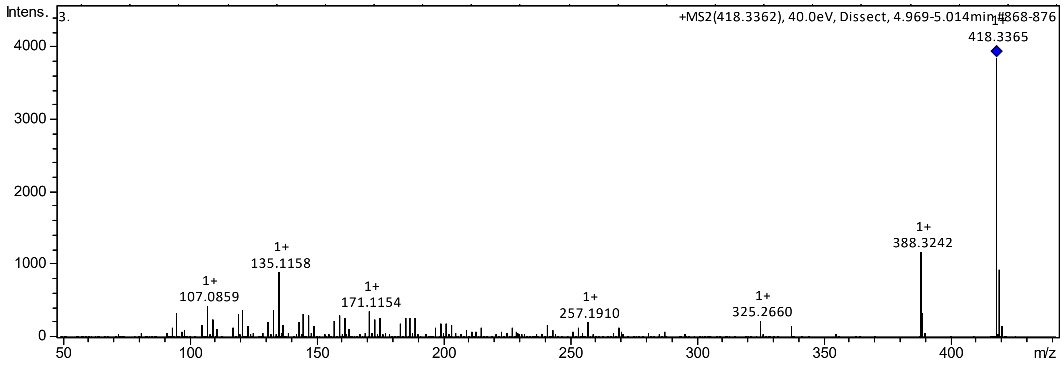

Figure S73. +ESI-QqTOF MS/MS spectrum of 29-hydroxy-cyclomikuranine-L (**11**).

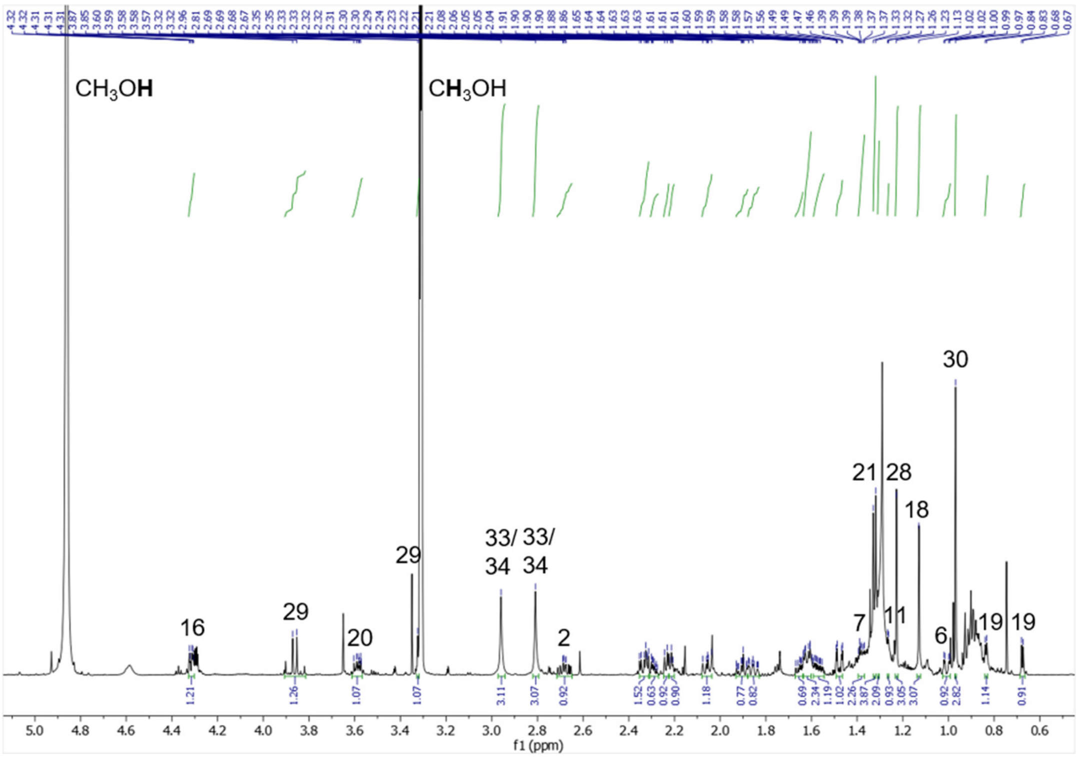

Figure S74. ^{1}H NMR spectrum of 29-hydroxy-cyclomikuranine-L (**11**) (CD_3OD, 600 MHz). The assignment of the signals between 1.45 and 2.4 ppm can be found in the enlarged Figure S75.

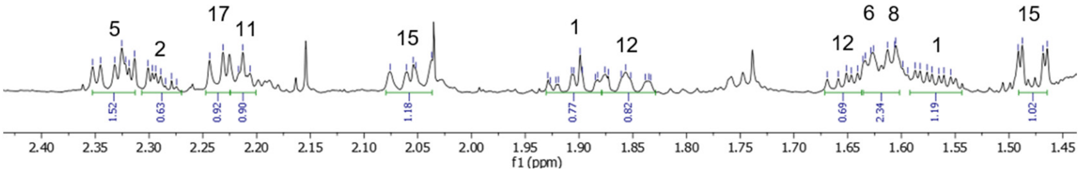

Figure S75. Detail of the ^{1}H NMR spectrum of 29-hydroxy-cyclomikuranine-L (**11**) (CD_3OD, 600 MHz).

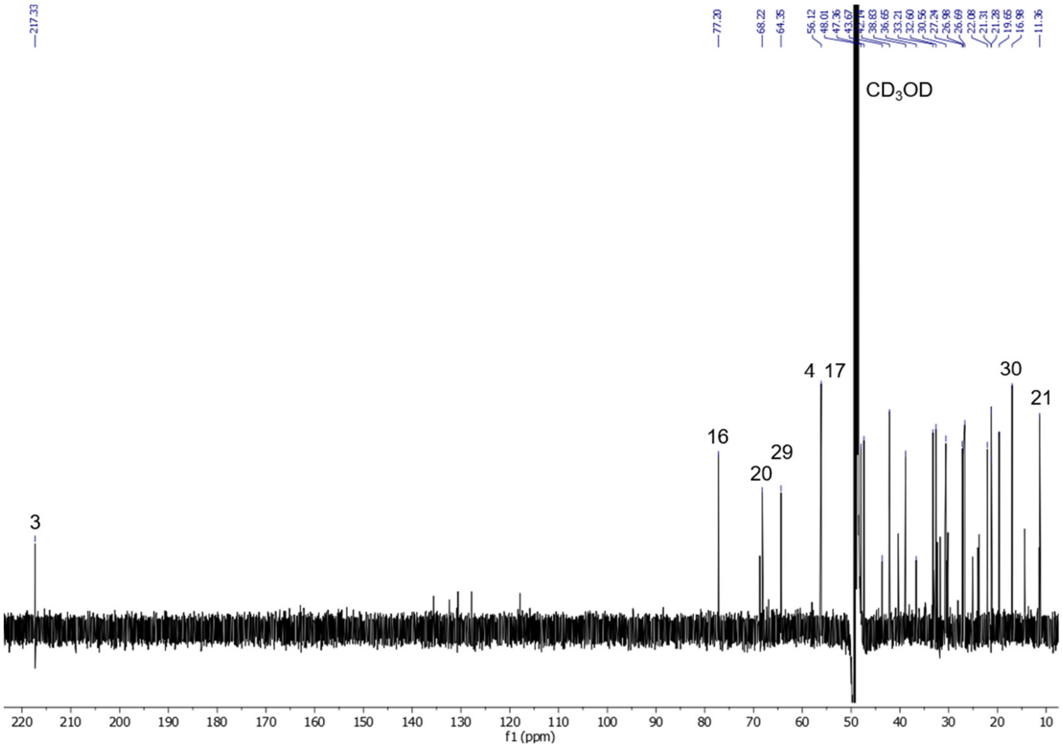

Figure S76. ^{13}C NMR spectrum of 29-hydroxy-cyclomikuranine-L (**11**) (CD$_3$OD, 150 MHz). The assignment of the signals between 19 and 49.5 ppm can be found in the enlarged Figure S77.

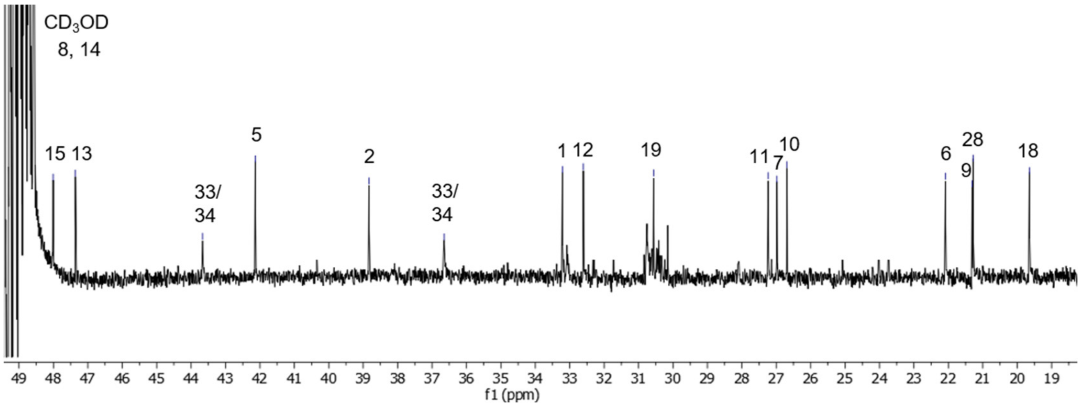

Figure S77. Detail of the ^{13}C NMR spectrum of 29-hydroxy-cyclomikuranine-L (**11**) (CD$_3$OD, 150 MHz).

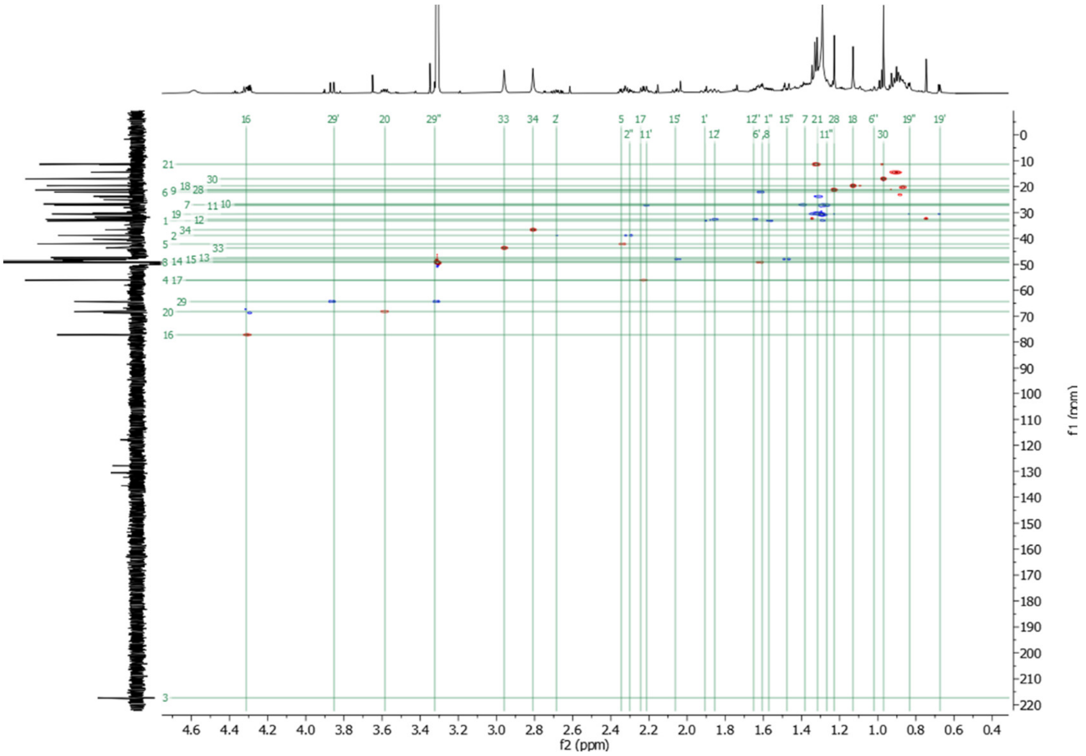

Figure S78. ^{1}H/^{13}C HSQC spectrum of 29-hydroxy-cyclomikuranine-L (**11**) (CD_3OD, 600/150 MHz).

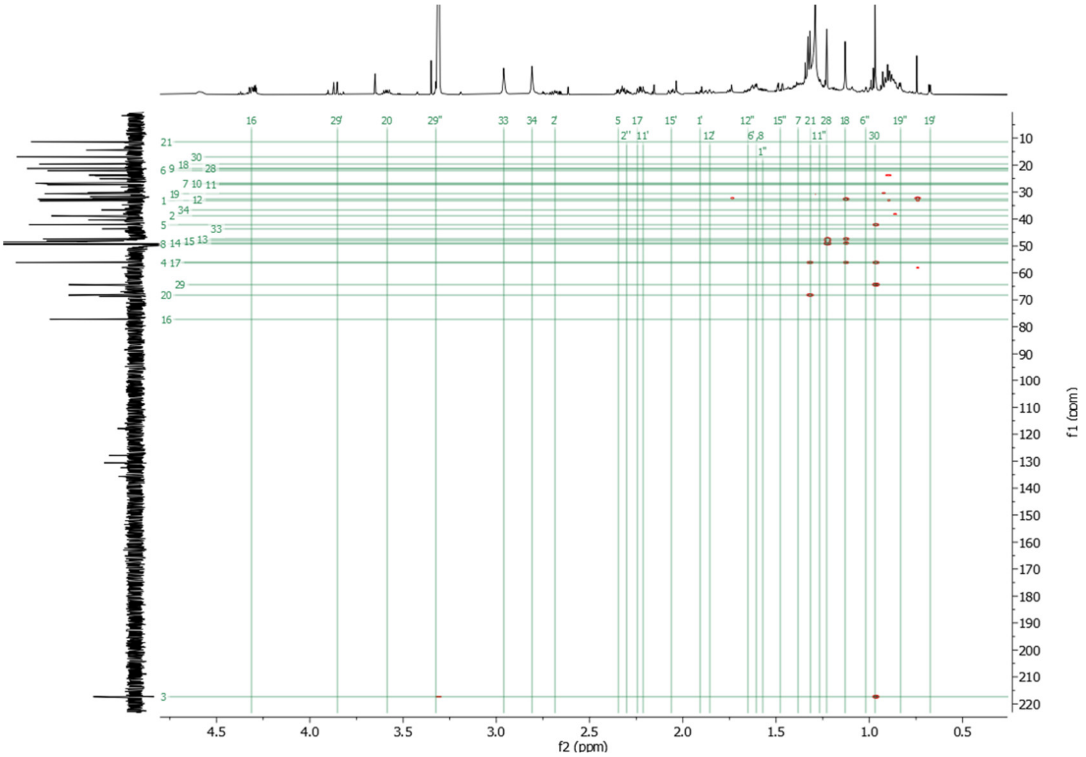

Figure S79. ^{1}H/^{13}C HMBC spectrum of 29-hydroxy-cyclomikuranine-L (**11**) (CD_3OD, 600/150 MHz).

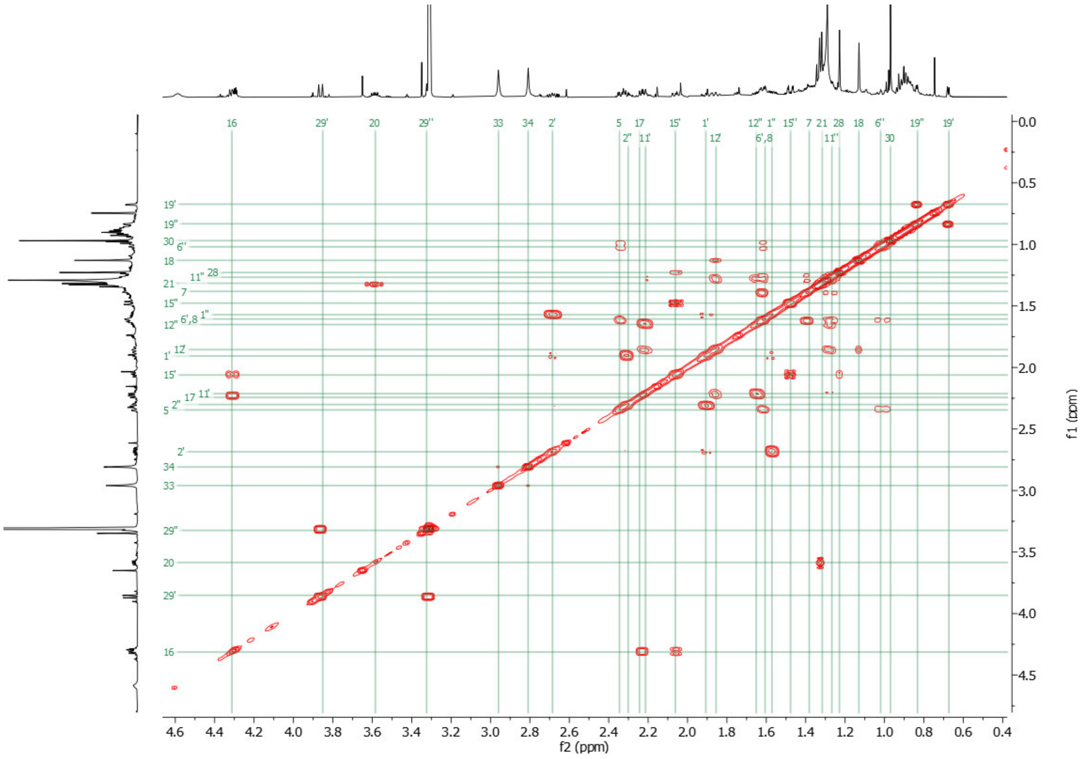

Figure S80. $^1H/^1H$ COSY spectrum of 29-hydroxy-cyclomikuranine-L (**11**) (CD_3OD, 600 MHz).

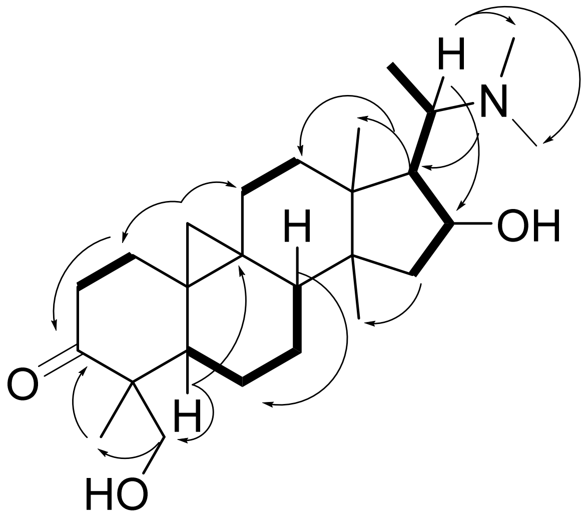

Figure S81. Key COSY (bold lines) and HMBC (arrows) correlations of 29-hydroxy-cyclomikuranine-L (**11**).

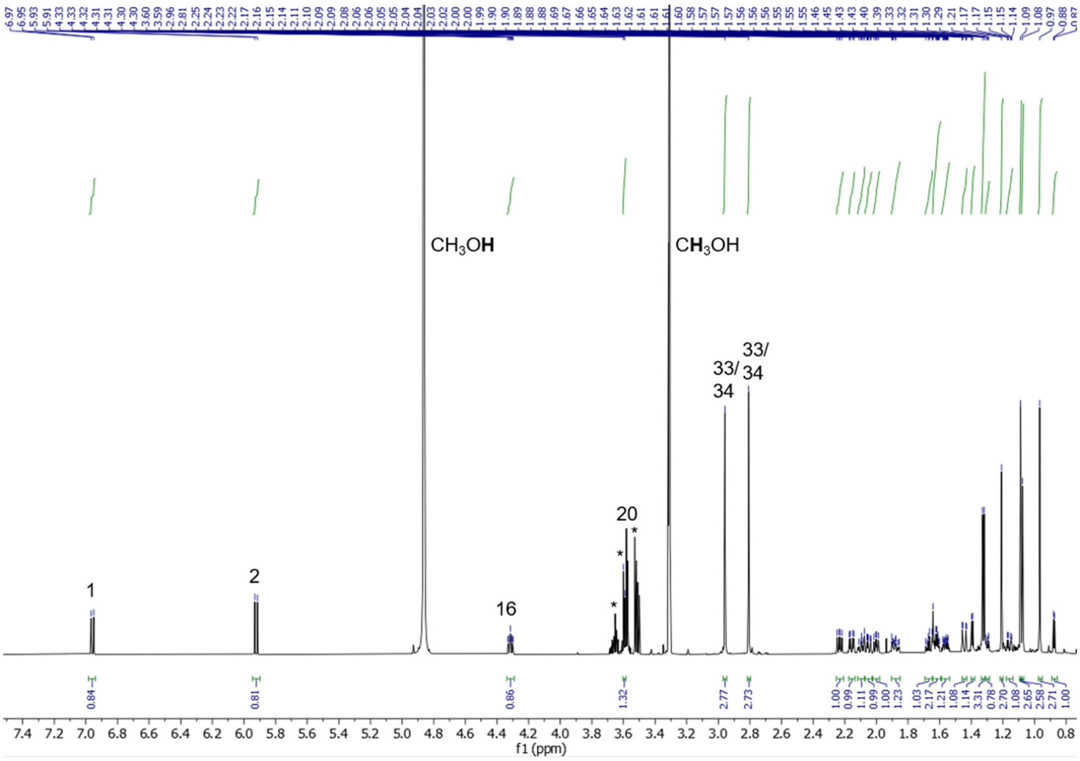

Figure S82. ^{1}H NMR spectrum of N$_b$-dimethylcycloxobuxoviricine (**12**) (CD_3OD, 600 MHz). The assignment of the signals between 0.8 and 2.3 ppm can be found in the enlarged Figure S83 (*signals of 11.9% glycerol).

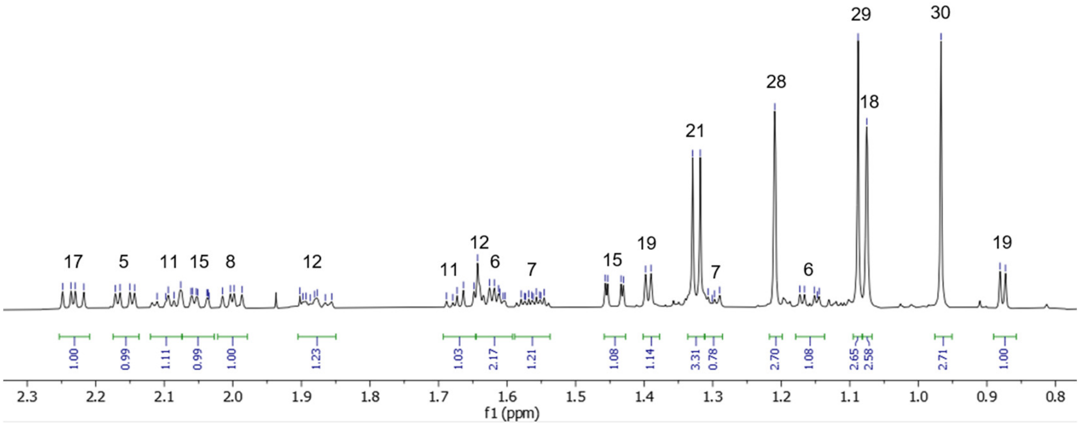

Figure S83. Detail of the ^{1}H NMR spectrum of N$_b$-dimethylcycloxobuxoviricine (**12**) (CD_3OD, 600 MHz).

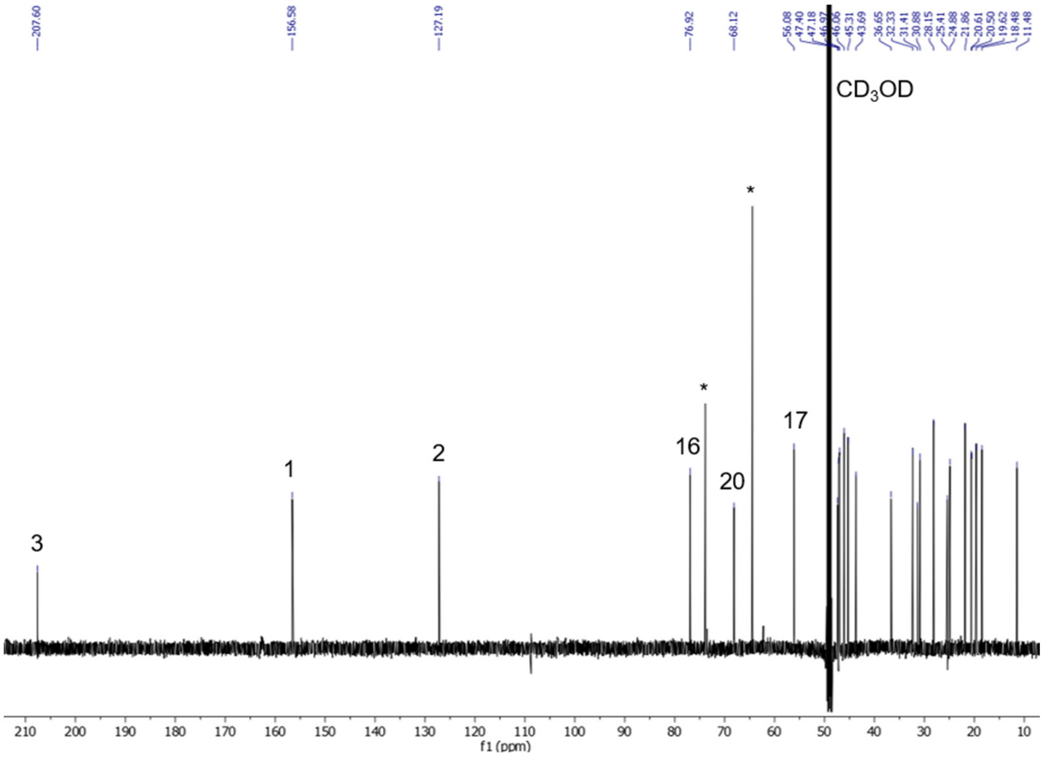

Figure S84. ^{13}C NMR spectrum of N_b-dimethylcycloxobuxoviricine (**12**) (CD_3OD, 150 MHz). The assignment of the signals between 11 and 50 ppm can be found in the enlarged Figure S85 (*signals of 11.9% glycerol).

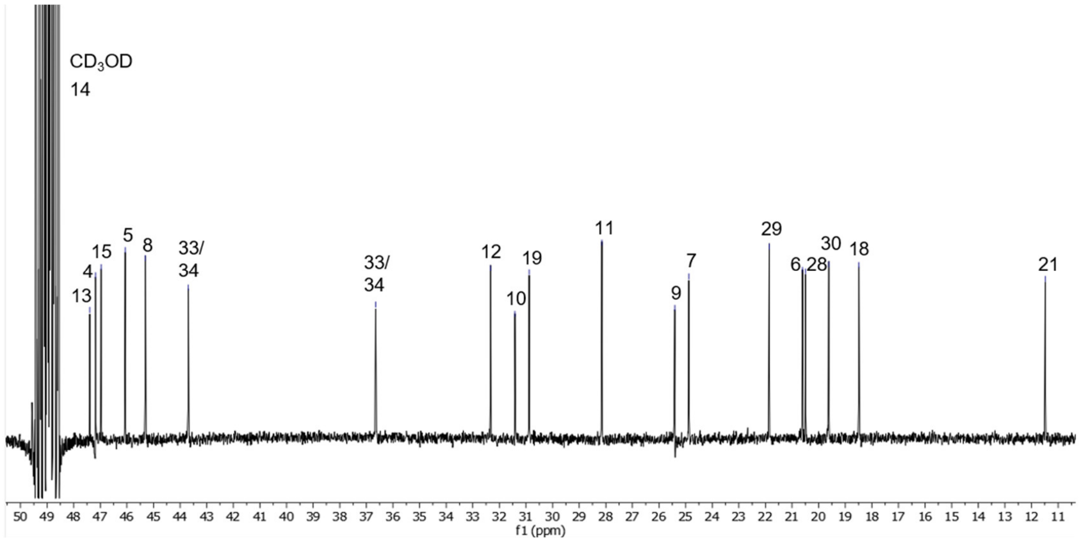

Figure S85. Detail of the ^{13}C NMR spectrum of N_b-dimethylcycloxobuxoviricine (**12**) (CD_3OD, 150 MHz).

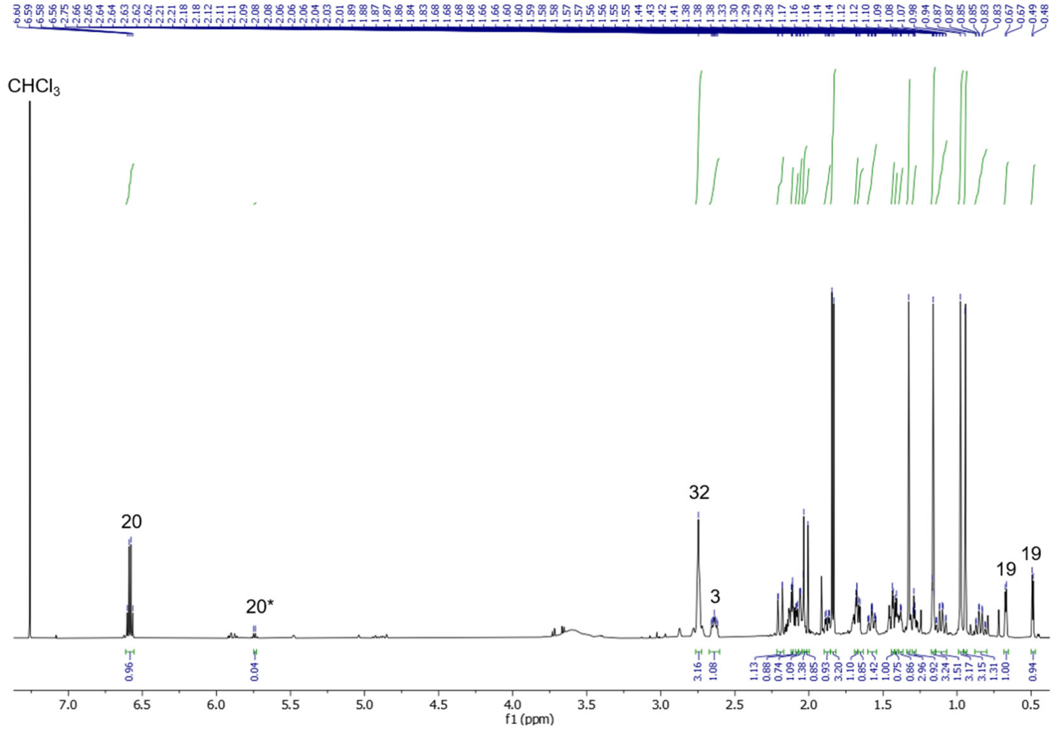

Figure S86. ^{1}H NMR spectrum of (*E*)-cyclobuxophyllinine-M (**13**) and (*Z*)-cyclobuxophyllinine-M (**14**) ($CDCl_3$, 600 MHz). The assignment of the signals between 0.8 and 2.2 ppm can be found in the enlarged Figure S87 (*signal of compound **14**).

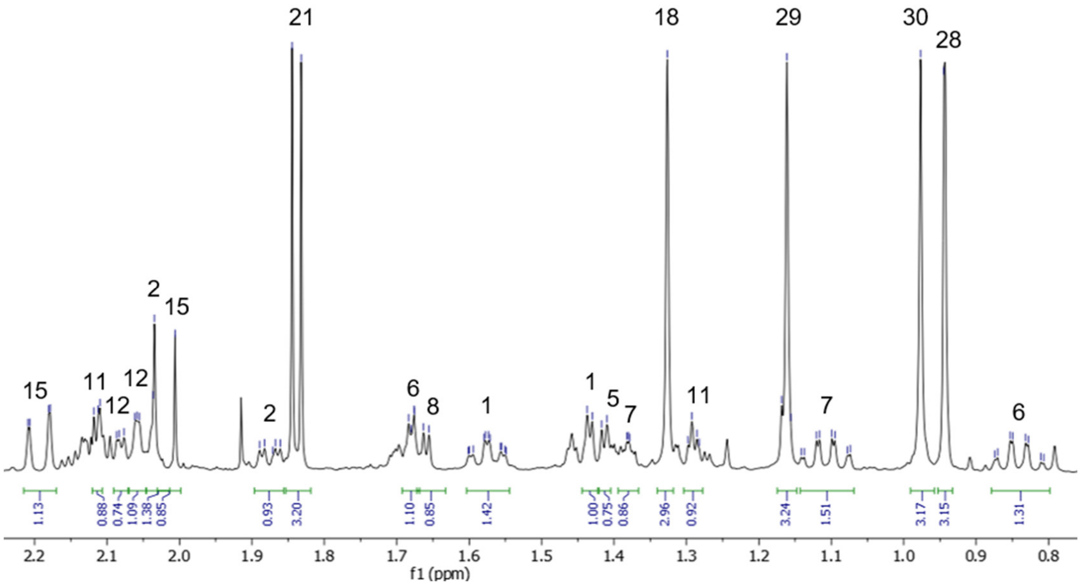

Figure S87. Detail of the ^{1}H NMR spectrum of (*E*)-cyclobuxophyllinine-M (**13**) and (*Z*)-cyclobuxophyllinine-M (**14**) ($CDCl_3$, 600 MHz).

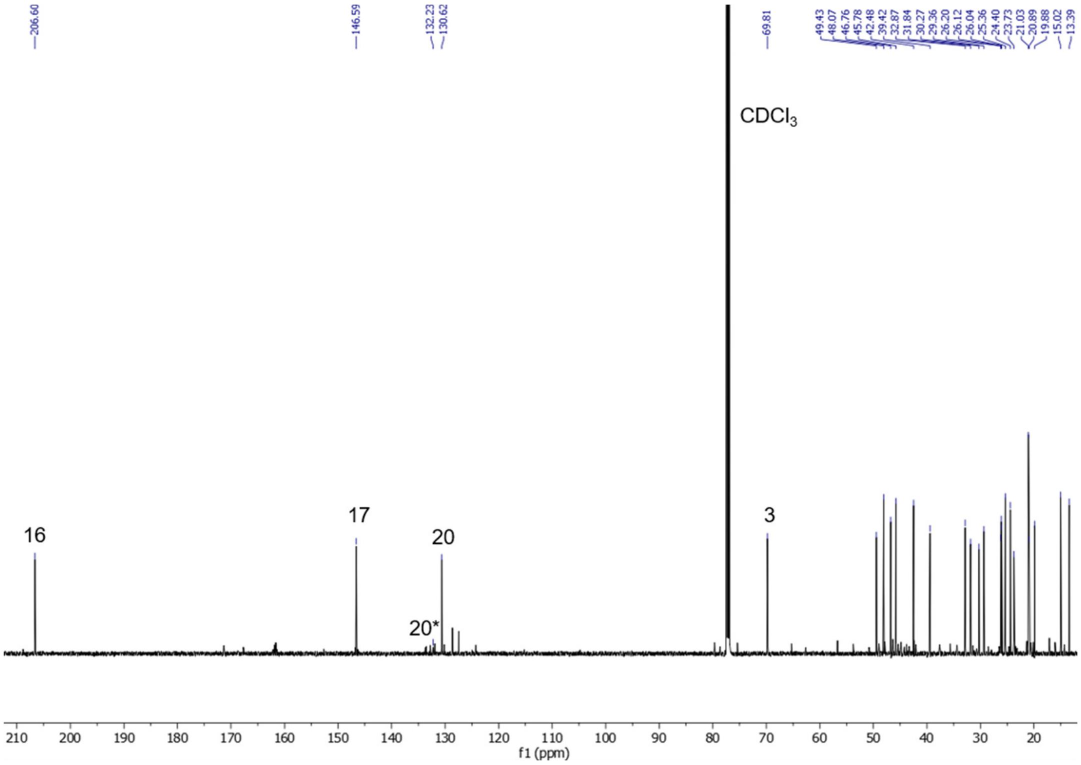

Figure S88. ^{13}C NMR spectrum of (*E*)-cyclobuxophyllinine-M (**13**) and (*Z*)-cyclobuxophyllinine-M (**14**) ($CDCl_3$, 150 MHz). The assignment of the signals between 13 and 50 ppm can be found in the enlarged Figure S89 (*signal of compound **14**).

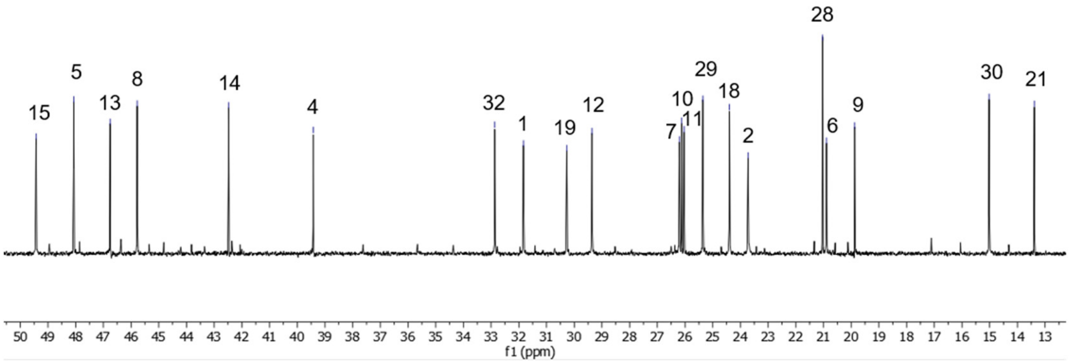

Figure S89. Detail of the ^{13}C NMR spectrum of (*E*)-cyclobuxophyllinine-M (**13**) and (*Z*)-cyclobuxophyllinine-M (**14**) ($CDCl_3$, 150 MHz).

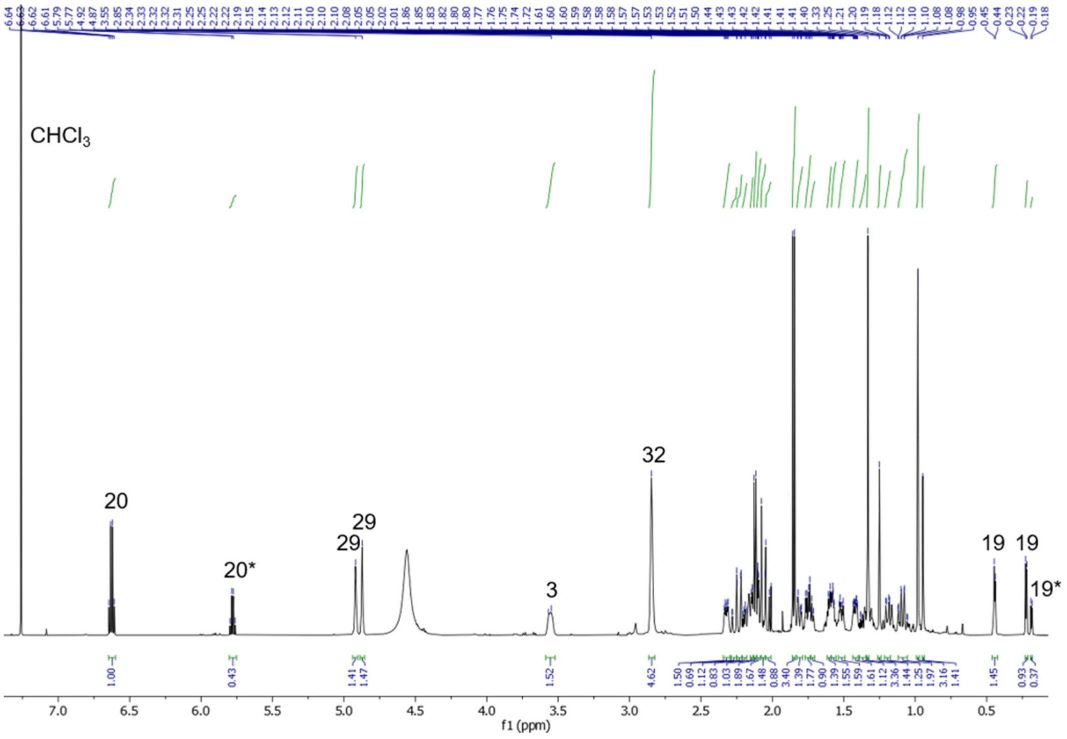

Figure S90. ^{1}H NMR spectrum of (*E*)-cyclosuffrobuxinine-M (**15**) and (*Z*)-cyclosuffrobuxinine-M (**16**) (CDCl3, 600 MHz). The assignment of the signals between 0.9 and 2.4 ppm can be found in the enlarged Figure S91 (*signals of compound **16**).

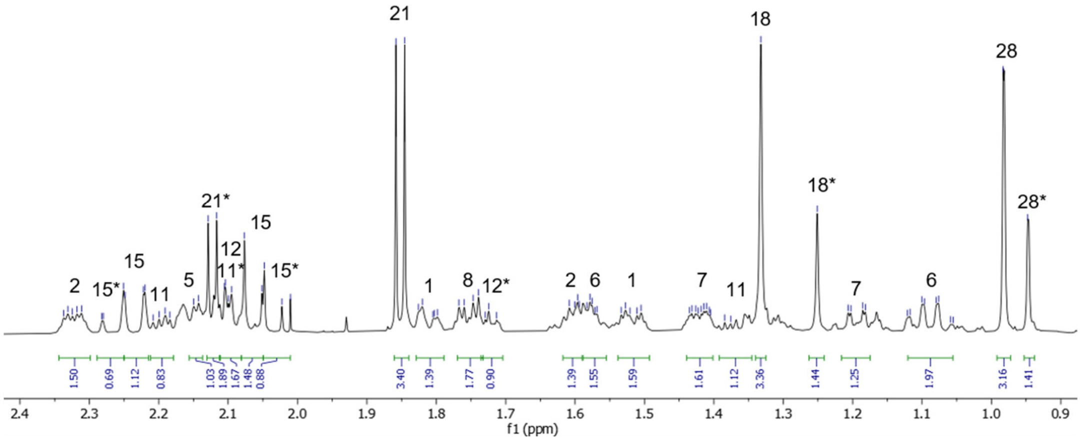

Figure S91. Detail of the ^{1}H NMR spectrum of (*E*)-cyclosuffrobuxinine-M (**15**) and (*Z*)-cyclosuffrobuxinine-M (**16**) (CDCl3, 600 MHz) (*signals of compound **16**).

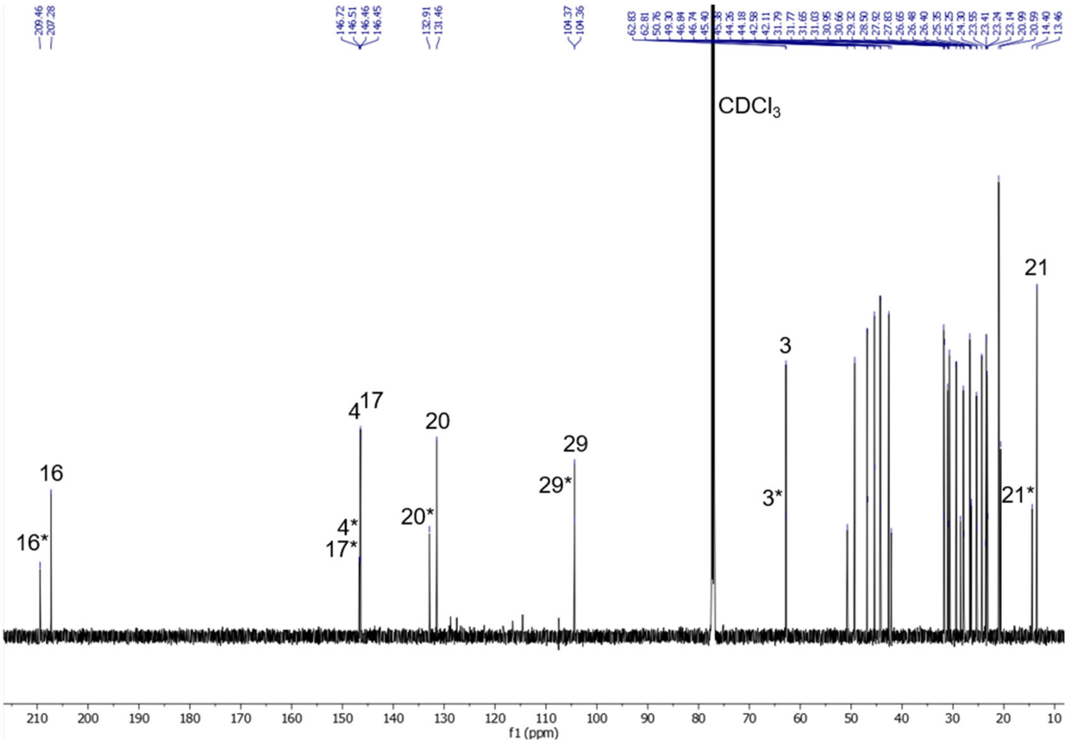

Figure S92. ^{13}C NMR spectrum of (*E*)-cyclosuffrobuxinine-M (**15**) and (*Z*)-cyclosuffrobuxinine-M (**16**) (CDCl3, 150 MHz). The assignment of the signals between 20 and 52 ppm can be found in the enlarged Figure S93 (*signals of compound **16**).

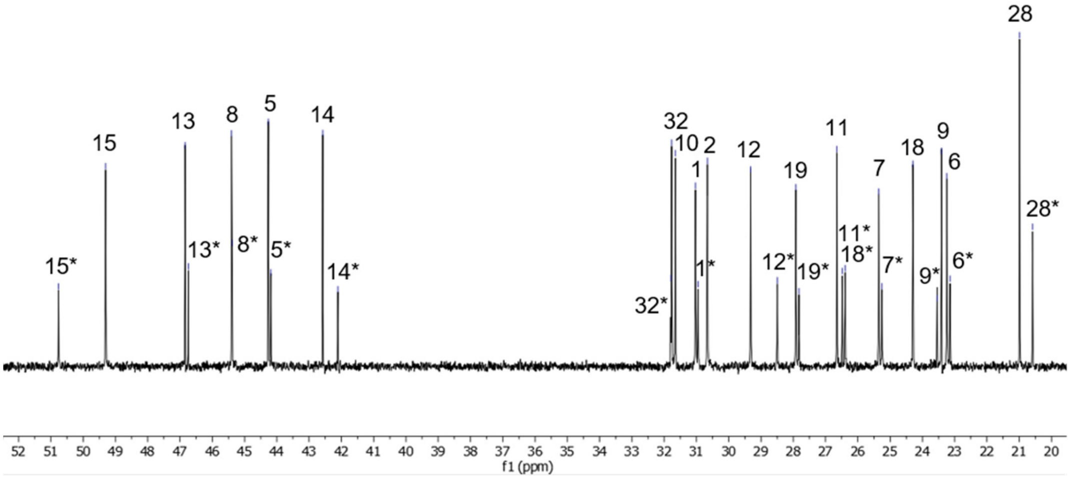

Figure S93. Detail of the ^{13}C NMR spectrum of (*E*)-cyclosuffrobuxinine-M (**15**) and (*Z*)-cyclosuffrobuxinine-M (**16**) (CDCl3, 150 MHz) (*signals of compound **16**).

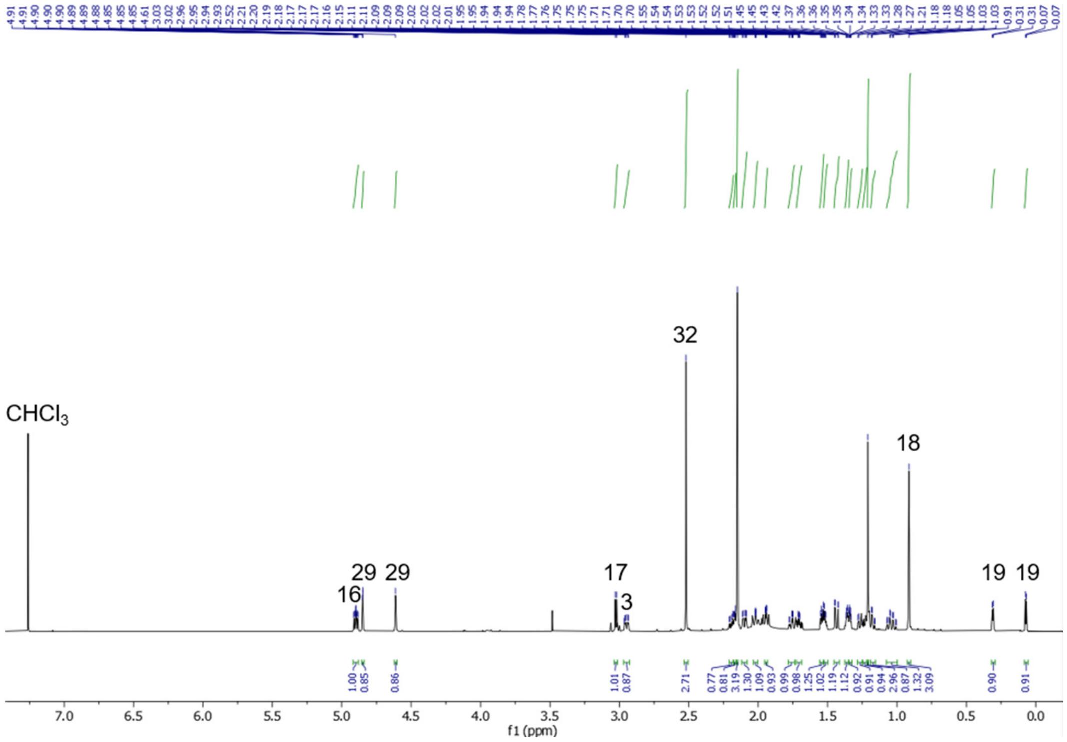

Figure S94. ^1H NMR spectrum of Cyclomicrobuxinine (**17**) ($CDCl_3$, 600 MHz). The assignment of the signals between 1.0 and 2.2 ppm can be found in the enlarged Figure S95.

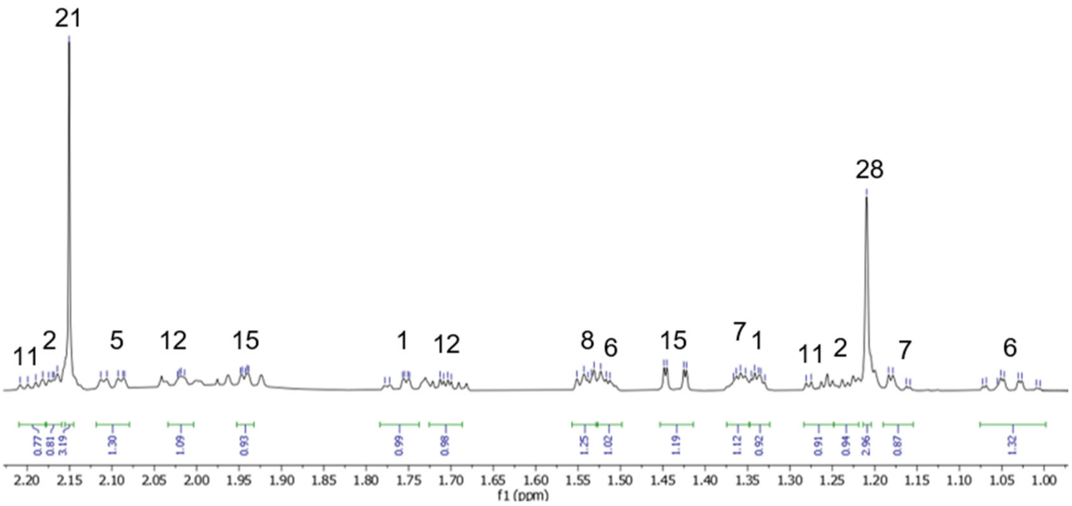

Figure S95. Detail of the ^1H NMR spectrum of Cyclomicrobuxinine (**17**) ($CDCl_3$, 600 MHz).

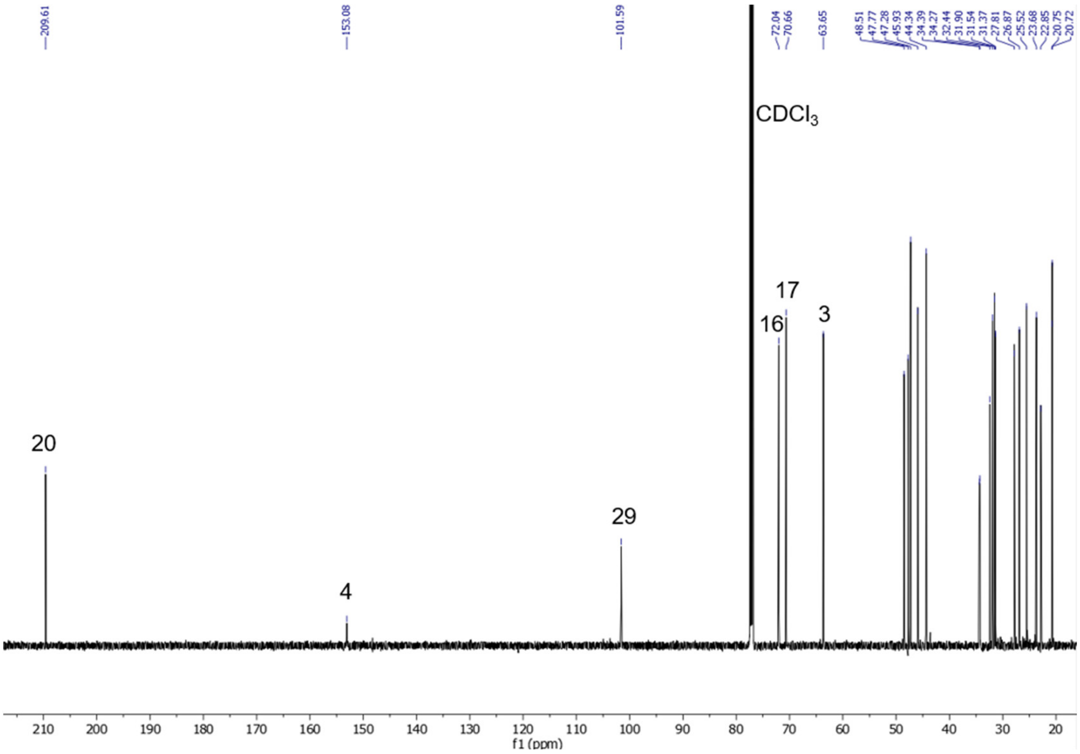

Figure S96. ^{13}C NMR spectrum of Cyclomicrobuxinine (**17**) ($CDCl_3$, 150 MHz). The assignment of the signals between 20 and 49 ppm can be found in the enlarged Figure S97.

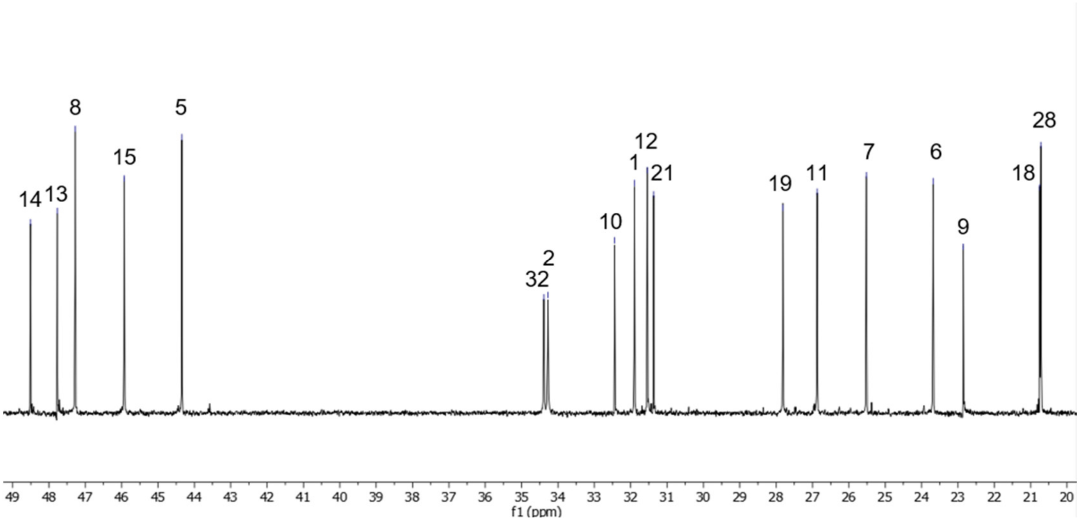

Figure S97. Detail of the ^{13}C NMR spectrum of Cyclomicrobuxinine (**17**) ($CDCl_3$, 150 MHz).

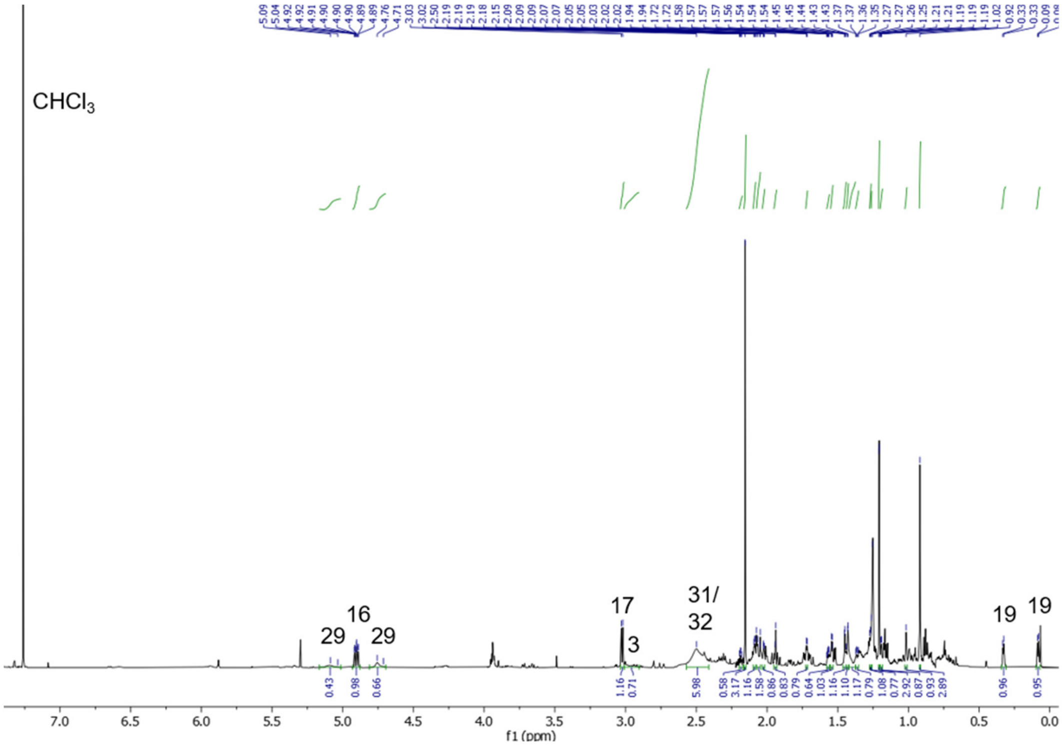

Figure S98. ^{1}H NMR spectrum of Cyclomicrobuxine (**18**) ($CDCl_3$, 600 MHz). The assignment of the signals between 0.9 and 2.2 ppm can be found in the enlarged Figure S99.

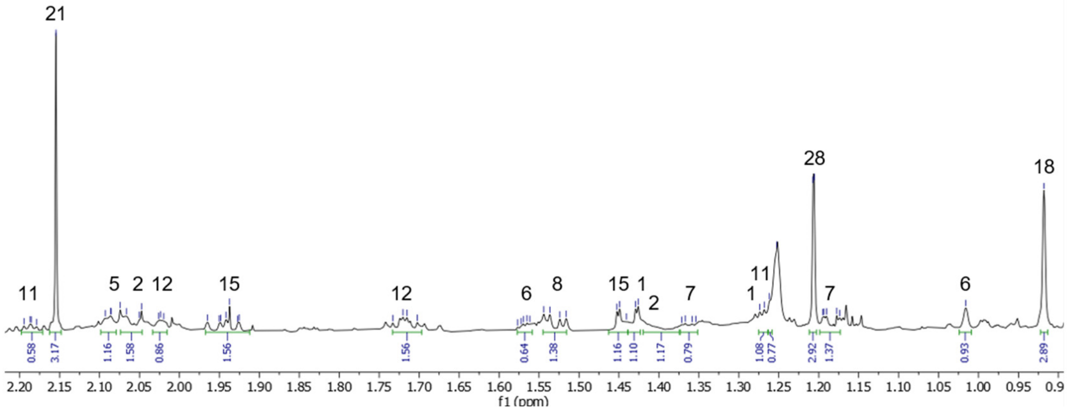

Figure S99. Detail of the ^{1}H NMR spectrum of Cyclomicrobuxine (**18**) ($CDCl_3$, 600 MHz).

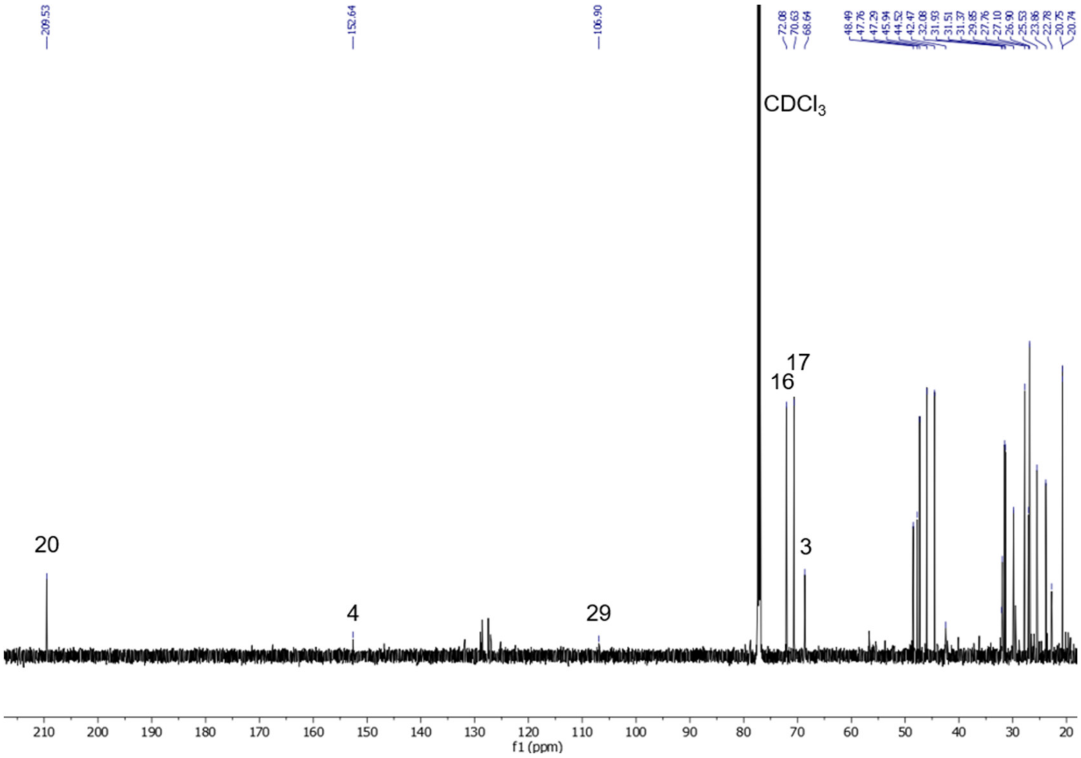

Figure S100. ^{13}C NMR spectrum of Cyclomicrobuxine (**18**) ($CDCl_3$, 150 MHz). The assignment of the signals between 20.5 and 49 ppm can be found in the enlarged Figure S101.

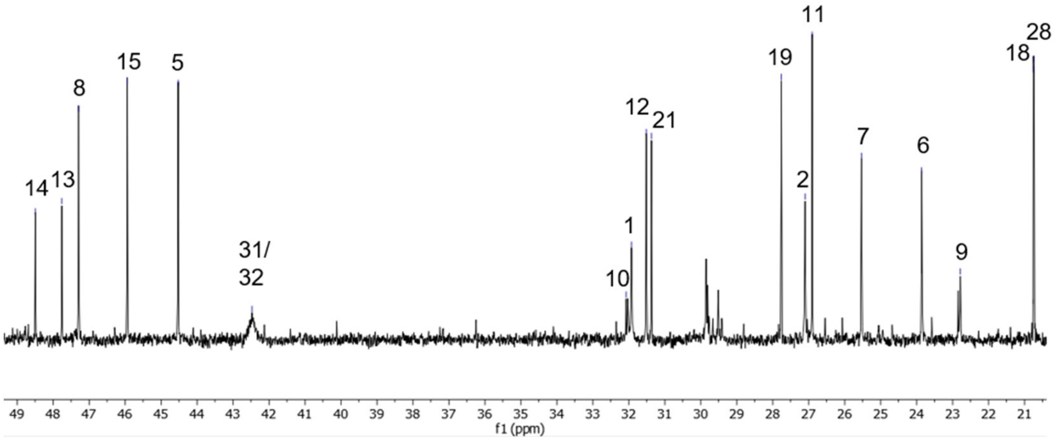

Figure S101. Detail of the ^{13}C NMR spectrum of Cyclomicrobuxine (**18**) ($CDCl_3$, 150 MHz).

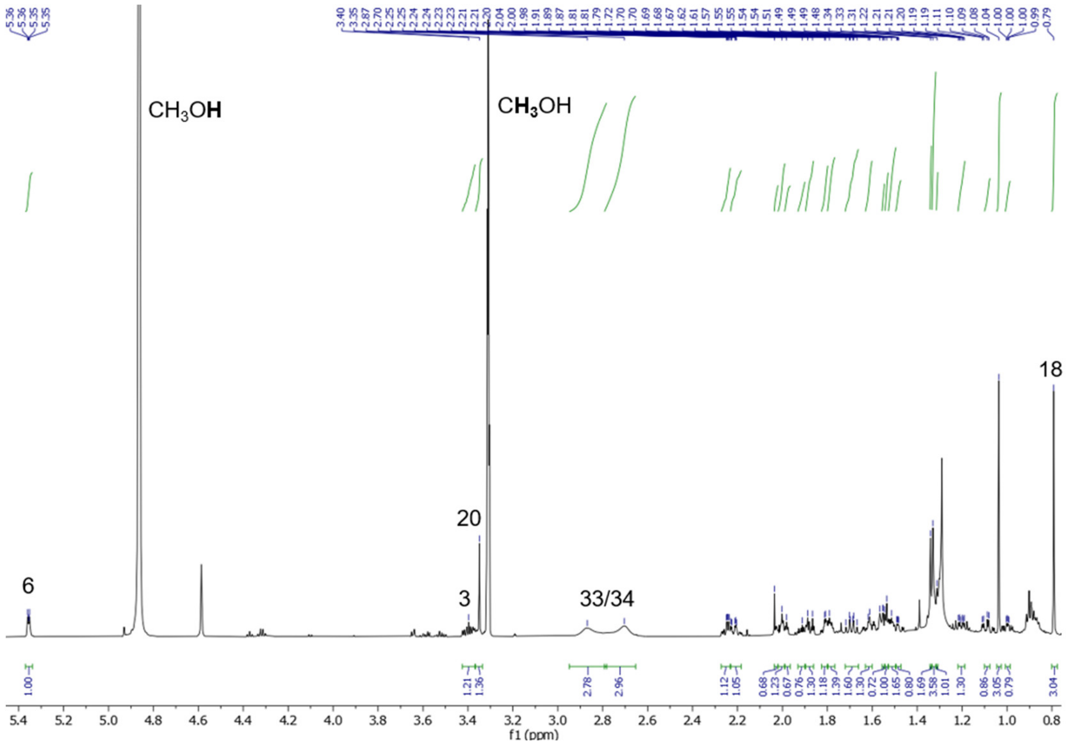

Figure S102. ^{1}H NMR spectrum of Irehine (**19**) (CD_3OD, 600 MHz). The assignment of the signals between 0.95 and 2.3 ppm can be found in the enlarged Figure S103.

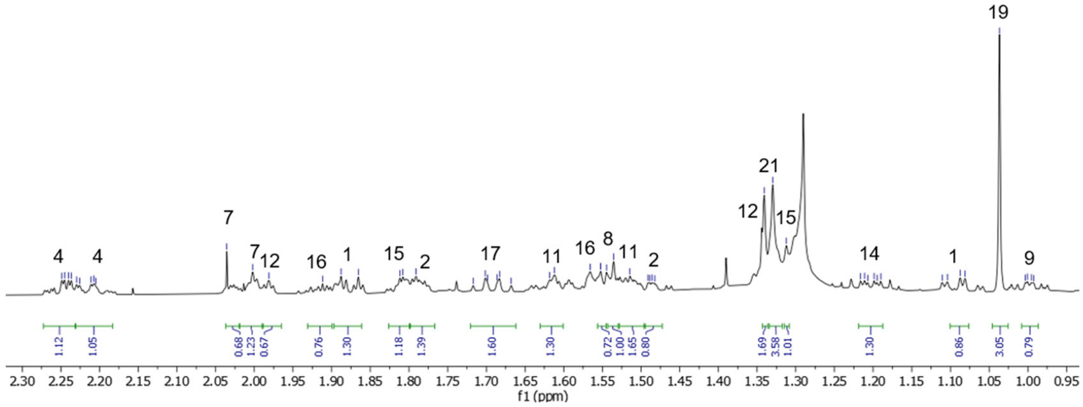

Figure S103. Detail of the ^{1}H NMR spectrum of Irehine (**19**) (CD_3OD, 600 MHz).

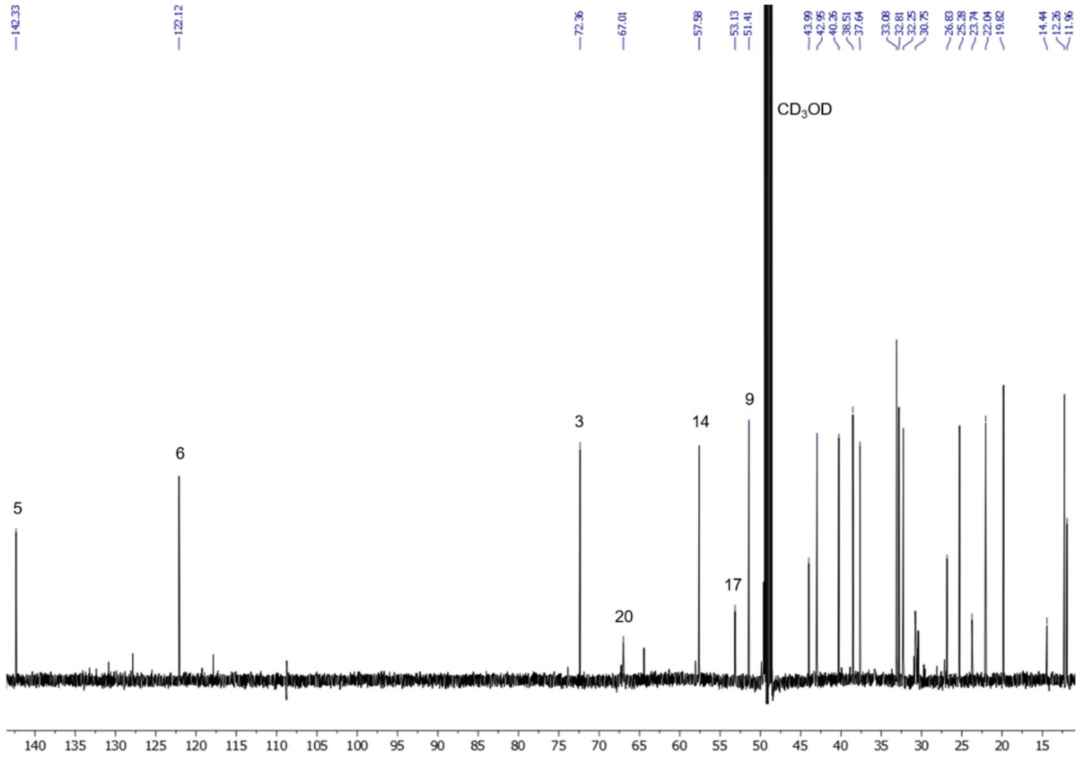

Figure S104. ^{13}C NMR spectrum of Irehine (**19**) (CD_3OD, 150 MHz). The assignment of the signals between 11 and 44 ppm can be found in the enlarged Figure S105.

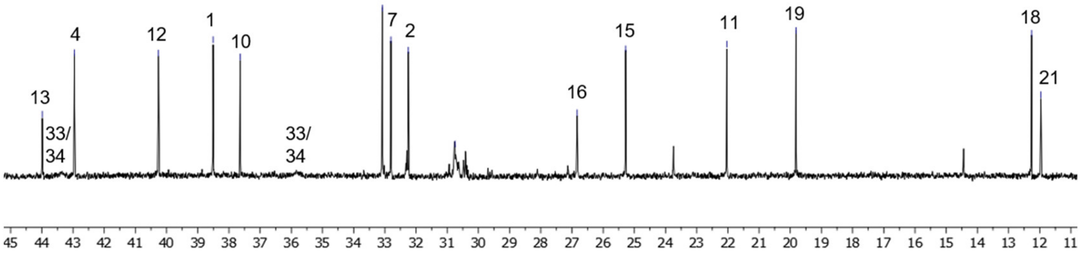

Figure S105. Detail of the ^{13}C NMR spectrum of Irehine (**19**) (CD_3OD, 150 MHz).

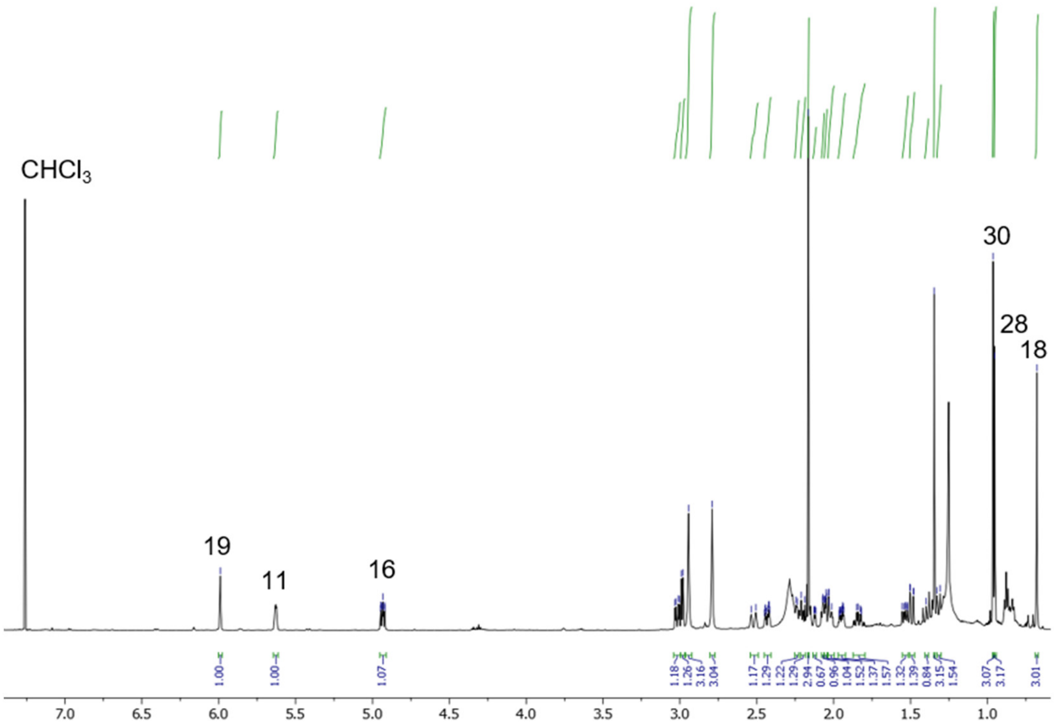

Figure S106. ^{1}H NMR spectrum of 16-α-hydroxybuxaminone (**20**) ($CDCl_3$, 600 MHz). The assignment of the signals between 1.3 and 3.0 ppm can be found in the enlarged Figure S107.

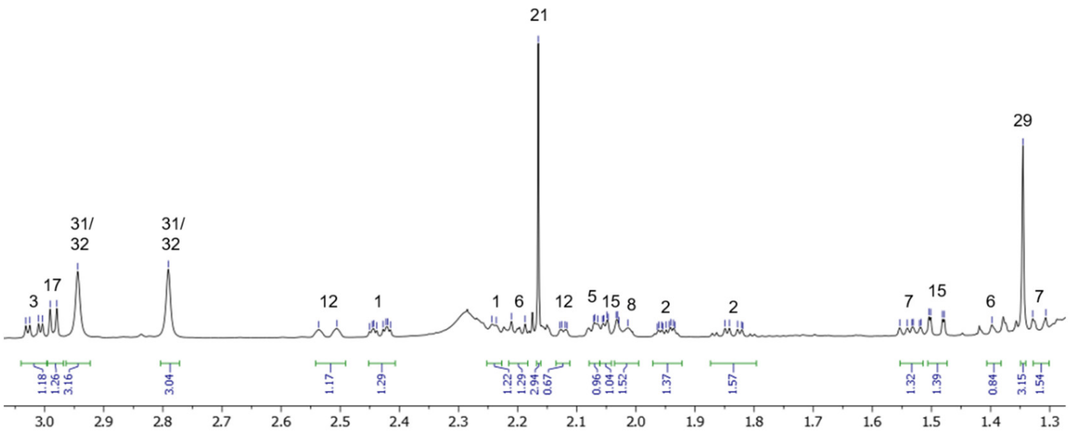

Figure S107. Detail of the ^{1}H NMR spectrum of 16-α-hydroxybuxaminone (**20**) ($CDCl_3$, 600 MHz).

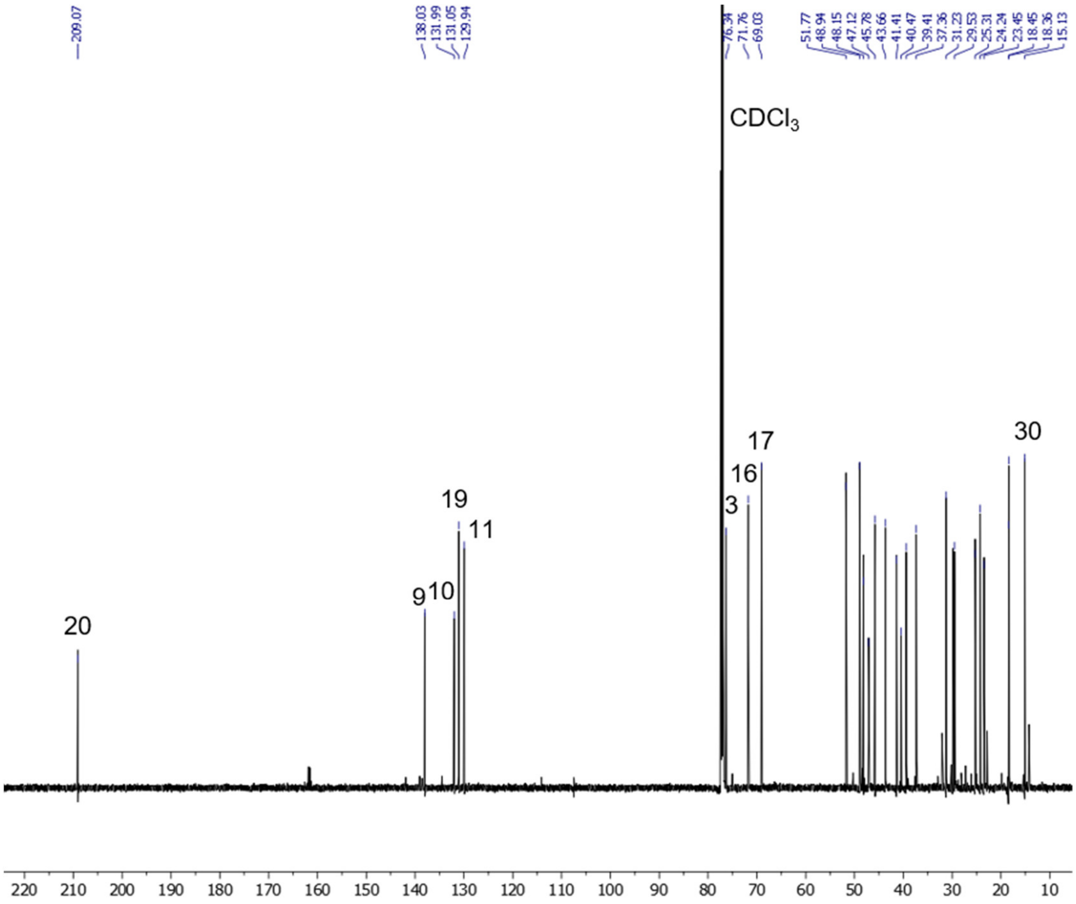

Figure S108. ^{13}C NMR spectrum of 16-α-hydroxybuxaminone (**20**) ($CDCl_3$, 150 MHz). The assignment of the signals between 18 and 52 ppm can be found in the enlarged Figure S109.

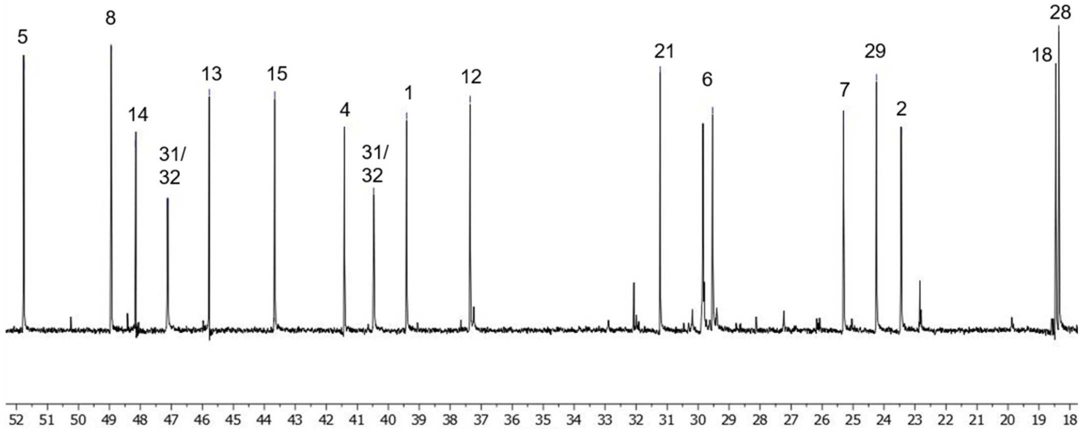

Figure S109. Detail of the ^{13}C NMR spectrum of 16-α-hydroxybuxaminone (**20**) ($CDCl_3$, 150 MHz).

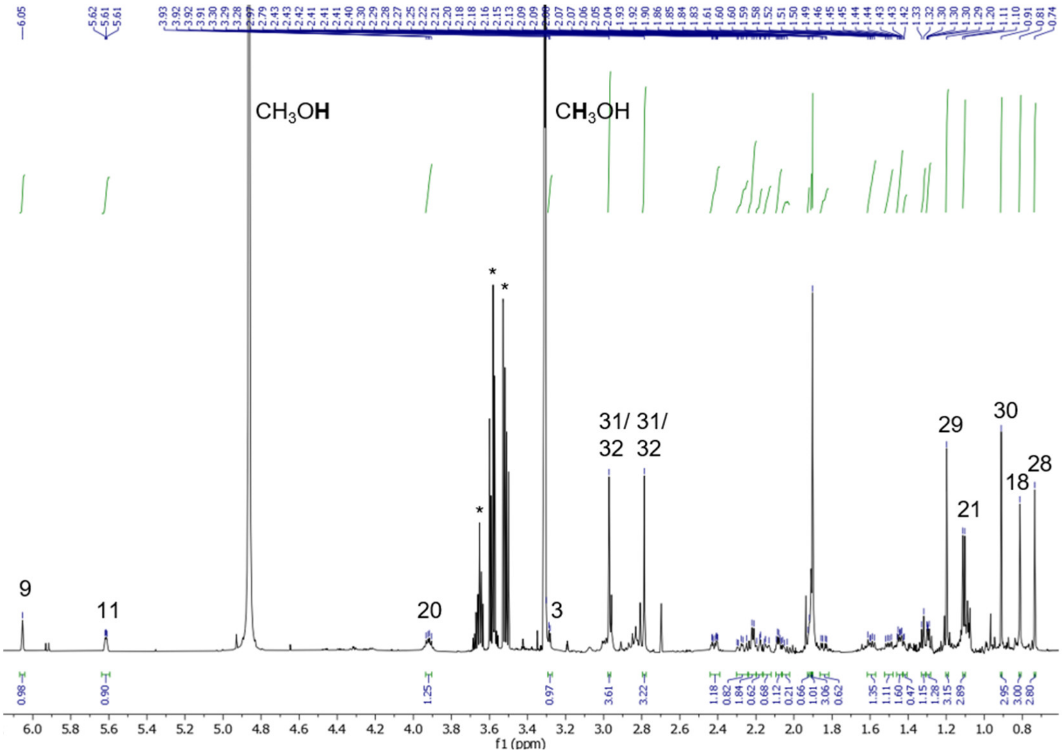

Figure S110. ^{1}H NMR spectrum of N20-acetylbuxamine-E (**21**) (CD3OD, 600 MHz). The assignment of the signals between 1.25 and 2.45 ppm can be found in the enlarged Figure S111 (*signals of 36.4% glycerol).

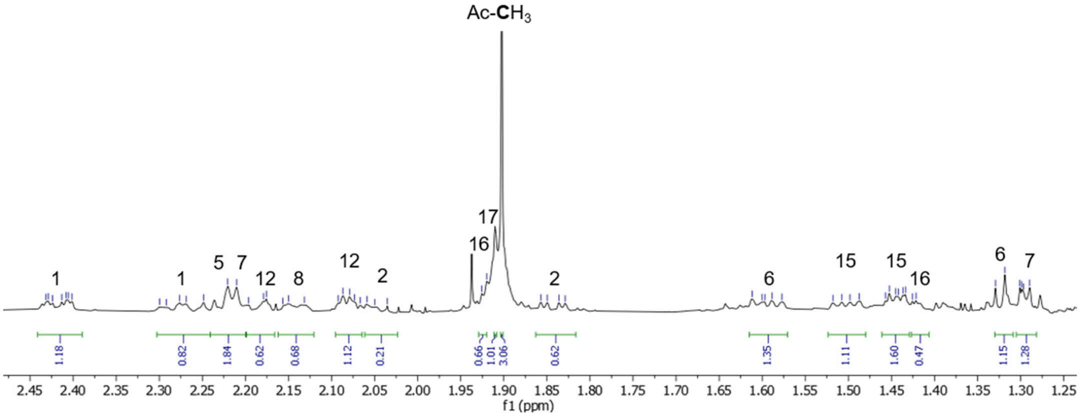

Figure S111. Detail of the ^{1}H NMR spectrum of N20-acetylbuxamine-E (**21**) (CD3OD, 600 MHz).

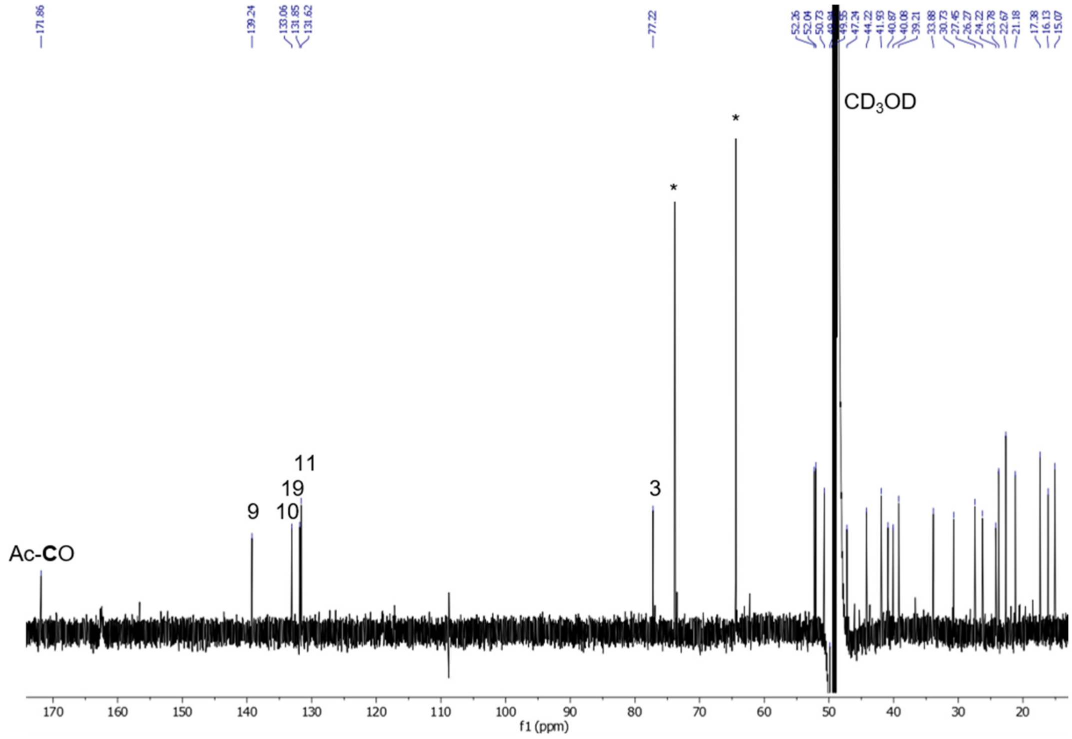

Figure S112. ^{13}C NMR spectrum of N_{20}-acetylbuxamine-E (**21**) (CD_3OD, 150 MHz). The assignment of the signals between 15 and 53 ppm can be found in the enlarged Figure S113 (*signals of 36.4% glycerol).

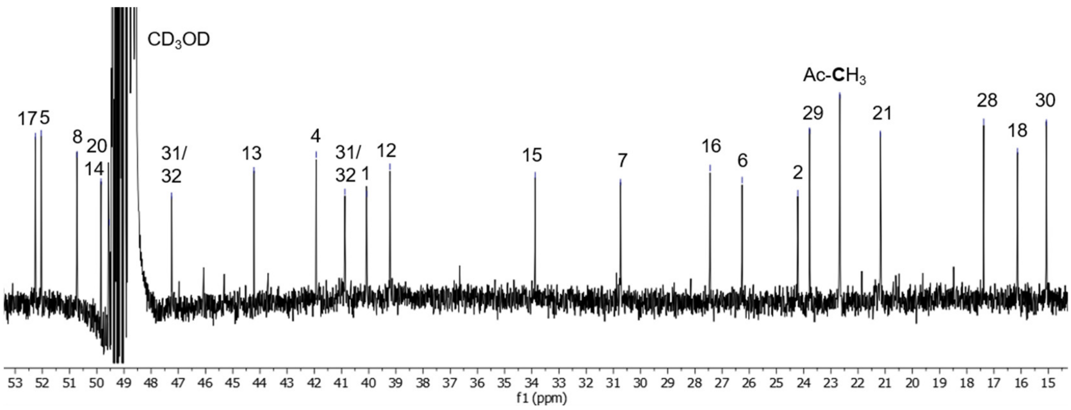

Figure S113. Detail of the ^{13}C NMR spectrum of N_{20}-acetylbuxamine-E (**21**) (CD_3OD, 150 MHz).

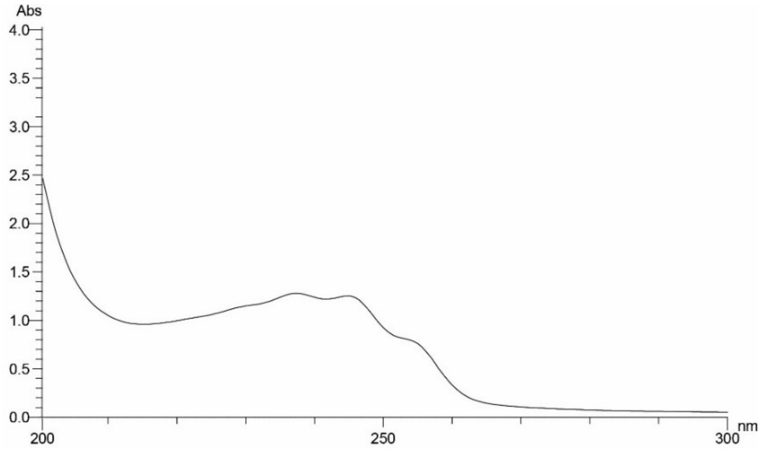

Figure S114. UV spectrum of N-benzoyl-O-acetylbuxodienine-E (**22**) (c = 0.02 mg/mL) in methanol; λ_{max} (log ε): 237 nm (4.54), 245 nm (4.53), 253 nm (4.35).

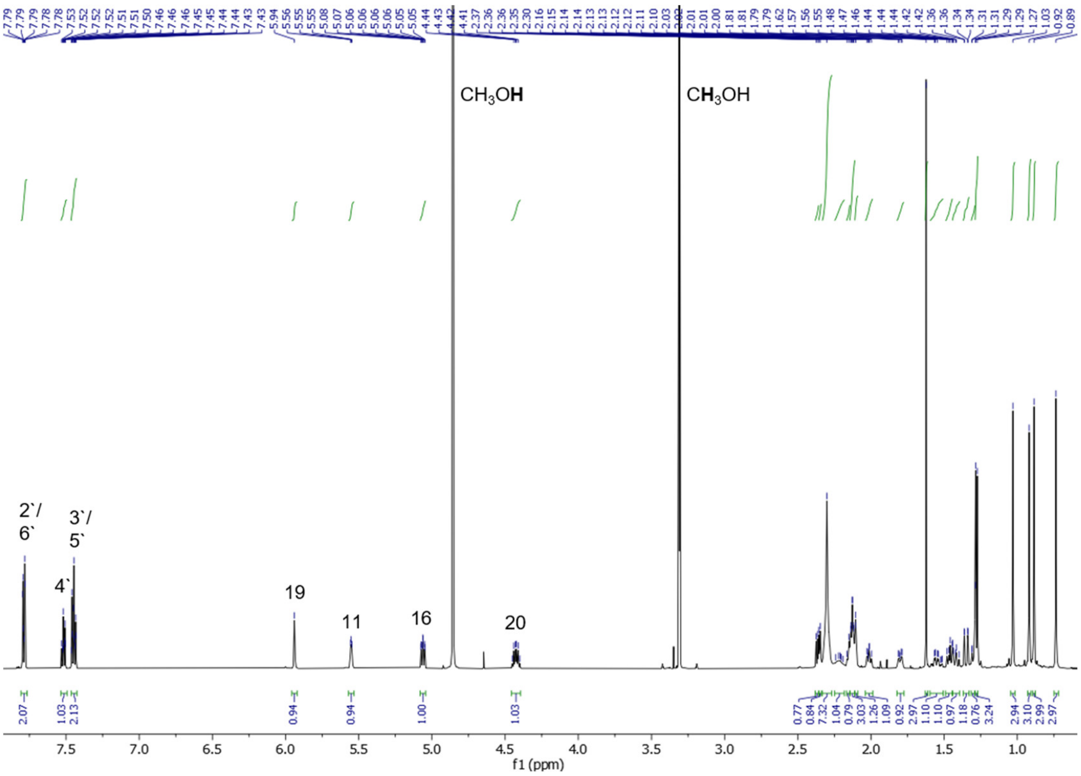

Figure S115. ^{1}H NMR spectrum of N-benzoyl-O-acetylbuxodienine-E (**22**) (CD_3OD, 600 MHz). The assignment of the signals between 0.6 and 2.5 ppm can be found in the enlarged Figure S116.

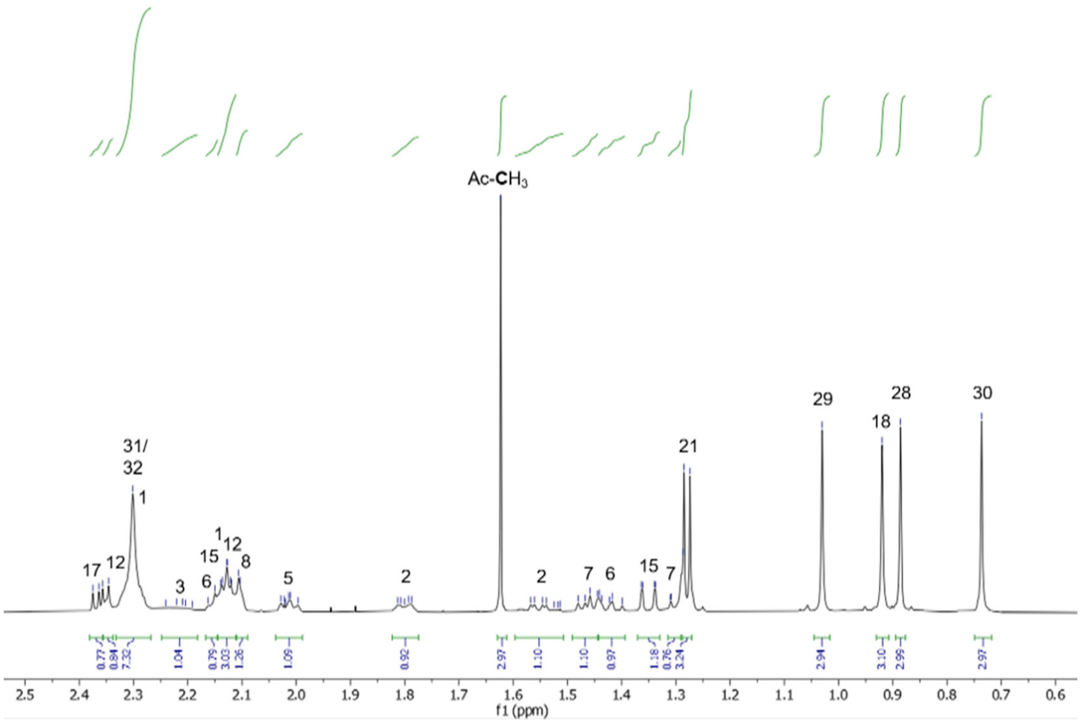

Figure S116. Detail of the ^{1}H NMR spectrum of N-benzoyl-O-acetylbuxodienine-E (**22**) (CD_3OD, 600 MHz).

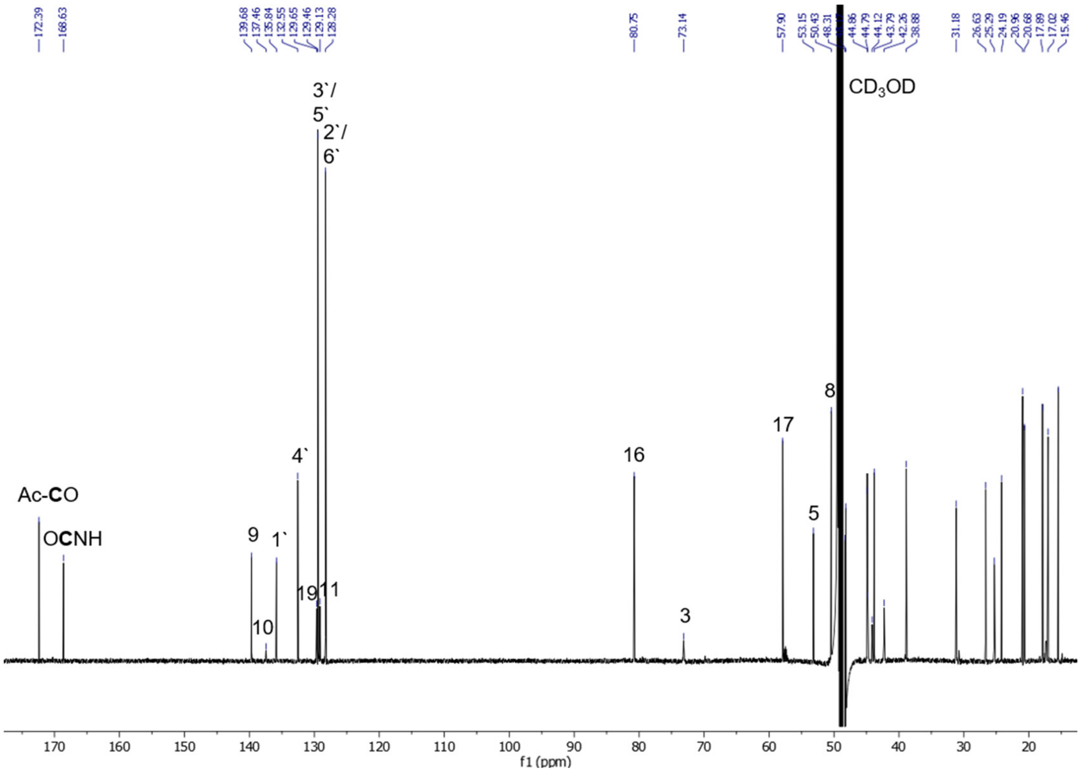

Figure S117. ^{13}C NMR spectrum of N-benzoyl-O-acetylbuxodienine-E (**22**) (CD_3OD, 150 MHz). The assignment of the signals between 15 and 49 ppm can be found in the enlarged Figure S118.

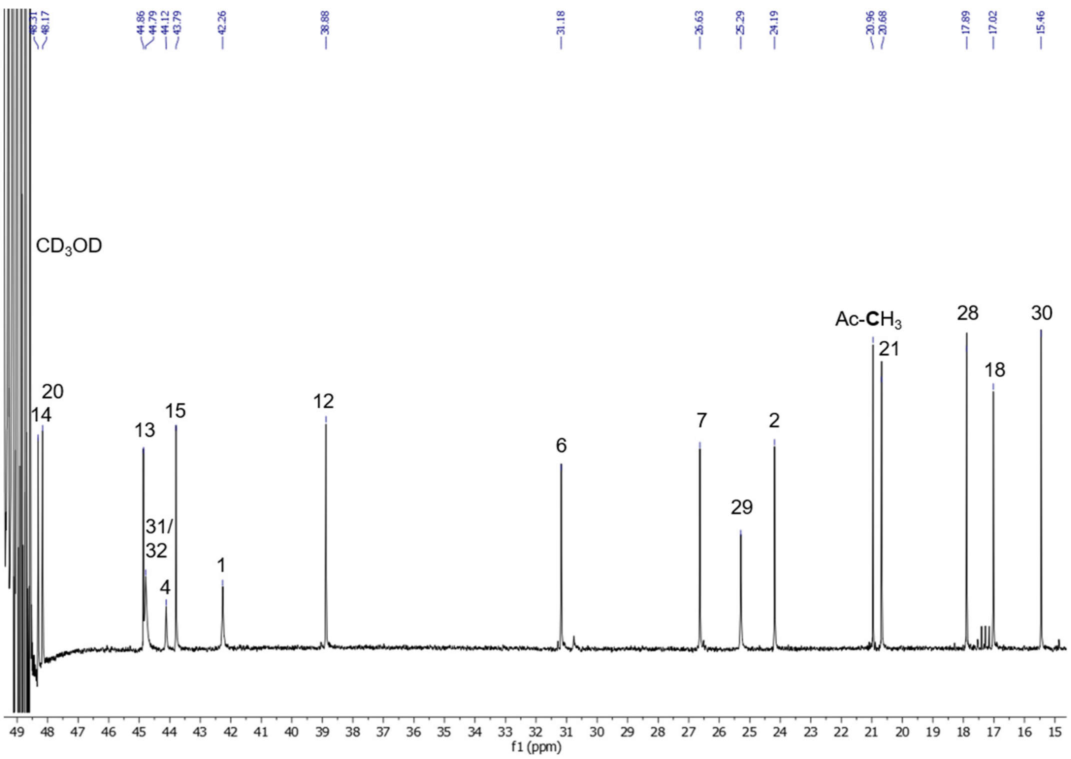

Figure S118. Detail of the ^{13}C NMR spectrum of N-benzoyl-O-acetylbuxodienine-E (**22**) (CD_3OD, 150 MHz).

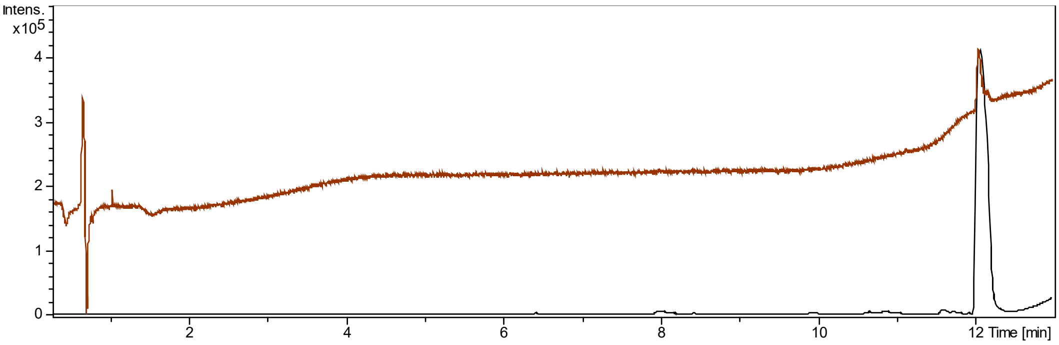

Figure S119. UHPLC/+ESI-QqTOF-MS/MS chromatogram of N-benzoyl-O-acetylbuxadine-E (**23**). Base peak chromatogram 200.0000-1000.0000 +All MS (black); UV-Chromatogramm, 200-400 nm (red).

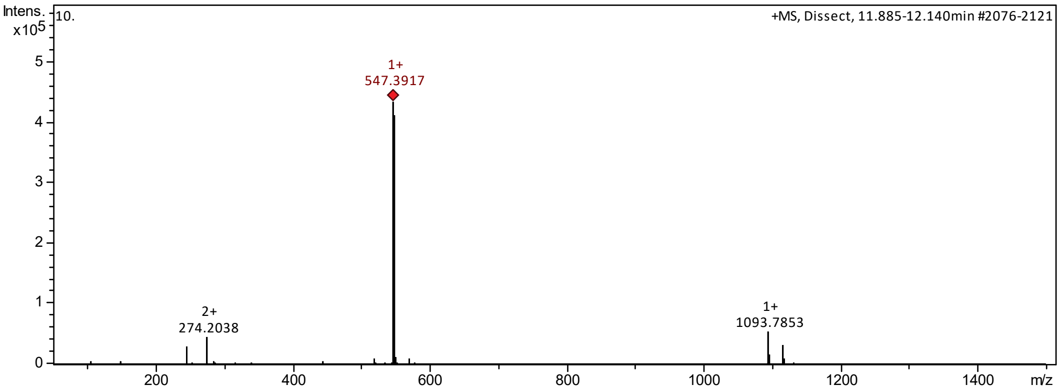

Figure S120. +ESI-QqTOF MS spectrum of N-benzoyl-O-acetylbuxadine-E (**23**); *m/z* 274.2038 $[M+2H]^{2+}$, 547.3917 $[M+H]^{+}$ and 1093.7853 $[2M+H]^{+}$.

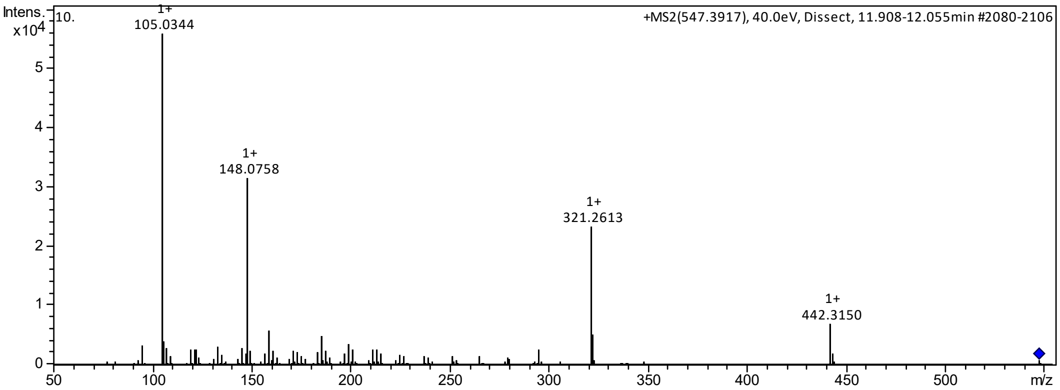

Figure S121. +ESI-QqTOF MS/MS spectrum of N-benzoyl-O-acetylbuxadine-E (**23**).

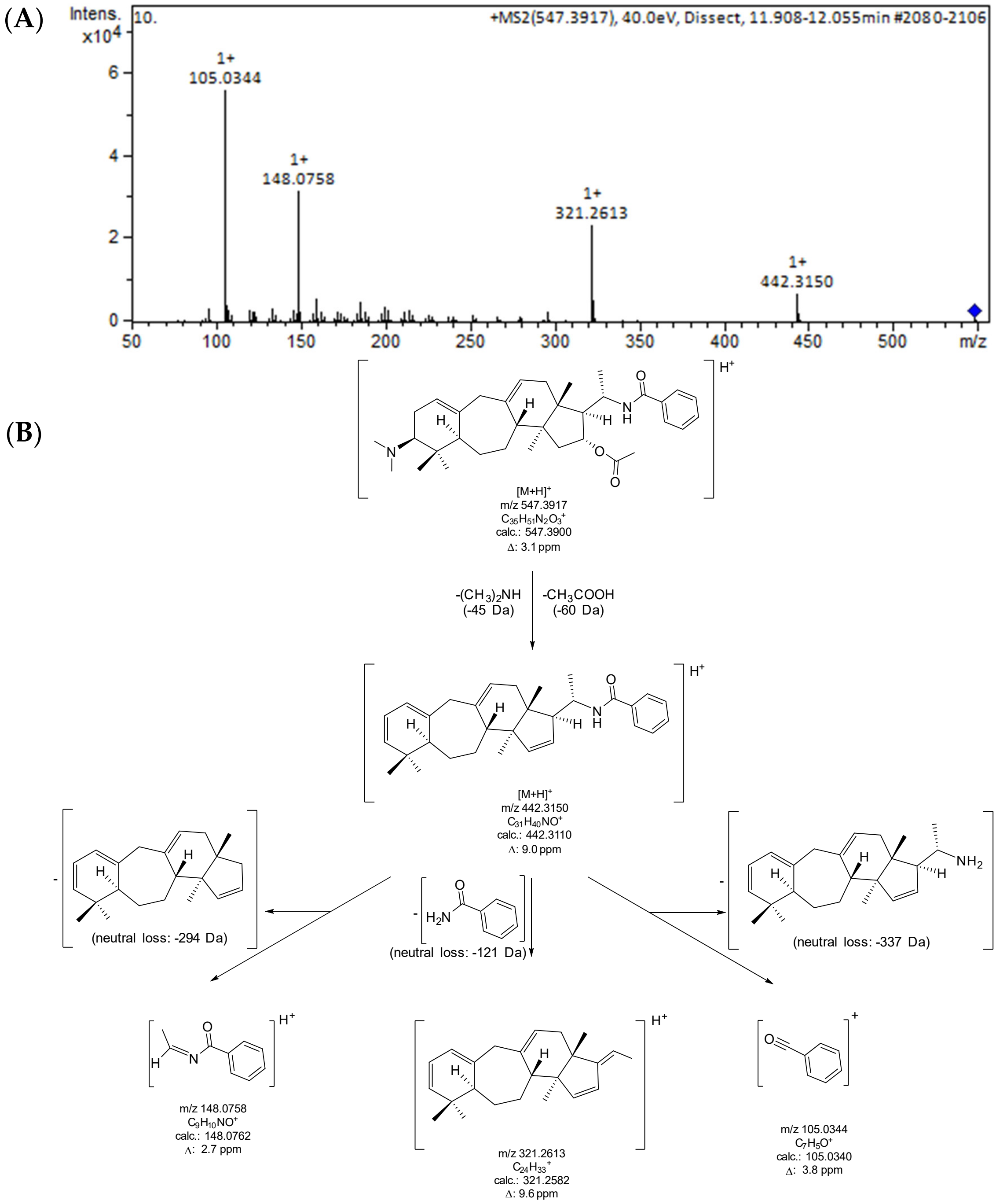

Figure S122. (**A**) +ESI MS/MS spectrum (CID 40 eV). (**B**) fragments and possible fragmentation pathway of the $[M+H]^+$ ion of N-benzoyl-O-acetylbuxadine-E (**23**).

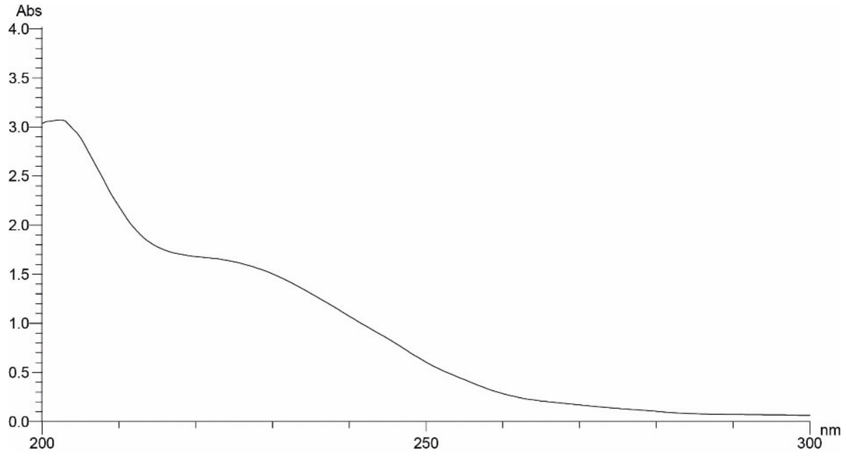

Figure S123. UV spectrum of N-benzoyl-O-acetylbuxadine-E (**23**) (c = 0.086 mg/mL) in methanol; λ_{max} (log ε): 225 nm (4.01).

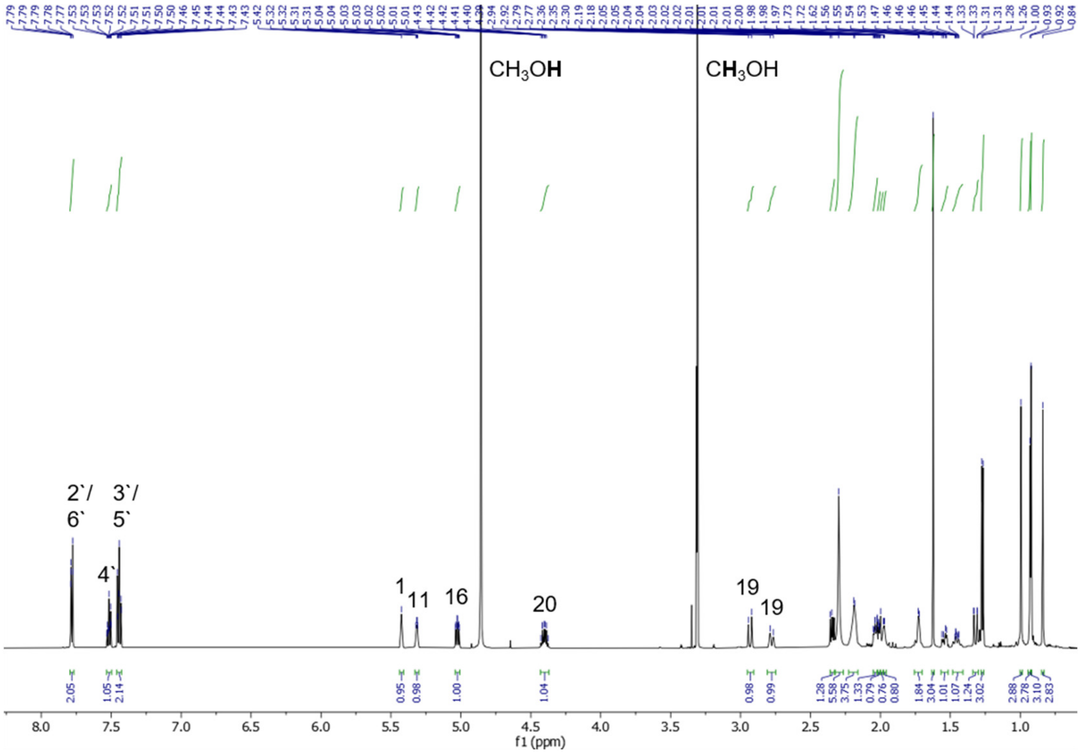

Figure S124. ^{1}H NMR spectrum of N-benzoyl-O-acetylbuxadine-E (**23**) (CD_3OD, 600 MHz). The assignment of the signals between 0.8 and 2.4 ppm can be found in the enlarged Figure S125.

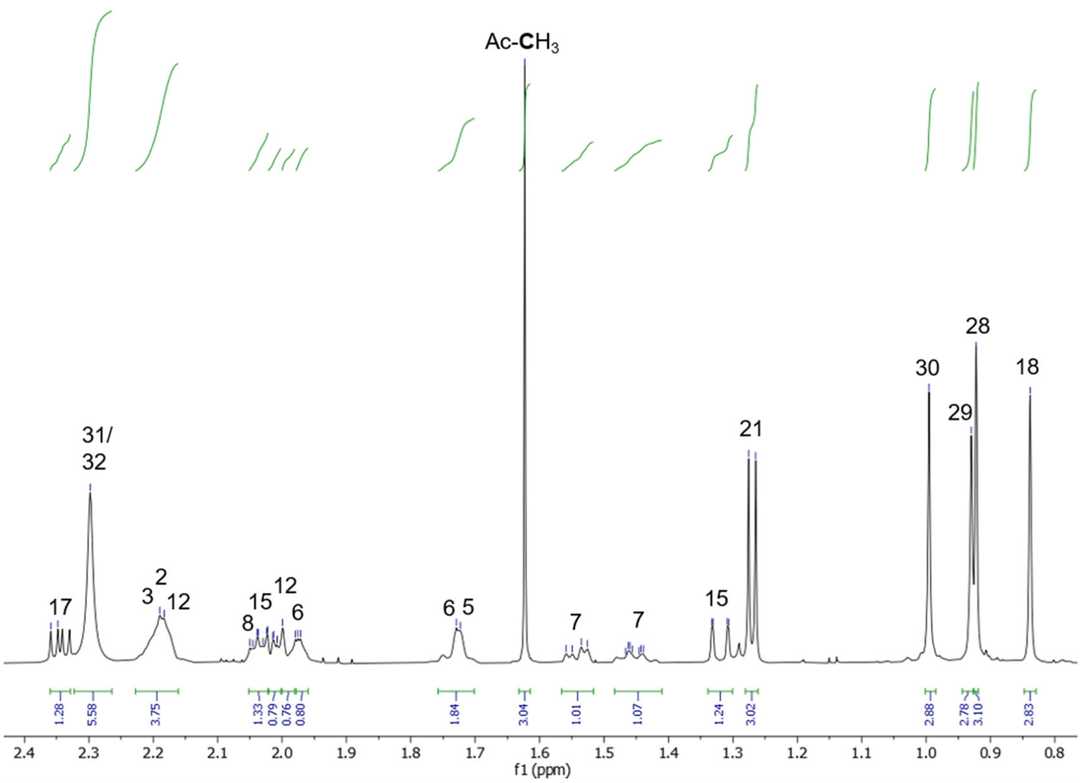

Figure S125. Detail of the ^{1}H NMR spectrum of N-benzoyl-O-acetylbuxadine-E (**23**) (CD_3OD, 600 MHz).

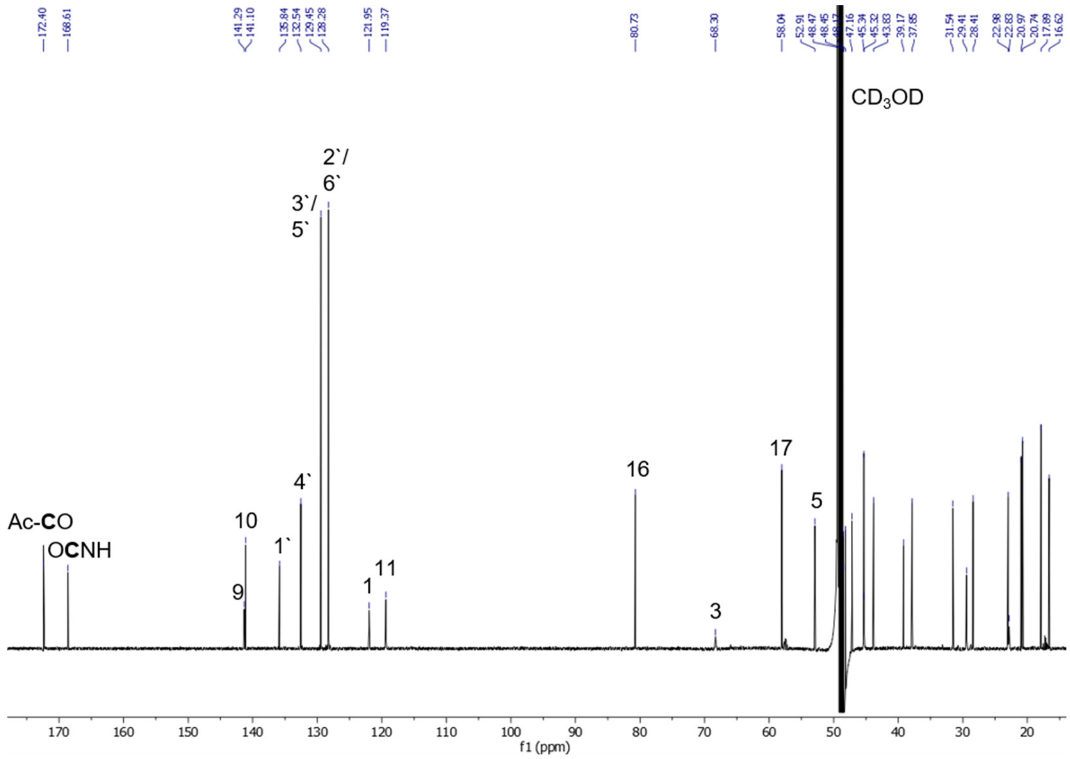

Figure S126. ^{13}C NMR spectrum of N-benzoyl-O-acetylbuxadine-E (**23**) (CD_3OD, 150 MHz). The assignment of the signals between 16 and 49 ppm can be found in the enlarged Figure S127.

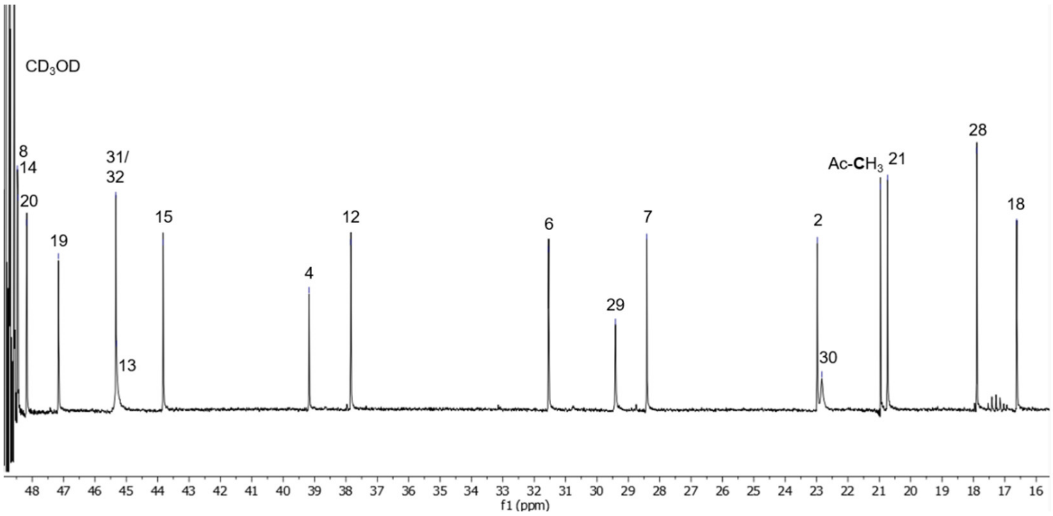

Figure S127. Detail of the ^{13}C NMR spectrum of N-benzoyl-O-acetylbuxadine-E (**23**) (CD_3OD, 150 MHz).

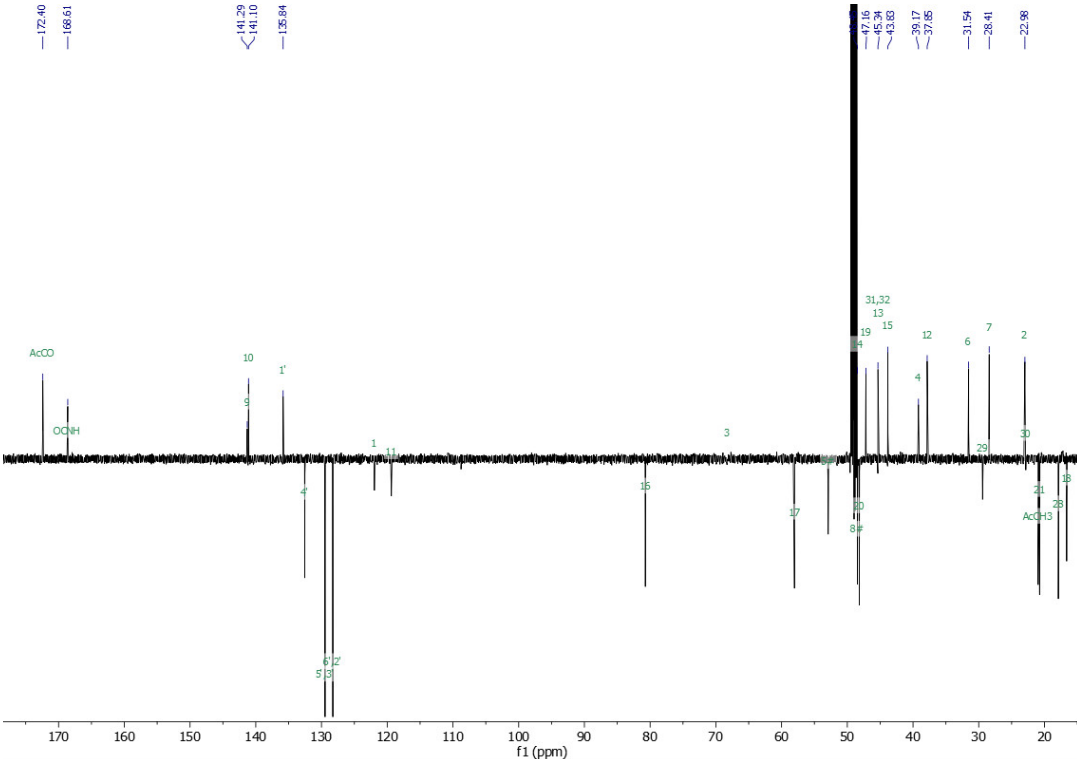

Figure S128. ^{13}C APT spectrum of N-benzoyl-O-acetylbuxadine-E (**23**) (CD_3OD, 150 MHz).

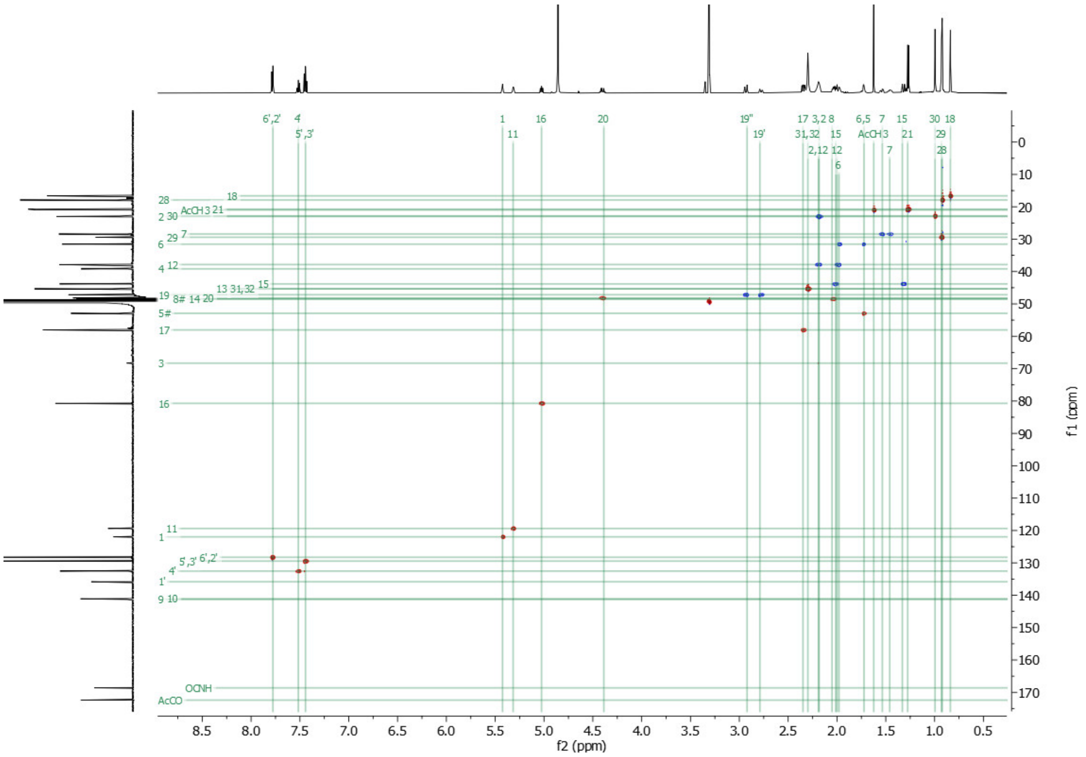

Figure S129. $^{1}H/^{13}C$ HSQC spectrum of N-benzoyl-O-acetylbuxadine-E (**23**) (CD_3OD, 600/150 MHz).

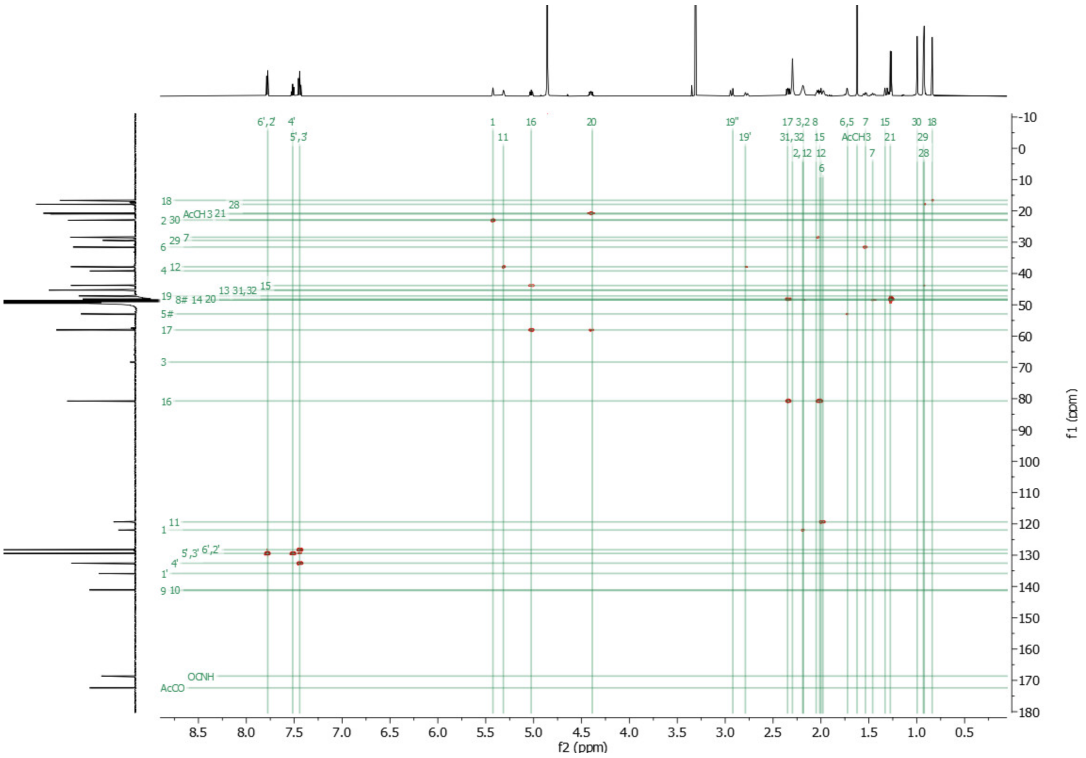

Figure S130. $^{1}H/^{13}C$ H2BC spectrum of N-benzoyl-O-acetylbuxadine-E (**23**) (CD_3OD, 600/150 MHz).

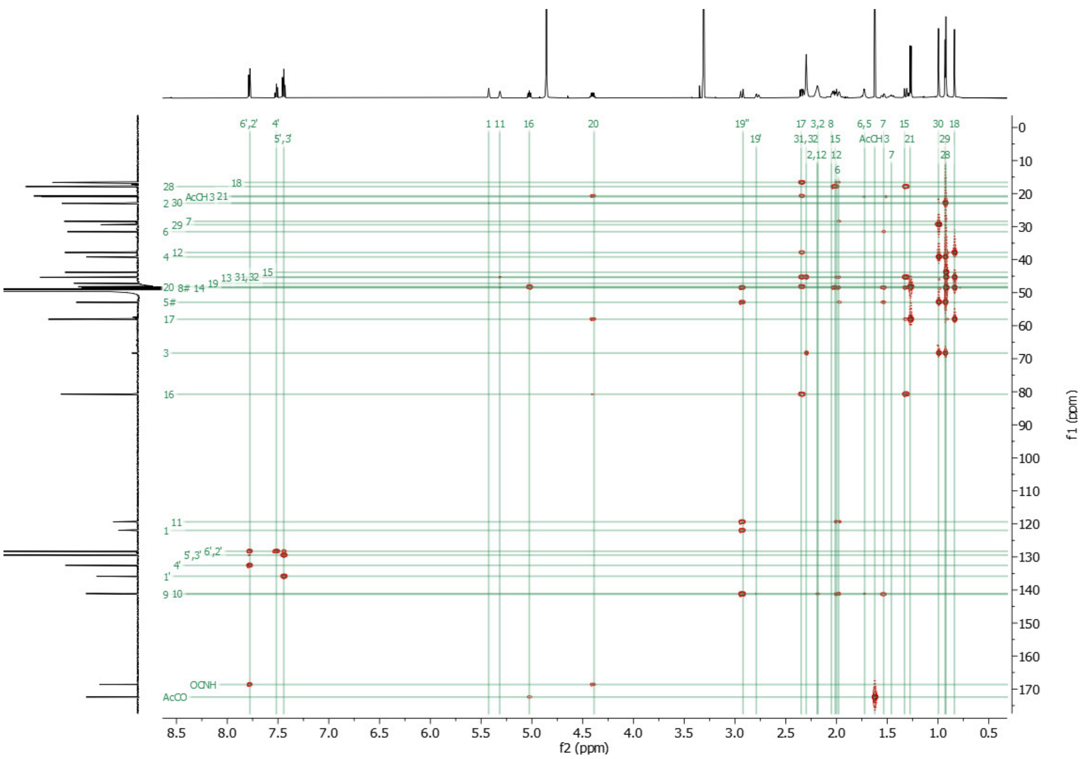

Figure S131. $^{1}H/^{13}C$ HMBC spectrum of N-benzoyl-O-acetylbuxadine-E (**23**) (CD_3OD, 600/150 MHz).

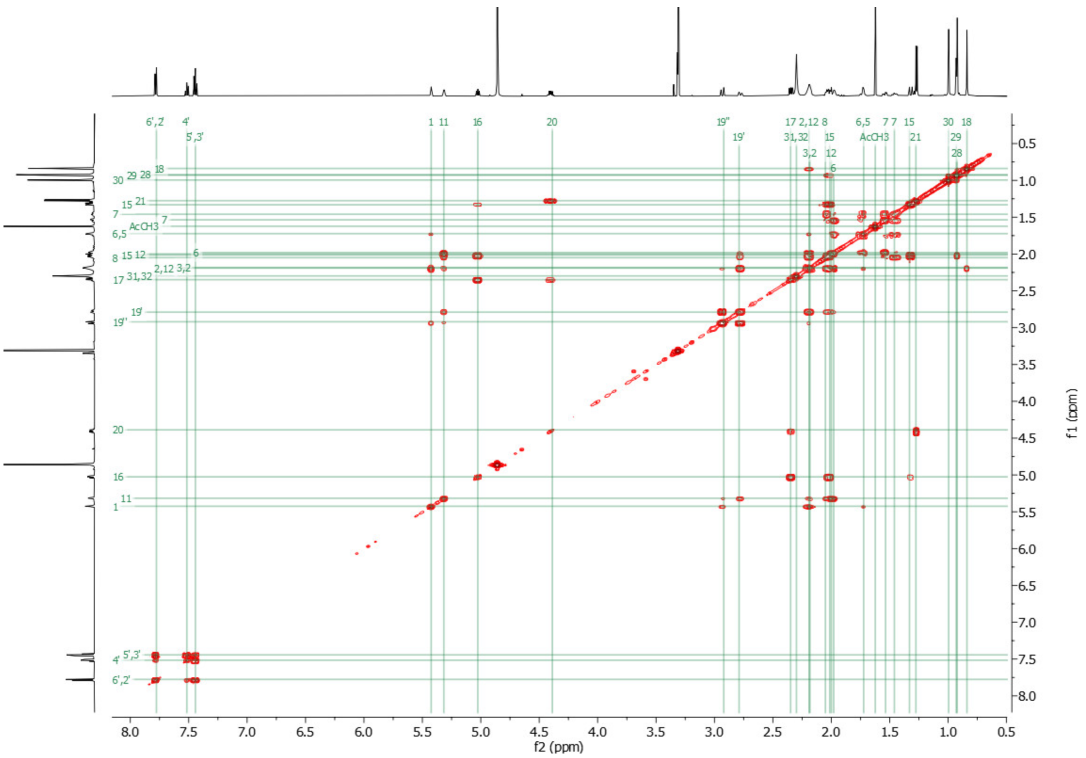

Figure S132. $^{1}H/^{1}H$ COSY spectrum of N-benzoyl-O-acetylbuxadine-E (**23**) (CD_3OD, 600 MHz).

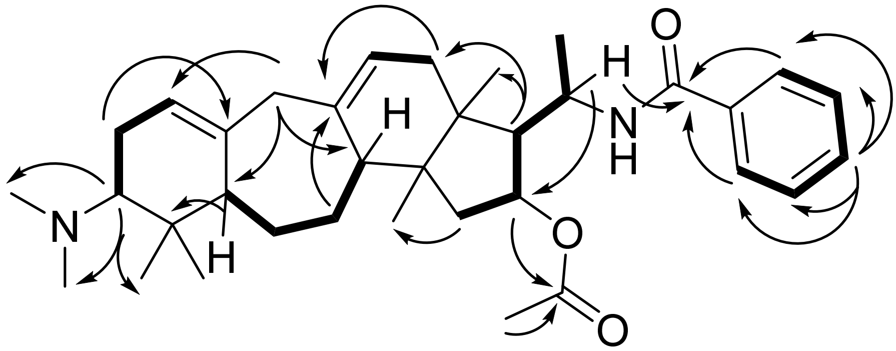

Figure S133. Key COSY (bold lines) and HMBC (arrows) correlations of N-benzoyl-O-acetylbuxadine-E (**23**).

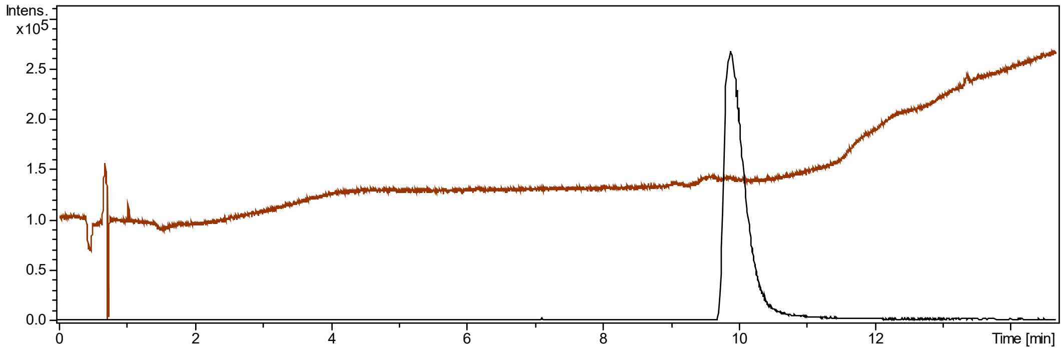

Figure S134. UHPLC/+ESI-QqTOF-MS/MS chromatogram of N20-acetylbuxadine-G (**24**). Base peak chromatogram 200.0000-1000.0000 +All MS (black); UV-Chromatogramm, 200-400 nm (red).

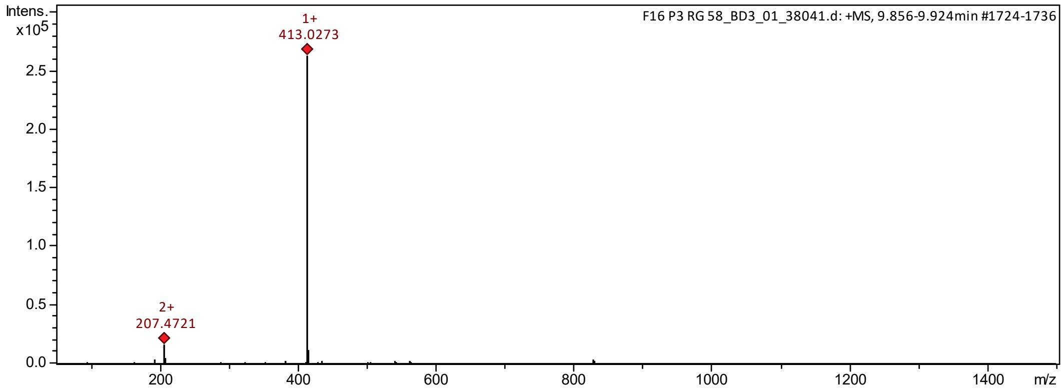

Figure S135. +ESI-QqTOF MS spectrum of N20-acetylbuxadine-G (**24**); *m/z* 207.4721 $[M+2H]^{2+}$ and 413.0273 $[M+H]^{+}$.

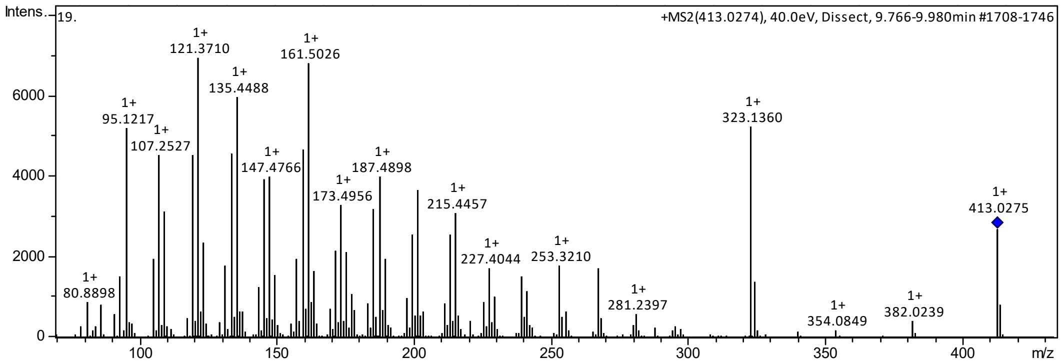

Figure S136. +ESI-QqTOF MS/MS spectrum of N20-acetylbuxadine-G (**24**).

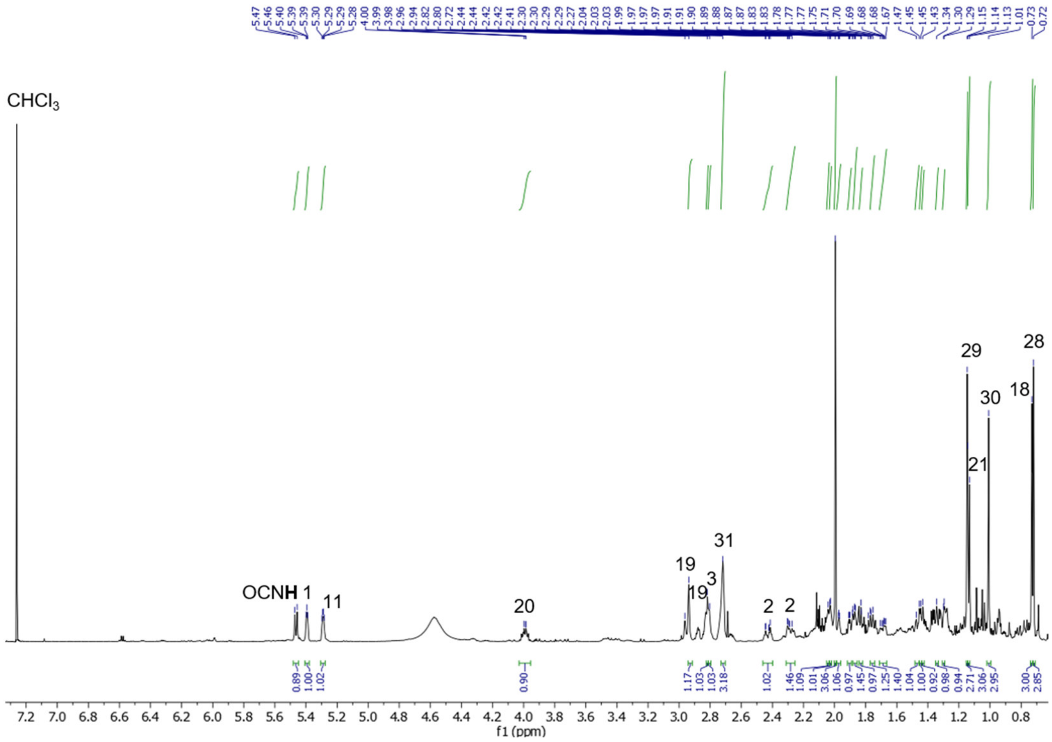

Figure S137. ^{1}H NMR spectrum of N20-acetylbuxadine-G (**24**) ($CDCl_3$, 600 MHz). The assignment of the signals between 1.25 and 2.05 ppm can be found in the enlarged Figure S138.

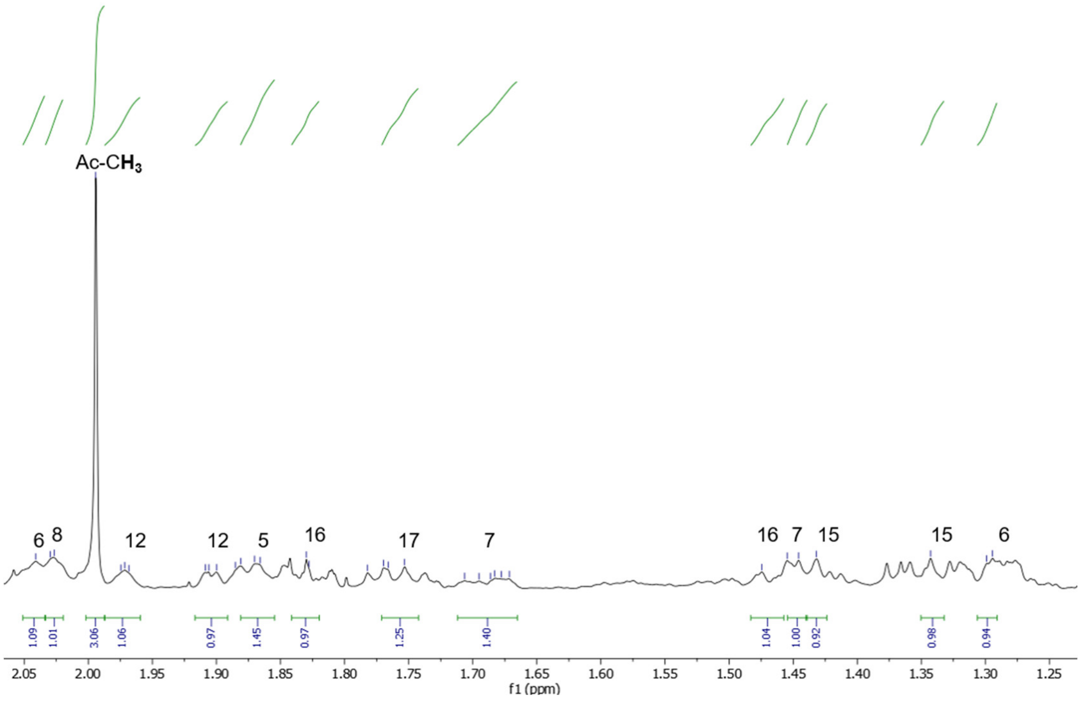

Figure S138. Detail of the ^{1}H NMR spectrum of N20-acetylbuxadine-G (**24**) ($CDCl_3$, 600 MHz).

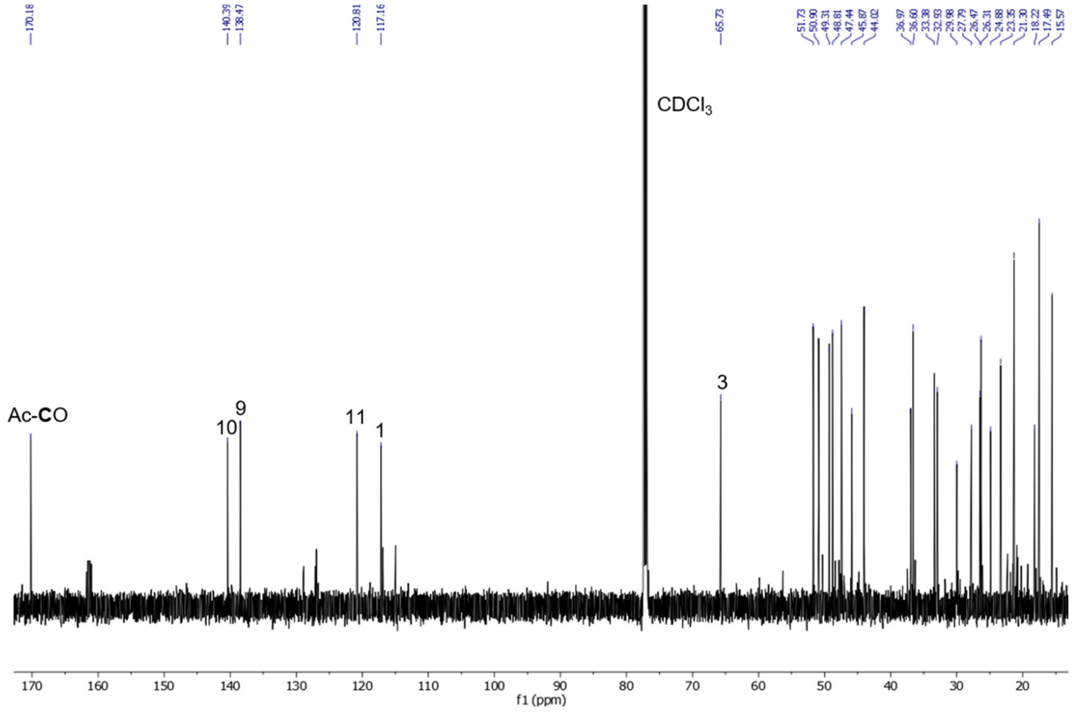

Figure S139. ^{13}C NMR spectrum of N20-acetylbuxadine-G (**24**) ($CDCl_3$, 150 MHz). The assignment of the signals between 15 and 52 ppm can be found in the enlarged Figure S140.

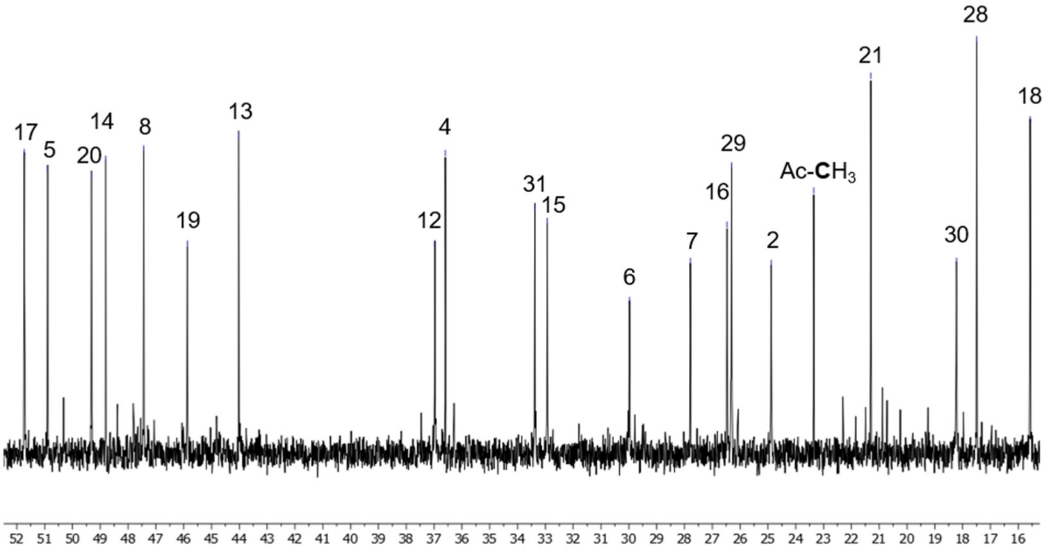

Figure S140. Detail of the ^{13}C NMR spectrum of N$_{20}$-acetylbuxadine-G (**24**) (CDCl$_3$, 150 MHz).

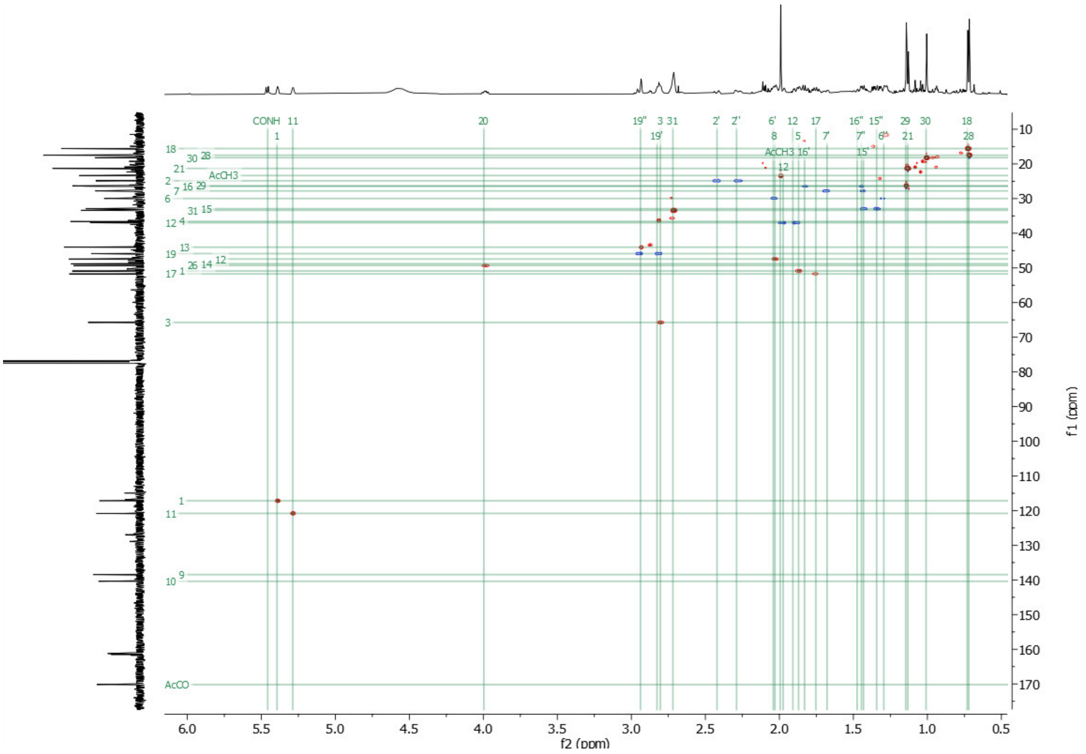

Figure S141. ^{1}H/^{13}C HSQC spectrum of N$_{20}$-acetylbuxadine-G (**24**) (CDCl$_3$, 600/150 MHz).

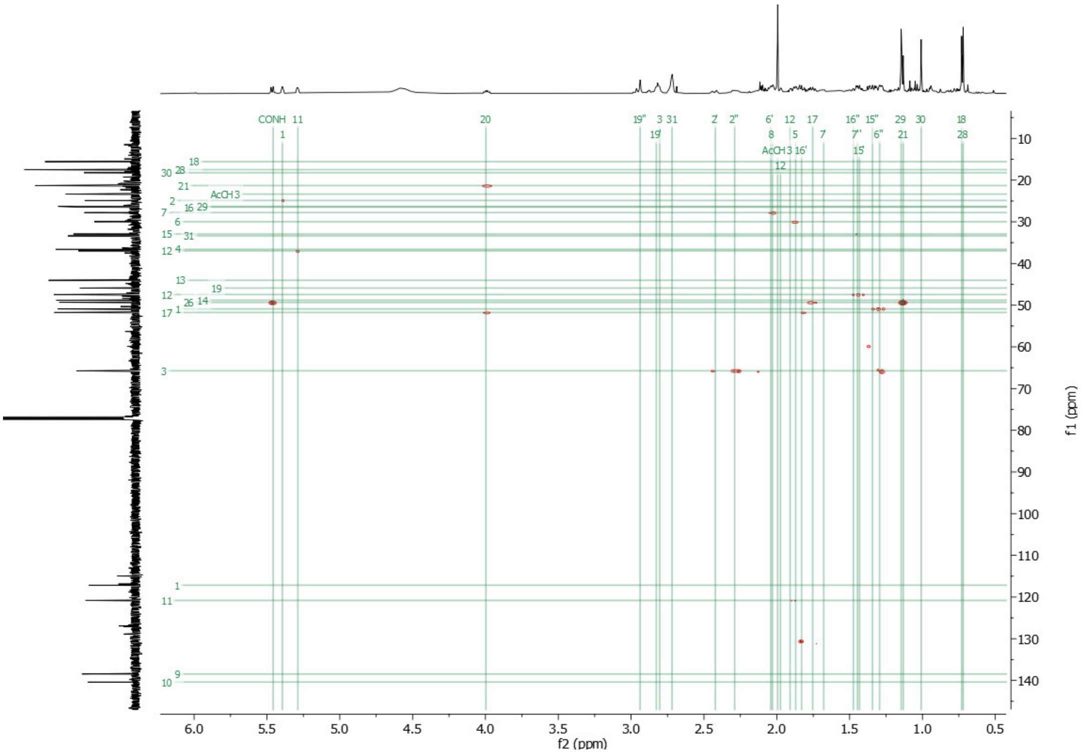

Figure S142. $^{1}H/^{13}C$ H2BC spectrum of N_{20}-acetylbuxadine-G (**24**) ($CDCl_3$, 600/150 MHz).

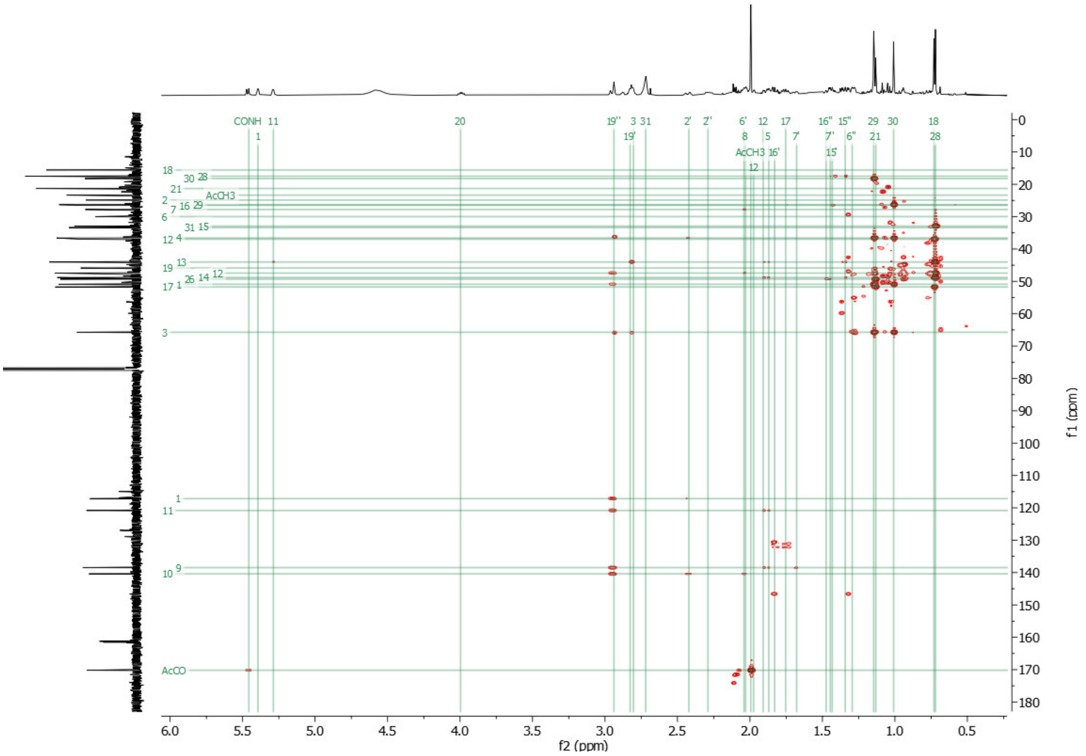

Figure S143. $^{1}H/^{13}C$ HMBC spectrum of N_{20}-acetylbuxadine-G (**24**) ($CDCl_3$, 600/150 MHz).

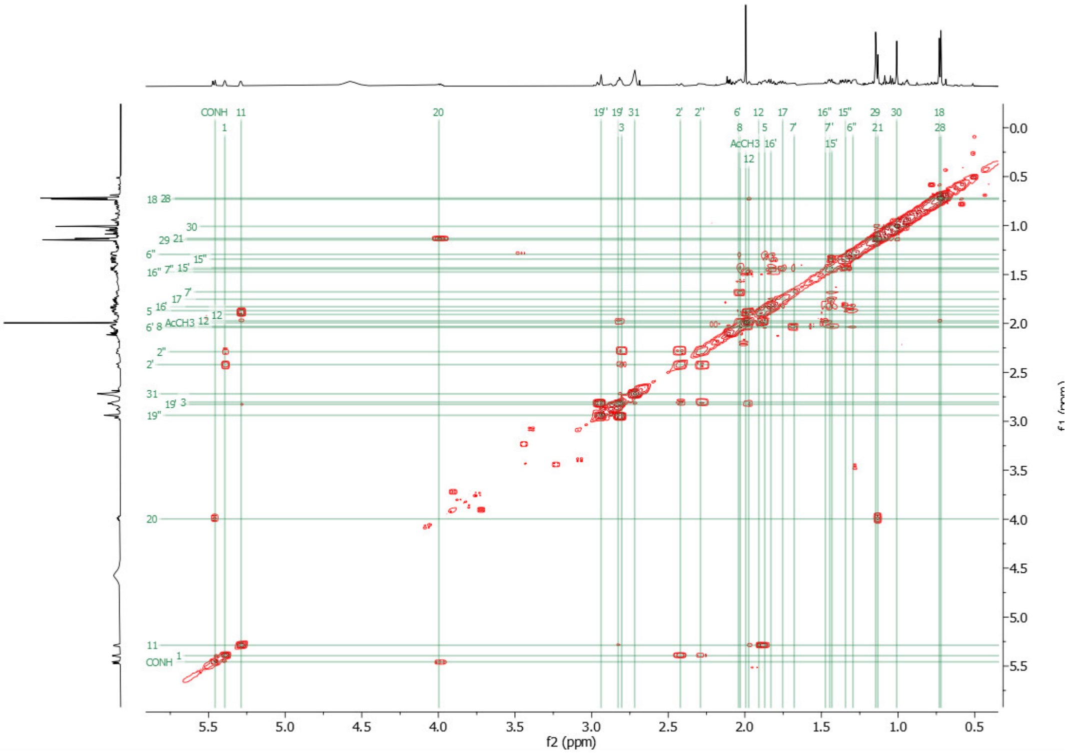

Figure S144. ^{1}H/^{1}H COSY spectrum of N$_{20}$-acetylbuxadine-G (**24**) (CDCl$_3$, 600 MHz).

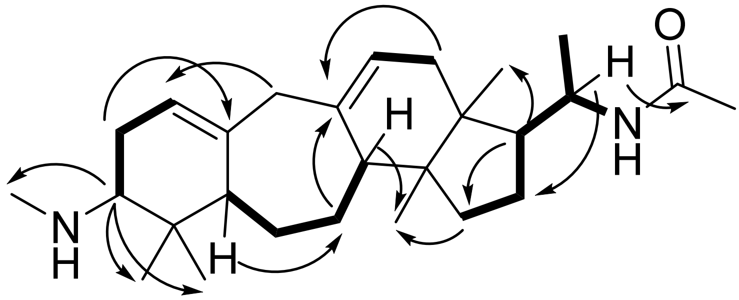

Figure S145. Key COSY (bold lines) and HMBC (arrows) correlations of N$_{20}$-acetylbuxadine-G (**24**).

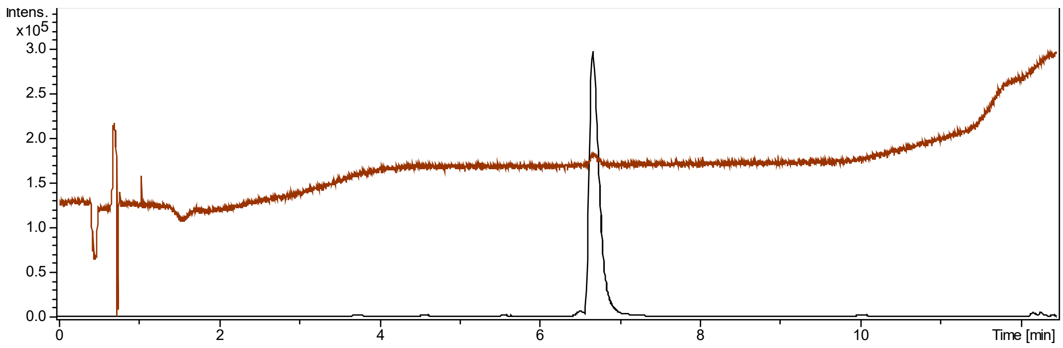

Figure S146. UHPLC/+ESI-QqTOF-MS/MS chromatogram of 17,20-dihydroxybuxadine-M (**25**). Base peak chromatogram 200.0000-1000.0000 +All MS (black); UV-Chromatogramm, 200-400 nm (red).

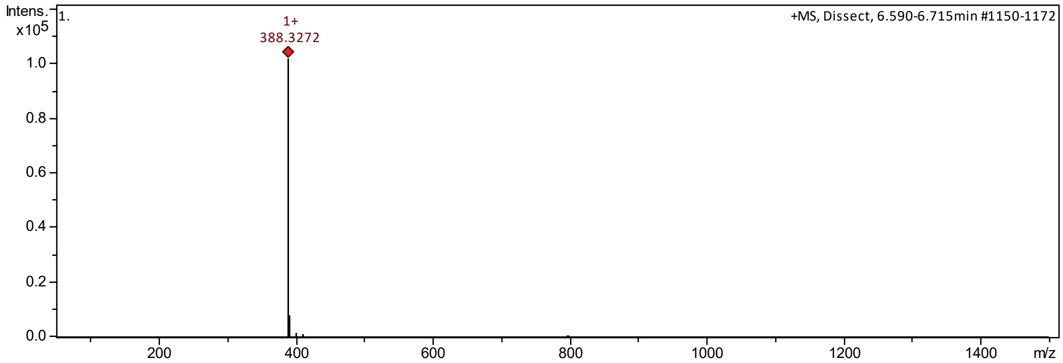

Figure S147. +ESI-QqTOF MS spectrum of 17,20-dihydroxybuxadine-M (**25**); *m*/*z* 388.3272 [M+H]$^{+}$.

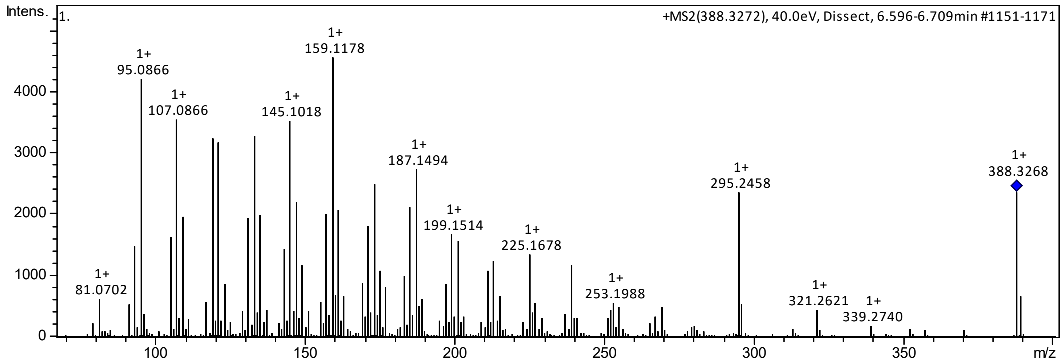

Figure S148. +ESI-QqTOF MS/MS spectrum of 17,20-dihydroxybuxadine-M (**25**).

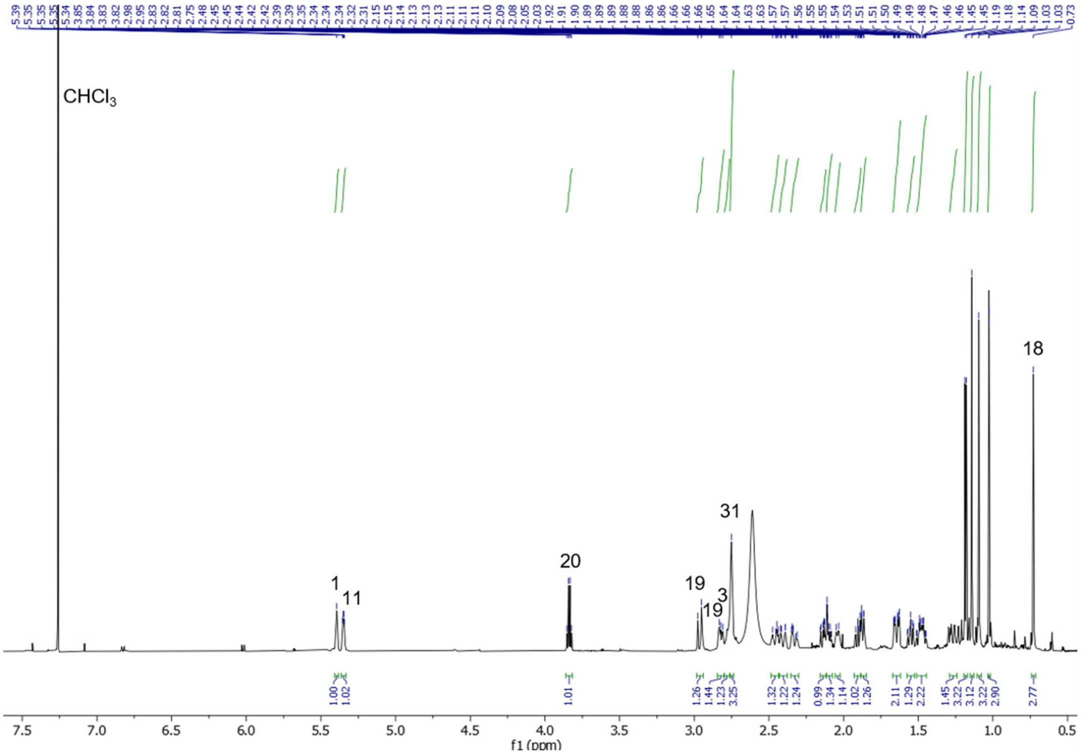

Figure S149. ^{1}H NMR spectrum of 17,20-dihydroxybuxadine-M (**25**) ($CDCl_3$, 600 MHz). The assignment of the signals between 1.0 and 2.5 ppm can be found in the enlarged Figure S150.

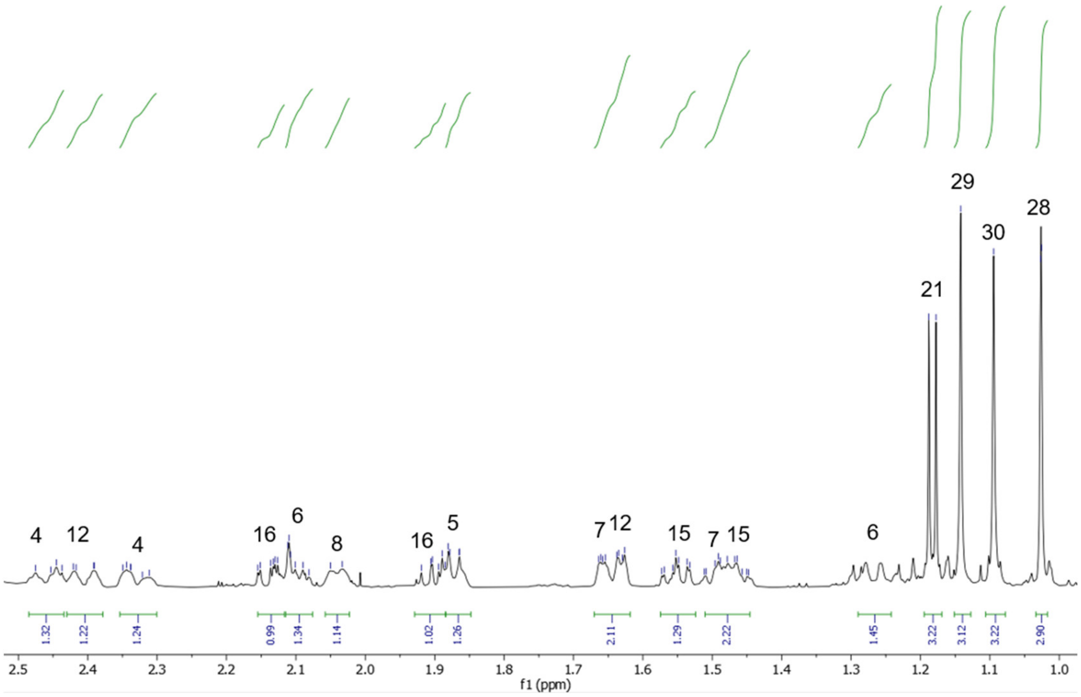

Figure S150. Detail of the ^{1}H NMR spectrum of 17,20-dihydroxybuxadine-M (**25**) ($CDCl_3$, 600 MHz).

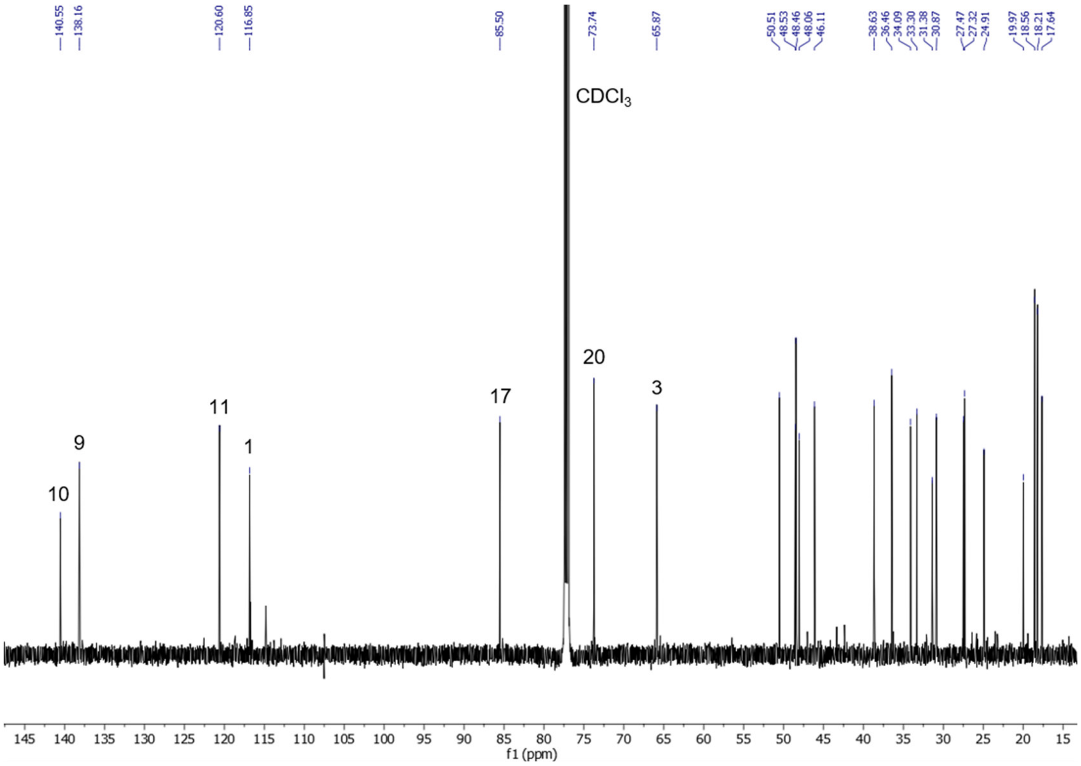

Figure S151. ^{13}C NMR spectrum of 17,20-dihydroxybuxadine-M (**25**) ($CDCl_3$, 150 MHz). The assignment of the signals between 17 and 51 ppm can be found in the enlarged Figure S152.

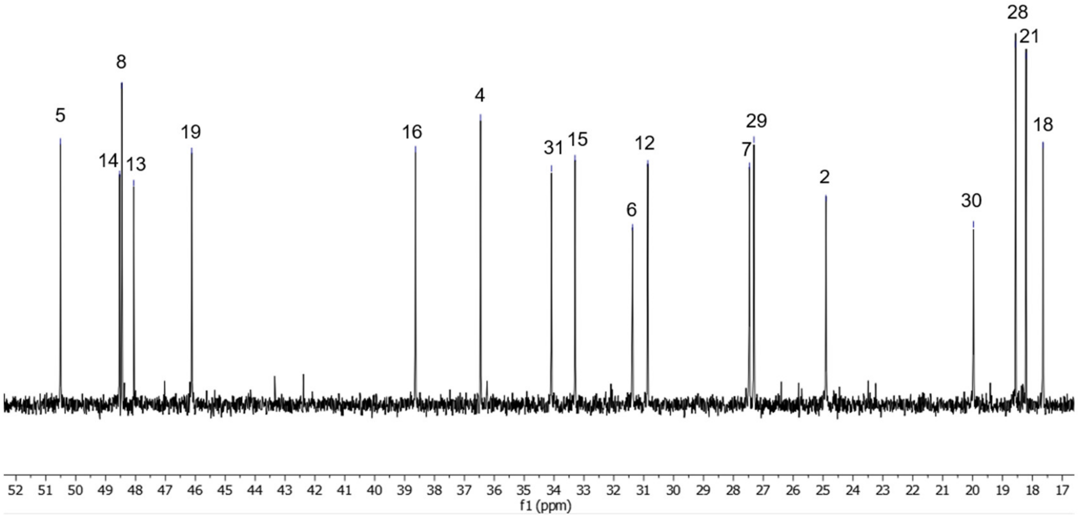

Figure S152. Detail of the ^{13}C NMR spectrum of 17,20-dihydroxybuxadine-M (**25**) (CDCl$_3$, 150 MHz).

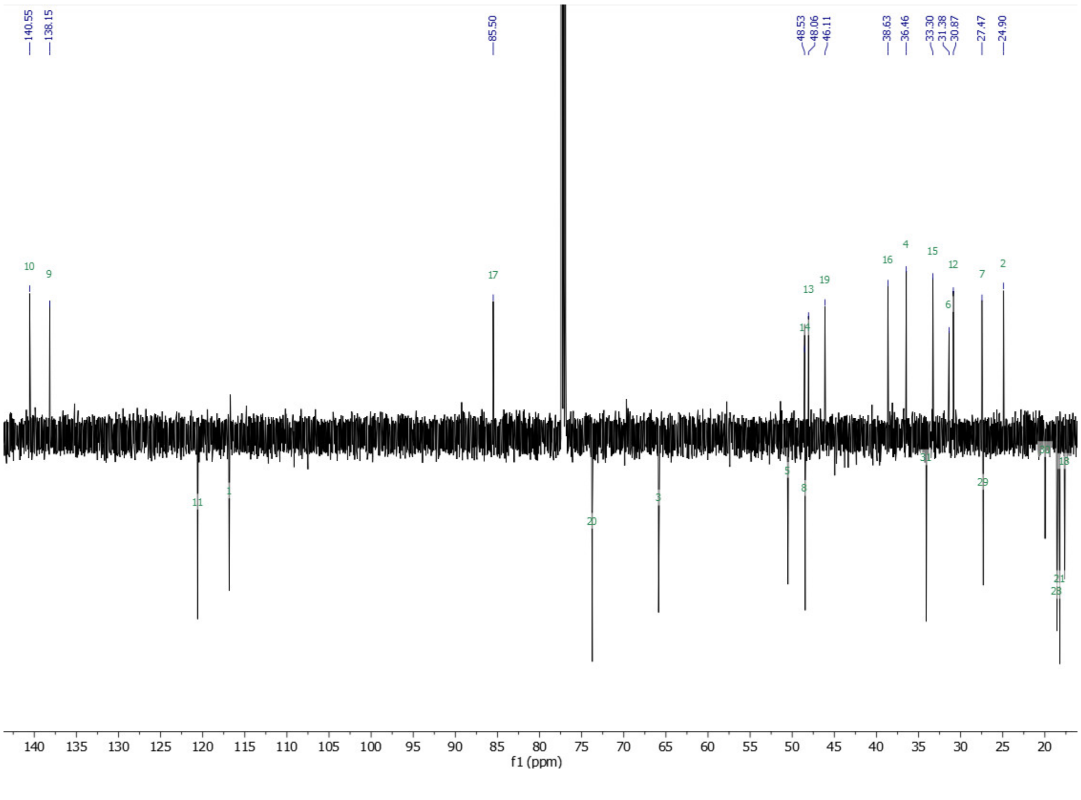

Figure S153. ^{13}C APT spectrum of 17,20-dihydroxybuxadine-M (**25**) (CDCl$_3$, 150 MHz).

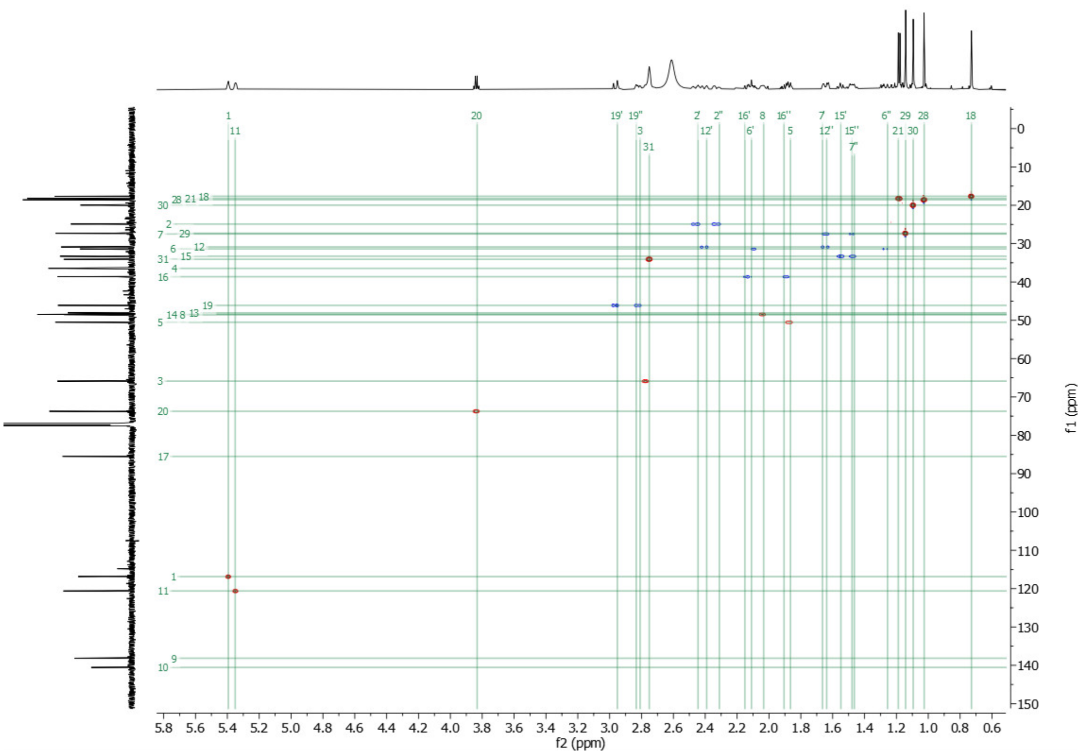

Figure S154. ^{1}H/^{13}C HSQC spectrum of 17,20-dihydroxybuxadine-M (**25**) ($CDCl_3$, 600/150 MHz).

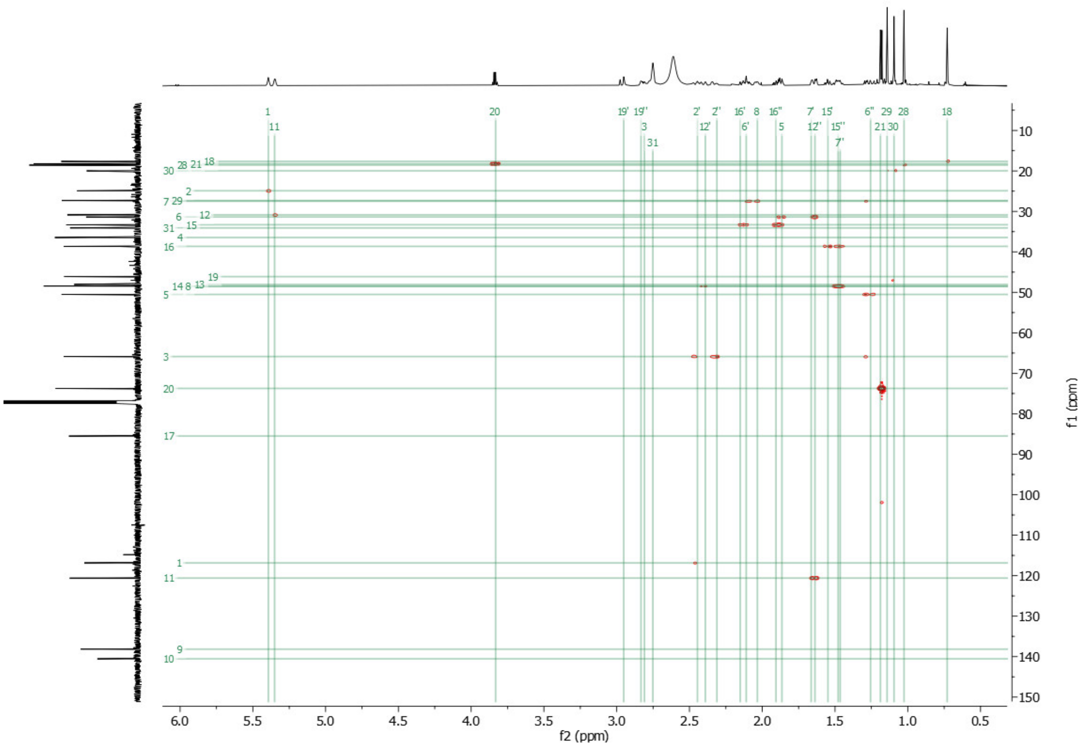

Figure S155. ^{1}H/^{13}C H2BC spectrum of 17,20-dihydroxybuxadine-M (**25**) ($CDCl_3$, 600/150 MHz).

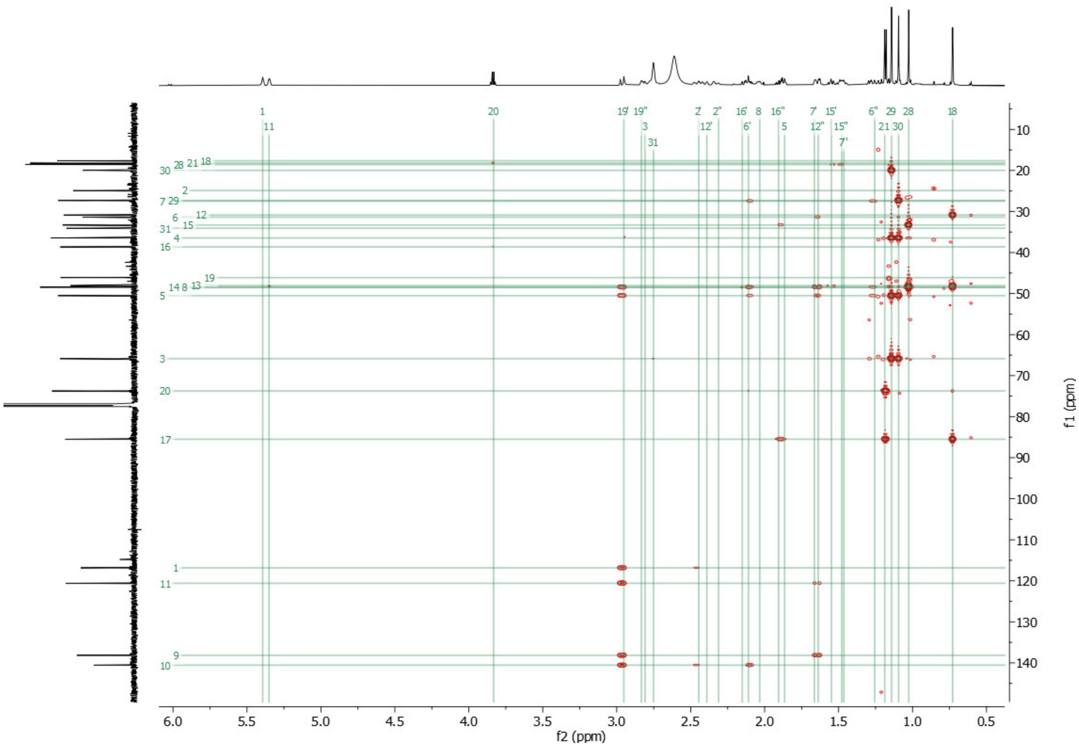

Figure S156. $^1H/^{13}C$ HMBC spectrum of 17,20-dihydroxybuxadine-M (**25**) ($CDCl_3$, 600/150 MHz).

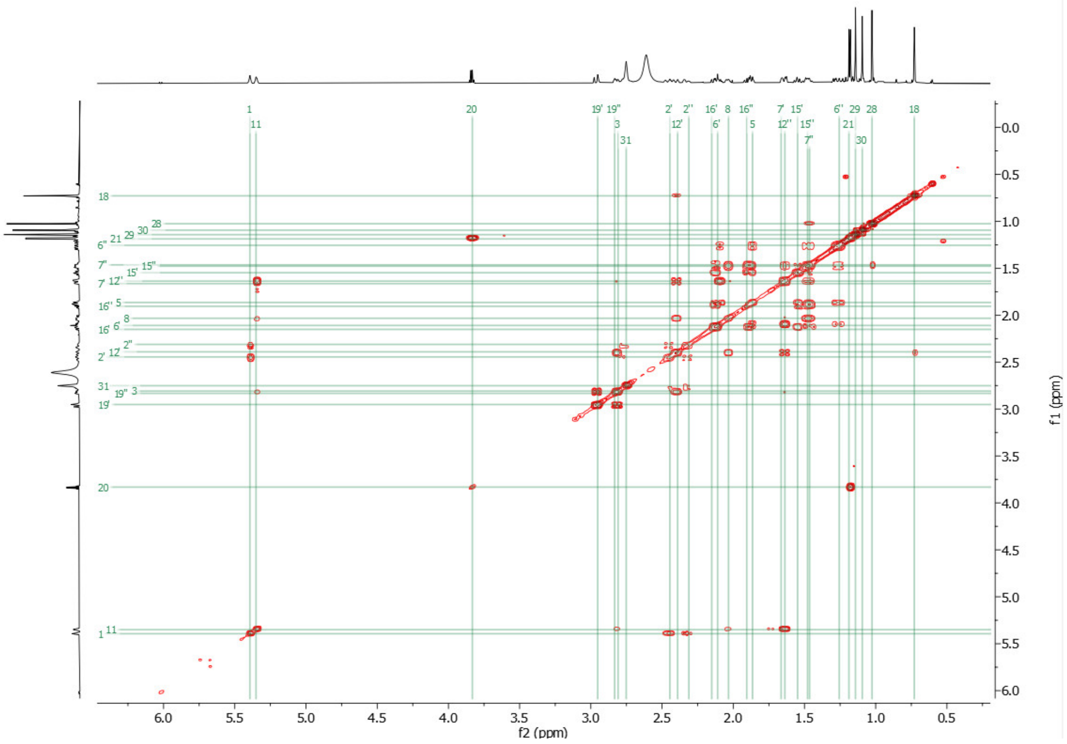

Figure S157. $^1H/^1H$ COSY spectrum of 17,20-dihydroxybuxadine-M (**25**) ($CDCl_3$, 600 MHz).

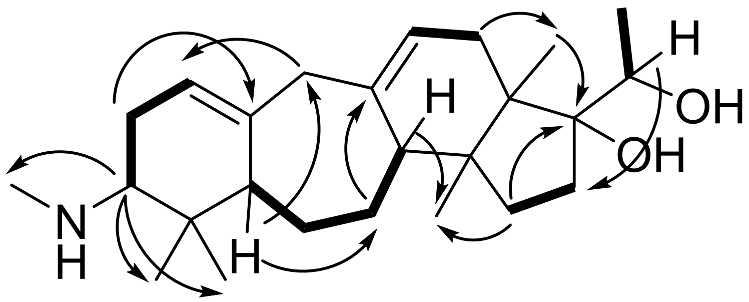

Figure S158. Key COSY (bold lines) and HMBC (arrows) correlations of 17,20-dihydroxybuxadine-M (**25**).
